# Comparative effectiveness of tirzepatide and semaglutide for obesity management in US clinical practice: a 6-month retrospective cohort study

**DOI:** 10.1007/s40618-025-02792-1

**Published:** 2026-02-09

**Authors:** Carel W. le Roux, Nicolae Done, Alan J. M. Brnabic, Abigail Zion, Ilya Lipkovich, Zbigniew Kadziola, Julia P. Dunn, Urvi Desai, Noam Kirson, Georgios K. Dimitriadis, Hong Kan

**Affiliations:** 1 University College Dublin, Conway Institute Belfield Dublin 4, Ireland and Ulster University, Cromore Road, Coleraine, Co., Londonderry, BT52 1SA UK; 2 Analysis Group, Inc, 111 Huntington Av, 14th Floor, Boston, MA 02199-7668 USA; 3 Eli Lilly and Company, Level 9, 60 Margaret St, Sydney, NSW 2000 Australia; 4 Eli Lilly and Company, 893 S Delaware St, Indianapolis, IN 46285 USA

**Keywords:** Comparative effectiveness, Obesity, Real-world evidence, Semaglutide, Tirzepatide

## Abstract

**Purpose:**

The SURMOUNT-5 trial demonstrated greater weight reduction with tirzepatide vs. semaglutide in adults with obesity without diabetes. This study compared real-world weight reduction and cardiometabolic parameters associated with tirzepatide and semaglutide for obesity management.

**Methods:**

A retrospective cohort study was conducted using Truveta de-identified US electronic health record data. Adults with obesity or overweight and ≥ 1 obesity-related complication, without diabetes, who initiated tirzepatide or semaglutide December 2023–June 2024 and adhered to treatment, were followed for 6 months. Primary outcome was percentage weight change from baseline. Secondary outcomes included weight-reduction targets and changes in body mass index (BMI) and cardiometabolic parameters. Primary analysis employed propensity-score weighted regression. Sensitivity analyses included modified intention-to-treat.

**Results:**

Among 2,396 on-treatment patients (1,003 tirzepatide; 1,393 semaglutide), greater 6-month mean percentage weight reduction was observed with tirzepatide (–11.15% vs. −8.83%; adjusted difference −2.32%-points [95% CI: −3.17, −1.48]). Higher proportions of tirzepatide-treated patients achieved 5%, 10%, 15%, and 20% weight-reduction targets. Greater reductions in BMI, blood pressure, and haemoglobin A1c were observed with tirzepatide. More patients received higher doses of semaglutide (≥ 1.7 mg; 67.7%) vs. tirzepatide (≥ 10 mg; 42.4%). Sensitivity analysis findings were consistent.

**Conclusions:**

Consistent with clinical trials, real-world tirzepatide treatment was associated with greater 6-month weight reduction and more frequent achievement of weight-reduction targets and improvements in select cardiometabolic parameters than semaglutide among adults with obesity without diabetes. This early emergence of tirzepatide’s comparative advantage over semaglutide was observed despite more semaglutide-treated patients receiving higher doses than tirzepatide-treated patients.

**Supplementary Information:**

The online version contains supplementary material available at 10.1007/s40618-025-02792-1.

## Introduction

Obesity is a chronic, progressive, relapsing disease that affects over 40% of adults in the US [1] and is associated with increased risk of morbidity, disability, mortality, and reduced quality of life [2–4]. Obesity management medications (OMMs) play an increasingly important role alongside lifestyle interventions and metabolic bariatric surgery to treat the disease of obesity [5, 6]. In particular, GLP-1 receptor agonists such as semaglutide and the glucose-dependent insulinotropic polypeptide (GIP) and glucagon-like peptide-1 (GLP-1) receptor agonist tirzepatide have demonstrated substantial efficacy for weight reduction in individuals with obesity. The SURMOUNT-5 trial, a phase 3b, head-to-head, open-label, active comparator study of 751 adults with obesity, without diabetes, demonstrated the superior efficacy of tirzepatide vs. semaglutide in percentage weight change and weight reduction targets of ≥ 10%, ≥ 15%, ≥ 20%, and ≥ 25% at 72 weeks [7]. Additionally, tirzepatide demonstrated significantly greater improvements than semaglutide at 72 weeks in cardiometabolic risk parameters, including systolic and diastolic blood pressure (SBP, DBP), haemoglobin HbA1c, fasting insulin, triglycerides, and high-density lipoprotein (HDL) cholesterol.

Despite the growing use of these medications for obesity management, to our knowledge, no real-world evaluation has compared the effectiveness of tirzepatide and semaglutide in populations with the disease of obesity, without diabetes. Existing observational studies comparing weight reduction with these two treatments were conducted in patients with and without type 2 diabetes before FDA approval of tirzepatide for obesity management [8–15].

This study aimed to address this knowledge gap by assessing the comparative effectiveness of these medications approved for obesity management among US adults with the disease of obesity, without diabetes in routine care settings.

## Methods

### Data sources

The study was conducted using the Truveta electronic health record (EHR) database, which aggregates de-identified patient data from 30 health systems across all 50 US states. The data encompass a wide range of care settings and include patient demographics, diagnoses, procedures, medications (ordered, administered, and dispensed), laboratory and vital sign measurements, and social determinants of health (e.g., education and income, when available). The dataset used in this study reflected records available as of December 13, 2024. Dates in the Truveta database are shifted by up to 30 days to protect patient privacy, and all data are de-identified in accordance with the Health Insurance Portability and Accountability Act (HIPAA) Expert Determination standard. Because this was a retrospective study using de-identified secondary data, no institutional review board approval or informed consent was required.

## Study population

The study population included adults (aged ≥ 18 years) with body mass index (BMI) ≥ 27 kg/m² with ≥ 1 obesity-related complication or BMI ≥ 30 kg/m², who initiated tirzepatide (Zepbound^®^) or semaglutide (Wegovy^®^) for obesity management between December 1, 2023, and June 30, 2024. The first recorded dispensing of either of these medications in the database was deemed the index date. Obesity-related complications were identified within the 12-month period before index date (“baseline”) (Online Resource, p. 14). Patients were further required to have ≥ 1 record of clinical activity during the 6 months after index (“follow-up”) and in each consecutive 6-month period of baseline, and ≥ 1 record of weight and/or BMI within 60 days prior to index and 30 days before or after the end of the 6-month follow-up period. In addition, patients were required to be adherent to the index treatment, with a proportion of days covered ≥ 80% during the follow-up period (*on-treatment* cohort). Patients with GLP-1 receptor agonists or dual GIP and GLP-1 receptor agonist use during the 12-month baseline period were excluded, as were those with diabetes, prior metabolic bariatric procedures, or conditions associated with unintentional weight change. See Online Resource p. 3 for additional details regarding the selection criteria and associated definitions.

## Outcomes

The primary outcome was percentage change in body weight (i.e., weight) from index to 6 months.

Additional weight-related outcomes included the percentage of patients achieving ≥ 5%, ≥ 10%, ≥ 15%, and ≥ 20% weight reduction targets, absolute weight change from index (kg), absolute change in BMI (kg/m²), and BMI class shift, defined as a transition from one BMI category to a lower one over the 6-month follow-up.

Cardiometabolic risk parameters included changes in SBP, DBP, HbA1c, and lipid parameters (i.e., total cholesterol, HDL cholesterol, low-density lipoprotein (LDL) cholesterol, and triglycerides). For each measure, absolute and percentage change from index to 6 months were reported. Analyses were restricted to patients with non-missing values at both timepoints. Definitions for study outcomes and derived measures are detailed in the Online Resource (p. 256).

## Statistical analyses

The primary analysis estimand was the average treatment effect on the treated (ATT), estimated using a propensity score (PS) weighting approach in the on-treatment cohort. Under the primary analysis method, the treatment models for generating propensity scores were generalized boosted models [16] with the treatment indicator as a binary outcome and baseline covariates including sociodemographic variables: age (continuous), sex, race, ethnicity, geographic region, education level, individual and household income; anthropometric and clinical characteristics: index weight, BMI, BMI class (overweight, obesity class I–III), and comorbidities (see Online Resource for full list and definitions); healthcare system interactions: number of medical encounters, prescriptions, and laboratory assessments; and use of non-incretin OMMs (i.e., phentermine, phentermine/topiramate, bupropion/naltrexone). ATT weights were set to 1 for tirzepatide-treated patients and set to PS/(1–PS) for semaglutide-treated patients. Covariate balance was assessed using absolute standardized mean differences (ASMDs), with values < 0.1 considered acceptable, and variance ratios, with values between 0.5 and 2.0 considered acceptable.

Weighted treatment effect estimates were generated using generalized linear models (GLMs) with robust standard errors. For continuous outcomes (i.e., percent and absolute change in weight, BMI, and change in cardiometabolic parameters), results were reported as adjusted least squares mean differences (LSMDs) with 95% confidence intervals (CIs). For binary outcomes (i.e., ≥ 5%, ≥ 10%, ≥ 15%, or ≥ 20% weight reduction; BMI class shift), results were reported as adjusted odds ratios (aORs) with 95% CIs calculated using observed margins. In addition to PS weighting, outcome models were adjusted further using the following prespecified baseline covariates to help mitigate residual confounding: age, sex, race, ethnicity, value of the outcome, number of obesity-related comorbidities, and baseline use of non-incretin OMMs.

The main analyses were conducted using SAS 9.4 (SAS Institute Inc., Cary, NC, USA) and R Version 4.2.3 (R Foundation for Statistical Computing, Vienna, Austria). A list of R packages used for the analyses is provided in the Online Resource (p. 6).

## Handling of missing data

For all outcomes, patients were required to have non-missing values at both baseline and 6-month follow-up to be included in the analysis for that outcome. For missing weight, values were derived using BMI and height, as available. For missing BMI, values were derived using height and weight, as available. No imputation was applied for other outcome measures. When multiple values were available within the allowable baseline or follow-up windows, the closest valid value to the target timepoint was used, and implausible values were excluded based on predefined rules (Online Resource, p. 4).

For cardiometabolic parameters, all analyses were conducted within subsets with non-missing outcome data, with PS weights re-estimated in those subsets.

### Sensitivity analyses

Prespecified sensitivity analyses were conducted to evaluate the robustness of the primary outcome analyses, i.e., percentage weight change and other select additional outcomes and their sensitivity to alternative methodological assumptions and potential sources of bias.

The primary analysis was repeated for a broader modified intention-to-treat (*mITT*) cohort, which included patients regardless of adherence or switching status but required all other selection criteria applied to the primary cohort, including having non-missing baseline and 6-month outcome data. This analysis estimated the treatment effect of therapy initiation, regardless of subsequent treatment choices.

A Frequentist Model Averaging (FMA) analysis [17] was conducted to evaluate the robustness of the primary analysis method. FMA incorporated 11 alternative analytic strategies combining different treatment models (e.g., Least Absolute Shrinkage and Selection Operator [LASSO] regression, gradient boosting) and outcome models (e.g., GLMs, LASSO regression) to derive final estimates as weighted averages across models (details in Online Resource, p. 10).

An analysis based on principal stratification approach [18] was conducted in the mITT cohort to estimate the effect of tirzepatide vs. semaglutide in a hypothetical population of patients who would be adherent to tirzepatide if assigned to it (irrespective of actual treatment assignment), enabling a comparison that isolates the treatment effect in the subgroup most likely to adhere to tirzepatide therapy (details in Online Resource, p. 12).

Robustness of the treatment effect estimates was also assessed using the E-value [19], which quantifies the minimum strength of association on the risk ratio (RR) scale that an unmeasured confounder would need to have with both the treatment and the outcome to fully explain away the treatment effect, accounting for all observed confounders (details in Online Resource, p. 13).

## Role of the funding source

This study was funded by Eli Lilly and Company, which conceived the study idea, provided access to the data, and led the overall study design and interpretation. Primary and secondary analyses were conducted by Analysis Group, Inc. under contract with Eli Lilly and Company. Additional sensitivity analyses including FMA were conducted independently by Eli Lilly and Company.

Authors affiliated with Eli Lilly and Company, Analysis Group, Inc., and University College Dublin contributed to study design, statistical analysis, data interpretation, and drafting and critical revision of the manuscript. All authors had full access to the final study outputs and take responsibility for the decision to submit the manuscript for publication.

## Results

### Baseline characteristics

**Fig. 1:**
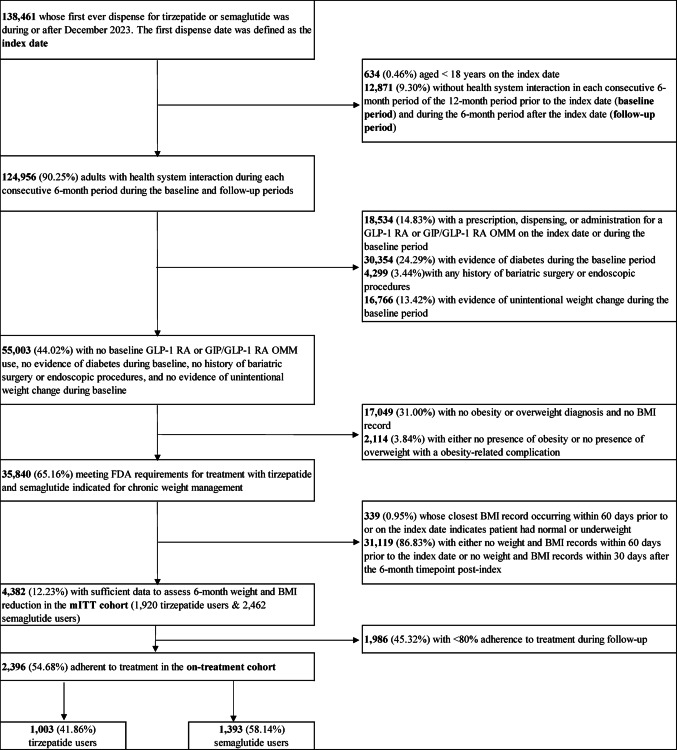
Cohort construction flowchart. BMI = body mass index; FDA = Food & Drug Administration; GIP = glucose-dependent insulinotropic peptide; GLP-1 = glucagon-like peptide 1; OMM = obesity management medication; RA = receptor agonist

A total of 2,396 patients met criteria for the on-treatment cohort, including 1,003 patients treated with tirzepatide and 1,393 treated with semaglutide (Fig. 1).

The baseline demographic and clinical characteristics of the two treatment groups before and after PS weighting are presented in Table 1. Briefly, patients in the tirzepatide and semaglutide groups had a mean age of 48.4 and 49.1 years, respectively. More patients in the semaglutide group were female (73.2%) and Black (18.0%) compared with those treated with tirzepatide (69.9% female; 11.5% Black, ASMD = 0.072 and 0.186, respectively). Conversely, more patients in the tirzepatide group had a college degree (60.8%) compared with semaglutide (57.9%, ASMD = 0.059). At baseline, the average weight and BMI in the tirzepatide group were approximately 104.0 kg and 38.3 kg/m^2^ vs. 104.6 kg and 38.4 kg/m^2^ in the semaglutide group, respectively. However, slightly more patients in the semaglutide group had Class III obesity (BMI ≥ 40 kg/m^2^) compared with the tirzepatide group (35.7% vs. 32.9%, ASMD = 0.059). The rates of various obesity-related complications and other comorbidities assessed in the study were generally similar between the two groups (ASMD < 0.1).

**Table 1:** Demographic and clinical characteristics of patients treated with Tirzepatide and semaglutide in the on-treatment cohort, before and after adjustment

Characteristic	Tirzepatide *N* = 1,003	Before PS Weighting		After PS Weighting	
		Semaglutide *N* = 1,393	ASMD	Semaglutide *N* = 1,393 ESS = 1,202^a^	ASMD
**Age on index (years)**,** mean (SD)**	48.4 (12.2)	49.1 (11.9)	0.064	48.3 (11.8)	0.004
**Female**	69.9%	73.2%	0.072	71.1%	0.027
**Race**					
White	75.2%	68.6%	0.146	73.9%	0.029
Black	11.5%	18.0%	0.186	12.4%	0.028
Asian	1.6%	1.4%	0.013	1.6%	0.002
American Indian or Alaska Native	4.9%	0.6%	0.020	5.5%	0.014
Native Hawaiian or Other Pacific Islander	6.9%	0.3%	0.043	6.7%	0.049
Other	75.2%	4.7%	0.018	73.9%	0.014
Unknown	11.5%	6.3%	0.023	12.4%	0.009
**Ethnicity**					
Not Hispanic or Latino	82.5%	81.8%	0.018	82.4%	0.025
Hispanic or Latino	11.3%	12.3%	0.034	12.1%	0.027
Unknown	6.3%	5.9%	0.017	5.5%	0.003
**US Census region**					
South	42.3%	43.6%	0.026	44.9%	0.027
West	14.7%	15.9%	0.034	14.4%	0.035
Midwest	13.4%	12.2%	0.035	12.1%	0.037
Northeast	2.3%	0.6%	0.137	1.1%	0.088
Unknown	27.4%	27.7%	0.007	27.4%	0.004
**Education level**					
College degree	60.8%	57.9%	0.059	59.6%	0.006
No college	22.7%	27.6%	0.112	24.7%	0.010
Some college	0.0%	0.0%	0.000	0.0%	0.000
Unknown	16.5%	14.5%	0.054	15.7%	0.020
**Income level (USD)**					
0–25k	0.5%	0.6%	0.020	0.5%	0.009
25–50k	15.2%	19.3%	0.110	16.3%	0.024
50–100k	36.8%	31.4%	0.113	35.3%	0.034
100–150k	10.1%	9.8%	0.010	9.3%	0.013
≥150k	0.5%	0.3%	0.034	0.3%	0.023
Unknown	37.0%	38.5%	0.032	38.2%	0.028
**Household income level (USD)**					
0–25k	0.1%	0.6%	0.090	0.3%	0.041
25–50k	6.4%	10.0%	0.132	6.9%	0.020
50–100k	47.9%	43.1%	0.095	47.1%	0.015
100–150k	4.8%	5.0%	0.008	4.9%	0.006
≥150k	0.0%	0.0%	0.000	0.0%	0.000
Unknown	40.9%	41.3%	0.008	40.8%	0.001
**Weight (kg)**,** mean (SD)**	104.0 (26.0)	104.0 (26.0)	0.021	104.4 (24.8)	0.013
**BMI (kg/m** ^**2**^ **)**,** mean (SD)**	38.3 (7.2)	38.3 (7.2)	0.018	38.1 (6.8)	0.024
**BMI class**					
Class 3 obesity (BMI ≥ 40 kg/m^2^)	32.9%	35.7%	0.059	33.0%	0.001
Class 2 obesity (BMI ≥ 35 and < 40 kg/m^2^)	28.0%	27.7%	0.007	28.6%	0.014
Class 1 obesity (BMI ≥ 30 and < 35 kg/m^2^)	33.0%	29.8%	0.069	32.5%	0.010
Overweight (BMI ≥ 25 and < 30 kg/m^2^)	6.1%	6.8%	0.030	5.9%	0.009
**Number of obesity-related complications**	1.5 (1.3)	1.6 (1.3)	0.064	1.5 (1.3)	0.004
Dyslipidaemia	48.3%	47.2%	0.022	46.7%	0.030
Hypertension	44.1%	47.5%	0.069	45.6%	0.030
Prediabetes	16.3%	19.7%	0.091	17.0%	0.019
Obstructive sleep apnea	16.8%	17.7%	0.023	16.9%	0.000
Osteoarthritis knee and/or hip	8.2%	9.5%	0.048	8.5%	0.012
Atherosclerotic cardiovascular disease	8.2%	8.9%	0.026	8.2%	0.000
Metabolic dysfunction-associated steatotic liver disease/steatohepatitis	5.9%	6.2%	0.012	6.3%	0.016
Metabolic syndrome	2.5%	1.8%	0.048	1.9%	0.043
Chronic heart failure - HFpEF	0.5%	0.3%	0.034	0.2%	0.053
**Number of other comorbidities**,** mean (SD)**	2.2 (1.7)	2.3 (1.8)	0.052	2.1 (1.7)	0.030
**Use of any non-incretin OMM**	11.0%	12.3%	0.041	11.5%	0.016

OMM = obesity management medication; ASMD = absolute standardized mean difference; BMI = body mass index; ESS = effective sample size; HFpEF = Heart failure with preserved ejection fraction; kg = kilograms; m = meters; PS = propensity score; SD = standard deviation; USD = United States dollars

^a^Effective sample size is calculated to account for variability in ATT weights [25]

**Fig. 2:**
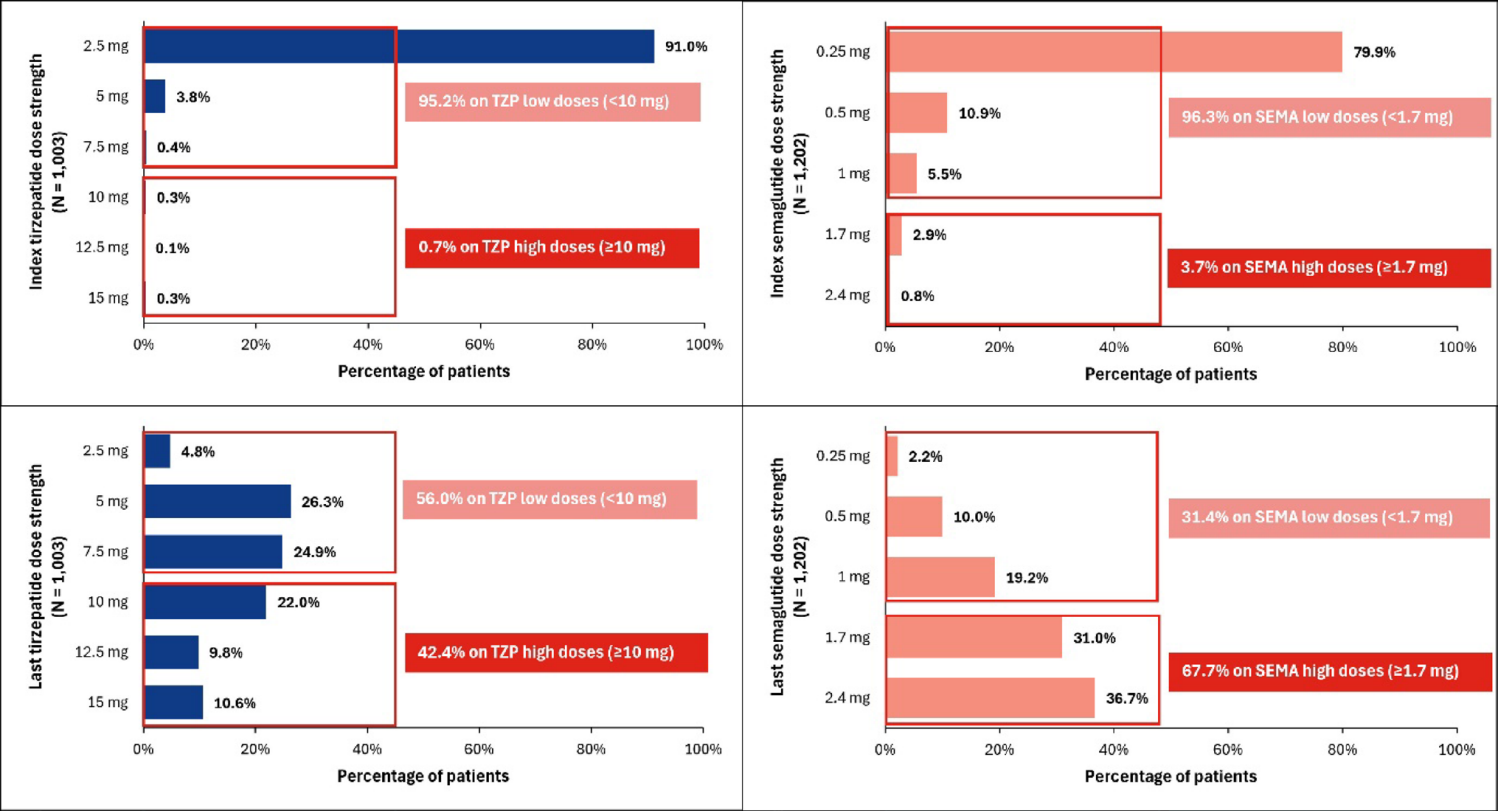
Dosage for the index fill and last observed fill in the 6-month follow-up period in patients treated with tirzepatide and semaglutide in the on-treatment cohort, after PS weighting. ESS = effective sample size; PS = propensity score; SEMA = semaglutide; TZP = tirzepatide. Percentages do not sum to 100% due to small percentages with unknown dose strength values

Patients initiating tirzepatide were most frequently started on the lowest available dose (91.0% at 2.5 mg) (Fig. 2) [20, 21]. In comparison, 79.9% of semaglutide initiators started on the lowest dose of 0.25 mg. By month 6, fewer tirzepatide patients received the highest available doses (≥ 10 mg) compared to semaglutide (≥ 1.7 mg) (42.4% vs. 67.7%).

The ASMDs for all characteristics were less than 0.1 (Table 1) and variance ratios for all continuous covariates were close to 1.0 after PS weighting (Online Resource p. 257), suggesting good balance was achieved between the two treatment groups. The PS distribution before and after adjustment and the PS weights distribution are presented in the Online Resource (p. 271–272).

### Weight reduction outcomes

**Fig. 3:**
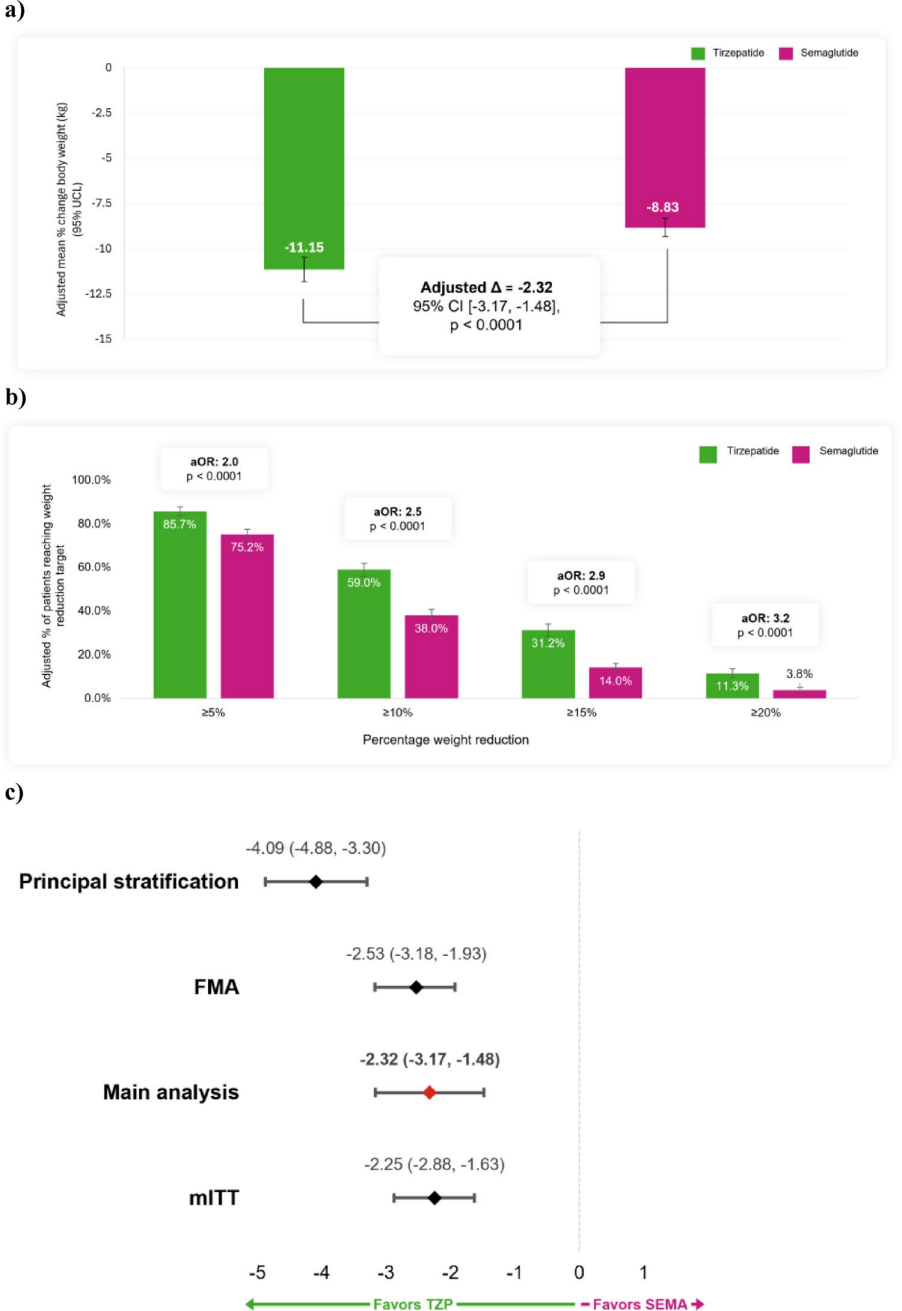
Adjusted 6-month mean percentage weight change in patients treated with tirzepatide and semaglutide and adjusted difference, primary analysis method (**a**); adjusted odds ratios for categorical 6-month weight reductions, primary analysis method (**b**); adjusted mean percentage weight change, sensitivity analyses (**c**). ∆ = change; aOR = adjusted odds ratio; ATT = average effect of treatment on the treated; CI = confidence interval; FMA = frequentist model averaging; LCL = lower confidence limit; mITT = modified intention-to-treat; PS = propensity score; SEMA = semaglutide; TZP = tirzepatide; UCL = upper confidence limit. Estimates in (**a**) and (**b**) were adjusted using ATT PS weights and multivariate regression modeling (primary analysis method). Estimates in (**c**) were obtained using each sensitivity analysis as indicated

In the primary on-treatment analysis, tirzepatide-treated patients had an adjusted percentage weight change of −11.15% (95% CI −11.82 to −10.48) compared to −8.83% (95% CI −9.33 to −8.33) among the semaglutide-treated patients, with an adjusted mean difference of −2.32% points (95% CI −3.17 to −1.48) in favour of tirzepatide (Fig. 3, Panel A). At 6 months, 85.7% of tirzepatide-treated patients achieved ≥ 5% weight reduction, compared to 75.2% in the semaglutide-treated group (aOR 2.03 [95% CI 1.63 to 2.54]). Greater percentages of tirzepatide-treated vs. semaglutide-treated patients also achieved ≥ 10% weight reduction (59.0% vs. 38.0%, aOR 2.46 [2.06 to 2.93]), ≥ 15% weight reduction (31.2% vs. 14.0%, aOR 2.88 [2.32 to 3.57]), and ≥ 20% weight reduction (11.3% vs. 3.8%, aOR 3.23 [2.29 to 4.55]) (all *p* < 0.0001) (Fig. 3, Panel B).

The 6-month adjusted mean absolute change in weight was −12.36 kg (95% CI −12.90 to −11.82) among tirzepatide-treated patients and −9.45 kg (95% CI −9.89 to −9.01) among semaglutide-treated patients, yielding an adjusted mean difference of −2.91 kg (95% CI −3.61 to −2.22; *p* < 0.0001) in favour of tirzepatide (Table 2).

**Table 2:** Adjusted absolute changes in weight, BMI, and cardiometabolic risk parameters over the 6-month follow-up

	*N*		Baseline Mean		6-Month Mean		Adjusted Mean Change		Adjusted Mean Difference^a^ [95% CI]	*P*-value
Outcome	TZP	SEMA	TZP	SEMA	TZP	SEMA	TZP	SEMA		
Weight, kg	1,003	1,393	103.6	103.9	93.5	96.4	−12.36	−9.45	**−2.91** **[−3.61**,** −2.22]**	< 0.0001*
BMI, kg/m^2^	1,003	1,393	38.3	38.1	33.2	34.3	−5.12	−3.86	**−1.25** **[−1.50**,** 1.00]**	< 0.0001*
Systolic blood pressure, mmHg	798	1,173	127.7	127.4	120.2	121.8	−7.32	−5.90	**−1.42** **[−2.50**,** −0.35]**	0.0097*
Diastolic blood pressure, mmHg	800	1,178	80.0	79.7	76.4	77.5	−3.51	−1.99	**−1.52** **[−2.29**,** −0.75]**	0.0001*
HbA1c, percent	69	106	5.7	5.7	5.2	5.4	−0.52	−0.37	**−0.15** **[−0.24**,** −0.05]**	0.0042*
Total cholesterol, mg/dL	62	110	191.0	192.3	172.7	171.8	−19.39	−22.49	3.10 [−6.26, 12.46]	0.5134
LDL cholesterol, mg/dL	27	47	124.6	130.9	108.9	111.7	−17.52	−19.14	1.62 [−8.96, 12.19]	0.7609
HDL cholesterol, mg/dL	69	119	50.9	50.4	51.5	49.6	0.35	−1.86	2.21 [−1.08, 5.49]	0.1861
Triglycerides, mg/dL	78	137	153.0	147.0	113.7	118.1	−37.17	−31.67	−5.50 [−19.90, 8.90]	0.4522

BMI = body mass index; CI = confidence interval; dL = decilitres; HbA1lc = haemoglobin A1c; HDL = high-density lipoprotein; kg = kilograms; LDL = low-density lipoprotein; m = meters; mg = milligrams; mmHg = millimetres of mercury; SEMA = semaglutide; TZP = tirzepatide

* Indicates statistical significance (*p* < 0.05)

^a^Estimates represent adjusted differences in mean changes from baseline to 6 months between the tirzepatide and semaglutide arms

In parallel with weight changes, the 6-month adjusted mean absolute change in BMI was −5.12 kg/m² (95% CI −5.30 to −4.94) in tirzepatide-treated patients vs. −3.86 kg/m² (95% CI −4.04 to −3.69) in semaglutide-treated patients, yielding an adjusted difference of −1.25 kg/m² (95% CI −1.50 to −1.00; *p* < 0.0001) in favour of tirzepatide (Table 2). BMI class shift also favoured tirzepatide. A significantly higher percentage of patients receiving tirzepatide achieved ≥ 1 reduction in BMI category compared with those receiving semaglutide (68.8% vs. 54.8%; aOR: 1.92; 95% CI 1.59 to 2.32; see Online Resource p. 267).

### Cardiometabolic risk parameters

Cardiometabolic risk parameters were assessed in on-treatment subsets with non-missing values at baseline and 6-month follow-up, ranging from 74 patients (LDL cholesterol) to 1,978 patients (DBP) (Table 2). Tirzepatide was associated with greater improvements in mean DBP (−3.51 mmHg vs. −1.99 mmHg; adjusted difference −1.52, 95% CI −2.29 to −0.75), mean SBP (−7.32 mmHg vs. −5.90 mmHg; difference −1.42, 95% CI −2.50 to −0.35), and mean HbA1c (−0.52% vs. −0.37%; difference −0.15, 95% CI −0.24 to −0.05) compared to semaglutide. These findings were directionally consistent across both percentage and absolute change analyses. Adjusted percentage changes in lipid parameters were not different between treatment groups. Full results are reported in the Online Resource (p. 266).

### Sensitivity analyses

In the mITT cohort, tirzepatide was associated with significantly greater weight reduction than semaglutide (Fig. 3, Panel C). The adjusted mean percentage weight change was −9.19% (95% CI −9.68 to −8.70) for tirzepatide vs. −6.94% (95% CI −7.33 to −6.55) for semaglutide, with an adjusted difference of − 2.25% points (95% CI −2.88 to −1.63).

Results from the sensitivity analyses using FMA and principal stratification in the on-treatment cohort were consistent with the primary analysis and are reported in Fig. 3 (Panel C) as well as in the Online Resource (p. 275). Across analytic strategies, the estimated differences in weight change between cohorts ranged from −2.53 (95% CI −3.18 to −1.93) to −4.09 (95% CI −4.88 to − 3.30) percentage points in favour of tirzepatide. The E-value analysis indicated that an unmeasured confounder would need an extremely strong association (RR > 16) with treatment selection and percentage weight change to nullify the observed advantage of tirzepatide over semaglutide (Online Resource p. 280). Even to reduce treatment effect to the lower confidence bound, a substantial confounder (RR > 7) would be required.

Achievement of categorical weight reduction targets in the mITT cohort similarly favoured tirzepatide. The adjusted percentage of patients achieving ≥ 5% weight reduction was 74.4% with tirzepatide and 61.7% with semaglutide (aOR: 1.80; 95% CI 1.57 to 2.07). The adjusted rates were 45.5% and 29.4%, respectively (aOR: 2.01; 95% CI 1.76 to 2.29) for ≥ 10% weight reduction; 23.3% vs. 10.8%, respectively (aOR: 2.51; 95% CI: 2.11 to 3.00) for ≥ 15% weight reduction; and 9.4% vs. 3.0%, respectively (aOR: 3.38; 95% CI 2.51 to 4.54) for ≥ 20% weight reduction (Online Resource p. 269–270).

## Discussion

In this large real-world comparative effectiveness study of adults without diabetes initiating pharmacotherapy for obesity management, tirzepatide was associated with greater reductions in weight and BMI, and a higher likelihood of achieving weight reduction targets of ≥ 5%, ≥ 10%, ≥ 15%, and ≥ 20% at 6 months compared with semaglutide. Although the adjusted mean difference in weight reduction between treatments at 6 months was moderate in absolute terms, categorical outcomes reflect its strong clinical importance: for example, approximately twice as many tirzepatide-treated patients achieved ≥ 15% weight reduction compared with semaglutide-treated patients (31% vs. 14%). These differences were robust across multiple population specifications and analytic approaches. Tirzepatide was also associated with greater improvements in mean systolic and diastolic blood pressure and HbA1c compared to semaglutide, though differences in lipid parameters were similar.

These real-world findings complement and extend results from randomized controlled trials of tirzepatide and semaglutide for obesity management. In the SURMOUNT-1 trial, patients without diabetes treated with tirzepatide 15 mg achieved a mean weight reduction of 16.0% at 72 weeks [22], while semaglutide 2.4 mg in the STEP-1 trial yielded a 14.9% reduction over a similar period (treatment regimen estimand) [23]. Recently reported results using the efficacy estimand from SURMOUNT-5, a direct comparison of tirzepatide and semaglutide in adults with obesity, found a 21.6% weight reduction with tirzepatide vs. 15.4% with semaglutide at 72 weeks, confirming the superior efficacy of tirzepatide in a randomized setting [7]. In SURMOUNT-5, at 24 weeks, tirzepatide 10 or 15 mg led to an estimated mean percentage weight reduction of 14.4% (95% CI −15.0% to −13.8%) compared with 10.8% (95% CI –11.4% to −10.2%) for semaglutide 1.7 or 2.4 mg (efficacy estimand). The current study provides support for these findings in a real-world clinical setting, demonstrating that tirzepatide was associated with greater weight reduction than semaglutide in a patient population treated for obesity management—rather than for glycaemic control.

Importantly, the magnitude of weight reduction observed in the current 6-month analysis (−11.15% with tirzepatide vs. −8.83% with semaglutide in the on-treatment cohort) aligns closely with interim timepoints in these pivotal trials and supports the early emergence and clinical meaningfulness of the comparative advantage of tirzepatide over semaglutide. The achievement of weight-reduction targets was also more common with tirzepatide than semaglutide, further aligning with SURMOUNT-5 findings. Moreover, despite more semaglutide-treated patients starting at doses higher than the minimal dose and reaching the higher approved maintenance doses (i.e., ≥ 10 mg for tirzepatide and ≥ 1.7 mg for semaglutide) during follow-up, weight reduction remained greater in the tirzepatide group. Given identical follow-up duration and labeled 4-week titration permitting escalation to ≥ 10 mg (tirzepatide) and ≥ 1.7 mg (semaglutide) by week 16, this difference likely reflects clinical titration patterns and differences in clinical effectiveness between the two treatments rather than limited time to escalate. Taken together, our findings support the external validity of trial-based evidence and highlight the real-world effectiveness of tirzepatide in US clinical practice.

The results of this study are also consistent with previous real-world studies that compared the effectiveness of tirzepatide vs. semaglutide for weight reduction, using data from time periods prior to the approval of tirzepatide for obesity management. In particular, using the same data source but from May 2022 to September 2023 instead, Rodriguez et al. (2024) found that among the subpopulation without type 2 diabetes, patients treated with tirzepatide had greater mean percentage weight reduction at 6 months compared to similar patients treated with semaglutide, with a difference of approximately 4.5% points [8]. Trinh et al. (2025) found that patients without diabetes lost an average of 7.0% of their weight over 6 months if treated with tirzepatide compared to 3.4% if treated with semaglutide, but the study did not analyse dose or titration schedules [9]. Several observational studies have reported greater 12-month weight loss with tirzepatide than semaglutide approved for the treatment of type 2 diabetes, but differences in follow-up duration, dosing, and adherence limit comparability with shorter-term outcomes [12, 13]. Other retrospective cohort studies found similar results on weight reduction in populations with conditions other than obesity [10, 11, 14, 15].

This study has several strengths. First, we leveraged a large, geographically diverse real-world database spanning multiple US health systems, enabling the evaluation of treatment effectiveness in a clinically relevant population of adults treated specifically for obesity management—rather than glycaemic control. The analysis employed a robust causal inference framework, multiple analytic strategies under an FMA framework, and extensive sensitivity analyses such as principal stratification and E-value estimation. These methods collectively enhance the internal validity of the findings, attempt to mitigate observed confounding, as well as help understand the impact of confounding due to unmeasured factors. The inclusion of both continuous and categorical weight reduction measures, BMI measures, and cardiometabolic parameters provides a comprehensive overview of comparative treatment effectiveness. The inclusion of both an on-treatment cohort and an mITT cohort further enhances the robustness and generalizability of findings by capturing both treatment-adherent patients and the broader target population initiating therapy.

This study also has limitations. As with all observational research, residual confounding cannot be fully excluded despite extensive covariate adjustment and sensitivity analyses. The analysis was limited to a 6-month follow-up period, which may not capture the full magnitude or durability of treatment effects, as clinical trial data suggest that weight reduction typically continues beyond 6 months and often does not plateau until approximately 36 weeks or later, even at lower doses [24]. Dose exposure was inferred from dispensing records, which may not reflect actual patient use. Additionally, missingness in key clinical variables—such as lab values and follow-up weights—may have introduced selection bias, as patients included in the analyses may differ systematically from those without complete data. Although the study used complete case analysis and prespecified outcome windows, the possibility of differential data availability across treatment groups remains a concern. Moreover, in contrast to SURMOUNT-5, the study did not capture waist circumference change and therefore cannot provide insights on the impact of these treatments to clinical measures of excess visceral adiposity, which translate to estimated cardiometabolic risk [7].

In summary, this real-world comparative effectiveness study found that adults without diabetes initiating tirzepatide for obesity management achieved greater weight reduction and improvements in select cardiometabolic risk parameters at 6 months than those initiating semaglutide. Notably, this difference in effectiveness was observed even though fewer tirzepatide-treated patients reached higher available doses compared to semaglutide-treated patients. These findings support and extend the evidence from clinical trials, demonstrating that the superior weight reduction efficacy of tirzepatide is evident in routine clinical practice. As the use of obesity medications expands, the real-world data presented here can inform clinical decision making, payer decisions, and future research aimed at optimizing long-term outcomes in clinical practice.

## Supplementary Information

Below is the link to the electronic supplementary material.

Supplementary Material 1

Supplementary Material 2

## Declarations

### Data sharing

The data that support the findings of this study were licensed from Truveta. Per the Data Use Agreement between Lilly and Truveta, the deposition of data into publicly available repositories is not allowed.

## References

[1] Stierman B, Afful J, CarrollMD et al (2025) National Health and Nutrition Examination Survey 2017-March 2020 Prepandemic Data Files-Development of Files and Prevalence Estimates for Selected Health Outcomes. Natl Health Stat Report 14;(158). 10.15620/cdc:106273 10.15620/cdc:106273 PMC11513744 39380201

[2] Masood B, Moorthy M (2023) Causes of obesity: a review. Clin Med 23(4):284–291 10.7861/clinmed.2023-0168 PMC10541056 37524429

[3] Spieker EA, Pyzocha N (2016) Economic impact of obesity. Prim Care 43(1):83–95 viii–ix 26896202 10.1016/j.pop.2015.08.013

[4] Massie DC, Amaro A, Kaplan M (2022) Patient well-being and the clinical and economic burdens associated with obesity in the united States. Am J Manag Care 28:S279–S287 36525675 10.37765/ajmc.2022.89291

[5] Hampl SE, Hassink SG, Skinner AC et al (2023) Clinical practice guideline for the evaluation and treatment of children and adolescents with obesity. Pediatrics 151(2):e2022060640 36622115 10.1542/peds.2022-060640

[6] Singhal V, Sella AC, Malhotra S (2021) Pharmacotherapy in pediatric obesity: current evidence and landscape. Curr Opin Endocrinol Diabetes Obes 28(1):55 33186194 10.1097/MED.0000000000000587 PMC8082722

[7] Aronne LJ, Horn DB, Le Roux CW et al (2025) Tirzepatide as compared with semaglutide for the treatment of obesity. N Engl J Med 393(1):26–36 10.1056/NEJMoa2416394 10.1056/NEJMoa2416394 40353578

[8] Rodriguez PJ, Goodwin Cartwright BM, Gratzl S et al (2024) Semaglutide vs tirzepatide for weight loss in adults with overweight or obesity. JAMA Intern Med. 10.1001/jamainternmed.2024.2525 38976257 10.1001/jamainternmed.2024.2525 PMC11231910

[9] Trinh H, Donovan A, McAdam-Marx C (2025) Real-world effectiveness of Tirzepatide versus semaglutide for weight loss in overweight or obese patients in an ambulatory care setting. Diabetes, obesity and metabolism [Internet]. 27(6):3523–3525 10.1111/dom.16343 10.1111/dom.16343 PMC12046463 40116184

[10] Jamal M, Alhashemi M, Dsouza C et al (2024) Semaglutide and tirzepatide for the management of weight recurrence after sleeve gastrectomy: a retrospective cohort study. Obes Surg 34(4):1324–1332 38430320 10.1007/s11695-024-07137-0

[11] St-Pierre J, Klein J, Choi NK et al (2024) Efficacy and safety of GLP-1 agonists on metabolic parameters in non-diabetic patients with inflammatory bowel disease. Dig Dis Sci 69(12):4437–4445 39516435 10.1007/s10620-024-08720-2

[12] Anson M, Henney AE, Broadwell N et al (2024) Incidence of new onset type 2 diabetes in adults living with obesity treated with Tirzepatide or semaglutide: real world evidence from an international retrospective cohort study. eClin Med 75:102777 10.1016/j.eclinm.2024.102777 PMC11377141 39246719

[13] Gasoyan H, Butsch WS, Schulte R et al (2025) Changes in weight and glycemic control following obesity treatment with semaglutide or Tirzepatide by discontinuation status. Obes (Silver Spring) 33(9):1657–1667. 10.1002/oby.24331 10.1002/oby.24331 PMC12381620 40491239

[14] Okuma H (2025) Effects of Tirzepatide on patients with type 2 diabetes and metabolic dysfunction-associated steatotic liver disease: a retrospective cohort study. Cureus 17(5):e83712 40486301 10.7759/cureus.83712 PMC12145500

[15] Gebre H, Snell JK, Shah VN (2024) 736-P: Comparison of Semaglutide vs. Tirzepatide Therapy in Adults with Type 1 Diabetes (T1D). Diabetes 73(Supplement_1):736-P

[16] Ridgeway G Generalized Boosted Models: A guide to the gbm package

[17] Zagar A, Kadziola Z, Lipkovich I et al (2022) Evaluating bias control strategies in observational studies using frequentist model averaging. J Biopharm Stat 32(2):247–276 35213288 10.1080/10543406.2021.1998095

[18] Lipkovich I, Ratitch B, Qu Y et al (2022) Using principal stratification in analysis of clinical trials. Stat Med 41(19):3837–3877 35851717 10.1002/sim.9439

[19] VanderWeele TJ, Ding P (2017) Sensitivity analysis in observational research: introducing the E-value. Ann Intern Med 167(4):268–274 28693043 10.7326/M16-2607

[20] Novo Nordisk Inc Wegovy (semaglutide) injection, for subcutaneous use [package insert] [Internet]. Plainsboro,NJ; 2021 [cited 2025 July 9]. Available from: https://www.accessdata.fda.gov/drugsatfda_docs/label/2023/215256s007lbl.pdf

[21] Eli Lilly and Company. Zepbound (tirzepatide) injection, for subcutaneous use [package insert] [Internet], Indianapolis IN (2024) [cited 2025 July 9]. Available from: https://www.accessdata.fda.gov/drugsatfda_docs/label/2024/217806s013lbl.pdf

[22] Jastreboff Ania M, Aronne Louis J, Ahmad Nadia N et al (2022) Tirzepatide once weekly for the treatment of obesity. N Engl J Med 387(3):205–216 35658024 10.1056/NEJMoa2206038

[23] Wilding JPH, Batterham RL, Calanna S et al (2021) Once-weekly semaglutide in adults with overweight or obesity. N Engl J Med 384(11):989–1002 33567185 10.1056/NEJMoa2032183

[24] Horn DB, Kahan S, Batterham RL et al Time to weight plateau with tirzepatide treatment in the SURMOUNT-1 and SURMOUNT-4 clinical trials. Clinical Obesity. n/a(n/a):e12734 10.1111/cob.12734 PMC12096058 39800653

[25] McCaffrey DF, Griffin BA, Almirall D et al (2013) A tutorial on propensity score estimation for multiple treatments using generalized boosted models. Stat Med 32(19):3388–3414 23508673 10.1002/sim.5753 PMC3710547

