# Supplementary material for: Comparative effectiveness of tirzepatide and semaglutide for obesity management in US clinical practice: a 6-month retrospective cohort study
Source: J Endocrinol Invest. 2026 Feb 9;49(2):413–23. doi: 10.1007/s40618-025-02792-1 (PMC12924827; doi:10.1007/s40618-025-02792-1)
Supplement: Supplementary file 1 — Supplementary Material 1 [file 40618_2025_2792_MOESM1_ESM.docx]

**Online Resource - Comparative effectiveness of tirzepatide and semaglutide for obesity management in US clinical practice: a 6-month retrospective cohort study**

Carel W. le Roux, PhD,^1^ Nicolae Done, PhD,^2^ Alan J. M. Brnabic, MS,^3^ Abigail Zion, BSc,^2^ Ilya Lipkovich, PhD,^4^ Zbigniew Kadziola, MSc,^4^ Julia P. Dunn, MD,^4^ Urvi Desai, PhD,^2^ Noam Kirson, PhD,^2^ Georgios K. Dimitriadis, PhD,^4^ Hong Kan, PhD^4^

^1^University College Dublin, Conway Institute Belfield Dublin 4, Ireland and Ulster University, Cromore Road, Coleraine, Co. Londonderry, BT52 1SA, UK.

^2^Analysis Group, Inc. 111 Huntington Av, 14th Floor, Boston, MA 02199-7668, USA.

^3^Eli Lilly and Company, Level 9, 60 Margaret St, Sydney, NSW 2000, Australia.

^4^Eli Lilly and Company, 893 S Delaware St, Indianapolis, IN 46285, USA.

**Corresponding author: Alan J. M. Brnabic, MS** ([alan_brnabic@lilly.com](mailto:alan_brnabic@lilly.com))

*Journal of Endocrinological Investigation*

Table of Contents

[Supplemental methodological details 3](#_Toc204066924)

[Methodological details for sample selection and cohort definitions 3](#_Toc204066925)

[Methodological details for data processing, detection and exclusion of implausible values 4](#_Toc204066926)

[List of R packages used for key analyses 6](#_Toc204066927)

[Methodological details for derivation of propensity score weights 7](#_Toc204066928)

[Descriptive methodological details for FMA analyses 9](#_Toc204066929)

[Methodological details for principal stratification analysis 10](#_Toc204066930)

[Methodological details for E-value analyses 11](#_Toc204066931)

[Supplemental Tables 12](#_Toc204066932)

[Supplemental Table 1. Diagnosis, procedure, and medication codes used for sample selection 12](#_Toc204066933)

[Supplemental Table 2. Diagnosis and procedure codes used to identify obesity-related complications 16](#_Toc204066934)

[Supplemental Table 3. Diagnosis, procedure, and drug codes used to identify diabetes 26](#_Toc204066935)

[Supplemental Table 4. Diagnosis, procedure, and drug codes used for identifying unintentional weight loss 38](#_Toc204066936)

[Supplemental Table 5. Drug codes used to identify obesity-related medications 171](#_Toc204066937)

[Supplemental Table 6. Diagnosis codes used to identify other comorbidities 206](#_Toc204066938)

[Supplemental Table 7. Codes used to identify laboratory and vital assessments 228](#_Toc204066939)

[Supplemental Table 8. Definitions and derivation for clinical outcomes analyzed in the study 230](#_Toc204066940)

[Supplemental Table 9. Variance ratio of demographic and clinical variables (continuous variables) in the on-treatment cohort, tirzepatide group versus semaglutide group 231](#_Toc204066941)

[Supplemental Table 10. Demographic and clinical characteristics by treatment group in the mITT cohort, before and after adjustment 232](#_Toc204066942)

[Supplemental Table 11. Variance ratio of demographic and clinical variables (continuous variables) in the mITT cohort, tirzepatide group vs semaglutide group 233](#_Toc204066943)

[Supplemental Table 12. Dosage patterns by treatment group in the on-treatment cohort 234](#_Toc204066944)

[Supplemental Table 13. Unadjusted and adjusted percentages of patients with weight reduction at 6 months in the on-treatment cohort (primary analysis method) 237](#_Toc204066945)

[Supplemental Table 14. Adjusted percentage change in cardiometabolic outcomes in the on-treatment cohort (primary analysis method) 238](#_Toc204066946)

[Supplemental Table 15. Adjusted odds ratios of downward BMI categorical shift in the on-treatment cohort (primary analysis method) 239](#_Toc204066947)

[Supplemental Table 16. FMA adjusted odds ratios and 95% CIs for weight reduction at 6 months 240](#_Toc204066948)

[Supplemental Table 17. Unadjusted and adjusted percentages of patients with weight reduction at 6 months in the mITT cohort 241](#_Toc204066949)

[Supplemental Table 18. Adjusted odds ratios of patients with weight reduction at 6 months, mITT cohort 242](#_Toc204066950)

[Supplemental Figures 243](#_Toc204066951)

[Supplemental Figure 1. Propensity score distribution before and after weighting in the on-treatment cohort 243](#_Toc204066952)

[Supplemental Figure 2. Propensity weight distribution in the on-treatment cohort 244](#_Toc204066953)

[Supplemental Figure 3. Love plot of standardized mean covariate differences between the treatment groups in the on-treatment cohort 245](#_Toc204066954)

[Supplemental Figure 4. Love plot of variance ratios of continuous covariates between the treatment groups in the on-treatment cohort 246](#_Toc204066955)

[Supplemental Figure 5. Forest plot of FMA model strategies for percentage weight change in the on-treatment cohort - Showing FMA, best model, and all 11 analysis strategies 247](#_Toc204066956)

[Supplemental Figure 6. Propensity score distribution before and after weighting in the mITT cohort 248](#_Toc204066957)

[Supplemental Figure 7. Propensity weight distribution after weighting in the mITT cohort 249](#_Toc204066958)

[Supplemental Figure 8. Love plot of standardized mean covariate differences between the treatment groups in the mITT cohort 250](#_Toc204066959)

[Supplemental Figure 9. Love plot of variance ratios for continuous covariates between the treatment groups in the mITT cohort 251](#_Toc204066960)

[Supplemental Figure 10. E-value analysis for unmeasured confounding (on-treatment cohort, primary analysis method) 252](#_Toc204066961)

# Supplemental methodological details

### Methodological details for sample selection and cohort definitions

The study population was drawn from the Truveta EHR database and included adults aged ≥18 years who initiated either tirzepatide (Zepbound) or semaglutide (Wegovy) for obesity management between December 1, 2023, and June 30, 2024. Patients were required to have a baseline BMI ≥30 kg/m², or BMI between 27 and 29·9 kg/m² (see Supplemental Table 1) with at least one obesity-related complication, consistent with FDA labelling. A comprehensive list of qualifying complications and codes used to identify them is provided in Supplemental Table 2.

The index date was defined as the first observed dispensing of Zepbound or Wegovy within the study window. Patients were required to have:

- At least one recorded healthcare encounter during the 6-month follow-up period
- At least one valid weight or BMI measurement within 60 days prior to the index date and within 30 days before or after the 6-month follow-up endpoint

To qualify for the on-treatment cohort, patients had to have adherence ≥80% (based on proportion of days covered, PDC) and could not switch to a non-index GLP-1 or dual GIP/GLP-1 agonist during follow-up. Adherence was calculated using days’ supply from dispensing records as the number of days with medication availability divided by the number of days in the 6-month follow-up window, allowing stockpiling across overlapping fills. Discontinuation was defined as a gap of >45 days without a new dispensing or administration. Switching was defined as initiation of a non-index GLP-1 receptor agonist (RA) or dual GIP-GLP-1 RA during the follow-up period.

Patients were excluded if they had:

- Any evidence of type 1 or type 2 diabetes, including (Supplemental Table 3):
  1. ≥1 diagnosis or procedure code indicating type 1, type 2, or other specified diabetes;
  2. ≥1 HbA1c ≥6.5%;
  3. ≥1 prescription/dispensing/administration of a non–GLP-1 RA glucose-lowering medication (insulin, metformin, sulfonylurea, sodium-glucose transport protein 2 (SGLT2) inhibitor, dipeptidyl peptidase 4 (DPP-4 inhibitor); or
  4. ≥1 prescription/dispensing/administration of a GIP/GLP-1 RA used for glycaemic control (dulaglutide, exenatide, liraglutide [including Xultophy], lixisenatide [including Soliqua], semaglutide [Ozempic/Rybelsus], tirzepatide [Mounjaro]).
- Evidence of prior GLP-1 or dual GIP/GLP-1 RA obesity management medication (OMM) use during the 12-month baseline (Supplemental Table 5)
- History of metabolic bariatric procedures, or
- Conditions associated with unintentional weight change (e.g., malignancy, eating disorders, hyperthyroidism – see complete list and codes used to identify conditions in Supplemental Table 4)

The modified intention-to-treat (mITT) cohort was defined to include patients regardless of adherence or switching status but otherwise meeting all other eligibility criteria and with non-missing weight and/or BMI values at both baseline and follow-up.

### Methodological details for data processing, detection and exclusion of implausible values

To ensure analytic integrity, biologically implausible or incorrectly formatted values were excluded from analysis according to rules prespecified in the study protocol and specification document. These exclusions applied to baseline and follow-up values used in all outcome derivations (e.g., weight change, BMI, cardiometabolic parameters).

**Weight**

- Weight values with negative values or nonsensical units (e.g., seconds) were discarded.
- When the unit was missing, thresholds were applied to infer the most likely unit:
  - 40,824–317,520 → grams
  - 1,440–11,200 → ounces
  - >317 → pounds
  - <125 → kilograms
- If a unit could not be assigned after applying consistency checks using the patient’s average weight over a 30-month window (15 months before and after index), the record was discarded.
- Final exclusion threshold: weight values <90 kg or >700 kg were discarded

**BMI**

- BMI records were excluded if the value was <14 or >100 kg/m².
- If multiple BMI values were recorded on the same date, the lowest valid value was used.

**Height**

- Implausible height values were excluded if <1 meter or >3 meters.
- Height records with incorrect units (e.g., seconds) were removed.
- When the unit was missing, heuristic rules were applied:
  - <3 → meters
  - 3–10 → feet
  - 10–100 → inches
  - 100 → centimeters
- If height could not be resolved or validated, derived BMI was not calculated for that record.

**HbA1c**

- HbA1c values >25% were excluded.
- When units were missing:
  - If value >25, assumed mg/dL and converted using the formula: $HbA1c\%= \frac{1000\times\frac{grams}{deciliter}+46.7}{28.7}.$
  - If ≤25, assumed percent.
- When units were g/dL, values were calculated using the formula:

$$HbA1c\%= \frac{1000\times\frac{grams}{deciliter}+46.7}{28.7}.$$

- Records with non-interpretable units or mismatched value-unit pairs were excluded.

**Cardiometabolic labs and vitals**

Although specific implausibility thresholds for all lab and vital signs (e.g., cholesterol, BP) were not explicitly listed, values with:

- missing units, or
- units incompatible with the expected range for the parameter, or
- values that could not be verified using standard derivation logic
  were excluded from the final analytic sample.

All exclusions were applied before imputation or outcome calculation and applied uniformly across treatment groups and time points.

**Days’ supply**

For administrations of tirzepatide and semaglutide, the days of supply was assumed to be 7 (equivalent to a one-time injection).

For dispenses where days of supply was missing, less than a plausible minimum of 5 days, or more than a plausible maximum of 365 for tirzepatide and semaglutide, days of supply was derived from the dispense quantity.

- When dispense quantity was equal to 2 mL, it was assumed that 4 injectors of 0·5 mL were dispensed for a day’s supply for 28 days.
- When dispense quantity was equal to 3 mL, it was assumed that 4 injectors of 0·75 mL were dispensed for a day’s supply for 28 days.
- When both days of supply and dispense quantity were missing, days of supply was assumed to be 28 days.

Medications and administrations were de-duplicated per day. The maximum days of supply reported for each day was used.

**Dosage**

Dosage for semaglutide was sometimes reported as the ratio of milligrams to milliliters (e.g., 1 mg per every 1 mL). The following dosages are available for Wegovy:

- 0·25 mg/0·5 mL
- 0·5 mg/0·5 mL
- 1 mg/0·5 mL
- 1·7 mg/0·75 mL
- 2·4 mg/0·75 mL

As such, dose per pen was assumed as follows:

| Dosage in data | Dose per pen assumed |
| --- | --- |
| 0·5 mg/mL | 0·25 mg/0·5 mL |
| 1 mg/mL | 0·5 mg/0·5 mL |
| 2 mg/mL | 1 mg/ 0·5 mL |
| 2·27 mg/mL | 1·7 mg/0·75 mL |
| 3·2 mg/mL | 2·4 mg/0·75 mL |

If records with known and unknown dose occurred on the same date, the known dose was selected. If multiple records with differing known doses occurred on the index date, the minimum dose was selected. If the multiple records with differing known doses occurred on another date, the maximum dose was selected.

### List of R versions and packages used for key analyses

R version 4.2.3 was used for the main analyses.

R version 4.3.2 was used for the sensitivity analyses.

The following R package versions were used:

- **WeightIt v. 1.3.2:** Used to estimate propensity score ATT weights via generalized boosted models (GBMs) as part of the causal inference framework.
- **cobalt v. 4.5.1:** Used to evaluate covariate balance before and after weighting. Generated diagnostics such as standardized mean differences and balance plots.
- **marginaleffects v. 0.25.0:** Used to compute average marginal effects and predicted treatment effects from weighted regression models.
- **tidymodels v. 1.3.0:** Used for bootstrapping and managing model workflows in a structured and reproducible manner.
- **Furrr v. 0.3.1:** Enabled parallel processing during computationally intensive procedures.
- **gbm v. 2.2.2:** Used for implementing generalized boosted models to estimate treatment assignment probabilities in the primary and FMA-based causal inference approaches.
- **fastDummies v. 1.7.5:** Used to create binary indicators (dummy variables) for categorical variables prior to modeling.

### Methodological details for derivation of propensity score weights

This study used propensity score (PS) weighting to estimate the average treatment effect on the treated (ATT), aligning with the study’s primary on-treatment estimand. PS weights were applied to control for confounding due to differences in baseline characteristics between patients initiating tirzepatide (Zepbound) and semaglutide (Wegovy) for weight management.

#### Propensity Score Estimation

Propensity scores were estimated using generalized boosted models (GBM), a machine-learning algorithm that constructs an ensemble of decision trees to flexibly model treatment assignment. The model used a Bernoulli distribution, with treatment group (Zepbound vs. Wegovy) as the dependent variable. The GBM was tuned to minimize the average absolute standardized mean difference (SMD) across all covariates, with hyperparameters set as follows: interaction depth = 3, shrinkage = 0·01, number of trees = 10,000, bag fraction = 1, and no trimming or offset. Alternate tuning configurations (e.g., tree depth = 2 or 4) were tested and yielded no substantial improvement in balance.

The model included a prespecified list of baseline covariates derived from the protocol and specification, encompassing:

- **Demographics**
  - Age at index (continuous)
  - Gender
  - Race
  - Ethnicity
  - U.S. census region
  - U.S. state
- **Socioeconomic status**
  - Highest education level
  - Individual income range
  - Household income range
- **Anthropometrics**
  - Baseline weight
  - Baseline BMI
  - BMI category indicators:
  - Overweight
  - Obesity Class 1
  - Obesity Class 2
  - Obesity Class 3
- **Clinical comorbidities**
  - Hypertension
  - Dyslipidaemia
  - Obstructive sleep apnea
  - Metabolic syndrome
  - Insulin resistance
  - Prediabetes
  - Abnormal glycemia
  - Hypercholesterolemia
  - Gastroesophageal reflux disease (GERD)
  - Osteoarthritis (knee and/or hip)
  - Chronic kidney disease
  - Atherosclerotic cardiovascular disease
  - Coronary artery disease
  - Cerebrovascular disease
  - Peripheral artery disease
  - Chronic heart failure (HFpEF)
  - Chronic obstructive pulmonary disease (COPD)
  - Asthma
  - Polycystic ovary syndrome (PCOS)
  - Anxiety and depression
  - Back pain
  - Rheumatoid arthritis
  - Psoriasis and psoriatic arthritis
  - Hidradenitis suppurativa
  - Atopic dermatitis
  - Irritable bowel syndrome
  - Atrial fibrillation
  - Urinary incontinence
  - Glaucoma
  - Male hypogonadism
  - Female infertility
  - Hyperuricemia
  - Obesity hypoventilation syndrome
  - Ankylosing spondylitis
  - Non-radiographic axial spondyloarthritis
  - Venous thromboembolism
  - Metabolic dysfunction-associated steatotic liver disease/steatohepatitis (MASLD/MASH)
- **Healthcare utilization**
  - Number of healthcare system interactions in the 6 months prior to index
  - Number of healthcare system interactions in the 12 months prior to index
- **Medication history**
  - Prior use of non-incretin anti-obesity medications:
  - Orlistat
  - Phentermine
  - Phentermine/topiramate
  - Bupropion/naltrexone
  - Diethylpropion
- **Summary counts**
  - Number of obesity-related complications
  - Number of other comorbidities

#### ATT Weighting and Balance Assessment

Weights were constructed to estimate the ATT: Zepbound-treated patients received a weight of 1, and Wegovy-treated patients received a weight of PS/(1−PS). Balance was evaluated by comparing absolute standardized mean differences (SMDs) and variance ratios across treatment groups before and after weighting. An absolute SMD <0·1 and a variance ratio between 0·5 and 2·0 were considered acceptable. The overall balance of the PS model was assessed using average absolute SMD across all covariates, and variance ratios for continuous covariates only.

#### Use of Weights in Outcome Models

All primary and secondary comparative analyses were conducted using the weighted samples, applying generalized linear models (GLMs) with robust standard errors. For continuous outcomes (e.g., percent weight change), GLMs with an identity link and Gaussian distribution were used. For binary outcomes (e.g., achieving ≥10% weight loss), logistic regression was used. The following covariates were included in the outcome models to adjust for residual confounding, per the prespecified analysis plan: baseline age [continuous], gender [categorical], race [categorical], ethnicity [categorical], baseline number of ORCs [continuous], any baseline use of non-incretin AOMs [binary], and baseline body weight [continuous].

Weights were also reused in secondary and exploratory descriptive analyses unless otherwise noted. In select cardiometabolic outcome analyses requiring complete cases, new weights were re-estimated in cohorts restricted to patients with non-missing outcome values at both baseline and follow-up.

### Descriptive methodological details for FMA analyses

To evaluate the robustness of treatment effect estimates and account for model uncertainty, we implemented a **Frequentist Model Averaging (FMA)** approach as a sensitivity analysis. This data-driven framework integrates multiple causal inference strategies and combines their estimates to produce a weighted average treatment effect. The approach follows the methods described by Zagar et al. (2022)^1^ and adapts them to a real-world comparative effectiveness setting.

#### Model Structure

The FMA included 11 prespecified model strategies, each combining a treatment model (TM) and an outcome model (OM).

Treatment models estimated propensity scores using either LASSO-penalized logistic regression or gradient boosted trees (GBTM).

Outcome models included:

- GLMs
- LASSO-penalized regression
- Unadjusted comparisons

Adjustment strategies spanned matching (1:1 greedy matching with caliper), stratification, and inverse probability weighting (IPTW). The covariates used in the TM were the full set of baseline characteristics specified above for the primary analysis method. For the OM, the following covariates were used:

- **Weight change models**: baseline age [continuous], gender [categorical], race [categorical], ethnicity [categorical], baseline number of ORCs [continuous], any baseline use of non-incretin AOMs [binary], and baseline body weight [continuous]
- **BMI change models**: baseline age [continuous], gender [categorical], race [categorical], ethnicity [categorical], baseline number of ORCs [continuous], any baseline use of non-incretin AOMs [binary], and baseline BMI [continuous]
- **Cardiometabolic parameter models**: baseline value of corresponding parameter, baseline age [continuous], gender [categorical], race [categorical], ethnicity [categorical], and baseline BMI [continuous]

#### Cross-validation and Weighting

Each TM/OM pair was fit using 5-fold cross-validation. For each fold, the model was trained on 80% of the data and tested on the remaining 20% to calculate the mean squared prediction error (MSPE), which served as a measure of model accuracy. The average MSPE across folds was used to assign a weight to each model, with lower MSPEs receiving greater weight.

The final treatment effect estimate was the weighted average of all individual treatment effects, where weights were proportional to inverse MSPE.

#### Bootstrapping and Confidence Intervals

To estimate 95% confidence intervals, the entire FMA process was repeated using 100 bootstrap samples drawn with replacement from the original dataset. The confidence bounds were calculated as the 2·5^th^ and 97·5^th^ percentiles of the bootstrapped treatment effect estimates.

#### Reporting and Interpretation

The FMA produced treatment effect estimates for both continuous outcomes (e.g., percent weight change, BMI change) and binary outcomes (e.g., ≥10% weight loss). Results are reported as least squares mean differences (LSMDs) or odds ratios (ORs) with 95% confidence intervals. Significance was interpreted based on whether the 95% CI excluded the null (0 for LSMD, 1 for OR).

The model with the highest weight (i.e., lowest MSPE) was identified and reported as the “best model.” In the primary FMA analysis of percent weight change, the best model used gradient boosted TM and weighted LASSO OM.

# References

1. Zagar A, Kadziola Z, Lipkovich I, Madigan D, Faries D. Evaluating bias control strategies in observational studies using frequentist model averaging. *Journal of Biopharmaceutical Statistics*. 2022;32(2):247-276. doi:10.1080/10543406.2021.1998095

### Methodological details for principal stratification analysis

To estimate the treatment effect of tirzepatide versus semaglutide in a hypothetical population of patients who would be adherent to tirzepatide, we applied a principal stratification framework. This approach isolates the (ATT) within the principal stratum of patients who would remain adherent to tirzepatide over the 6-month follow-up period, regardless of assigned treatment.

#### Step 1: Propensity ***Score*** Estimation

We first estimated propensity scores (PS) to model the probability of treatment assignment. These were calculated using a gradient boosted model, incorporating all baseline covariates used in the primary ATT analysis. The PS represents the probability of receiving tirzepatide (vs. semaglutide) given observed covariates:

PS(X) = P(T = Z | X)
where T denotes treatment assignment (Zepbound or Wegovy) and X represents baseline covariates.

#### Step 2: Principal Score Estimation

We then estimated **principal scores (PA)**, defined as the predicted probability of adherence to tirzepatide if assigned:

PA(X) = P(A = 1 | X, T = Z)
Here, A = 1 indicates adherence (≥80% proportion of days covered over 6 months), and the model was fit only among tirzepatide-treated patients using the same baseline covariates X. This yields an estimated probability of adherence conditional on treatment and covariates.

#### Step 3: Weight Construction and Estimation

Weights were constructed as follows:

- **Tirzepatide patients** who were adherent received a weight of **1** (the target population).
- **Semaglutide patients** received a weight proportional to their likelihood of being adherent if assigned to tirzepatide, scaled by their likelihood of treatment assignment:

Weight = PS(X) × PA(X) / [1 − PS(X) × PA(X)]

These weights were applied in a generalized linear model to estimate the treatment effect on percent weight change in this principal stratum. This approach allows for comparison of outcomes under semaglutide among patients similar to those who would have adhered to tirzepatide.

**Interpretation**

This analysis provides an estimate of the treatment effect among patients who would have been adherent to tirzepatide, had they received it. Results are presented as adjusted least squares mean differences in percent weight change with 95% confidence intervals based on robust (i.e., “sandwich”) error estimation.

### Methodological details for E-value analyses

To assess the robustness of the observed treatment effect to potential unmeasured confounding, the E-value was calculated. The E-value is a metric that quantifies the minimum strength of association that an unmeasured confounder would need to have with both the treatment and the outcome, conditional on the measured covariates, to fully explain away the observed association.

The E-value was calculated for the primary continuous outcome: percent change in body weight from baseline to 6 months. Although originally developed for binary outcomes on the risk ratio (RR) scale, the E-value method can be extended to continuous outcomes by first transforming the treatment effect estimate into a risk ratio equivalent. This transformation assumes a monotonic and meaningful relationship between the continuous outcome and a clinically interpretable event (e.g., weight loss).

**Calculation Steps**

1. **Estimate transformation**: The PAM-adjusted mean difference in percent weight change between Zepbound and Wegovy was converted to an approximate RR equivalent, using standard methods for interpreting continuous treatment effects in the context of binary risk.
2. **E-value derivation**:
   - The E-value for the point estimate was calculated using the transformed effect size.
   - A second E-value was calculated for the lower bound of the 95% confidence interval, representing the minimum strength of confounding required to reduce the lower bound to the null.
3. **Comparison to observed confounders**: To contextualize the magnitude of the E-value, it was compared to the observed strength of association between key measured covariates (e.g., baseline weight, age, sex, race, number of obesity-related complications, and prior medication use) and both treatment assignment and the outcome.

# Supplemental Tables

### Supplemental Table 1. Diagnosis, procedure, and medication codes used for sample selection

| **Code System** | **Code** | **Concept Name** |
| --- | --- | --- |
| *Zepbound* |  |  |
| NDC | 00020152 | tirzepatide 2·5mg/·5mL SUBCUTANEOUS INJECTION, SOLUTION |
| NDC | 0002015201 | 0·5 ML tirzepatide 5 MG/ML Vial, Single-dose, 1 Vial, Single-dose in 1 Carton [Zepbound] |
| NDC | 00020243 | tirzepatide 5mg/·5mL SUBCUTANEOUS INJECTION, SOLUTION |
| NDC | 0002024301 | 0·5 ML tirzepatide 10 MG/ML Vial, Single-dose, 1 Vial, Single-dose in 1 Carton [Zepbound] |
| NDC | 00021214 | tirzepatide 7·5mg/·5mL SUBCUTANEOUS INJECTION, SOLUTION |
| NDC | 0002121401 | 0·5 ML tirzepatide 15 MG/ML Vial, Single-dose, 1 Vial, Single-dose in 1 Carton [Zepbound] |
| NDC | 00021340 | tirzepatide 10mg/·5mL SUBCUTANEOUS INJECTION, SOLUTION |
| NDC | 0002134001 | 0·5 ML tirzepatide 20 MG/ML Vial, Single-dose, 1 Vial, Single-dose in 1 Carton [Zepbound] |
| NDC | 00021423 | tirzepatide 12·5mg/·5mL SUBCUTANEOUS INJECTION, SOLUTION |
| NDC | 0002142301 | 0·5 ML tirzepatide 25 MG/ML Vial, Single-dose, 1 Vial, Single-dose in 1 Carton [Zepbound] |
| NDC | 00022002 | tirzepatide 15mg/·5mL SUBCUTANEOUS INJECTION, SOLUTION |
| NDC | 0002200201 | 0·5 ML tirzepatide 30 MG/ML Vial, Single-dose, 1 Vial, Single-dose in 1 Carton [Zepbound] |
| NDC | 00022457 | tirzepatide 15mg/·5mL SUBCUTANEOUS INJECTION, SOLUTION |
| NDC | 0002245701 | 0·5 ML tirzepatide 30 MG/ML Syringe |
| NDC | 0002245780 | 0·5 ML tirzepatide 30 MG/ML Syringe, 4 Syringes in 1 Carton [Zepbound] |
| NDC | 00022460 | tirzepatide 12·5mg/·5mL SUBCUTANEOUS INJECTION, SOLUTION |
| NDC | 0002246001 | 0·5 ML tirzepatide 25 MG/ML Syringe |
| NDC | 0002246080 | 0·5 ML tirzepatide 25 MG/ML Syringe, 4 Syringes in 1 Carton [Zepbound] |
| NDC | 00022471 | tirzepatide 10mg/·5mL SUBCUTANEOUS INJECTION, SOLUTION |
| NDC | 0002247101 | 0·5 ML tirzepatide 20 MG/ML Syringe |
| NDC | 0002247180 | 0·5 ML tirzepatide 20 MG/ML Syringe, 4 Syringes in 1 Carton [Zepbound] |
| NDC | 00022484 | tirzepatide 7·5mg/·5mL SUBCUTANEOUS INJECTION, SOLUTION |
| NDC | 0002248401 | 0·5 ML tirzepatide 15 MG/ML Syringe |
| NDC | 0002248480 | 0·5 ML tirzepatide 15 MG/ML Syringe, 4 Syringes in 1 Carton [Zepbound] |
| NDC | 00022495 | tirzepatide 5mg/·5mL SUBCUTANEOUS INJECTION, SOLUTION |
| NDC | 0002249501 | 0·5 ML tirzepatide 10 MG/ML Syringe |
| NDC | 0002249580 | 0·5 ML tirzepatide 10 MG/ML Syringe, 4 Syringes in 1 Carton [Zepbound] |
| NDC | 00022506 | tirzepatide 2·5mg/·5mL SUBCUTANEOUS INJECTION, SOLUTION |
| NDC | 0002250601 | 0·5 ML tirzepatide 5 MG/ML Syringe |
| NDC | 0002250661 | 0·5 ML tirzepatide 5 MG/ML Syringe, 4 Syringes in 1 Carton [Zepbound] |
| NDC | 0002250680 | 0·5 ML tirzepatide 5 MG/ML Syringe, 4 Syringes in 1 Carton [Zepbound] |
| NDC | 00002015204 | tirzepatide 2·5 mg/·5mL SUBCUTANEOUS INJECTION, SOLUTION Zepbound |
| NDC | 00002024304 | tirzepatide 5 mg/·5mL SUBCUTANEOUS INJECTION, SOLUTION Zepbound |
| RxNorm | 2669702 | Zepbound |
| RxNorm | 2669703 | tirzepatide 5MG/ML [Zepbound] |
| RxNorm | 2669704 | Zepbound Injectable Product |
| RxNorm | 2669705 | tirzepatide Auto-Injector [Zepbound] |
| RxNorm | 2669706 | zepbound 2·5 MG in 0·5 ML Auto-Injector |
| RxNorm | 2669708 | tirzepatide 10MG/ML [Zepbound] |
| RxNorm | 2669709 | zepbound 5 MG in 0·5 ML Auto-Injector |
| RxNorm | 2669711 | tirzepatide 15MG/ML [Zepbound] |
| RxNorm | 2669712 | zepbound 7·5 MG in 0·5 ML Auto-Injector |
| RxNorm | 2669714 | tirzepatide 20MG/ML [Zepbound] |
| RxNorm | 2669715 | zepbound 10 MG in 0·5 ML Auto-Injector |
| RxNorm | 2669717 | tirzepatide 25MG/ML [Zepbound] |
| RxNorm | 2669718 | zepbound 12·5 MG in 0·5 ML Auto-Injector |
| RxNorm | 2669720 | tirzepatide 30MG/ML [Zepbound] |
| RxNorm | 2669721 | zepbound 15 MG in 0·5 ML Auto-Injector |
| RxNorm | 2669707 | tirzepatide 5 MG/ML Auto-Injector [Zepbound] |
| RxNorm | 2669710 | tirzepatide 10 MG/ML Auto-Injector [Zepbound] |
| RxNorm | 2669713 | tirzepatide 15 MG/ML Auto-Injector [Zepbound] |
| RxNorm | 2669716 | tirzepatide 20 MG/ML Auto-Injector [Zepbound] |
| RxNorm | 2669719 | tirzepatide 25 MG/ML Auto-Injector [Zepbound] |
| RxNorm | 2669722 | tirzepatide 30 MG/ML Auto-Injector [Zepbound] |
|  |  |  |
| *Obesity* |  |  |
| ICD-10-CM | E66.01 | Morbid (severe) obesity due to excess calories |
| ICD-10-CM | E66.09 | Other obesity due to excess calories |
| ICD-10-CM | E66.1 | Drug-induced obesity |
| ICD-10-CM | E66.2 | Morbid (severe) obesity with alveolar hypoventilation |
| ICD-10-CM | E66.8 | Other obesity |
| ICD-10-CM | E66.9 | Obesity, unspecified |
| ICD-10-CM | Z68.30 | Body mass index (BMI) 30·0-30·9, adult |
| ICD-10-CM | Z68.31 | Body mass index (BMI) 31·0-31·9, adult |
| ICD-10-CM | Z68.32 | Body mass index (BMI) 32·0-32·9, adult |
| ICD-10-CM | Z68.33 | Body mass index (BMI) 33·0-33·9, adult |
| ICD-10-CM | Z68.34 | Body mass index (BMI) 34·0-34·9, adult |
| ICD-10-CM | Z68.35 | Body mass index (BMI) 35·0-35·9, adult |
| ICD-10-CM | Z68.36 | Body mass index (BMI) 36·0-36·9, adult |
| ICD-10-CM | Z68.37 | Body mass index (BMI) 37·0-37·9, adult |
| ICD-10-CM | Z68.38 | Body mass index (BMI) 38·0-38·9, adult |
| ICD-10-CM | Z68.39 | Body mass index (BMI) 39·0-39·9, adult |
| ICD-10-CM | Z68.41 | Body mass index (BMI) 40·0-44·9, adult |
| ICD-10-CM | Z68.42 | Body mass index (BMI) 45·0-49·9, adult |
| ICD-10-CM | Z68.43 | Body mass index (BMI) 50·0-59·9, adult |
| ICD-10-CM | Z68.44 | Body mass index (BMI) 60·0-69·9, adult |
| ICD-10-CM | Z68.45 | Body mass index (BMI) 70 or greater, adult |
| SNOMED CT | 83911000119104 | Severe obesity |
| SNOMED CT | 190965006 | Drug-induced obesity |
| SNOMED CT | 190966007 | Extreme obesity with alveolar hypoventilation |
| SNOMED CT | 162864005 | Body mass index 30+ - obesity |
| SNOMED CT | 238136002 | Morbid obesity |
| SNOMED CT | 408512008 | Body mass index 40+ - severely obese |
| SNOMED CT | 414915002 | Obese |
| SNOMED CT | 414916001 | Obesity |
| SNOMED CT | 415530009 | Simple obesity |
| SNOMED CT | 788996008 | Obesity in adolescence |
| SNOMED CT | 819948005 | Obese class III |
| SNOMED CT | 443371000124107 | Obese class I |
| SNOMED CT | 443381000124105 | Obese class II |
| SNOMED CT | 461341000124106 | Lower body obesity |
| SNOMED CT | 162690006 | O/E - obese |
| SNOMED CT | 238134004 | Generalized obesity |
| SNOMED CT | 248311001 | Central obesity |
| SNOMED CT | 248312008 | Peripheral obesity |
| SNOMED CT | 414917005 | Obesity by adipocyte growth pattern |
| SNOMED CT | 414918000 | Obesity by age of onset |
| SNOMED CT | 414919008 | Obesity by contributing factors |
| SNOMED CT | 414920002 | Obesity by fat distribution pattern |
| SNOMED CT | 44772007 | Maternal obesity syndrome |
| SNOMED CT | 722596001 | Obesity caused by energy imbalance |
| SNOMED CT | 270486005 | Localized adiposity |
| SNOMED CT | 292464007 | Constitutional obesity |
| SNOMED CT | 294493008 | Lifelong obesity |
| SNOMED CT | 296526005 | Adult-onset obesity |
| SNOMED CT | 297500005 | Endogenous obesity |
| ICD-10-CM | O99.21 | Obesity complicating pregnancy, childbirth, and the puerperium |
| ICD-10-CM | Z68.54 | BMI pediatrics >= 95th percentile for age |
|  |  |  |
| *BMI laboratory assessments* | | |
| LOINC | 39156-5 | BMI ratio (kg/m2) |
| LOINC | 97057-4 | BMI score for risk calculation (Results: 1=overweight, 3=obese) |
| LOINC | 88087-2 | Estimated BMI greater than 40 (Results: Y, N) |
|  |  |  |
| *Weight laboratory assessments* | | |
| LOINC | 18690-8 | First Body weight Set |
| LOINC | 18692-4 | First Body weight special circumstances |
| LOINC | 18833-4 | First Body weight |
| LOINC | 29463-7 | Body weight |
| LOINC | 3141-9 | Body weight Measured |
| LOINC | 52416-5 | Body weight Set |
| LOINC | 58229-6 | Body weight Measured --when specimen taken |
| LOINC | 69460-4 | Body weight Mother --pre current pregnancy |
| LOINC | 8337-8 | Body weight special circumstances |
| LOINC | 8338-6 | Body weight Measured --ante partum |
| LOINC | 8341-0 | Dry body weight Measured |
| LOINC | 8344-4 | Body weight Measured --post dialysis |
| LOINC | 8345-1 | Body weight Measured --post partum |
| LOINC | 8346-9 | Body weight Measured --postoperative |
| LOINC | 8347-7 | Body weight Measured --pre dialysis |
| LOINC | 8348-5 | Body weight Measured --pre pregnancy |
| LOINC | 8349-3 | Body weight Measured --preoperative |
| LOINC | 8350-1 | Body weight Measured --with clothes |
| LOINC | 8351-9 | Body weight Measured --without clothes |
| LOINC | 8352-7 | Body weight - Clothing worn during measure |
| LOINC | 103693-8 | Body weight during assessment period |
|  |  |  |
| *Height laboratory assessments* | | |
| LOINC | 3137-7 | Body height measured |
| LOINC | 8302-2 | Body height |
| LOINC | 8305-5 | Body height --post partum |
| LOINC | 8306-3 | Body height --lying |
| LOINC | 8307-1 | Body height --preoperative |
| LOINC | 8308-9 | Body height --standing |
|  |  |  |
| *Overweight* |  |  |
| ICD-10-CM | E66.3 | Overweight |
| ICD-10-CM | Z68.27 | Body mass index (BMI) 27·0-27·9, adult |
| ICD-10-CM | Z68.28 | Body mass index (BMI) 28·0-28·9, adult |
| ICD-10-CM | Z68.29 | Body mass index (BMI) 29·0-29·9, adult |
| SNOMED CT | 238131007 | Overweight |
| SNOMED CT | 162863004 | Body mass index 25-29 - overweight |
| SNOMED CT | 722595002 | Overweight in adulthood with body mass index of 25 or more but less than 30 |
| SNOMED CT | 450451007 | Overweight in childhood |
| SNOMED CT | 268915006 | O/E - weight 10-20% over ideal |
| SNOMED CT | 268916007 | O/E - weight greater than 20% over ideal |
| SNOMED CT | 275947003 | O/E - overweight |

Abbreviations: ICD-10-CM = International Classification of Diseases, 10^th^ edition, Clinical Modification; LOINC = Logical Observation Identifiers Names and Codes; NDC = National Drug Code; O/E = on examination; SNOMED CT = Systematized Nomenclature of Medicine – Clinical Terms.

### Supplemental Table 2. Diagnosis and procedure codes used to identify obesity-related complications

| **Code System** | **Code** | **Concept Name** |
| --- | --- | --- |
| *ASCVD - Coronary artery disease (inclusive of MI) and include revascularization (CABG, PCI, PTCA)* | | |
| ICD-10-CM | I20 | Angina pectoris |
| ICD-10-CM | I21 | Acute myocardial infarction |
| ICD-10-CM | I22 | Subsequent ST elevation (STEMI) and non-ST elevation (NSTEMI) myocardial infarction |
| ICD-10-CM | I23 | Certain current complications following ST elevation (STEMI) and non-ST elevation (NSTEMI) myocardial infarction (within the 28 day period) |
| ICD-10-CM | I24 | Other acute ischemic heart diseases |
| ICD-10-CM | I25 | Chronic ischemic heart disease |
| ICD-10-CM | I46 | Cardiac arrest |
| CPT | 81493 | Coronary artery disease, mRNA, gene expression profiling by real-time RT-PCR of 23 genes, utilizing whole peripheral blood, algorithm reported as a risk score |
| HCPCS | G8039 | Coronary artery disease - patient with low-density lipoprotein documented to be greater than 100mg/dl |
| HCPCS | G8040 | Coronary artery disease - patient with low-density lipoprotein documented to be less than or equal to 100mg/dl |
| HCPCS | G8036 | Coronary artery disease patient documented to be on antiplatelet therapy |
| CPT | 0308U | Cardiology (coronary artery disease [CAD]), analysis of 3 proteins (high sensitivity [hs] troponin, adiponectin, and kidney injury molecule-1 [KIM-1]) with 3 clinical parameters (age, sex, history of cardiac intervention), plasma, algorithm reported as a risk score for obstructive CAD |
| CPT | 33510 | Coronary artery bypass, vein only; single coronary venous graft |
| CPT | 33511 | Coronary artery bypass, vein only; 2 coronary venous grafts |
| CPT | 33512 | Coronary artery bypass, vein only; 3 coronary venous grafts |
| CPT | 33513 | Coronary artery bypass, vein only; 4 coronary venous grafts |
| CPT | 33514 | Coronary artery bypass, vein only; 5 coronary venous grafts |
| CPT | 33516 | Coronary artery bypass, vein only; 6 or more coronary venous grafts |
| CPT | 33517 | Coronary artery bypass, using venous graft(s) and arterial graft(s); single vein graft (List separately in addition to code for primary procedure) |
| CPT | 33518 | Coronary artery bypass, using venous graft(s) and arterial graft(s); 2 venous grafts (List separately in addition to code for primary procedure) |
| CPT | 33519 | Coronary artery bypass, using venous graft(s) and arterial graft(s); 3 venous grafts (List separately in addition to code for primary procedure) |
| CPT | 33521 | Coronary artery bypass, using venous graft(s) and arterial graft(s); 4 venous grafts (List separately in addition to code for primary procedure) |
| CPT | 33522 | Coronary artery bypass, using venous graft(s) and arterial graft(s); 5 venous grafts (List separately in addition to code for primary procedure) |
| CPT | 33523 | Coronary artery bypass, using venous graft(s) and arterial graft(s); 6 or more venous grafts (List separately in addition to code for primary procedure) |
| CPT | 33533 | Coronary artery bypass, using arterial graft(s); single arterial graft |
| CPT | 33534 | Coronary artery bypass, using arterial graft(s); 2 coronary arterial grafts |
| CPT | 33535 | Coronary artery bypass, using arterial graft(s); 3 coronary arterial grafts |
| CPT | 33536 | Coronary artery bypass, using arterial graft(s); 4 or more coronary arterial grafts |
| CPT | 92920 | Percutaneous transluminal coronary angioplasty; single major coronary artery or branch |
| CPT | 92921 | Percutaneous transluminal coronary angioplasty; each additional branch of a major coronary artery (List separately in addition to code for primary procedure) |
| CPT | 92924 | Percutaneous transluminal coronary atherectomy, with coronary angioplasty when performed; single major coronary artery or branch |
| CPT | 92925 | Percutaneous transluminal coronary atherectomy, with coronary angioplasty when performed; each additional branch of a major coronary artery (List separately in addition to code for primary procedure) |
| CPT | 92933 | Percutaneous transluminal coronary atherectomy, with intracoronary stent, with coronary angioplasty when performed; single major coronary artery or branch |
| CPT | 92934 | Percutaneous transluminal coronary atherectomy, with intracoronary stent, with coronary angioplasty when performed; each additional branch of a major coronary artery (List separately in addition to code for primary procedure) |
| CPT | 92982 | Percutaneous transluminal coronary balloon angioplasty; single vessel |
| CPT | 92984 | Percutaneous transluminal coronary balloon angioplasty; each additional vessel (List separately in addition to code for primary procedure) |
| HCPCS | C7533 | Percutaneous transluminal coronary angioplasty, single major coronary artery or branch with transcatheter placement of radiation delivery device for subsequent coronary intravascular brachytherapy |
| HCPCS | G8033 | Prior myocardial infarction - coronary artery disease patient documented to be on beta-blocker therapy |
| HCPCS | G8034 | Prior myocardial infarction - coronary artery disease patient not documented to be on beta-blocker therapy |
| HCPCS | G8035 | Clinician documented that prior myocardial infarction - coronary artery disease patient was not eligible candidate for beta-blocker therapy measure |
| HCPCS | G8036 | Coronary artery disease patient documented to be on antiplatelet therapy |
| HCPCS | G8037 | Coronary artery disease patient not documented to be on antiplatelet therapy |
| HCPCS | G8038 | Clinician documented that coronary artery disease patient was not eligible candidate for antiplatelet therapy measure |
| HCPCS | G8041 | Clinician documented that coronary artery disease patient was not eligible candidate for low-density lipoprotein measure |
| HCPCS | G8489 | I intend to report the coronary artery disease (cad) measures group |
| HCPCS | G8498 | All quality actions for the applicable measures in the coronary artery disease (cad) measures group have been performed for this patient |
| HCPCS | S0340 | Lifestyle modification program for management of coronary artery disease, including all supportive services; first quarter / stage |
| HCPCS | S0341 | Lifestyle modification program for management of coronary artery disease, including all supportive services; second or third quarter / stage |
| HCPCS | S0342 | Lifestyle modification program for management of coronary artery disease, including all supportive services; fourth quarter / stage |
| ICD-10-CM | T82.211A | Breakdown (mechanical) of coronary artery bypass graft, initial encounter |
| ICD-10-CM | T82.211D | Breakdown (mechanical) of coronary artery bypass graft, subsequent encounter |
| ICD-10-CM | T82.211S | Breakdown (mechanical) of coronary artery bypass graft, sequela |
| ICD-10-CM | T82.212A | Displacement of coronary artery bypass graft, initial encounter |
| ICD-10-CM | T82.212D | Displacement of coronary artery bypass graft, subsequent encounter |
| ICD-10-CM | T82.212S | Displacement of coronary artery bypass graft, sequela |
| ICD-10-CM | T82.213A | Leakage of coronary artery bypass graft, initial encounter |
| ICD-10-CM | T82.213D | Leakage of coronary artery bypass graft, subsequent encounter |
| ICD-10-CM | T82.213S | Leakage of coronary artery bypass graft, sequela |
| ICD-10-CM | T82.218A | Other mechanical complication of coronary artery bypass graft, initial encounter |
| ICD-10-CM | T82.218D | Other mechanical complication of coronary artery bypass graft, subsequent encounter |
| ICD-10-CM | T82.218S | Other mechanical complication of coronary artery bypass graft, sequela |
| SNOMED CT | 468381000124109 | Atherosclerotic cardiovascular disease risk assessment score |
| SNOMED CT | 468371000124106 | Atherosclerotic cardiovascular disease risk estimator plus |
| SNOMED CT | 420006002 | Obliterative coronary artery disease |
| SNOMED CT | 373146007 | Triple vessel coronary artery disease |
| SNOMED CT | 373145006 | Double vessel coronary artery disease |
| SNOMED CT | 373144005 | Single vessel coronary artery disease |
| SNOMED CT | 371915000 | Diffuse disease of coronary artery |
| SNOMED CT | 371804009 | Left main coronary artery disease |
| SNOMED CT | 371803003 | Multi vessel coronary artery disease |
| SNOMED CT | 233844002 | Accelerated coronary artery disease in transplanted heart |
| SNOMED CT | 1293192009 | Premature coronary artery disease |
| SNOMED CT | 459701000124101 | Coronary small artery disease |
| SNOMED CT | 8957000 | Coronary artery disease |
| SNOMED CT | 282006 | Acute myocardial infarction of basal-lateral wall |
| SNOMED CT | 10273003 | Acute infarction of papillary muscle |
| SNOMED CT | 15990001 | Acute myocardial infarction of posterolateral wall |
| SNOMED CT | 30277009 | Rupture of ventricle due to acute myocardial infarction |
| SNOMED CT | 17531000119105 | Acute myocardial infarction due to left coronary artery occlusion |
| SNOMED CT | 23311000119105 | Acute myocardial infarction due to right coronary artery occlusion |
| SNOMED CT | 285981000119103 | Acute ST-segment elevation myocardial infarction involving left anterior descending coronary artery |
| SNOMED CT | 15962541000119106 | Acute ST-segment elevation myocardial infarction of anteroapical wall |
| SNOMED CT | 15713081000119108 | Acute ST-segment elevation myocardial infarction involving left main coronary artery |
| SNOMED CT | 15713121000119105 | Acute STEMI (ST-elevation myocardial infarction) due to RCA (right coronary artery) occlusion |
| SNOMED CT | 194802003 | True posterior myocardial infarction |
| SNOMED CT | 194809007 | Acute myocardial infarction of atrium |
| SNOMED CT | 233825009 | Acute Q-wave infarction - anteroseptal |
| SNOMED CT | 233826005 | Acute non-Q-wave infarction - anteroseptal |
| SNOMED CT | 233827001 | Acute Q-wave infarction - anterolateral |
| SNOMED CT | 233828006 | Acute non-Q-wave infarction - anterolateral |
| SNOMED CT | 233829003 | Acute Q-wave infarction - inferior |
| SNOMED CT | 233830008 | Acute non-Q-wave infarction - inferior |
| SNOMED CT | 233831007 | Acute Q-wave infarction - inferolateral |
| SNOMED CT | 233832000 | Acute non-Q-wave infarction - inferolateral |
| SNOMED CT | 233833005 | Acute Q-wave infarction - lateral |
| SNOMED CT | 233834004 | Acute non-Q-wave infarction - lateral |
| SNOMED CT | 233835003 | Acute widespread myocardial infarction |
| SNOMED CT | 233836002 | Acute Q-wave infarction - widespread |
| SNOMED CT | 233837006 | Acute non-Q-wave infarction - widespread |
| SNOMED CT | 233838001 | Acute posterior myocardial infarction |
| SNOMED CT | 401303003 | Acute ST-segment elevation myocardial infarction |
| SNOMED CT | 401314000 | Acute non-ST-segment elevation myocardial infarction |
| SNOMED CT | 52035003 | Acute anteroapical myocardial infarction |
| SNOMED CT | 54329005 | Acute myocardial infarction of anterior wall |
| SNOMED CT | 57054005 | Acute myocardial infarction |
| SNOMED CT | 58612006 | Acute myocardial infarction of lateral wall |
| SNOMED CT | 59063002 | Acute myocardial infarction of apical-lateral wall |
| SNOMED CT | 62695002 | Acute anteroseptal myocardial infarction |
| SNOMED CT | 64627002 | Acute myocardial infarction of high lateral wall |
| SNOMED CT | 65547006 | Acute myocardial infarction of inferolateral wall |
| SNOMED CT | 70211005 | Acute myocardial infarction of anterolateral wall |
| SNOMED CT | 70422006 | Acute subendocardial infarction |
| SNOMED CT | 70998009 | Acute myocardial infarction of posterobasal wall |
| SNOMED CT | 73795002 | Acute myocardial infarction of inferior wall |
| SNOMED CT | 76593002 | Acute myocardial infarction of inferoposterior wall |
| SNOMED CT | 79009004 | Acute myocardial infarction of septum |
| SNOMED CT | 703164000 | Acute anterior ST-segment elevation myocardial infarction |
| SNOMED CT | 304914007 | Acute Q-wave myocardial infarction |
| SNOMED CT | 307140009 | Acute non-Q-wave infarction |
| SNOMED CT | 1163440003 | Postoperative acute myocardial infarction |
| SNOMED CT | 1208872002 | Subsequent anterior non-ST-segment elevation myocardial infarction |
| SNOMED CT | 836293000 | Acute myocardial infarction of right ventricle |
| SNOMED CT | 836294006 | Acute myocardial infarction of apex of heart |
| SNOMED CT | 836295007 | Acute myocardial infarction of inferolateral wall with posterior extension |
| SNOMED CT | 840309000 | Acute ST-segment elevation myocardial infarction due to proximal left anterior descending coronary artery occlusion |
| SNOMED CT | 840312002 | Acute ST-segment elevation myocardial infarction due to mid left anterior descending coronary artery occlusion |
| SNOMED CT | 840316004 | Acute ST-segment elevation myocardial infarction due to distal left anterior descending coronary artery occlusion |
| SNOMED CT | 840609007 | Acute ST-segment elevation myocardial infarction due to occlusion of anterior descending branch of left coronary artery |
| SNOMED CT | 840680009 | Acute ST-segment elevation myocardial infarction due to left coronary artery |
| SNOMED CT | 846668006 | Acute ST-segment elevation myocardial infarction due to occlusion of diagonal branch of anterior descending branch of left coronary artery |
| SNOMED CT | 846683001 | Acute ST-segment elevation myocardial infarction due to occlusion of intermediate artery |
| SNOMED CT | 868214006 | Acute ST-segment elevation myocardial infarction due to occlusion of proximal portion of right coronary artery |
| SNOMED CT | 868217004 | Acute ST-segment elevation myocardial infarction due to occlusion of distal portion of right coronary artery |
| SNOMED CT | 868220007 | Acute ST-segment elevation myocardial infarction due to occlusion of midportion of right coronary artery |
| SNOMED CT | 868224003 | Acute ST-segment elevation myocardial infarction due to occlusion of marginal branch of right coronary artery |
| SNOMED CT | 868225002 | Acute ST-segment elevation myocardial infarction due to occlusion of posterior descending branch of right coronary artery |
| SNOMED CT | 868226001 | Acute ST-segment elevation myocardial infarction due to occlusion of posterior lateral branch of right coronary artery |
| SNOMED CT | 12238111000119106 | Acute ST-segment elevation myocardial infarction of inferolateral wall |
| SNOMED CT | 12238151000119107 | Acute ST-segment elevation myocardial infarction of inferoposterior wall |
| SNOMED CT | 15712841000119100 | Acute ST-segment elevation myocardial infarction of posterolateral wall |
| SNOMED CT | 15712881000119105 | Acute ST-segment elevation myocardial infarction of anterolateral wall |
| SNOMED CT | 15712921000119103 | Acute ST-segment elevation myocardial infarction of lateral wall |
| SNOMED CT | 15712961000119108 | ST Acute teroseptal wall segment elevation myocardial infarction of an- |
| SNOMED CT | 15713041000119103 | Acute ST-segment elevation myocardial infarction of posterior wall |
| SNOMED CT | 15713161000119100 | Acute ST-segment elevation myocardial infarction of septum |
| SNOMED CT | 15713201000119105 | Acute ST-segment elevation myocardial infarction of posterobasal wall |
| SNOMED CT | 15963181000119104 | Acute ST-segment elevation myocardial infarction due to occlusion of circumflex coronary artery |
| SNOMED CT | 1204151009 | Acute inferior non-ST-segment elevation myocardial infarction of right ventricle |
| SNOMED CT | 1204154001 | Acute anterior non-ST-segment elevation myocardial infarction with right ventricular involvement |
| SNOMED CT | 1204155000 | Acute anterior non-ST-segment elevation myocardial infarction |
| SNOMED CT | 1204152002 | Acute inferior non-ST-segment elevation myocardial infarction |
| SNOMED CT | 1204222000 | Acute non-ST segment right ventricle elevation myocardial infarction of |
| SNOMED CT | 15713001000119100 | Acute ST-segment elevation myocardial infarction of atrium |
| SNOMED CT | 44811000087108 | Acute ST-segment elevation myocardial infarction due to distal left circumflex coronary artery occlusion |
| SNOMED CT | 44821000087100 | Acute ST-segment elevation myocardial infarction due to mid left circumflex coronary artery occlusion |
| SNOMED CT | 44831000087103 | Acute ST-segment elevation myocardial infarction due to obtuse marginal branch of left circumflex coronary artery occlusion |
| SNOMED CT | 44841000087109 | Acute ST-segment elevation myocardial infarction due to posterolateral branch of left circumflex coronary artery occlusion |
| SNOMED CT | 44851000087107 | Acute ST-segment elevation myocardial infarction due to proximal left circumflex coronary artery occlusion |
| SNOMED CT | 703165004 | Acute ST-segment elevation myocardial infarction of anterior wall involving right ventricle |
| SNOMED CT | 703212004 | Acute myocardial infarction during procedure |
| SNOMED CT | 703213009 | Acute ST-segment elevation myocardial infarction of inferior wall |
| SNOMED CT | 703251009 | Acute myocardial infarction of inferior wall involving right ventricle |
| SNOMED CT | 703252002 | Acute myocardial infarction of anterior wall involving right ventricle |
| SNOMED CT | 703253007 | Acute ST-segment elevation myocardial infarction of inferior wall involving right ventricle |
| SNOMED CT | 896689003 | Acute myocardial infarction due to occlusion of circumflex branch of left coronary artery |
| SNOMED CT | 896691006 | Acute ST-segment elevation myocardial infarction due to occlusion of circumflex branch of left coronary artery |
| SNOMED CT | 896696001 | Acute ST-segment elevation myocardial infarction of apex of heart |
| SNOMED CT | 896697005 | Acute ST-segment elevation myocardial infarction of right ventricle |
|  |  |  |
| *ASCVD - Cerebrovascular Disease (inclusive of ischemic stroke and hemorrhagic stroke)* | | |
| ICD-10-CM | G45 | Transient cerebral ischemic attacks and related syndromes |
| ICD-10-CM | G46 | Vascular syndromes of brain in cerebrovascular diseases |
| ICD-10-CM | H34.0 | Vitreous prolapse |
| ICD-10-CM | I60 | Nontraumatic subarachnoid hemorrhage |
| ICD-10-CM | I61 | Nontraumatic intracerebral hemorrhage |
| ICD-10-CM | I62 | Other and unspecified nontraumatic intracranial hemorrhage |
| ICD-10-CM | I63 | Cerebral infarction |
| ICD-10-CM | I65 | Occlusion and stenosis of precerebral arteries, not resulting in cerebral infarction |
| ICD-10-CM | I66 | Occlusion and stenosis of cerebral arteries, not resulting in cerebral infarction |
| ICD-10-CM | I67 | Other cerebrovascular diseases |
| ICD-10-CM | I68 | Cerebrovascular disorders in diseases classified elsewhere |
| ICD-10-CM | I69 | Sequelae of cerebrovascular disease |
| SNOMED CT | 15982271000119104 | Weakness of right facial muscle as sequela of cerebrovascular disease |
| SNOMED CT | 15982311000119104 | Weakness of left facial muscle as sequela of cerebrovascular disease |
| SNOMED CT | 16703551000119107 | Memory deficit due to and following cerebrovascular disease |
| SNOMED CT | 1259552002 | Cerebrovascular disease due to late-delayed irradiation of brain |
| SNOMED CT | 155388006 | Cerebrovascular: [disease] or [accident] |
| SNOMED CT | 266312006 | Cerebrovascular: [disease] or [accident] |
| SNOMED CT | 195221006 | Generalized ischemic cerebrovascular disease NOS |
| SNOMED CT | 195239002 | Late effects of cerebrovascular disease |
| SNOMED CT | 290621000119101 | Cognitive deficit due to and following cerebrovascular disease |
| SNOMED CT | 308064009 | H/O: cerebrovascular disease |
| SNOMED CT | 62914000 | Cerebrovascular disease |
| SNOMED CT | 195224003 | Generalized ischemic cerebrovascular disease NOS |
| SNOMED CT | 443929000 | Small vessel cerebrovascular disease |
| SNOMED CT | 133981000119106 | Dysarthria as late effects of cerebrovascular disease |
| SNOMED CT | 133991000119109 | Fluency disorder as sequela of cerebrovascular disease |
| SNOMED CT | 140281000119108 | Hemiparesis as late effect of cerebrovascular disease |
| SNOMED CT | 145741000119101 | Apraxia as late effect of cerebrovascular disease |
| SNOMED CT | 18751000119106 | Monoplegia of leg dominant side as sequela of cerebrovascular disease |
| SNOMED CT | 18761000119108 | Monoplegia of arm dominant side as sequela of cerebrovascular disease |
| SNOMED CT | 192813004 | Cerebral degeneration due to cerebrovascular disease |
| SNOMED CT | 194497005 | [X]Other vascular syndromes of the brain in cerebrovascular diseases |
| SNOMED CT | 195219001 | Other cerebrovascular disease |
| SNOMED CT | 195237000 | Other cerebrovascular disease OS |
| SNOMED CT | 195238005 | Other cerebrovascular disease NOS |
| SNOMED CT | 195248007 | Other specified cerebrovascular disease |
| SNOMED CT | 195249004 | Cerebrovascular disease NOS |
| SNOMED CT | 195603001 | [X]Other specified cerebrovascular diseases |
| SNOMED CT | 195606009 | [X]Other cerebrovascular disorders in diseases classified elsewhere |
| SNOMED CT | 195609002 | [X]Sequelae of other and unspecified cerebrovascular diseases |
| SNOMED CT | 230738008 | Asymptomatic cerebrovascular disease |
| SNOMED CT | 26021000119107 | Vertigo as sequela of cerebrovascular disease |
| SNOMED CT | 288723005 | Acute ill-defined cerebrovascular disease |
| SNOMED CT | 29941000119105 | Ataxia as sequela of cerebrovascular disease |
| SNOMED CT | 302909007 | Diffuse cerebrovascular disease |
| SNOMED CT | 441529001 | Dysphasia as late effect of cerebrovascular disease |
| SNOMED CT | 441630004 | Aphasia as late effect of cerebrovascular disease |
| SNOMED CT | 441735003 | Sensory disorder as a late effect of cerebrovascular disease |
| SNOMED CT | 441759008 | Abnormal vision as a late effect of cerebrovascular disease |
| SNOMED CT | 703163006 | Secondary cerebrovascular disease |
| SNOMED CT | 87551000119101 | Visual disturbance as sequela of cerebrovascular disease |
| SNOMED CT | 138755007 | H/O: cerebrovascular disease |
| SNOMED CT | 155406007 | Other cerebrovascular disease |
| SNOMED CT | 155410005 | Other cerebrovascular disease NOS |
| SNOMED CT | 155411009 | Late effects of cerebrovascular disease |
| SNOMED CT | 155412002 | Cerebrovascular disease NOS |
| SNOMED CT | 161484004 | H/O: cerebrovascular disease |
| SNOMED CT | 195244009 | [X]Sequelae of other and unspecified cerebrovascular diseases |
| SNOMED CT | 195595007 | Cerebrovascular disease |
| SNOMED CT | 1010648002 | Acute vestibular syndrome due to cerebrovascular disease |
| SNOMED CT | 1260337002 | Hydrocephalus ex vacuo due to cerebrovascular disease |
|  |  |  |
| *ASCVD - Peripheral Artery Disease (inclusive of PAD itself) as lower limb amputation or peripheral revascularization)* | | |
| ICD-10-CM | I70.2 | Atherosclerosis of native arteries of the extremities |
| ICD-10-CM | I70.3 | Atherosclerosis of unspecified type of bypass graft(s) of the extremities |
| ICD-10-CM | I70.4 | Atherosclerosis of autologous vein bypass graft(s) of the extremities |
| ICD-10-CM | I70.5 | Atherosclerosis of nonautologous biological bypass graft(s) of the extremities |
| ICD-10-CM | I70.6 | Atherosclerosis of nonbiological bypass graft(s) of the extremities |
| ICD-10-CM | I70.7 | Atherosclerosis of other type of bypass graft(s) of the extremities |
| ICD-10-CM | I70.8 | Atherosclerosis of other arteries |
| ICD-10-CM | I70.91 | Generalized atherosclerosis |
| ICD-10-CM | I70.92 | Chronic total occlusion of artery of the extremities |
| ICD-10-CM | I73.1 | Thromboangiitis obliterans [Buerger's disease] |
| ICD-10-CM | I73.9 | Peripheral vascular disease, unspecified |
| ICD-10-CM | I77.7 | Other arterial dissection |
| ICD-10-CM | M48.062 | Spinal stenosis, lumbar region with neurogenic claudication |
| CPT | 93668 | Peripheral arterial disease (PAD) rehabilitation, per session |
| SNOMED CT | 840580004 | Peripheral arterial disease |
|  |  |  |
| *Chronic heart failure - Heart failure with preserved ejection fraction (HFpEF)* | | |
| ICD-10-CM | I50. 3 | Diastolic (congestive) heart failure |
|  |  |  |
| *Dyslipidaemia* |  |  |
| ICD-10-CM | E78 | Disorders of lipoprotein metabolism and other lipidemias |
| SNOMED CT | 13644009 | Hypercholesterolemia |
| SNOMED CT | 33513003 | Familial apoC-II deficiency |
| SNOMED CT | 34349009 | Familial type 5 hyperlipoproteinemia |
| SNOMED CT | 34528009 | Familial hypertriglyceridemia |
| SNOMED CT | 773649005 | Transient infantile hypertriglyceridemia and hepatosteatosis |
| SNOMED CT | 773726000 | Hypercholesterolemia due to cholesterol 7 alpha-hydroxylase deficiency |
| SNOMED CT | 15771000119109 | Familial hyperalpha lipoproteinemia |
| SNOMED CT | 114831000119107 | Hyperlipidemia caused by steroid |
| SNOMED CT | 403829002 | Familial hypercholesterolemia due to heterozygous low-density lipoprotein receptor mutation |
| SNOMED CT | 403830007 | Familial hypercholesterolemia due to homozygous low-density lipoprotein receptor mutation |
| SNOMED CT | 767133009 | Compound heterozygous familial hypercholesterolemia |
| SNOMED CT | 190774002 | Hyperlipidemia, group A |
| SNOMED CT | 238040008 | Familial combined hyperlipidemia |
| SNOMED CT | 238076009 | Primary hypercholesterolemia |
| SNOMED CT | 238077000 | Polygenic hypercholesterolemia |
| SNOMED CT | 238078005 | Familial hypercholesterolemia - homozygous |
| SNOMED CT | 238079002 | Familial hypercholesterolemia - heterozygous |
| SNOMED CT | 238080004 | Hyperalpha lipoproteinemia |
| SNOMED CT | 238081000 | Familial defective apolipoprotein B-100 |
| SNOMED CT | 238082007 | Secondary hypercholesterolemia |
| SNOMED CT | 238083002 | Primary hypertriglyceridemia |
| SNOMED CT | 238084008 | Very low-density lipoproteinemia |
| SNOMED CT | 238085009 | Fredrickson type IV hyperlipoproteinemia |
| SNOMED CT | 238087001 | Secondary hypertriglyceridemia |
| SNOMED CT | 238088006 | Primary combined hyperlipidemia |
| SNOMED CT | 238089003 | Secondary combined hyperlipidemia |
| SNOMED CT | 397915002 | Fredrickson type IIa hyperlipoproteinemia |
| SNOMED CT | 398036000 | Familial hypercholesterolemia |
| SNOMED CT | 402473001 | Sporadic primary hypertriglyceridemia |
| SNOMED CT | 402474007 | Primary polygenic type IIb combined hyperlipidemia |
| SNOMED CT | 402475008 | Primary acquired chylomicronemia |
| SNOMED CT | 402725005 | Hyperlipidemia with lipid deposition in skin |
| SNOMED CT | 402726006 | Primary chylomicronemia |
| SNOMED CT | 402727002 | Secondary hyperlipidemia |
| SNOMED CT | 402785008 | Primary genetic hyperlipidemia |
| SNOMED CT | 402786009 | Chylomicronemia syndrome |
| SNOMED CT | 402787000 | Primary genetic mixed hyperlipidemia |
| SNOMED CT | 403827000 | Familial lipoprotein lipase deficiency with type I phenotype |
| SNOMED CT | 403828005 | Familial lipoprotein lipase deficiency with type V phenotype |
| SNOMED CT | 403831006 | Familial Combined Hypercholesterolaemia |
| SNOMED CT | 426161002 | Chemically induced hyperlipidemia |
| SNOMED CT | 55822004 | Hyperlipidemia |
| SNOMED CT | 445261005 | Post-transplant hyperlipidemia |
| SNOMED CT | 267432004 | Pure hypercholesterolemia |
| SNOMED CT | 267433009 | Pure hyperglyceridemia |
| SNOMED CT | 267434003 | Mixed hyperlipidemia |
| SNOMED CT | 267435002 | Familial hyperchylomicronemia |
| SNOMED CT | 299465007 | Familial multiple lipoprotein-type hyperlipidemia |
| SNOMED CT | 302870006 | Hypertriglyceridemia |
| SNOMED CT | 129589009 | Endogenous hyperlipidemia |
| SNOMED CT | 129590000 | Exogenous hyperlipidemia |
| SNOMED CT | 129591001 | Mixed hypercholesterolemia and hypertriglyceridemia |
| SNOMED CT | 1197489003 | Familial chylomicronemia syndrome |
| SNOMED CT | 1208738002 | TMEM199 congenital disorder of glycosylation |
| SNOMED CT | 275598004 | Familial hyperlipoproteinemia, type I |
| SNOMED CT | 109041000119107 | Complex dyslipidemia |
| SNOMED CT | 1671000124102 | Dyslipidemia medication review |
| SNOMED CT | 370992007 | Dyslipidemia |
| SNOMED CT | 433031000124105 | Documentation of dyslipidemia medication action plan |
| SNOMED CT | 473234001 | Dyslipidemia medication review |
|  |  |  |
| *Hypertension* |  |  |
| ICD-10-CM | I10 | Essential (primary) hypertension |
| ICD-10-CM | I11 | Hypertensive heart disease with (congestive) heart failure. |
| ICD-10-CM | I12 | Hypertensive chronic kidney disease |
| ICD-10-CM | I13 | Hypertensive heart and chronic kidney disease |
| ICD-10-CM | I15 | Secondary hypertension |
| ICD-10-CM | H35.03 | Hypertensive retinopathy |
| ICD-10-CM | I67.4 | Hypertensive encephalopathy |
| ICD-10-CM | I16 | Hypertensive crisis |
| SNOMED CT | 1201005 | Benign essential hypertension |
| SNOMED CT | 10725009 | Benign hypertension |
| SNOMED CT | 14973001 | Renal sclerosis with hypertension |
| SNOMED CT | 28119000 | Renal hypertension |
| SNOMED CT | 31992008 | Secondary hypertension |
| SNOMED CT | 762463000 | Diastolic hypertension and systolic hypertension |
| SNOMED CT | 284981000119102 | Chronic kidney disease stage 2 due to benign hypertension |
| SNOMED CT | 284991000119104 | Chronic kidney disease stage 3 due to benign hypertension |
| SNOMED CT | 461301000124109 | Resistant hypertensive disorder |
| SNOMED CT | 1078301000112109 | Multiple drug intolerant hypertension |
| SNOMED CT | 194783001 | Malignant secondary renovascular hypertension |
| SNOMED CT | 194785008 | Benign secondary hypertension |
| SNOMED CT | 194788005 | Hypertension secondary to endocrine disorder |
| SNOMED CT | 194791005 | Hypertension secondary to drug |
| SNOMED CT | 169465000 | Hypertension induced by oral contraceptive pill |
| SNOMED CT | 371125006 | Labile essential hypertension |
| SNOMED CT | 38341003 | Hypertensive disorder |
| SNOMED CT | 39018007 | Renal arterial hypertension |
| SNOMED CT | 46481004 | Low-renin essential hypertension |
| SNOMED CT | 48146000 | Diastolic hypertension |
| SNOMED CT | 56218007 | Systolic hypertension |
| SNOMED CT | 57684003 | Parenchymal renal hypertension |
| SNOMED CT | 59621000 | Essential hypertension |
| SNOMED CT | 59720008 | Sustained diastolic hypertension |
| SNOMED CT | 65518004 | Labile diastolic hypertension |
| SNOMED CT | 73410007 | Benign secondary renovascular hypertension |
| SNOMED CT | 74451002 | Secondary diastolic hypertension |
| SNOMED CT | 78975002 | Malignant essential hypertension |
| SNOMED CT | 429457004 | Systolic essential hypertension |
| SNOMED CT | 89242004 | Malignant secondary hypertension |
| SNOMED CT | 123799005 | Renovascular hypertension |
| SNOMED CT | 123800009 | Goldblatt hypertension |
| SNOMED CT | 19769006 | High-renin essential hypertension |
| SNOMED CT | 23130000 | Paroxysmal hypertension |
| SNOMED CT | 1204139007 | Hypertension due to congenital adrenal hyperplasia |
| SNOMED CT | 1208845005 | Secondary hypertension due to congenital heart disorder |
| SNOMED CT | 1208839002 | Secondary hypertension due to renal tubular disorder |
| SNOMED CT | 871642009 | Hypertension due to aortic arch obstruction |
| SNOMED CT | 5501000119106 | Postoperative hypertension |
| SNOMED CT | 132721000119104 | Hypertensive emergency |
| SNOMED CT | 367821000119106 | Page kidney |
| SNOMED CT | 434711000124103 | Perioperative hypertension |
| SNOMED CT | 16229371000119106 | Labile systemic arterial hypertension |
| SNOMED CT | 397748008 | Hypertension with albuminuria |
| SNOMED CT | 52698002 | Transient hypertension |
| SNOMED CT | 70272006 | Malignant hypertension |
| SNOMED CT | 429198000 | Exertional hypertension |
| SNOMED CT | 443482000 | Hypertensive urgency |
| SNOMED CT | 472749004 | Coronary sinus hypertension as complication of procedure |
| SNOMED CT | 697929007 | Intermittent hypertension |
| SNOMED CT | 697930002 | Labile hypertension due to being in a clinical environment |
| SNOMED CT | 706882009 | Hypertensive crisis |
| SNOMED CT | 712832005 | Supine hypertension |
| SNOMED CT | 720568003 | Brachydactyly and arterial hypertension syndrome |
| SNOMED CT | 84094009 | Rebound hypertension |
|  |  |  |
| *Metabolic dysfunction-associated steatotic liver disease (MASLD) / Metabolic dysfunction-associated steatohepatitis (MASH)* | | |
| ICD-10-CM | K76.0 | Fatty (change of) liver, not elsewhere classified |
| ICD-10-CM | K75.8 | Other specified inflammatory liver diseases |
| SNOMED CT | 722866000 | Non-alcoholic fatty liver disease without non-alcoholic steatohepatitis |
| SNOMED CT | 79720007 | Chronic nonalcoholic liver disease |
| SNOMED CT | 442685003 | Nonalcoholic steatohepatitis |
| SNOMED CT | 1231824009 | Non-alcoholic fatty liver disease |
| SNOMED CT | 197315008 | Non-alcoholic fatty liver |
|  |  |  |
| *Metabolic syndrome* |  |  |
| ICD-10-CM | E88.810 | Metabolic syndrome |
| SNOMED CT | 237602007 | Metabolic syndrome X |
|  |  |  |
| *Obstructive Sleep Apnea (OSA)* |  |  |
| ICD-10-CM | G47.33 | Obstructive Sleep Apnea |
|  |  |  |
| *Osteoarthritis (OA) knee and/or hip* | | |
| ICD-10-CM | M16 | Osteoarthritis of hip |
| ICD-10-CM | M17 | Osteoarthritis of knee |
|  |  |  |
| *Prediabetes* |  |  |
| ICD-10-CM | R73.03 | Prediabetes |
| SNOMED CT | 714628002 | Prediabetes |
| SNOMED CT | 15777000 | Prediabetes |

Abbreviations: ASCVD = atherosclerotic cardiovascular disease; CABG = coronary artery bypass graft; CPT = Current Procedural Terminology; HCPCS = Healthcare Common Procedure Coding System; H/O = history of; ICD-10-CM = International Classification of Diseases, 10^th^ edition, Clinical Modification; MI = myocardial infarction; NOS = not otherwise specified; PCI = percutaneous coronary intervention; PTCA = percutaneous transluminal coronary angioplasty; OS = otherwise specified; SNOMED CT = Systematized Nomenclature of Medicine – Clinical Terms.

### Supplemental Table 3. Diagnosis, procedure, and drug codes used to identify diabetes

| **Code System** | **Code** | **Concept Name** |
| --- | --- | --- |
| *Diabetes* |  |  |
| ICD-10-CM | E08 | Diabetes mellitus due to underlying condition |
| ICD-10-CM | E09 | Drug or chemical induced diabetes mellitus |
| ICD-10-CM | E10 | Type 1 diabetes mellitus |
| ICD-10-CM | E11 | Type 2 diabetes mellitus |
| ICD-10-CM | E13 | Other specified diabetes mellitus |
| ICD-10-CM | O24 | Diabetes mellitus in pregnancy, childbirth, and the puerperium |
| ICD-10-CM | O9981 | Abnormal glucose complicating pregnancy, childbirth and the puerperium |
| ICD-10-CM | Z86.32 | Personal history of gestational diabetes |
| SNOMED CT | 721000119107 | Chronic kidney disease stage 4 due to type 2 diabetes mellitus |
| SNOMED CT | 731000119105 | Chronic kidney disease stage 3 due to type 2 diabetes mellitus |
| SNOMED CT | 741000119101 | Chronic kidney disease stage 2 due to type 2 diabetes mellitus |
| SNOMED CT | 751000119104 | Chronic kidney disease stage 1 due to type 2 diabetes mellitus |
| SNOMED CT | 771000119108 | Chronic kidney disease due to type 2 diabetes mellitus |
| SNOMED CT | 71421000119105 | Hypertension in chronic kidney disease due to type 2 diabetes mellitus |
| SNOMED CT | 71701000119105 | Hypertension in chronic kidney disease due to type 1 diabetes mellitus |
| SNOMED CT | 90721000119101 | Chronic kidney disease stage 1 due to type 1 diabetes mellitus |
| SNOMED CT | 90731000119103 | Chronic kidney disease stage 2 due to type 1 diabetes mellitus |
| SNOMED CT | 90741000119107 | Chronic kidney disease stage 3 due to type 1 diabetes mellitus |
| SNOMED CT | 90751000119109 | Chronic kidney disease stage 4 due to type 1 diabetes mellitus |
| SNOMED CT | 90761000119106 | Chronic kidney disease stage 5 due to type 1 diabetes mellitus |
| SNOMED CT | 90771000119100 | End stage renal disease on dialysis due to type 1 diabetes mellitus |
| SNOMED CT | 90791000119104 | End stage renal disease on dialysis due to type 2 diabetes mellitus |
| SNOMED CT | 96441000119101 | Chronic kidney disease due to type 1 diabetes mellitus |
| SNOMED CT | 127991000119101 | Hypertension concurrent and due to end stage renal disease on dialysis due to type 2 diabetes mellitus |
| SNOMED CT | 128001000119105 | Hypertension concurrent and due to end stage renal disease on dialysis due to type 1 diabetes mellitus |
| SNOMED CT | 140101000119109 | Hypertension in chronic kidney disease stage 5 due to type 2 diabetes mellitus |
| SNOMED CT | 140111000119107 | Hypertension in chronic kidney disease stage 4 due to type 2 diabetes mellitus |
| SNOMED CT | 140121000119100 | Hypertension in chronic kidney disease stage 3 due to type 2 diabetes mellitus |
| SNOMED CT | 140131000119102 | Hypertension in chronic kidney disease stage 2 due to type 2 diabetes mellitus |
| SNOMED CT | 368421000119108 | Chronic kidney disease stage 1 due to drug induced diabetes mellitus |
| SNOMED CT | 368431000119106 | Chronic kidney disease stage 2 due to drug induced diabetes mellitus |
| SNOMED CT | 368441000119102 | Chronic kidney disease stage 3 due to drug induced diabetes mellitus |
| SNOMED CT | 368451000119100 | Chronic kidney disease stage 4 due to drug induced diabetes mellitus |
| SNOMED CT | 368461000119103 | Chronic kidney disease stage 5 due to drug induced diabetes mellitus |
| SNOMED CT | 368471000119109 | End stage renal disease on dialysis due to drug induced diabetes mellitus |
| *GLP-1 glucose-lowering medications* | | |
| *Dulaglutide (Trulicity)* | | |
| RxNorm | 1551291 | dulaglutide |
| RxNorm | 1551294 | dulaglutide Prefilled Syringe |
| RxNorm | 1551298 | dulaglutide Prefilled Syringe [Trulicity] |
| RxNorm | 1551292 | dulaglutide 1·5 MG/ML |
| RxNorm | 1551293 | dulaglutide Injectable Product |
| RxNorm | 1551295 | 0·5 ML dulaglutide 1·5 MG/ML Auto-Injector |
| RxNorm | 1551296 | Trulicity |
| RxNorm | 1551297 | dulaglutide 1·5 MG/ML [Trulicity] |
| RxNorm | 1551299 | Trulicity Injectable Product |
| RxNorm | 1551300 | 0·5 ML dulaglutide 1·5 MG/ML Auto-Injector [Trulicity] |
| RxNorm | 1551301 | dulaglutide 1·5 MG/ML Auto-Injector |
| RxNorm | 1551302 | dulaglutide 1·5 MG/ML Auto-Injector [Trulicity] |
| RxNorm | 1551303 | dulaglutide 3 MG/ML |
| RxNorm | 1551304 | 0·5 ML dulaglutide 3 MG/ML Auto-Injector |
| RxNorm | 1551305 | dulaglutide 3 MG/ML [Trulicity] |
| RxNorm | 1551306 | 0·5 ML dulaglutide 3 MG/ML Auto-Injector [Trulicity] |
| RxNorm | 1551307 | dulaglutide 3 MG/ML Auto-Injector |
| RxNorm | 1551308 | dulaglutide 3 MG/ML Auto-Injector [Trulicity] |
| RxNorm | 1649584 | dulaglutide Auto-Injector |
| RxNorm | 1649586 | dulaglutide Auto-Injector [Trulicity] |
| RxNorm | 2395776 | dulaglutide 6 MG/ML |
| RxNorm | 2395777 | 0·5 ML dulaglutide 6 MG/ML Auto-Injector |
| RxNorm | 2395778 | dulaglutide 6 MG/ML [Trulicity] |
| RxNorm | 2395779 | 0·5 ML dulaglutide 6 MG/ML Auto-Injector [Trulicity] |
| RxNorm | 2395780 | dulaglutide 6 MG/ML Auto-Injector |
| RxNorm | 2395781 | dulaglutide 6 MG/ML Auto-Injector [Trulicity] |
| RxNorm | 2395782 | dulaglutide 9 MG/ML |
| RxNorm | 2395783 | 0·5 ML dulaglutide 9 MG/ML Auto-Injector |
| RxNorm | 2395784 | dulaglutide 9 MG/ML [Trulicity] |
| RxNorm | 2395785 | 0·5 ML dulaglutide 9 MG/ML Auto-Injector [Trulicity] |
| RxNorm | 2395786 | dulaglutide 9 MG/ML Auto-Injector |
| RxNorm | 2395787 | dulaglutide 9 MG/ML Auto-Injector [Trulicity] |
| SNOMED CT | 714080005 | Dulaglutide |
| SNOMED CT | 714081009 | Dulaglutide-containing product |
| SNOMED CT | 775712006 | Dulaglutide only product |
| SNOMED CT | 1010536005 | Dulaglutide 1·5 mg/mL solution for injection |
| SNOMED CT | 1010537001 | Dulaglutide 3 mg/mL solution for injection |
| SNOMED CT | 1010538006 | Dulaglutide 6 mg/mL solution for injection |
| SNOMED CT | 1010539003 | Dulaglutide 9 mg/mL solution for injection |
| SNOMED CT | 1010540001 | Dulaglutide-containing product in parenteral dose form |
| SNOMED CT | 1010541002 | Dulaglutide only product in parenteral dose form |
| NDC | 545680433 | dulaglutide ·75mg/·5mL SUBCUTANEOUS INJECTION, SOLUTION [trulicity] |
| NDC | 54568043363 | 0·5 ML dulaglutide 1·5 MG/ML Auto-Injector [Trulicity] |
| NDC | 54568043371 | 0·5 ML dulaglutide 1·5 MG/ML Auto-Injector [Trulicity] |
| NDC | 545680434 | dulaglutide 1·5mg/·5mL SUBCUTANEOUS INJECTION, SOLUTION [trulicity] |
| NDC | 54568043463 | 0·5 ML dulaglutide 3 MG/ML Auto-Injector [Trulicity] |
| NDC | 54568043471 | 0·5 ML dulaglutide 3 MG/ML Auto-Injector [Trulicity] |
| NDC | 000021433 | dulaglutide ·75mg/·5mL SUBCUTANEOUS INJECTION, SOLUTION |
| NDC | 00002143301 | 0·5 ML dulaglutide 1·5 MG/ML Auto-Injector [Trulicity] |
| NDC | 00002143361 | 0·5 ML dulaglutide 1·5 MG/ML Auto-Injector [Trulicity] |
| NDC | 00002143380 | 0·5 ML dulaglutide 1·5 MG/ML Auto-Injector [Trulicity] |
| NDC | 000021434 | dulaglutide 1·5mg/·5mL SUBCUTANEOUS INJECTION, SOLUTION |
| NDC | 00002143401 | 0·5 ML dulaglutide 3 MG/ML Auto-Injector [Trulicity] |
| NDC | 00002143461 | 0·5 ML dulaglutide 3 MG/ML Auto-Injector [Trulicity] |
| NDC | 00002143480 | 0·5 ML dulaglutide 3 MG/ML Auto-Injector [Trulicity] |
| NDC | 000022236 | dulaglutide 3mg/·5mL SUBCUTANEOUS INJECTION, SOLUTION |
| NDC | 00002223601 | 0·5 ML dulaglutide 6 MG/ML Auto-Injector [Trulicity] |
| NDC | 00002223661 | 0·5 ML dulaglutide 6 MG/ML Auto-Injector [Trulicity] |
| NDC | 00002223680 | 0·5 ML dulaglutide 6 MG/ML Auto-Injector [Trulicity] |
| NDC | 000023182 | dulaglutide 4·5mg/·5mL SUBCUTANEOUS INJECTION, SOLUTION |
| NDC | 00002318201 | 0·5 ML dulaglutide 9 MG/ML Auto-Injector [Trulicity] |
| NDC | 00002318261 | 0·5 ML dulaglutide 9 MG/ML Auto-Injector [Trulicity] |
| NDC | 00002318280 | 0·5 ML dulaglutide 9 MG/ML Auto-Injector [Trulicity] |
| NDC | 500903483 | dulaglutide 1·5mg/·5mL SUBCUTANEOUS INJECTION, SOLUTION |
| NDC | 50090348300 | 0·5 ML dulaglutide 3 MG/ML Auto-Injector [Trulicity] |
| NDC | 500903484 | dulaglutide ·75mg/·5mL SUBCUTANEOUS INJECTION, SOLUTION |
| NDC | 50090348400 | 0·5 ML dulaglutide 1·5 MG/ML Auto-Injector [Trulicity] |
| NDC | 500905467 | dulaglutide 3mg/·5mL SUBCUTANEOUS INJECTION, SOLUTION |
| NDC | 50090546700 | 0·5 ML dulaglutide 6 MG/ML Auto-Injector [Trulicity] |
| NDC | 500906453 | dulaglutide ·75mg/·5mL SUBCUTANEOUS INJECTION, SOLUTION |
| NDC | 5009064530 | 0·5 ML dulaglutide 1·5 MG/ML Auto-Injector [Trulicity] |
| NDC | 500906456 | dulaglutide 1·5mg/·5mL SUBCUTANEOUS INJECTION, SOLUTION |
| NDC | 5009064560 | 0·5 ML dulaglutide 3 MG/ML Auto-Injector [Trulicity] |
| NDC | 500906571 | dulaglutide 3mg/·5mL SUBCUTANEOUS INJECTION, SOLUTION |
| NDC | 5009065710 | 0·5 ML dulaglutide 6 MG/ML Auto-Injector [Trulicity] |
| *Exenatide (e.g., Bydureon, Byetta)* | | |
| RxNorm | 60548 | exenatide |
| RxNorm | 1242962 | exenatide Injectable Suspension |
| RxNorm | 1544917 | exenatide Prefilled Syringe [Bydureon] |
| RxNorm | 604750 | exenatide 0·250 MG/ML Injectable Solution |
| RxNorm | 604749 | exenatide Injectable Solution |
| RxNorm | 604752 | exenatide 0·250 MG/ML [Byetta] |
| RxNorm | 604748 | exenatide 0·250 MG/ML |
| RxNorm | 604753 | exenatide Injectable Solution [Byetta] |
| RxNorm | 604754 | exenatide 0·250 MG/ML Injectable Solution [Byetta] |
| RxNorm | 847909 | exenatide Prefilled Syringe |
| RxNorm | 847912 | exenatide Prefilled Syringe [Byetta] |
| RxNorm | 1163790 | exenatide Injectable Product |
| RxNorm | 1169415 | Byetta Injectable Product |
| RxNorm | 1242961 | exenatide 3·08 MG/ML |
| RxNorm | 1242963 | exenatide 2 MG Injection |
| RxNorm | 1242964 | Bydureon |
| RxNorm | 1242965 | exenatide 3·08 MG/ML [Bydureon] |
| RxNorm | 1242967 | Bydureon Injectable Product |
| RxNorm | 1242968 | exenatide 2 MG Injection [Bydureon] |
| RxNorm | 1359802 | exenatide 0·005 MG/ACTUAT Pen Injector [Byetta] |
| RxNorm | 1359979 | exenatide 0·01 MG/ACTUAT Pen Injector [Byetta] |
| RxNorm | 1360454 | exenatide 0·01 MG/ACTUAT Pen Injector |
| RxNorm | 1359640 | exenatide 0·005 MG/ACTUAT Pen Injector |
| RxNorm | 1544919 | exenatide 3·08 MG/ML Pen Injector |
| RxNorm | 1544920 | exenatide 3·08 MG/ML Pen Injector [Bydureon] |
| RxNorm | 1544916 | 0·65 ML exenatide 3·08 MG/ML Pen Injector |
| RxNorm | 1544918 | 0·65 ML exenatide 3·08 MG/ML Pen Injector [Bydureon] |
| RxNorm | 1653610 | exenatide 2 MG |
| RxNorm | 1653611 | exenatide Injection |
| RxNorm | 1653613 | exenatide 2 MG [Bydureon] |
| RxNorm | 1653614 | exenatide Injection [Bydureon] |
| RxNorm | 1653616 | exenatide Pen Injector |
| RxNorm | 1653619 | exenatide Pen Injector [Bydureon] |
| RxNorm | 1653625 | exenatide Pen Injector [Byetta] |
| RxNorm | 1990864 | exenatide 2·35 MG/ML |
| RxNorm | 1990865 | exenatide Auto-Injector |
| RxNorm | 1990866 | 0·85 ML exenatide 2·35 MG/ML Auto-Injector |
| RxNorm | 1990867 | exenatide 2·35 MG/ML [Bydureon] |
| RxNorm | 1990868 | exenatide Auto-Injector [Bydureon] |
| RxNorm | 1990869 | 0·85 ML exenatide 2·35 MG/ML Auto-Injector [Bydureon] |
| RxNorm | 1990870 | exenatide 2·35 MG/ML Auto-Injector |
| RxNorm | 1990871 | exenatide 2·35 MG/ML Auto-Injector [Bydureon] |
| RxNorm | 847908 | exenatide 0·01 MG/ACTUAT |
| RxNorm | 847910 | 60 ACTUAT exenatide 0·01 MG/ACTUAT Pen Injector |
| RxNorm | 847911 | exenatide 0·01 MG/ACTUAT [Byetta] |
| RxNorm | 847913 | 60 ACTUAT exenatide 0·01 MG/ACTUAT Pen Injector [Byetta] |
| RxNorm | 847914 | exenatide 0·005 MG/ACTUAT |
| RxNorm | 847915 | 60 ACTUAT exenatide 0·005 MG/ACTUAT Pen Injector |
| RxNorm | 847916 | exenatide 0·005 MG/ACTUAT [Byetta] |
| RxNorm | 847917 | 60 ACTUAT exenatide 0·005 MG/ACTUAT Pen Injector [Byetta] |
| RxNorm | 744863 | exenatide 0·25 MG/ML Injectable Solution |
| RxNorm | 744862 | exenatide 0·25 MG/ML |
| RxNorm | 744864 | exenatide 0·25 MG/ML [Byetta] |
| RxNorm | 744865 | exenatide 0·25 MG/ML Injectable Solution [Byetta] |
| RxNorm | 1242966 | exenatide Injectable Suspension [Bydureon] |
| RxNorm | 604751 | Byetta |
| SNOMED CT | 416525003 | Exenatide 250mcg/mL injection solution prefilled pen |
| SNOMED CT | 416859008 | Exenatide |
| SNOMED CT | 417734003 | Exenatide-containing product |
| SNOMED CT | 438958002 | Exenatide 250micrograms/mL injection solution 2·4mL prefilled pen |
| SNOMED CT | 440246006 | Exenatide 250micrograms/mL injection solution 1·2mL prefilled pen |
| SNOMED CT | 775913009 | Exenatide only product |
| SNOMED CT | 1155637009 | Exenatide 250 microgram/mL solution for injection |
| SNOMED CT | 1155638004 | Exenatide-containing product in parenteral dose form |
| SNOMED CT | 1155639007 | Exenatide only product in parenteral dose form |
| SNOMED CT | 1237218000 | Exenatide 2 mg powder for prolonged-release suspension for injection vial |
| NDC | 003106512 | exenatide 250ug/mL SUBCUTANEOUS INJECTION |
| NDC | 00310651201 | 60 ACTUAT exenatide 0·005 MG/ACTUAT Pen Injector [Byetta] |
| NDC | 00310651285 | 60 ACTUAT exenatide 0·005 MG/ACTUAT Pen Injector |
| NDC | 003106520 | exenatide KIT |
| NDC | 00310652004 | exenatide 2 MG Injection [Bydureon] |
| NDC | 003106524 | exenatide 250ug/mL SUBCUTANEOUS INJECTION |
| NDC | 00310652401 | 60 ACTUAT exenatide 0·01 MG/ACTUAT Pen Injector [Byetta] |
| NDC | 003106530 | exenatide 2mg/·65mL SUBCUTANEOUS INJECTION, SUSPENSION, EXTENDED RELEASE |
| NDC | 00310653001 | 0·65 ML exenatide 3·08 MG/ML Pen Injector [Bydureon] |
| NDC | 00310653004 | 0·65 ML exenatide 3·08 MG/ML Pen Injector [Bydureon] |
| NDC | 00310653085 | 0·65 ML exenatide 3·08 MG/ML Pen Injector [Bydureon] |
| NDC | 003106540 | exenatide 2mg/·85mL SUBCUTANEOUS INJECTION, SUSPENSION, EXTENDED RELEASE |
| NDC | 00310654001 | 0·85 ML exenatide 2·35 MG/ML Auto-Injector [Bydureon] |
| NDC | 00310654004 | 0·85 ML exenatide 2·35 MG/ML Auto-Injector [Bydureon] |
| NDC | 00310654085 | 0·85 ML exenatide 2·35 MG/ML Auto-Injector [Bydureon] |
| NDC | 548685384 | exenatide 250ug/mL SUBCUTANEOUS INJECTION |
| NDC | 54868538400 | 60 ACTUAT exenatide 0·005 MG/ACTUAT Pen Injector [Byetta] |
| NDC | 54868538401 | 60 ACTUAT exenatide 0·01 MG/ACTUAT Pen Injector [Byetta] |
| NDC | 54868538402 | 60 ACTUAT exenatide 0·005 MG/ACTUAT Prefilled Syringe [Byetta] |
| NDC | 68258894701 | 60 ACTUAT exenatide 0·005 MG/ACTUAT Prefilled Syringe [Byetta] |
| NDC | 68258894802 | 60 ACTUAT exenatide 0·01 MG/ACTUAT Prefilled Syringe [Byetta] |
| NDC | 00002021007 | 60 ACTUAT exenatide 0·005 MG/ACTUAT Prefilled Syringe [Byetta] |
| NDC | 00002021008 | 60 ACTUAT exenatide 0·01 MG/ACTUAT Prefilled Syringe [Byetta] |
| NDC | 00002021009 | 60 ACTUAT exenatide 0·005 MG/ACTUAT Prefilled Syringe [Byetta] |
| NDC | 66029021007 | 60 ACTUAT exenatide 0·005 MG/ACTUAT Prefilled Syringe [Byetta] |
| NDC | 66029021008 | 60 ACTUAT exenatide 0·01 MG/ACTUAT Prefilled Syringe [Byetta] |
| NDC | 667800210 | exenatide 250ug/mL SUBCUTANEOUS INJECTION [byetta] |
| NDC | 66780021007 | 60 ACTUAT exenatide 0·005 MG/ACTUAT Pen Injector [Byetta] |
| NDC | 66780021008 | 60 ACTUAT exenatide 0·01 MG/ACTUAT Prefilled Syringe [Byetta] |
| NDC | 66780021009 | 60 ACTUAT exenatide 0·005 MG/ACTUAT Pen Injector [Byetta] |
| NDC | 667800212 | exenatide 250ug/mL SUBCUTANEOUS INJECTION [byetta] |
| NDC | 66780021201 | 60 ACTUAT exenatide 0·01 MG/ACTUAT Pen Injector [Byetta] |
| NDC | 667800219 | exenatide KIT [bydureon] |
| NDC | 66780021902 | exenatide 2 MG Injection [Bydureon] |
| NDC | 66780021904 | exenatide 2 MG Injection [Bydureon] |
| NDC | 667800226 | exenatide KIT [bydureon] |
| NDC | 66780022601 | exenatide 2 MG Injection [Bydureon] |
| NDC | 66914103504 | 60 ACTUAT exenatide 0·005 MG/ACTUAT Prefilled Syringe [Byetta] |
| NDC | 66914103505 | 60 ACTUAT exenatide 0·01 MG/ACTUAT Prefilled Syringe [Byetta] |
| *Liraglutide (Victoza)* | | |
| RxNorm | 897125 | liraglutide Prefilled Syringe [Victoza] |
| RxNorm | 897123 | Victoza |
| RxNorm | 897124 | liraglutide 6 MG/ML [Victoza] |
| RxNorm | 897126 | 3 ML liraglutide 6 MG/ML Pen Injector [Victoza] |
| RxNorm | 1186578 | Victoza Injectable Product |
| RxNorm | 1360495 | liraglutide 6 MG/ML Pen Injector [Victoza] |
| RxNorm | 1653597 | liraglutide Pen Injector [Victoza] |
| NDC | 54569650700 | 3 ML liraglutide 6 MG/ML Pen Injector [Victoza] |
| NDC | 001694060 | liraglutide 6mg/mL SUBCUTANEOUS INJECTION |
| NDC | 00169406012 | 3 ML liraglutide 6 MG/ML Pen Injector [Victoza] |
| NDC | 00169406013 | 3 ML liraglutide 6 MG/ML Pen Injector [Victoza] |
| NDC | 00169406090 | 3 ML liraglutide 6 MG/ML Pen Injector [Victoza] |
| NDC | 00169406097 | 3 ML liraglutide 6 MG/ML Pen Injector [Victoza] |
| NDC | 00169406098 | 3 ML liraglutide 6 MG/ML Pen Injector [Victoza] |
| NDC | 00169406099 | 3 ML liraglutide 6 MG/ML Pen Injector [Victoza] |
| NDC | 500902853 | liraglutide 6mg/mL SUBCUTANEOUS INJECTION |
| NDC | 50090285300 | 3 ML liraglutide 6 MG/ML Pen Injector [Victoza] |
| NDC | 500904503 | liraglutide 6mg/mL SUBCUTANEOUS INJECTION |
| NDC | 50090450300 | 3 ML liraglutide 6 MG/ML Pen Injector [Victoza] |
| *Liraglutide (Xultophy)* | | |
| RxNorm | 1860168 | Xultophy |
| RxNorm | 1860169 | insulin degludec 100 UNT/ML / liraglutide 3·6 MG/ML [Xultophy] |
| RxNorm | 1860170 | insulin degludec / liraglutide Pen Injector [Xultophy] |
| RxNorm | 1860171 | Xultophy Injectable Product |
| RxNorm | 1860172 | 3 ML insulin degludec 100 UNT/ML / liraglutide 3·6 MG/ML Pen Injector [Xultophy] |
| RxNorm | 1860174 | insulin degludec 100 UNT/ML / liraglutide 3·6 MG/ML Pen Injector [Xultophy] |
| NDC | 001692911 | (insulin degludec and liraglutide) 3·6mg/mL / 100[iU]/mL SUBCUTANEOUS INJECTION, SOLUTION |
| NDC | 00169291115 | 3 ML insulin degludec 100 UNT/ML / liraglutide 3·6 MG/ML Pen Injector [Xultophy] |
| NDC | 00169291190 | 3 ML insulin degludec 100 UNT/ML / liraglutide 3·6 MG/ML Pen Injector [Xultophy] |
| NDC | 00169291197 | 3 ML insulin degludec 100 UNT/ML / liraglutide 3·6 MG/ML Pen Injector [Xultophy] |
| *Liraglutide (non-specific)* | | |
| RxNorm | 475968 | liraglutide |
| RxNorm | 897121 | liraglutide Prefilled Syringe |
| RxNorm | 897120 | liraglutide 6 MG/ML |
| RxNorm | 897122 | 3 ML liraglutide 6 MG/ML Pen Injector |
| RxNorm | 1163230 | liraglutide Injectable Product |
| RxNorm | 1360105 | liraglutide 6 MG/ML Pen Injector |
| RxNorm | 1653594 | liraglutide Pen Injector |
| RxNorm | 1727493 | insulin degludec / liraglutide |
| RxNorm | 1860164 | liraglutide 3·6 MG/ML |
| RxNorm | 1860165 | insulin degludec / liraglutide Injectable Product |
| RxNorm | 1860166 | insulin degludec / liraglutide Pen Injector |
| RxNorm | 1860167 | 3 ML insulin degludec 100 UNT/ML / liraglutide 3·6 MG/ML Pen Injector |
| RxNorm | 1860173 | insulin degludec Pen Injector |
| SNOMED CT | 444828003 | Liraglutide |
| SNOMED CT | 444829006 | Liraglutide 6mg/mL injection solution |
| SNOMED CT | 444907006 | Liraglutide-containing product |
| SNOMED CT | 715778002 | Insulin degludec- and liraglutide-containing product |
| SNOMED CT | 715779005 | Insulin degludec- and liraglutide-containing product in parenteral dose form |
| SNOMED CT | 776341007 | Insulin degludec and liraglutide only product |
| SNOMED CT | 776548002 | Liraglutide only product |
| SNOMED CT | 779559007 | Insulin degludec and liraglutide only product in parenteral dose form |
| SNOMED CT | 789390003 | Liraglutide-containing product in parenteral dose form |
| SNOMED CT | 789391004 | Liraglutide only product in parenteral dose form |
| SNOMED CT | 1236758005 | Liraglutide 6 mg/mL solution for injection |
| *Lixisenatide (e.g., Adlyzin, Soliqua)* | | |
| RxNorm | 1440051 | lixisenatide |
| RxNorm | 1440054 | Lixisenatide Injectable Solution |
| RxNorm | 1440055 | Lixisenatide 0·1 MG/ML Injectable Solution |
| RxNorm | 1440057 | Lixisenatide 0·05 MG/ML Injectable Solution |
| RxNorm | 1440052 | lixisenatide 0·1 MG/ML |
| RxNorm | 1440053 | lixisenatide Injectable Product |
| RxNorm | 1440056 | lixisenatide 0·05 MG/ML |
| RxNorm | 1858991 | lixisenatide 0·033 MG/ML |
| RxNorm | 1858992 | insulin glargine / lixisenatide Injectable Product |
| RxNorm | 1858993 | insulin glargine / lixisenatide Pen Injector |
| RxNorm | 1858994 | insulin glargine / lixisenatide |
| RxNorm | 1858995 | 3 ML insulin MG/ML Pen Injector 100 UNT/ML / lixisenatide 0·033 MG/ML Pen Injector |
| RxNorm | 1858996 | Soliqua |
| RxNorm | 1858997 | insulin glargine 100 UNT/ML / lixisenatide 0·033 MG/ML [Soliqua] |
| RxNorm | 1858998 | insulin glargine / lixisenatide Pen Injector [Soliqua] |
| RxNorm | 1858999 | Soliqua Injectable Product |
| RxNorm | 1859000 | 3 ML insulin glargine 100 UNT/ML / lixisenatide 0·033 MG/ML Pen Injector [Soliqua] |
| RxNorm | 1859001 | insulin glargine 100 UNT/ML / lixisenatide 0·033 MG/ML Pen Injector |
| RxNorm | 1859002 | insulin glargine 100 UNT/ML / lixisenatide 0·033 MG/ML Pen Injector [Soliqua] |
| RxNorm | 1803885 | lixisenatide Pen Injector |
| RxNorm | 1803886 | lixisenatide 0·05 MG/ML Pen Injector |
| RxNorm | 1803887 | Adlyxin |
| RxNorm | 1803888 | lixisenatide 0·05 MG/ML [Adlyxin] |
| RxNorm | 1803889 | lixisenatide Pen Injector [Adlyxin] |
| RxNorm | 1803890 | Adlyxin Injectable Product |
| RxNorm | 1803891 | lixisenatide 0·05 MG/ML PenInjector [Adlyxin] |
| RxNorm | 1803892 | 3 ML lixisenatide 0·05 MG/ML Pen Injector |
| RxNorm | 1803893 | 3 ML lixisenatide 0·05 MG/ML Pen Injector [Adlyxin] |
| RxNorm | 1803894 | 3 ML lixisenatide 0·1 MG/ML Pen Injector |
| RxNorm | 1803895 | lixisenatide 0·1 MG/ML [Adlyxin] |
| RxNorm | 1803896 | 3 ML lixisenatide 0·1 MG/ML Pen Injector [Adlyxin] |
| RxNorm | 1803897 | lixisenatide 0·1 MG/ML Pen Injector |
| RxNorm | 1803898 | lixisenatide 0·1 MG/ML Pen Injector [Adlyxin] |
| RxNorm | 1803902 | {1 (3 ML lixisenatide 0·05 MG/ML Pen Injector) / 1 (3 ML lixisenatide 0·1 MG/ML Pen Injector) } Pack |
| RxNorm | 1803903 | {1 (3 ML lixisenatide 0·05 MG/ML Pen Injector [Adlyxin]) / 1 (3 ML lixisenatide 0·1 MG/ML Pen Injector [Adlyxin]) } Pack [Adlyxin Starter Kit] |
| SNOMED CT | 763570007 | Lixisenatide-containing product |
| SNOMED CT | 764330009 | Lixisenatide-containing product in parenteral dose form |
| SNOMED CT | 708808004 | Lixisenatide |
| SNOMED CT | 776560001 | Lixisenatide only product |
| SNOMED CT | 779728007 | Lixisenatide only product in parenteral dose form |
| NDC | 00024574000 | 3 ML lixisenatide 0·1 MG/ML Pen Injector [Adlyxin] |
| NDC | 00024574101 | 3 ML lixisenatide 0·05 MG/ML Pen Injector [Adlyxin] |
| NDC | 000245745 | lixisenatide KIT |
| NDC | 00024574502 | {1 (3 ML lixisenatide 0·05 MG/ML Pen Injector [Adlyxin]) / 1 (3 ML lixisenatide 0·1 MG/ML Pen Injector [Adlyxin]) } Pack [Adlyxin Starter Kit] |
| NDC | 000245747 | lixisenatide 100ug/mL SUBCUTANEOUS INJECTION, SOLUTION |
| NDC | 00024574702 | 3 ML lixisenatide 0·1 MG/ML Pen Injector [Adlyxin] |
| NDC | 000245761 | insulin glargine and lixisenatide 100 U/mL / 33ug/mL SUBCUTANEOUS INJECTION, SOLUTION |
| NDC | 00024576101 | 3 ML insulin glargine 100 UNT/ML / lixisenatide 0·033 MG/ML Pen Injector [Soliqua] |
| NDC | 00024576102 | 3 ML insulin glargine 100 UNT/ML / lixisenatide 0·033 MG/ML Pen Injector [Soliqua] |
| NDC | 00024576105 | 3 ML insulin glargine 100 UNT/ML / lixisenatide 0·033 MG/ML Pen Injector [Soliqua] |
| NDC | 00024576302 | 3 ML insulin glargine 100 UNT/ML / lixisenatide 0·033 MG/ML Pen Injector [Soliqua] |
| *Semaglutide (Ozempic)* | | |
| NDC | 001694130 | semaglutide 1·34mg/mL SUBCUTANEOUS INJECTION, SOLUTION |
| NDC | 00169413001 | 3 ML semaglutide 1·34 MG/ML Pen Injector [Ozempic] |
| NDC | 00169413013 | 3 ML semaglutide 1·34 MG/ML Pen Injector [Ozempic] |
| NDC | 001694132 | semaglutide 1·34mg/mL SUBCUTANEOUS INJECTION, SOLUTION |
| NDC | 00169413211 | 0·25 MG, 0·5 MG Dose 1·5 ML semaglutide 1·34 MG/ML Pen Injector [Ozempic] |
| NDC | 00169413212 | 0·25 MG, 0·5 MG Dose 1·5 ML semaglutide 1·34 MG/ML Pen Injector [Ozempic] |
| NDC | 00169413290 | 0·25 MG, 0·5 MG Dose 1·5 ML semaglutide 1·34 MG/ML Pen Injector [Ozempic] |
| NDC | 00169413297 | 0·25 MG, 0·5 MG Dose 1·5 ML semaglutide 1·34 MG/ML Pen Injector [Ozempic] |
| NDC | 001694136 | semaglutide 1·34mg/mL SUBCUTANEOUS INJECTION, SOLUTION |
| NDC | 00169413602 | 1 MG Dose 1·5 ML semaglutide 1·34 MG/ML Pen Injector [Ozempic] |
| NDC | 00169413611 | 1 MG Dose 1·5 ML semaglutide 1·34 MG/ML Pen Injector [Ozempic] |
| NDC | 001694772 | semaglutide 2·68mg/mL SUBCUTANEOUS INJECTION, SOLUTION |
| NDC | 00169477211 | 3 ML semaglutide 2·68 MG/ML Pen Injector [Ozempic] |
| NDC | 00169477212 | 3 ML semaglutide 2·68 MG/ML Pen Injector [Ozempic] |
| NDC | 00169477290 | 3 ML semaglutide 2·68 MG/ML Pen Injector [Ozempic] |
| NDC | 00169477297 | 3 ML semaglutide 2·68 MG/ML Pen Injector [Ozempic] |
| NDC | 01694181 | semaglutide ·68mg/mL SUBCUTANEOUS INJECTION, SOLUTION |
| NDC | 0169418103 | 3 ML semaglutide 0·68 MG/ML Pen Injector [Ozempic] |
| NDC | 0169418113 | 3 ML semaglutide 0·68 MG/ML Pen Injector [Ozempic] |
| NDC | 0169418190 | 3 ML semaglutide 0·68 MG/ML Pen Injector [Ozempic] |
| NDC | 0169418197 | 3 ML semaglutide 0·68 MG/ML Pen Injector [Ozempic] |
| NDC | 500906051 | semaglutide 2·68mg/mL SUBCUTANEOUS INJECTION, SOLUTION |
| NDC | 5009060510 | 3 ML semaglutide 2·68 MG/ML Pen Injector [Ozempic] |
| RxNorm | 1991307 | Ozempic |
| RxNorm | 1991308 | semaglutide 1·34 MG/ML [Ozempic] |
| RxNorm | 1991309 | semaglutide Pen Injector [Ozempic] |
| RxNorm | 1991310 | Ozempic Injectable Product |
| RxNorm | 1991311 | 0·25 MG, 0·5 MG Dose 1·5 ML semaglutide 1·34 MG/ML Pen Injector [Ozempic] |
| RxNorm | 1991317 | 1 MG Dose 1·5 ML semaglutide 1·34 MG/ML Pen Injector [Ozempic] |
| RxNorm | 2398842 | 3 ML semaglutide 1·34 MG/ML Pen Injector [Ozempic] |
| RxNorm | 2398844 | semaglutide 1·34 MG/ML Pen Injector [Ozempic] |
| RxNorm | 2599364 | semaglutide 2·68 MG/ML [Ozempic] |
| RxNorm | 2599365 | 3 ML semaglutide 2·68 MG/ML Pen Injector [Ozempic] |
| RxNorm | 2599366 | semaglutide 2·68 MG/ML Pen Injector [Ozempic] |
| RxNorm | 2619153 | semaglutide 0·68 MG/ML [Ozempic] |
| RxNorm | 2619154 | 0·25 MG, 0·5 MG Dose 3 ML semaglutide 0·68 MG/ML Pen Injector [Ozempic] |
| NDC | 500905138 | semaglutide 1·34mg/mL SUBCUTANEOUS INJECTION, SOLUTION |
| NDC | 50090513800 | 0·25 MG, 0·5 MG Dose 1·5 ML semaglutide 1·34 MG/ML Pen Injector [Ozempic] |
| NDC | 500905139 | semaglutide 1·34mg/mL SUBCUTANEOUS INJECTION, SOLUTION |
| NDC | 50090513900 | 1 MG Dose 1·5 ML semaglutide 1·34 MG/ML Pen Injector [Ozempic] |
| NDC | 500905824 | semaglutide ·25mg/·5mL SUBCUTANEOUS INJECTION, SOLUTION |
| NDC | 500905949 | semaglutide 1·34mg/mL SUBCUTANEOUS INJECTION, SOLUTION |
| NDC | 50090594900 | 3 ML semaglutide 1·34 MG/ML Pen Injector [Ozempic] |
| NDC | 705182143 | semaglutide 1·34mg/mL SUBCUTANEOUS INJECTION, SOLUTION |
| NDC | 70518214300 | 0·25 MG, 0·5 MG Dose 1·5 ML semaglutide 1·34 MG/ML Pen Injector [Ozempic] |
| *Semaglutide (Rybelsus)* | | |
| NDC | 001694303 | oral semaglutide 3mg/1 ORAL TABLET |
| NDC | 00169430301 | semaglutide 3 MG Oral Tablet [Rybelsus] |
| NDC | 00169430313 | semaglutide 3 MG Oral Tablet [Rybelsus] |
| NDC | 00169430330 | semaglutide 3 MG Oral Tablet [Rybelsus] |
| NDC | 00169430390 | semaglutide 3 MG Oral Tablet [Rybelsus] |
| NDC | 00169430393 | semaglutide 3 MG Oral Tablet [Rybelsus] |
| NDC | 00169430399 | semaglutide 3 MG Oral Tablet [Rybelsus] |
| NDC | 001694307 | oral semaglutide 7mg/1 ORAL TABLET |
| NDC | 00169430701 | semaglutide 7 MG Oral Tablet [Rybelsus] |
| NDC | 00169430713 | semaglutide 7 MG Oral Tablet [Rybelsus] |
| NDC | 00169430730 | semaglutide 7 MG Oral Tablet [Rybelsus] |
| NDC | 001694314 | oral semaglutide 14mg/1 ORAL TABLET |
| NDC | 00169431401 | semaglutide 14 MG Oral Tablet [Rybelsus] |
| NDC | 00169431413 | semaglutide 14 MG Oral Tablet [Rybelsus] |
| NDC | 00169431430 | semaglutide 14 MG Oral Tablet [Rybelsus] |
| RxNorm | 2200645 | Rybelsus |
| RxNorm | 2200646 | semaglutide 14 MG [Rybelsus] |
| RxNorm | 2200647 | semaglutide Oral Tablet [Rybelsus] |
| RxNorm | 2200648 | Rybelsus Oral Product |
| RxNorm | 2200649 | Rybelsus Pill |
| RxNorm | 2200650 | semaglutide 14 MG Oral Tablet [Rybelsus] |
| RxNorm | 2200653 | semaglutide 3 MG [Rybelsus] |
| RxNorm | 2200654 | semaglutide 3 MG Oral Tablet [Rybelsus] |
| RxNorm | 2200657 | semaglutide 7 MG [Rybelsus] |
| RxNorm | 2200658 | semaglutide 7 MG Oral Tablet [Rybelsus] |
| SNOMED CT | 1003647000 | Semaglutide 3 mg oral tablet |
| SNOMED CT | 1003648005 | Semaglutide 7 mg oral tablet |
| SNOMED CT | 1003649002 | Semaglutide 14 mg oral tablet |
| RxNorm | 2200640 | semaglutide 14 MG |
| RxNorm | 2200641 | semaglutide Oral Product |
| RxNorm | 2200642 | semaglutide Pill |
| RxNorm | 2200643 | semaglutide Oral Tablet |
| RxNorm | 2200644 | semaglutide 14 MG Oral Tablet |
| RxNorm | 2200651 | semaglutide 3 MG |
| RxNorm | 2200652 | semaglutide 3 MG Oral Tablet |
| RxNorm | 2200655 | semaglutide 7 MG |
| RxNorm | 2200656 | semaglutide 7 MG Oral Tablet |
| SNOMED CT | 764285005 | Semaglutide-containing product in oral dose form |
| SNOMED CT | 780439007 | Semaglutide only product in oral dose form |
| *Semaglutide (non-specific)* | | |
| RxNorm | 1991302 | semaglutide |
| RxNorm | 1991303 | semaglutide 1·34 MG/ML |
| RxNorm | 1991304 | semaglutide Injectable Product |
| RxNorm | 1991305 | semaglutide Pen Injector |
| RxNorm | 1991306 | 0·25 MG, 0·5 MG Dose 1·5 ML semaglutide 1·34 MG/ML Pen Injector |
| RxNorm | 1991316 | 1 MG Dose 1·5 ML semaglutide 1·34 MG/ML Pen Injector |
| RxNorm | 2398841 | 3 ML semaglutide 1·34 MG/ML Pen Injector |
| RxNorm | 2398843 | semaglutide 1·34 MG/ML Pen Injector |
| RxNorm | 2599361 | semaglutide 2·68 MG/ML |
| RxNorm | 2599362 | 3 ML semaglutide 2·68 MG/ML Pen Injector |
| RxNorm | 2599363 | semaglutide 2·68 MG/ML Pen Injector |
| RxNorm | 2619151 | semaglutide 0·68 MG/ML |
| RxNorm | 2619152 | 0·25 MG, 0·5 MG Dose 3 ML semaglutide 0·68 MG/ML Pen Injector |
| SNOMED CT | 764283003 | Semaglutide |
| SNOMED CT | 764284009 | Semaglutide-containing product |
| SNOMED CT | 770768004 | Semaglutide-containing product in parenteral dose form |
| SNOMED CT | 777514008 | Semaglutide only product |
| SNOMED CT | 780440009 | Semaglutide only product in parenteral dose form |
| SNOMED CT | 782102009 | Semaglutide 1·34 mg/mL solution for injection |
| *Tirzepatide (Mounjaro)* | | |
| RxNorm | 2601745 | tirzepatide 10 MG/ML [Mounjaro] |
| RxNorm | 2601747 | tirzepatide 10 MG/ML Auto-Injector [Mounjaro] |
| RxNorm | 2601781 | tirzepatide 15 MG/ML [Mounjaro] |
| RxNorm | 2601783 | tirzepatide 15 MG/ML Auto-Injector [Mounjaro] |
| RxNorm | 2601759 | tirzepatide 30 MG/ML Auto-Injector [Mounjaro] |
| RxNorm | 2601763 | tirzepatide 5 MG/ML [Mounjaro] |
| RxNorm | 2601765 | tirzepatide 5 MG/ML Auto-Injector [Mounjaro] |
| RxNorm | 2601737 | tirzepatide Auto-Injector [Mounjaro] |
| RxNorm | 2601746 | 0·5 ML tirzepatide 10 MG/ML Auto-Injector [Mounjaro] |
| RxNorm | 2601785 | 0·5 ML tirzepatide 15 MG/ML Auto-Injector [Mounjaro] |
| RxNorm | 2601770 | 0·5 ML tirzepatide 20 MG/ML Auto-Injector [Mounjaro] |
| RxNorm | 2601764 | 0·5 ML tirzepatide 5 MG/ML Auto-Injector [Mounjaro] |
| RxNorm | 2644403 | 0·5 ML tirzepatide 10 MG/ML Injection [Mounjaro] |
| RxNorm | 2644399 | 0·5 ML tirzepatide 15 MG/ML Injection [Mounjaro] |
| RxNorm | 2644419 | 0·5 ML tirzepatide 20 MG/ML Injection [Mounjaro] |
| RxNorm | 2644415 | 0·5 ML tirzepatide 25 MG/ML Injection [Mounjaro] |
| RxNorm | 2644411 | 0·5 ML tirzepatide 30 MG/ML Injection [Mounjaro] |
| RxNorm | 2644407 | 0·5 ML tirzepatide 5 MG/ML Injection [Mounjaro] |
| RxNorm | 2601769 | tirzepatide 20 MG/ML [Mounjaro] |
| RxNorm | 2601757 | tirzepatide 30 MG/ML [Mounjaro] |
| RxNorm | 2644404 | tirzepatide 10 MG/ML Injection [Mounjaro] |
| RxNorm | 2644400 | tirzepatide 15 MG/ML Injection [Mounjaro] |
| RxNorm | 2644420 | tirzepatide 20 MG/ML Injection [Mounjaro] |
| RxNorm | 2644416 | tirzepatide 25 MG/ML Injection [Mounjaro] |
| RxNorm | 2644412 | tirzepatide 30 MG/ML Injection [Mounjaro] |
| RxNorm | 2644408 | tirzepatide 5 MG/ML Injection [Mounjaro] |
| RxNorm | 2644398 | tirzepatide Injection [Mounjaro] |
| RxNorm | 2601776 | 0·5 ML tirzepatide 25 MG/ML Auto-Injector [Mounjaro] |
| RxNorm | 2601771 | tirzepatide 20 MG/ML Auto-Injector [Mounjaro] |
| RxNorm | 2601775 | tirzepatide 25 MG/ML [Mounjaro] |
| RxNorm | 2601777 | tirzepatide 25 MG/ML Auto-Injector [Mounjaro] |
| RxNorm | 2601758 | 0·5 ML tirzepatide 30 MG/ML Auto-Injector [Mounjaro] |
| RxNorm | 2601734 | Mounjaro |
| NDC | 00002147180 | 0·5 ML tirzepatide 20 MG/ML Auto-Injector [Mounjaro] |
| NDC | 00002146080 | 0·5 ML tirzepatide 25 MG/ML Auto-Injector [Mounjaro] |
| NDC | 00002150680 | 0·5 ML tirzepatide 5 MG/ML Auto-Injector [Mounjaro] |
| NDC | 00002145780 | 0·5 ML tirzepatide 30 MG/ML Auto-Injector [Mounjaro] |
| NDC | 00002146001 | 0·5 ML tirzepatide 25 MG/ML Auto-Injector [Mounjaro] |
| NDC | 00002149580 | 0·5 ML tirzepatide 10 MG/ML Auto-Injector [Mounjaro] |
| NDC | 00002148480 | 0·5 ML tirzepatide 15 MG/ML Auto-Injector [Mounjaro] |
| NDC | 00002150601 | 0·5 ML tirzepatide 5 MG/ML Auto-Injector [Mounjaro] |
| NDC | 00002145701 | 0·5 ML tirzepatide 30 MG/ML Auto-Injector [Mounjaro] |
| NDC | 00002147101 | 0·5 ML tirzepatide 20 MG/ML Auto-Injector [Mounjaro] |
| NDC | 00002148401 | 0·5 ML tirzepatide 15 MG/ML Auto-Injector [Mounjaro] |
| NDC | 00002149501 | 0·5 ML tirzepatide 10 MG/ML Auto-Injector [Mounjaro] |
| NDC | 00002115201 | 0·5 ML tirzepatide 5 MG/ML Auto-Injector [Mounjaro] |
| NDC | 00002124301 | 0·5 ML tirzepatide 10 MG/ML Auto-Injector [Mounjaro] |
| NDC | 00002221401 | 0·5 ML tirzepatide 15 MG/ML Auto-Injector [Mounjaro] |
| NDC | 00002234001 | 0·5 ML tirzepatide 20 MG/ML Auto-Injector [Mounjaro] |
| NDC | 00002242301 | 0·5 ML tirzepatide 25 MG/ML Auto-Injector [Mounjaro] |
| NDC | 00002300201 | 0·5 ML tirzepatide 30 MG/ML Auto-Injector [Mounjaro] |
| *Tirzepatide (non-specific)* | | |
| RxNorm | 2601723 | tirzepatide |
| RxNorm | 2601742 | tirzepatide 10 MG/ML |
| RxNorm | 2601744 | tirzepatide 10 MG/ML Auto-Injector |
| RxNorm | 2601778 | tirzepatide 15 MG/ML |
| RxNorm | 2601780 | tirzepatide 15 MG/ML Auto-Injector |
| RxNorm | 2601736 | Mounjaro Injectable Product |
| RxNorm | 2601760 | tirzepatide 5 MG/ML |
| RxNorm | 2601762 | tirzepatide 5 MG/ML Auto-Injector |
| RxNorm | 2601731 | tirzepatide Auto-Injector |
| RxNorm | 2601730 | tirzepatide Injectable Product |
| RxNorm | 2601743 | 0·5 ML tirzepatide 10 MG/ML Auto-Injector |
| RxNorm | 2601784 | 0·5 ML tirzepatide 15 MG/ML Auto-Injector |
| RxNorm | 2601767 | 0·5 ML tirzepatide 20 MG/ML Auto-Injector |
| RxNorm | 2601773 | 0·5 ML tirzepatide 25 MG/ML Auto-Injector |
| RxNorm | 2601761 | 0·5 ML tirzepatide 5 MG/ML Auto-Injector |
| RxNorm | 2601755 | 0·5 ML tirzepatide 30 MG/ML Auto-Injector |
| RxNorm | 2644401 | 0·5 ML tirzepatide 10 MG/ML Injection |
| RxNorm | 2644396 | 0·5 ML tirzepatide 15 MG/ML Injection |
| RxNorm | 2644417 | 0·5 ML tirzepatide 20 MG/ML Injection |
| RxNorm | 2644413 | 0·5 ML tirzepatide 25 MG/ML Injection |
| RxNorm | 2644409 | 0·5 ML tirzepatide 30 MG/ML Injection |
| RxNorm | 2644405 | 0·5 ML tirzepatide 5 MG/ML Injection |
| RxNorm | 2644402 | tirzepatide 10 MG/ML Injection |
| RxNorm | 2644397 | tirzepatide 15 MG/ML Injection |
| RxNorm | 2644418 | tirzepatide 20 MG/ML Injection |
| RxNorm | 2644414 | tirzepatide 25 MG/ML Injection |
| RxNorm | 2644410 | tirzepatide 30 MG/ML Injection |
| RxNorm | 2644406 | tirzepatide 5 MG/ML Injection |
| RxNorm | 2644395 | tirzepatide Injection |
| RxNorm | 2601756 | tirzepatide 30 MG/ML Auto-Injector |
| RxNorm | 2601772 | tirzepatide 25 MG/ML |
| RxNorm | 2601774 | tirzepatide 25 MG/ML Auto-Injector |
| RxNorm | 2601768 | tirzepatide 20 MG/ML Auto-Injector |
| RxNorm | 2601754 | tirzepatide 30 MG/ML |
| RxNorm | 2601766 | tirzepatide 20 MG/ML |
| NDC | 00002150661 | tirzepatide 2·5mg/·5mL SUBCUTANEOUS INJECTION, SOLUTION |
| NDC | 000021471 | tirzepatide 10mg/·5mL SUBCUTANEOUS INJECTION, SOLUTION |
| NDC | 000021460 | tirzepatide 12·5mg/·5mL SUBCUTANEOUS INJECTION, SOLUTION |
| NDC | 000021484 | tirzepatide 7·5mg/·5mL SUBCUTANEOUS INJECTION, SOLUTION |
| NDC | 000021506 | tirzepatide 2·5mg/·5mL SUBCUTANEOUS INJECTION, SOLUTION |
| NDC | 000021457 | tirzepatide 15mg/·5mL SUBCUTANEOUS INJECTION, SOLUTION |
| NDC | 000021495 | tirzepatide 5mg/·5mL SUBCUTANEOUS INJECTION, SOLUTION |
|  |  |  |
| *Other anti-diabetes medications (e.g., metforimin, insulin, sulfonylurea, SGLT2 inhibitors, DPP-4)* | | |
| [Please see pages 75-140 of the following citation:  https://www.medrxiv.org/content/medrxiv/early/2023/11/22/2023.11.21.23298775/DC2/embed/media-2.pdf](https://www.medrxiv.org/content/medrxiv/early/2023/11/22/2023.11.21.23298775/DC2/embed/media-2.pdf) | | |
|  |  |  |
| *HbA1c laboratory assessments* | | |
| LOINC | 4548-4 | Haemoglobin A1c/Haemoglobin.total in Blood |
| LOINC | 4549-2 | Haemoglobin A1c/Haemoglobin.total in Blood by Electrophoresis |
| LOINC | 17856-6 | Haemoglobin A1c/Haemoglobin.total in Blood by HPLC |
| LOINC | 62388-4 | Haemoglobin A1c/Haemoglobin.total in Blood by JDS/JSCC protocol |
| LOINC | 71875-9 | Haemoglobin A1c/Haemoglobin.total [Pure mass fraction] in Blood |
| LOINC | 59261-8 | Haemoglobin A1c/Haemoglobin.total in Blood by IFCC protocol |
| LOINC | 96595-4 | Haemoglobin A1c/Haemoglobin.total in DBS |

Abbreviations: ICD-10-CM = International Classification of Diseases, 10^th^ edition, Clinical Modification; LOINC = Logical Observation Identifiers Names and Codes; NDC = National Drug Code; SNOMED CT = Systematized Nomenclature of Medicine – Clinical Terms.

### Supplemental Table 4. Diagnosis, procedure, and drug codes used for identifying unintentional weight loss

| **Code System** | **Code** | **Concept Name** |
| --- | --- | --- |
| *Abnormal weight gain or loss or unintentional weight loss* | | | |
| ICD-10-CM | R63.4 | Abnormal weight loss |
| ICD-10-CM | R63.5 | Abnormal weight gain |
|  |  |  |
| *Acute or chronic pancreatitis* | | | |
| ICD-10-CM | K8500 | Idiopathic acute pancreatitis without necrosis or infection |
| ICD-10-CM | K8501 | Idiopathic acute pancreatitis with uninfected necrosis |
| ICD-10-CM | K8502 | Idiopathic acute pancreatitis with infected necrosis |
| ICD-10-CM | K8510 | Biliary acute pancreatitis without necrosis or infection |
| ICD-10-CM | K8511 | Biliary acute pancreatitis with uninfected necrosis |
| ICD-10-CM | K8512 | Biliary acute pancreatitis with infected necrosis |
| ICD-10-CM | K8520 | Alcohol induced acute pancreatitis without necrosis or infection |
| ICD-10-CM | K8521 | Alcohol induced acute pancreatitis with uninfected necrosis |
| ICD-10-CM | K8522 | Alcohol induced acute pancreatitis with infected necrosis |
| ICD-10-CM | K8530 | Drug induced acute pancreatitis without necrosis or infection |
| ICD-10-CM | K8531 | Drug induced acute pancreatitis with uninfected necrosis |
| ICD-10-CM | K8532 | Drug induced acute pancreatitis with infected necrosis |
| ICD-10-CM | K8580 | Other acute pancreatitis without necrosis or infection |
| ICD-10-CM | K8581 | Other acute pancreatitis with uninfected necrosis |
| ICD-10-CM | K8582 | Other acute pancreatitis with infected necrosis |
| ICD-10-CM | K8590 | Acute pancreatitis without necrosis or infection, unspecified |
| ICD-10-CM | K8591 | Acute pancreatitis with uninfected necrosis, unspecified |
| ICD-10-CM | K8592 | Acute pancreatitis with infected necrosis, unspecified |
| ICD-10-CM | K860 | Alcohol-induced chronic pancreatitis |
| ICD-10-CM | K861 | Other chronic pancreatitis |
| ICD-10-CM | K86.81 | Exocrine pancreatic insufficiency |
| ICD-10-CM | K86.89 | Other specified diseases of pancreas |
| ICD-10-CM | K87 | Disorders of gallbladder, biliary tract and pancreas in diseases classified elsewhere |
| CPT | 48000 | Placement of drains, peripancreatic, for acute pancreatitis |
| CPT | 48001 | Placement of drains, peripancreatic, for acute pancreatitis; with cholecystostomy, gastrostomy, and jejunostomy |
| CPT | 48105 | Resection or debridement of pancreas and peripancreatic tissue for acute necrotizing pancreatitis |
|  |  |  |
| *Alcohol use disorder* | | | |
| ICD-10-CM | F10 | Alcohol related disorders |
| ICD-10-CM | G62.1 | Alcoholic polyneuropathy |
| ICD-10-CM | I42.6 | Alcoholic cardiomyopathy |
| ICD-10-CM | K29.2 | Alcoholic gastritis |
| ICD-10-CM | K70.0 | Alcoholic fatty liver |
| ICD-10-CM | K70.3 | Alcoholic cirrhosis of liver |
| ICD-10-CM | K70.9 | Alcoholic liver disease, unspecified |
| ICD-10-CM | T51 | Toxic effect of alcohol |
| ICD-10-CM | Z50.2 | Alcohol rehabilitation |
| ICD-10-CM | Z71.4 | Alcohol abuse counseling and surveillance |
| ICD-10-CM | Z72.1 | Alcohol use |
| ICD-10-CM | G31.2 | Degeneration of nervous system due to alcohol |
| ICD-10-CM | P04.3 | Newborn affected by maternal use of alcohol |
| ICD-10-CM | Q86.0 | Fetal alcohol syndrome (dysmorphic) |
|  |  |  |
| *Anorexia nervosa* | | | |
| ICD-10-CM | F50.00 | Anorexia nervosa, unspecified |
| ICD-10-CM | F50.01 | Anorexia nervosa, restricting type |
| ICD-10-CM | F50.02 | Anorexia nervosa, binge eating/purging type |
| ICD-10-CM | R63.0 | Anorexia |
|  |  |  |
| *Cachexia* |  |  |
| ICD-10-CM | R64 | Cachexia |
|  |  |  |
| *Celiac disease* |  |  |
| ICD-10-CM | K90.0 | Celiac disease |
| SNOMED CT | 396331005 | Celiac disease |
| SNOMED CT | 61715008 | Celiac disease with diffuse intestinal ulceration |
| SNOMED CT | 91867008 | Adult form of celiac disease |
| SNOMED CT | 396330006 | Celiac crisis |
| SNOMED CT | 722386009 | Celiac disease with epilepsy and cerebral calcification syndrome |
| SNOMED CT | 770593004 | Refractory celiac disease |
| SNOMED CT | 1148861009 | Iron deficiency anemia due to celiac disease |
| SNOMED CT | 57850000 | Celiac rickets |
| SNOMED CT | 1197676001 | Duodenitis due to celiac disease |
| SNOMED CT | 197478000 | Congenital celiac disease |
| SNOMED CT | 1197691004 | Duodenal ulcer due to celiac disease |
| SNOMED CT | 1259581001 | Dementia due to celiac disease |
| SNOMED CT | 6659005 | Megaloblastic anemia due to celiac disease |
| SNOMED CT | 1196966007 | Gingival disease due to celiac disease |
| SNOMED CT | 197479008 | Acquired celiac disease |
| SNOMED CT | 197481005 | Celiac disease NOS |
| SNOMED CT | 197477005 | (Coeliac: [disease] or [rickets]) or (Gee-Herter disease) |
|  |  |  |
| *Cognitive impairment, including dementia* | | | |
| ICD-10-CM | A8100 | Creutzfeldt-Jakob disease, unspecified |
| ICD-10-CM | A8101 | Variant Creutzfeldt-Jakob disease |
| ICD-10-CM | A8109 | Other Creutzfeldt-Jakob disease |
| ICD-10-CM | E7502 | Tay-Sachs disease |
| ICD-10-CM | E7519 | Other gangliosidosis |
| ICD-10-CM | E7523 | Krabbe disease |
| ICD-10-CM | E7525 | Metachromatic leukodystrophy |
| ICD-10-CM | E7529 | Other sphingolipidosis |
| ICD-10-CM | E754 | Neuronal ceroid lipofuscinosis |
| ICD-10-CM | F0150 | Vascular dementia without behavioral disturbance |
| ICD-10-CM | F0151 | Vascular dementia with behavioral disturbance |
| ICD-10-CM | F0280 | Dementia in other diseases classified elsewhere without behavioral disturbance |
| ICD-10-CM | F0281 | Dementia in other diseases classified elsewhere with behavioral disturbance |
| ICD-10-CM | F0390 | Unspecified dementia without behavioral disturbance |
| ICD-10-CM | F0391 | Unspecified dementia with behavioral disturbance |
| ICD-10-CM | F04 | Amnestic disorder due to known physiological condition |
| ICD-10-CM | F1026 | Alcohol dependence with alcohol-induced persisting amnestic disorder |
| ICD-10-CM | F1027 | Alcohol dependence with alcohol-induced persisting dementia |
| ICD-10-CM | F1096 | Alcohol use, unspecified with alcohol-induced persisting amnestic disorder |
| ICD-10-CM | F1097 | Alcohol use, unspecified with alcohol-induced persisting dementia |
| ICD-10-CM | F1326 | Sedative, hypnotic or anxiolytic dependence with sedative, hypnotic or anxiolytic-induced persisting amnestic disorder |
| ICD-10-CM | F1327 | Sedative, hypnotic or anxiolytic dependence with sedative, hypnotic or anxiolytic-induced persisting dementia |
| ICD-10-CM | F1396 | Sedative, hypnotic or anxiolytic use, unspecified with sedative, hypnotic or anxiolytic-induced persisting amnestic disorder |
| ICD-10-CM | F1397 | Sedative, hypnotic or anxiolytic use, unspecified with sedative, hypnotic or anxiolytic-induced persisting dementia |
| ICD-10-CM | F1817 | Inhalant abuse with inhalant-induced dementia |
| ICD-10-CM | F1827 | Inhalant dependence with inhalant-induced dementia |
| ICD-10-CM | F1897 | Inhalant use, unspecified with inhalant-induced persisting dementia |
| ICD-10-CM | F1916 | Other psychoactive substance abuse with psychoactive substance-induced persisting amnestic disorder |
| ICD-10-CM | F1917 | Other psychoactive substance abuse with psychoactive substance-induced persisting dementia |
| ICD-10-CM | F1926 | Other psychoactive substance dependence with psychoactive substance-induced persisting amnestic disorder |
| ICD-10-CM | F1927 | Other psychoactive substance dependence with psychoactive substance-induced persisting dementia |
| ICD-10-CM | F1996 | Other psychoactive substance use, unspecified with psychoactive substance-induced persisting amnestic disorder |
| ICD-10-CM | F1997 | Other psychoactive substance use, unspecified with psychoactive substance-induced persisting dementia |
| ICD-10-CM | F70 | Mild intellectual disabilities |
| ICD-10-CM | F71 | Moderate intellectual disabilities |
| ICD-10-CM | F72 | Severe intellectual disabilities |
| ICD-10-CM | F73 | Profound intellectual disabilities |
| ICD-10-CM | F78 | Other intellectual disabilities |
| ICD-10-CM | F79 | Unspecified intellectual disabilities |
| ICD-10-CM | F842 | Rett's syndrome |
| ICD-10-CM | G10 | Huntington's disease |
| ICD-10-CM | G132 | Systemic atrophy primarily affecting the central nervous system in myxedema |
| ICD-10-CM | G138 | Systemic atrophy primarily affecting central nervous system in other diseases classified elsewhere |
| ICD-10-CM | G231 | Progressive supranuclear ophthalmoplegia [Steele-Richardson-Olszewski] |
| ICD-10-CM | G300 | Alzheimer's disease with early onset |
| ICD-10-CM | G301 | Alzheimer's disease with late onset |
| ICD-10-CM | G308 | Other Alzheimer's disease |
| ICD-10-CM | G309 | Alzheimer's disease, unspecified |
| ICD-10-CM | G3101 | Pick's disease |
| ICD-10-CM | G3109 | Other frontotemporal dementia |
| ICD-10-CM | G311 | Senile degeneration of brain, not elsewhere classified |
| ICD-10-CM | G312 | Degeneration of nervous system due to alcohol |
| ICD-10-CM | G3181 | Alpers disease |
| ICD-10-CM | G3182 | Leigh's disease |
| ICD-10-CM | G3183 | Dementia with Lewy bodies |
| ICD-10-CM | G3184 | Mild cognitive impairment, so stated |
| ICD-10-CM | G3185 | Corticobasal degeneration |
| ICD-10-CM | G3189 | Other specified degenerative diseases of nervous system |
| ICD-10-CM | G319 | Degenerative disease of nervous system, unspecified |
| ICD-10-CM | G910 | Communicating hydrocephalus |
| ICD-10-CM | G911 | Obstructive hydrocephalus |
| ICD-10-CM | G912 | (Idiopathic) normal pressure hydrocephalus |
| ICD-10-CM | G937 | Reye's syndrome |
| ICD-10-CM | I6901 | Cognitive deficits following nontraumatic subarachnoid hemorrhage |
| ICD-10-CM | I69015 | Cognitive social or emotional deficit following nontraumatic subarachnoid hemorrhage |
| ICD-10-CM | I69018 | Other symptoms and signs involving cognitive functions following nontraumatic subarachnoid hemorrhage |
| ICD-10-CM | I69019 | Unspecified symptoms and signs involving cognitive functions following nontraumatic subarachnoid hemorrhage |
| ICD-10-CM | I6911 | Cognitive deficits following nontraumatic intracerebral hemorrhage |
| ICD-10-CM | I69115 | Cognitive social or emotional deficit following nontraumatic intracerebral hemorrhage |
| ICD-10-CM | I69118 | Other symptoms and signs involving cognitive functions following nontraumatic intracerebral hemorrhage |
| ICD-10-CM | I69119 | Unspecified symptoms and signs involving cognitive functions following nontraumatic intracerebral hemorrhage |
| ICD-10-CM | I6921 | Cognitive deficits following other nontraumatic intracranial hemorrhage |
| ICD-10-CM | I69215 | Cognitive social or emotional deficit following other nontraumatic intracranial hemorrhage |
| ICD-10-CM | I69218 | Other symptoms and signs involving cognitive functions following other nontraumatic intracranial hemorrhage |
| ICD-10-CM | I69219 | Unspecified symptoms and signs involving cognitive functions following other nontraumatic intracranial hemorrhage |
| ICD-10-CM | I6931 | Cognitive deficits following cerebral infarction |
| ICD-10-CM | I69315 | Cognitive social or emotional deficit following cerebral infarction |
| ICD-10-CM | I69318 | Other symptoms and signs involving cognitive functions following cerebral infarction |
| ICD-10-CM | I69319 | Unspecified symptoms and signs involving cognitive functions following cerebral infarction |
| ICD-10-CM | I6981 | Cognitive deficits following other cerebrovascular disease |
| ICD-10-CM | I69815 | Cognitive social or emotional deficit following other cerebrovascular disease |
| ICD-10-CM | I69818 | Other symptoms and signs involving cognitive functions following other cerebrovascular disease |
| ICD-10-CM | I69819 | Unspecified symptoms and signs involving cognitive functions following other cerebrovascular disease |
| ICD-10-CM | I6991 | Cognitive deficits following unspecified cerebrovascular disease |
| ICD-10-CM | I69915 | Cognitive social or emotional deficit following unspecified cerebrovascular disease |
| ICD-10-CM | I69918 | Other symptoms and signs involving cognitive functions following unspecified cerebrovascular disease |
| ICD-10-CM | I69919 | Unspecified symptoms and signs involving cognitive functions following unspecified cerebrovascular disease |
| ICD-10-CM | R411 | Anterograde amnesia |
| ICD-10-CM | R412 | Retrograde amnesia |
| ICD-10-CM | R413 | Other amnesia |
| ICD-10-CM | R4181 | Age-related cognitive decline |
| ICD-10-CM | R4183 | Borderline intellectual functioning |
| ICD-10-CM | R41841 | Cognitive communication deficit |
| ICD-10-CM | R4189 | Other symptoms and signs involving cognitive functions and awareness |
| ICD-10-CM | R419 | Unspecified symptoms and signs involving cognitive functions and awareness |
| ICD-10-CM | F00 | Dementia in Alzheimer disease |
| ICD-10-CM | F01 | Vascular dementia |
| ICD-10-CM | F02 | Dementia in other diseases classified elsewhere |
| ICD-10-CM | F03 | Unspecified dementia |
|  |  |  |
| *Cushing syndrome* | | | |
| ICD-10-CM | E240 | Pituitary-dependent Cushing's disease |
| ICD-10-CM | E241 | Nelson's syndrome |
| ICD-10-CM | E242 | Drug-induced Cushing's syndrome |
|  |  |  |
| *End-stage renal disease (ESRD)* | | | |
| ICD-10-CM | N185 | Chronic kidney disease, stage 5 |
| ICD-10-CM | N186 | End stage renal disease |
|  |  |  |
| *Feeding difficulties* | | | |
| ICD-10-CM | R633 | Feeding difficulties |
|  |  |  |
| *Genetic obesity/monogenic obesity/syndromic obesity* | | | |
| ICD-10-CM | Q78.1 | Polyostotic fibrous dysplasia (includes Albright syndrome) |
| ICD-10-CM | N25.89 | Other disorders resulting from impaired renal tubular function (includes Albright syndrome) |
| ICD-10-CM | Q87.1 | Congenital malform syndromes predom assoc w short stature (includes Prader Willi) |
| CPT | 5H | SNRPN (Prader Willi-Angelman syndrome) |
| CPT | 5E | GABRA5, NIPA1, UBE3A, or ANCR GABRA (Prader Willi-Angelman syndrome) |
| CPT | 81331 | SNRPN/UBE3A (small nuclear ribonucleoprotein polypeptide N and ubiquitin protein ligase E3A) (eg, Prader-Willi syndrome and/or Angelman syndrome), methylation analysis |
| ICD-10-CM | Q87.83 | Bardet-Biedl syndrome |
| SNOMED CT | 1187531009 | Obesity due to pituitary disease |
| SNOMED CT | 5036006 | Hypogonadal obesity |
| SNOMED CT | 1229946007 | MAGEL2-related Prader-Willi-like syndrome |
| SNOMED CT | 1208987006 | PHIP-related behavioral problems, intellectual disability, obesity, dysmorphic features syndrome |
| SNOMED CT | 1229943004 | SIM1-related Prader-Willi-like syndrome |
| SNOMED CT | 763350002 | Intellectual disability, obesity, brain malformation, facial dysmorphism syndrome |
| SNOMED CT | 770680004 | Prader-Willi-like syndrome |
| SNOMED CT | 770750002 | Intellectual disability, seizures, macrocephaly, obesity syndrome |
| SNOMED CT | 773663004 | Rapid-onset childhood obesity, hypothalamic dysfunction, hypoventilation, autonomic dysregulation syndrome |
| SNOMED CT | 774102003 | Intellectual disability, obesity, prognathism, eye and skin anomalies syndrome |
| SNOMED CT | 776204008 | Colobomatous microphthalmia, obesity, hypogenitalism, intellectual disability syndrome |
| SNOMED CT | 783549006 | Obesity due to CEP19 deficiency |
| SNOMED CT | 783556000 | Severe early-onset obesity insulin resistance syndrome due to SH2B1 deficiency |
| SNOMED CT | 783719006 | Obesity due to SIM1 deficiency |
| SNOMED CT | 785722006 | Obesity due to leptin receptor gene deficiency |
| SNOMED CT | 171000119107 | Maternal obesity complicating pregnancy, childbirth and the puerperium, antepartum |
| SNOMED CT | 1076701000119104 | Hypertrophy of fat pad of right knee |
| SNOMED CT | 1076711000119101 | Hypertrophy of fat pad of left knee |
| SNOMED CT | 10750551000119100 | Obesity in mother complicating childbirth |
| SNOMED CT | 15750121000119108 | Severe obesity complicating pregnancy |
| SNOMED CT | 722037004 | Intellectual disability, epileptic seizures, hypogonadism and hypogenitalism, microcephaly, obesity syndrome |
| SNOMED CT | 238132000 | Android obesity |
| SNOMED CT | 238133005 | Gynecoid obesity |
| SNOMED CT | 238135003 | Fat pad syndrome |
| SNOMED CT | 360566006 | Buffalo obesity |
| SNOMED CT | 414438005 | Hyperplastic obesity |
| SNOMED CT | 53146006 | Hypothyroid obesity |
| SNOMED CT | 57337005 | Steatopygia |
| SNOMED CT | 62999006 | Adiposogenital dystrophy |
| SNOMED CT | 63702009 | Alstrom syndrome |
| SNOMED CT | 72894001 | Hypertrophy of fat pad of knee |
| SNOMED CT | 80660001 | Mauriac’s syndrome |
| SNOMED CT | 700150001 | Congenital leptin deficiency |
| SNOMED CT | 702949005 | Proopiomelanocortin deficiency syndrome |
| SNOMED CT | 715628009 | MORM syndrome |
| SNOMED CT | 717269008 | Obesity due to melanocortin 4 receptor deficiency |
| SNOMED CT | 717761005 | Choroideremia with deafness and obesity syndrome |
| SNOMED CT | 719160009 | Syndromic X-linked intellectual disability type 7 |
| SNOMED CT | 719834005 | Wilson Turner syndrome |
| SNOMED CT | 721231007 | Hydrocephalus with obesity and hypogonadism syndrome |
| SNOMED CT | 722051004 | Obesity, colitis, hypothyroidism, cardiac hypertrophy, developmental delay syndrome |
| SNOMED CT | 722053001 | Obesity due to prohormone convertase I deficiency |
| SNOMED CT | 724137002 | MOMO syndrome |
| SNOMED CT | 290439001 | Familial obesity |
| SNOMED CT | 293481008 | Hyperplastic-hypertrophic obesity |
| SNOMED CT | 295509007 | Hypertrophic obesity |
| SNOMED CT | 298464002 | Obesity of endocrine origin |
| SNOMED CT | 1003380001 | 6q16 microdeletion syndrome |
| SNOMED CT | 1255335006 | X-linked intellectual disability, short stature, overweight syndrome |
| SNOMED CT | 82793005 | Hypothalamic obesity |
| SNOMED CT | 111036000 | Hyperinsulinar obesity |
| SNOMED CT | 1260134001 | Spastic paraplegia, intellectual disability, nystagmus, obesity syndrome |
| SNOMED CT | 1260139006 | Genetic non-syndromic obesity |
|  |  |  |
| *Human immunodeficiency virus (HIV)* | | | |
| ICD-10-CM | B20 | Human immunodeficiency virus [HIV] disease |
|  |  |  |
| *Hyperthyroidism* |  |  |
| ICD-10-CM | E01.0 | Iodine-deficiency related diffuse (endemic) goiter |
| ICD-10-CM | E01.1 | Iodine-deficiency related multinodular (endemic) goiter |
| ICD-10-CM | E01.2 | Iodine-deficiency related (endemic) goiter, unspecified |
| ICD-10-CM | E03.0 | Congenital hypothyroidism with diffuse goiter |
| ICD-10-CM | E07.1 | Dyshormogenetic goiter |
| ICD-10-CM | E0500 | Thyrotoxicosis with diffuse goiter without thyrotoxic crisis or storm |
| ICD-10-CM | E0501 | Thyrotoxicosis with diffuse goiter with thyrotoxic crisis or storm |
| ICD-10-CM | E0510 | Thyrotoxicosis with toxic single thyroid nodule without thyrotoxic crisis or storm |
| ICD-10-CM | E0511 | Thyrotoxicosis with toxic single thyroid nodule with thyrotoxic crisis or storm |
| ICD-10-CM | E0520 | Thyrotoxicosis with toxic multinodular goiter without thyrotoxic crisis or storm |
| ICD-10-CM | E0521 | Thyrotoxicosis with toxic multinodular goiter with thyrotoxic crisis or storm |
| ICD-10-CM | E0530 | Thyrotoxicosis from ectopic thyroid tissue without thyrotoxic crisis or storm |
| ICD-10-CM | E0531 | Thyrotoxicosis from ectopic thyroid tissue with thyrotoxic crisis or storm |
| ICD-10-CM | E0540 | Thyrotoxicosis factitia without thyrotoxic crisis or storm |
| ICD-10-CM | E0541 | Thyrotoxicosis factitia with thyrotoxic crisis or storm |
| ICD-10-CM | E0580 | Other thyrotoxicosis without thyrotoxic crisis or storm |
| ICD-10-CM | E0581 | Other thyrotoxicosis with thyrotoxic crisis or storm |
| ICD-10-CM | E0590 | Thyrotoxicosis, unspecified without thyrotoxic crisis or storm |
| ICD-10-CM | E0591 | Thyrotoxicosis, unspecified with thyrotoxic crisis or storm |
| ICD-10-CM | E062 | Chronic thyroiditis with transient thyrotoxicosis |
|  |  |  |
| *Inflammatory bowel disease (IBD)* | | | |
| ICD-10-CM | K5000 | Crohn's disease of small intestine without complications |
| ICD-10-CM | K50011 | Crohn's disease of small intestine with rectal bleeding |
| ICD-10-CM | K50012 | Crohn's disease of small intestine with intestinal obstruction |
| ICD-10-CM | K50013 | Crohn's disease of small intestine with fistula |
| ICD-10-CM | K50014 | Crohn's disease of small intestine with abscess |
| ICD-10-CM | K50018 | Crohn's disease of small intestine with other complication |
| ICD-10-CM | K50019 | Crohn's disease of small intestine with unspecified complications |
| ICD-10-CM | K5010 | Crohn's disease of large intestine without complications |
| ICD-10-CM | K50111 | Crohn's disease of large intestine with rectal bleeding |
| ICD-10-CM | K50112 | Crohn's disease of large intestine with intestinal obstruction |
| ICD-10-CM | K50113 | Crohn's disease of large intestine with fistula |
| ICD-10-CM | K50114 | Crohn's disease of large intestine with abscess |
| ICD-10-CM | K50118 | Crohn's disease of large intestine with other complication |
| ICD-10-CM | K50119 | Crohn's disease of large intestine with unspecified complications |
| ICD-10-CM | K5080 | Crohn's disease of both small and large intestine without complications |
| ICD-10-CM | K50811 | Crohn's disease of both small and large intestine with rectal bleeding |
| ICD-10-CM | K50812 | Crohn's disease of both small and large intestine with intestinal obstruction |
| ICD-10-CM | K50813 | Crohn's disease of both small and large intestine with fistula |
| ICD-10-CM | K50814 | Crohn's disease of both small and large intestine with abscess |
| ICD-10-CM | K50818 | Crohn's disease of both small and large intestine with other complication |
| ICD-10-CM | K50819 | Crohn's disease of both small and large intestine with unspecified complications |
| ICD-10-CM | K5090 | Crohn's disease, unspecified, without complications |
| ICD-10-CM | K50911 | Crohn's disease, unspecified, with rectal bleeding |
| ICD-10-CM | K50912 | Crohn's disease, unspecified, with intestinal obstruction |
| ICD-10-CM | K50913 | Crohn's disease, unspecified, with fistula |
| ICD-10-CM | K50914 | Crohn's disease, unspecified, with abscess |
| ICD-10-CM | K50918 | Crohn's disease, unspecified, with other complication |
| ICD-10-CM | K50919 | Crohn's disease, unspecified, with unspecified complications |
| ICD-10-CM | K5100 | Ulcerative (chronic) pancolitis without complications |
| ICD-10-CM | K51011 | Ulcerative (chronic) pancolitis with rectal bleeding |
| ICD-10-CM | K51012 | Ulcerative (chronic) pancolitis with intestinal obstruction |
| ICD-10-CM | K51013 | Ulcerative (chronic) pancolitis with fistula |
| ICD-10-CM | K51014 | Ulcerative (chronic) pancolitis with abscess |
| ICD-10-CM | K51018 | Ulcerative (chronic) pancolitis with other complication |
| ICD-10-CM | K51019 | Ulcerative (chronic) pancolitis with unspecified complications |
| ICD-10-CM | K5120 | Ulcerative (chronic) proctitis without complications |
| ICD-10-CM | K51211 | Ulcerative (chronic) proctitis with rectal bleeding |
| ICD-10-CM | K51212 | Ulcerative (chronic) proctitis with intestinal obstruction |
| ICD-10-CM | K51213 | Ulcerative (chronic) proctitis with fistula |
| ICD-10-CM | K51214 | Ulcerative (chronic) proctitis with abscess |
| ICD-10-CM | K51218 | Ulcerative (chronic) proctitis with other complication |
| ICD-10-CM | K51219 | Ulcerative (chronic) proctitis with unspecified complications |
| ICD-10-CM | K5130 | Ulcerative (chronic) rectosigmoiditis without complications |
| ICD-10-CM | K51311 | Ulcerative (chronic) rectosigmoiditis with rectal bleeding |
| ICD-10-CM | K51312 | Ulcerative (chronic) rectosigmoiditis with intestinal obstruction |
| ICD-10-CM | K51313 | Ulcerative (chronic) rectosigmoiditis with fistula |
| ICD-10-CM | K51314 | Ulcerative (chronic) rectosigmoiditis with abscess |
| ICD-10-CM | K51318 | Ulcerative (chronic) rectosigmoiditis with other complication |
| ICD-10-CM | K51319 | Ulcerative (chronic) rectosigmoiditis with unspecified complications |
| ICD-10-CM | K5140 | Inflammatory polyps of colon without complications |
| ICD-10-CM | K51411 | Inflammatory polyps of colon with rectal bleeding |
| ICD-10-CM | K51412 | Inflammatory polyps of colon with intestinal obstruction |
| ICD-10-CM | K51413 | Inflammatory polyps of colon with fistula |
| ICD-10-CM | K51414 | Inflammatory polyps of colon with abscess |
| ICD-10-CM | K51418 | Inflammatory polyps of colon with other complication |
| ICD-10-CM | K51419 | Inflammatory polyps of colon with unspecified complications |
| ICD-10-CM | K5150 | Left sided colitis without complications |
| ICD-10-CM | K51511 | Left sided colitis with rectal bleeding |
| ICD-10-CM | K51512 | Left sided colitis with intestinal obstruction |
| ICD-10-CM | K51513 | Left sided colitis with fistula |
| ICD-10-CM | K51514 | Left sided colitis with abscess |
| ICD-10-CM | K51518 | Left sided colitis with other complication |
| ICD-10-CM | K51519 | Left sided colitis with unspecified complications |
| ICD-10-CM | K5180 | Other ulcerative colitis without complications |
| ICD-10-CM | K51811 | Other ulcerative colitis with rectal bleeding |
| ICD-10-CM | K51812 | Other ulcerative colitis with intestinal obstruction |
| ICD-10-CM | K51813 | Other ulcerative colitis with fistula |
| ICD-10-CM | K51814 | Other ulcerative colitis with abscess |
| ICD-10-CM | K51818 | Other ulcerative colitis with other complication |
| ICD-10-CM | K51819 | Other ulcerative colitis with unspecified complications |
| ICD-10-CM | K5190 | Ulcerative colitis, unspecified, without complications |
| ICD-10-CM | K51911 | Ulcerative colitis, unspecified with rectal bleeding |
| ICD-10-CM | K51912 | Ulcerative colitis, unspecified with intestinal obstruction |
| ICD-10-CM | K51913 | Ulcerative colitis, unspecified with fistula |
| ICD-10-CM | K51914 | Ulcerative colitis, unspecified with abscess |
| ICD-10-CM | K51918 | Ulcerative colitis, unspecified with other complication |
| ICD-10-CM | K51919 | Ulcerative colitis, unspecified with unspecified complications |
|  |  |  |
| *Intestinal malabsorption* |  |  |
| ICD-10-CM | K90 | Intestinal malabsorption |
| SNOMED CT | 197476001 | Intestinal malabsorption |
| SNOMED CT | 197488004 | Other intestinal malabsorption |
| SNOMED CT | 197494007 | Intestinal malabsorption of fat |
| SNOMED CT | 78625002 | Hyperparathyroidism due to intestinal malabsorption |
| SNOMED CT | 197491004 | Intestinal malabsorption of carbohydrate |
| SNOMED CT | 197490003 | Intestinal malabsorption of protein |
| SNOMED CT | 420321004 | Intestinal malabsorption with AIDS |
| SNOMED CT | 717186009 | Intestinal malabsorption due to bile acid synthesis defect |
| SNOMED CT | 197497000 | Intestinal malabsorption NOS |
| SNOMED CT | 22892000 | AIDS with intestinal malabsorption |
| SNOMED CT | 197570005 | [X]Other intestinal malabsorption |
| SNOMED CT | 155846005 | (Intestinal malabsorption NOS) or (tropical sprue) |
| SNOMED CT | 266547002 | (Intestinal malabsorption NOS) or (tropical sprue) |
| SNOMED CT | 32230006 | Malabsorption syndrome |
| SNOMED CT | 197493001 | Malabsorption due to intolerance to soya protein |
| SNOMED CT | 235720008 | Malabsorption due to intolerance to carbohydrate |
| SNOMED CT | 302921006 | Malabsorption due to intolerance to protein |
| SNOMED CT | 303062005 | Malabsorption due to intolerance to fat |
| SNOMED CT | 26629001 | Short bowel syndrome |
| SNOMED CT | 27868004 | Chronic steatorrhea |
| SNOMED CT | 36261000 | Drug-induced malabsorption |
| SNOMED CT | 47384003 | Tropical sprue |
| SNOMED CT | 54576000 | Pancreatic steatorrhea |
| SNOMED CT | 59285001 | Bile acid malabsorption syndrome type II |
| SNOMED CT | 61738006 | Non-gluten sensitive enteropathy syndrome |
| SNOMED CT | 69478001 | Pancreatic colipase deficiency |
| SNOMED CT | 77147000 | Malabsorption in the elderly |
| SNOMED CT | 77225009 | Blind loop syndrome |
| SNOMED CT | 81704009 | Sprue |
| SNOMED CT | 235723005 | Chemically-induced malabsorption |
| SNOMED CT | 235724004 | Acquired short bowel syndrome |
| SNOMED CT | 235725003 | Malabsorption due to infective agent |
| SNOMED CT | 235727006 | Post-infective malabsorption |
| SNOMED CT | 359653006 | Unclassified sprue |
| SNOMED CT | 713505000 | Gastrointestinal malabsorption syndrome co-occurrent with human immunodeficiency virus infection |
| SNOMED CT | 715201005 | Congenital short bowel syndrome |
| SNOMED CT | 717186009 | Intestinal malabsorption due to bile acid synthesis defect |
| SNOMED CT | 785704008 | Malabsorption syndrome due to acquired intolerance to fructose |
| SNOMED CT | 201517005 | Seronegative arthritis secondary to Whipple disease |
| SNOMED CT | 230194002 | Neuro - Whipple disease |
| SNOMED CT | 78960005 | Pancreatic triacylglycerol lipase deficiency |
| SNOMED CT | 197495008 | Malabsorption - iron |
| SNOMED CT | 733148009 | Duodenitis caused by Tropheryma whipplei |
|  |  |  |
| *Limb amputation* |  |  |
| CPT | 23900 | Interthoracoscapular amputation (forequarter) |
| CPT | 23920 | Disarticulation of shoulder; |
| CPT | 24900 | Amputation, arm through humerus; with primary closure |
| CPT | 24920 | Amputation, arm through humerus; open, circular (guillotine) |
| CPT | 24930 | Amputation, arm through humerus; re-amputation |
| CPT | 24931 | Amputation, arm through humerus; with implant |
| CPT | 25900 | Amputation, forearm, through radius and ulna; |
| CPT | 25905 | Amputation, forearm, through radius and ulna; open, circular (guillotine) |
| CPT | 25909 | Amputation, forearm, through radius and ulna; re-amputation |
| CPT | 27290 | Interpelviabdominal amputation (hindquarter amputation) |
| CPT | 27295 | Disarticulation of hip |
| CPT | 27590 | Amputation, thigh, through femur, any level; |
| CPT | 27591 | Amputation, thigh, through femur, any level; immediate fitting technique including first cast |
| CPT | 27592 | Amputation, thigh, through femur, any level; open, circular (guillotine) |
| CPT | 27596 | Amputation, thigh, through femur, any level; re-amputation |
| CPT | 27598 | Disarticulation at knee |
| CPT | 27880 | Amputation, leg, through tibia and fibula; |
| CPT | 27881 | Amputation, leg, through tibia and fibula; with immediate fitting technique including application of first cast |
| CPT | 27882 | Amputation, leg, through tibia and fibula; open, circular (guillotine) |
| CPT | 27886 | Amputation, leg, through tibia and fibula; re-amputation |
| CPT | 27888 | Amputation, ankle, through malleoli of tibia and fibula (eg, Syme, Pirogoff type procedures), with plastic closure and resection of nerves |
| ICD-10-PCS | 0X600ZZ | Detachment at Right Forequarter, Open Approach |
| ICD-10-PCS | 0X610ZZ | Detachment at Left Forequarter, Open Approach |
| ICD-10-PCS | 0X620ZZ | Detachment at Right Shoulder Region, Open Approach |
| ICD-10-PCS | 0X630ZZ | Detachment at Left Shoulder Region, Open Approach |
| ICD-10-PCS | 0X680Z1 | Detachment at Right Upper Arm, High, Open Approach |
| ICD-10-PCS | 0X680Z2 | Detachment at Right Upper Arm, Mid, Open Approach |
| ICD-10-PCS | 0X680Z3 | Detachment at Right Upper Arm, Low, Open Approach |
| ICD-10-PCS | 0X690Z1 | Detachment at Left Upper Arm, High, Open Approach |
| ICD-10-PCS | 0X690Z2 | Detachment at Left Upper Arm, Mid, Open Approach |
| ICD-10-PCS | 0X690Z3 | Detachment at Left Upper Arm, Low, Open Approach |
| ICD-10-PCS | 0X6B0ZZ | Detachment at Right Elbow Region, Open Approach |
| ICD-10-PCS | 0X6C0ZZ | Detachment at Left Elbow Region, Open Approach |
| ICD-10-PCS | 0X6D0Z1 | Detachment at Right Lower Arm, High, Open Approach |
| ICD-10-PCS | 0X6D0Z2 | Detachment at Right Lower Arm, Mid, Open Approach |
| ICD-10-PCS | 0X6D0Z3 | Detachment at Right Lower Arm, Low, Open Approach |
| ICD-10-PCS | 0X6F0Z1 | Detachment at Left Lower Arm, High, Open Approach |
| ICD-10-PCS | 0X6F0Z2 | Detachment at Left Lower Arm, Mid, Open Approach |
| ICD-10-PCS | 0X6F0Z3 | Detachment at Left Lower Arm, Low, Open Approach |
| ICD-10-PCS | 0Y620ZZ | Detachment at Right Hindquarter, Open Approach |
| ICD-10-PCS | 0Y630ZZ | Detachment at Left Hindquarter, Open Approach |
| ICD-10-PCS | 0Y640ZZ | Detachment at Bilateral Hindquarter, Open Approach |
| ICD-10-PCS | 0Y670ZZ | Detachment at Right Femoral Region, Open Approach |
| ICD-10-PCS | 0Y680ZZ | Detachment at Left Femoral Region, Open Approach |
| ICD-10-PCS | 0Y6C0Z1 | Detachment at Right Upper Leg, High, Open Approach |
| ICD-10-PCS | 0Y6C0Z2 | Detachment at Right Upper Leg, Mid, Open Approach |
| ICD-10-PCS | 0Y6C0Z3 | Detachment at Right Upper Leg, Low, Open Approach |
| ICD-10-PCS | 0Y6D0Z1 | Detachment at Left Upper Leg, High, Open Approach |
| ICD-10-PCS | 0Y6D0Z2 | Detachment at Left Upper Leg, Mid, Open Approach |
| ICD-10-PCS | 0Y6D0Z3 | Detachment at Left Upper Leg, Low, Open Approach |
| ICD-10-PCS | 0Y6F0ZZ | Detachment at Right Knee Region, Open Approach |
| ICD-10-PCS | 0Y6G0ZZ | Detachment at Left Knee Region, Open Approach |
| ICD-10-PCS | 0Y6H0Z1 | Detachment at Right Lower Leg, High, Open Approach |
| ICD-10-PCS | 0Y6H0Z2 | Detachment at Right Lower Leg, Mid, Open Approach |
| ICD-10-PCS | 0Y6H0Z3 | Detachment at Right Lower Leg, Low, Open Approach |
| ICD-10-PCS | 0Y6J0Z1 | Detachment at Left Lower Leg, High, Open Approach |
| ICD-10-PCS | 0Y6J0Z2 | Detachment at Left Lower Leg, Mid, Open Approach |
| ICD-10-PCS | 0Y6J0Z3 | Detachment at Left Lower Leg, Low, Open Approach |
|  |  |  |
| *Liver cirrhosis* |  |  |
| ICD-10-CM | K70.2 | Alcoholic fibrosis and sclerosis of liver |
| ICD-10-CM | K70.41 | Alcoholic hepatic failure with coma |
| ICD-10-CM | K70.3 | Alcoholic cirrhosis of liver [Non-Specific Code] |
| ICD-10-CM | K71.11 | Toxic liver disease with hepatic necrosis with coma |
| ICD-10-CM | K71.7 | Toxic liver disease with fibrosis and cirrhosis of liver |
| ICD-10-CM | K72.01 | Acute and subacute hepatic failure with coma |
| ICD-10-CM | K72.11 | Chronic hepatic failure with coma |
| ICD-10-CM | K72.91 | Hepatic failure, unspecified with coma |
| ICD-10-CM | K74.3 | Primary biliary cirrhosis |
| ICD-10-CM | K74.4 | Secondary biliary cirrhosis |
| ICD-10-CM | K74.5 | Biliary cirrhosis, unspecified |
| ICD-10-CM | K74.6 | Other and unspecified cirrhosis of liver [Non-Specific Code] |
| ICD-10-CM | K74.60 | Unspecified cirrhosis of liver |
| ICD-10-CM | K74.69 | Other cirrhosis of liver |
| ICD-10-CM | K76.6 | Portal hypertension |
| ICD-10-CM | K76.7 | Hepatorenal syndrome |
| ICD-10-CM | P78.81 | Congenital cirrhosis (of liver) |
| ICD-10-CM | I85 | Esophageal varices |
| CPT | 43204 | ESOPHAGOSCOPY FLEX TRANSORAL INJECTION VARICES |
| CPT | 43205 | ESPHGOSCOPY FLEX W/BAND LIGATION ESOPHGL VARICES |
| CPT | 43400 | LIGATION DIRECT ESOPHAGEAL VARICES |
| CPT | 43401 | Transection of esophagus with repair, for esophageal varices |
|  |  |  |
| *Malignant cancer* |  |  |
| ICD-10-CM | C01 | Malignant neoplasm of base of tongue |
| ICD-10-CM | C020 | Malignant neoplasm of dorsal surface of tongue |
| ICD-10-CM | C021 | Malignant neoplasm of border of tongue |
| ICD-10-CM | C022 | Malignant neoplasm of ventral surface of tongue |
| ICD-10-CM | C023 | Malignant neoplasm of anterior two-thirds of tongue, part unspecified |
| ICD-10-CM | C024 | Malignant neoplasm of lingual tonsil |
| ICD-10-CM | C028 | Malignant neoplasm of overlapping sites of tongue |
| ICD-10-CM | C029 | Malignant neoplasm of tongue, unspecified |
| ICD-10-CM | C030 | Malignant neoplasm of upper gum |
| ICD-10-CM | C031 | Malignant neoplasm of lower gum |
| ICD-10-CM | C039 | Malignant neoplasm of gum, unspecified |
| ICD-10-CM | C040 | Malignant neoplasm of anterior floor of mouth |
| ICD-10-CM | C041 | Malignant neoplasm of lateral floor of mouth |
| ICD-10-CM | C048 | Malignant neoplasm of overlapping sites of floor of mouth |
| ICD-10-CM | C049 | Malignant neoplasm of floor of mouth, unspecified |
| ICD-10-CM | C050 | Malignant neoplasm of hard palate |
| ICD-10-CM | C051 | Malignant neoplasm of soft palate |
| ICD-10-CM | C052 | Malignant neoplasm of uvula |
| ICD-10-CM | C058 | Malignant neoplasm of overlapping sites of palate |
| ICD-10-CM | C059 | Malignant neoplasm of palate, unspecified |
| ICD-10-CM | C060 | Malignant neoplasm of cheek mucosa |
| ICD-10-CM | C061 | Malignant neoplasm of vestibule of mouth |
| ICD-10-CM | C062 | Malignant neoplasm of retromolar area |
| ICD-10-CM | C0680 | Malignant neoplasm of overlapping sites of unspecified parts of mouth |
| ICD-10-CM | C0689 | Malignant neoplasm of overlapping sites of other parts of mouth |
| ICD-10-CM | C069 | Malignant neoplasm of mouth, unspecified |
| ICD-10-CM | C07 | Malignant neoplasm of parotid gland |
| ICD-10-CM | C080 | Malignant neoplasm of submandibular gland |
| ICD-10-CM | C081 | Malignant neoplasm of sublingual gland |
| ICD-10-CM | C089 | Malignant neoplasm of major salivary gland, unspecified |
| ICD-10-CM | C090 | Malignant neoplasm of tonsillar fossa |
| ICD-10-CM | C091 | Malignant neoplasm of tonsillar pillar (anterior) (posterior) |
| ICD-10-CM | C098 | Malignant neoplasm of overlapping sites of tonsil |
| ICD-10-CM | C099 | Malignant neoplasm of tonsil, unspecified |
| ICD-10-CM | C100 | Malignant neoplasm of vallecula |
| ICD-10-CM | C101 | Malignant neoplasm of anterior surface of epiglottis |
| ICD-10-CM | C102 | Malignant neoplasm of lateral wall of oropharynx |
| ICD-10-CM | C103 | Malignant neoplasm of posterior wall of oropharynx |
| ICD-10-CM | C104 | Malignant neoplasm of branchial cleft |
| ICD-10-CM | C108 | Malignant neoplasm of overlapping sites of oropharynx |
| ICD-10-CM | C109 | Malignant neoplasm of oropharynx, unspecified |
| ICD-10-CM | C110 | Malignant neoplasm of superior wall of nasopharynx |
| ICD-10-CM | C111 | Malignant neoplasm of posterior wall of nasopharynx |
| ICD-10-CM | C112 | Malignant neoplasm of lateral wall of nasopharynx |
| ICD-10-CM | C113 | Malignant neoplasm of anterior wall of nasopharynx |
| ICD-10-CM | C118 | Malignant neoplasm of overlapping sites of nasopharynx |
| ICD-10-CM | C119 | Malignant neoplasm of nasopharynx, unspecified |
| ICD-10-CM | C12 | Malignant neoplasm of pyriform sinus |
| ICD-10-CM | C130 | Malignant neoplasm of postcricoid region |
| ICD-10-CM | C131 | Malignant neoplasm of aryepiglottic fold, hypopharyngeal aspect |
| ICD-10-CM | C132 | Malignant neoplasm of posterior wall of hypopharynx |
| ICD-10-CM | C138 | Malignant neoplasm of overlapping sites of hypopharynx |
| ICD-10-CM | C139 | Malignant neoplasm of hypopharynx, unspecified |
| ICD-10-CM | C140 | Malignant neoplasm of pharynx, unspecified |
| ICD-10-CM | C142 | Malignant neoplasm of Waldeyer's ring |
| ICD-10-CM | C148 | Malignant neoplasm of overlapping sites of lip, oral cavity and pharynx |
| ICD-10-CM | C153 | Malignant neoplasm of upper third of esophagus |
| ICD-10-CM | C154 | Malignant neoplasm of middle third of esophagus |
| ICD-10-CM | C155 | Malignant neoplasm of lower third of esophagus |
| ICD-10-CM | C158 | Malignant neoplasm of overlapping sites of esophagus |
| ICD-10-CM | C159 | Malignant neoplasm of esophagus, unspecified |
| ICD-10-CM | C160 | Malignant neoplasm of cardia |
| ICD-10-CM | C161 | Malignant neoplasm of fundus of stomach |
| ICD-10-CM | C162 | Malignant neoplasm of body of stomach |
| ICD-10-CM | C163 | Malignant neoplasm of pyloric antrum |
| ICD-10-CM | C164 | Malignant neoplasm of pylorus |
| ICD-10-CM | C165 | Malignant neoplasm of lesser curvature of stomach, unspecified |
| ICD-10-CM | C166 | Malignant neoplasm of greater curvature of stomach, unspecified |
| ICD-10-CM | C168 | Malignant neoplasm of overlapping sites of stomach |
| ICD-10-CM | C169 | Malignant neoplasm of stomach, unspecified |
| ICD-10-CM | C170 | Malignant neoplasm of duodenum |
| ICD-10-CM | C171 | Malignant neoplasm of jejunum |
| ICD-10-CM | C172 | Malignant neoplasm of ileum |
| ICD-10-CM | C173 | Meckel's diverticulum, malignant |
| ICD-10-CM | C178 | Malignant neoplasm of overlapping sites of small intestine |
| ICD-10-CM | C179 | Malignant neoplasm of small intestine, unspecified |
| ICD-10-CM | C180 | Malignant neoplasm of cecum |
| ICD-10-CM | C181 | Malignant neoplasm of appendix |
| ICD-10-CM | C182 | Malignant neoplasm of ascending colon |
| ICD-10-CM | C183 | Malignant neoplasm of hepatic flexure |
| ICD-10-CM | C184 | Malignant neoplasm of transverse colon |
| ICD-10-CM | C185 | Malignant neoplasm of splenic flexure |
| ICD-10-CM | C186 | Malignant neoplasm of descending colon |
| ICD-10-CM | C187 | Malignant neoplasm of sigmoid colon |
| ICD-10-CM | C188 | Malignant neoplasm of overlapping sites of colon |
| ICD-10-CM | C189 | Malignant neoplasm of colon, unspecified |
| ICD-10-CM | C19 | Malignant neoplasm of rectosigmoid junction |
| ICD-10-CM | C20 | Malignant neoplasm of rectum |
| ICD-10-CM | C210 | Malignant neoplasm of anus, unspecified |
| ICD-10-CM | C211 | Malignant neoplasm of anal canal |
| ICD-10-CM | C212 | Malignant neoplasm of cloacogenic zone |
| ICD-10-CM | C218 | Malignant neoplasm of overlapping sites of rectum, anus and anal canal |
| ICD-10-CM | C220 | Liver cell carcinoma |
| ICD-10-CM | C221 | Intrahepatic bile duct carcinoma |
| ICD-10-CM | C222 | Hepatoblastoma |
| ICD-10-CM | C223 | Angiosarcoma of liver |
| ICD-10-CM | C224 | Other sarcomas of liver |
| ICD-10-CM | C227 | Other specified carcinomas of liver |
| ICD-10-CM | C228 | Malignant neoplasm of liver, primary, unspecified as to type |
| ICD-10-CM | C229 | Malignant neoplasm of liver, not specified as primary or secondary |
| ICD-10-CM | C23 | Malignant neoplasm of gallbladder |
| ICD-10-CM | C240 | Malignant neoplasm of extrahepatic bile duct |
| ICD-10-CM | C241 | Malignant neoplasm of ampulla of Vater |
| ICD-10-CM | C248 | Malignant neoplasm of overlapping sites of biliary tract |
| ICD-10-CM | C249 | Malignant neoplasm of biliary tract, unspecified |
| ICD-10-CM | C250 | Malignant neoplasm of head of pancreas |
| ICD-10-CM | C251 | Malignant neoplasm of body of pancreas |
| ICD-10-CM | C252 | Malignant neoplasm of tail of pancreas |
| ICD-10-CM | C253 | Malignant neoplasm of pancreatic duct |
| ICD-10-CM | C254 | Malignant neoplasm of endocrine pancreas |
| ICD-10-CM | C257 | Malignant neoplasm of other parts of pancreas |
| ICD-10-CM | C258 | Malignant neoplasm of overlapping sites of pancreas |
| ICD-10-CM | C259 | Malignant neoplasm of pancreas, unspecified |
| ICD-10-CM | C260 | Malignant neoplasm of intestinal tract, part unspecified |
| ICD-10-CM | C261 | Malignant neoplasm of spleen |
| ICD-10-CM | C269 | Malignant neoplasm of ill-defined sites within the digestive system |
| ICD-10-CM | C300 | Malignant neoplasm of nasal cavity |
| ICD-10-CM | C301 | Malignant neoplasm of middle ear |
| ICD-10-CM | C310 | Malignant neoplasm of maxillary sinus |
| ICD-10-CM | C311 | Malignant neoplasm of ethmoidal sinus |
| ICD-10-CM | C312 | Malignant neoplasm of frontal sinus |
| ICD-10-CM | C313 | Malignant neoplasm of sphenoid sinus |
| ICD-10-CM | C318 | Malignant neoplasm of overlapping sites of accessory sinuses |
| ICD-10-CM | C319 | Malignant neoplasm of accessory sinus, unspecified |
| ICD-10-CM | C320 | Malignant neoplasm of glottis |
| ICD-10-CM | C321 | Malignant neoplasm of supraglottis |
| ICD-10-CM | C322 | Malignant neoplasm of subglottis |
| ICD-10-CM | C323 | Malignant neoplasm of laryngeal cartilage |
| ICD-10-CM | C328 | Malignant neoplasm of overlapping sites of larynx |
| ICD-10-CM | C329 | Malignant neoplasm of larynx, unspecified |
| ICD-10-CM | C33 | Malignant neoplasm of trachea |
| ICD-10-CM | C3400 | Malignant neoplasm of unspecified main bronchus |
| ICD-10-CM | C3401 | Malignant neoplasm of right main bronchus |
| ICD-10-CM | C3402 | Malignant neoplasm of left main bronchus |
| ICD-10-CM | C3410 | Malignant neoplasm of upper lobe, unspecified bronchus or lung |
| ICD-10-CM | C3411 | Malignant neoplasm of upper lobe, right bronchus or lung |
| ICD-10-CM | C3412 | Malignant neoplasm of upper lobe, left bronchus or lung |
| ICD-10-CM | C342 | Malignant neoplasm of middle lobe, bronchus or lung |
| ICD-10-CM | C3430 | Malignant neoplasm of lower lobe, unspecified bronchus or lung |
| ICD-10-CM | C3431 | Malignant neoplasm of lower lobe, right bronchus or lung |
| ICD-10-CM | C3432 | Malignant neoplasm of lower lobe, left bronchus or lung |
| ICD-10-CM | C3480 | Malignant neoplasm of overlapping sites of unspecified bronchus and lung |
| ICD-10-CM | C3481 | Malignant neoplasm of overlapping sites of right bronchus and lung |
| ICD-10-CM | C3482 | Malignant neoplasm of overlapping sites of left bronchus and lung |
| ICD-10-CM | C3490 | Malignant neoplasm of unspecified part of unspecified bronchus or lung |
| ICD-10-CM | C3491 | Malignant neoplasm of unspecified part of right bronchus or lung |
| ICD-10-CM | C3492 | Malignant neoplasm of unspecified part of left bronchus or lung |
| ICD-10-CM | C37 | Malignant neoplasm of thymus |
| ICD-10-CM | C380 | Malignant neoplasm of heart |
| ICD-10-CM | C381 | Malignant neoplasm of anterior mediastinum |
| ICD-10-CM | C382 | Malignant neoplasm of posterior mediastinum |
| ICD-10-CM | C383 | Malignant neoplasm of mediastinum, part unspecified |
| ICD-10-CM | C384 | Malignant neoplasm of pleura |
| ICD-10-CM | C388 | Malignant neoplasm of overlapping sites of heart, mediastinum and pleura |
| ICD-10-CM | C390 | Malignant neoplasm of upper respiratory tract, part unspecified |
| ICD-10-CM | C399 | Malignant neoplasm of lower respiratory tract, part unspecified |
| ICD-10-CM | C4000 | Malignant neoplasm of scapula and long bones of unspecified upper limb |
| ICD-10-CM | C4001 | Malignant neoplasm of scapula and long bones of right upper limb |
| ICD-10-CM | C4002 | Malignant neoplasm of scapula and long bones of left upper limb |
| ICD-10-CM | C4010 | Malignant neoplasm of short bones of unspecified upper limb |
| ICD-10-CM | C4011 | Malignant neoplasm of short bones of right upper limb |
| ICD-10-CM | C4012 | Malignant neoplasm of short bones of left upper limb |
| ICD-10-CM | C4020 | Malignant neoplasm of long bones of unspecified lower limb |
| ICD-10-CM | C4021 | Malignant neoplasm of long bones of right lower limb |
| ICD-10-CM | C4022 | Malignant neoplasm of long bones of left lower limb |
| ICD-10-CM | C4030 | Malignant neoplasm of short bones of unspecified lower limb |
| ICD-10-CM | C4031 | Malignant neoplasm of short bones of right lower limb |
| ICD-10-CM | C4032 | Malignant neoplasm of short bones of left lower limb |
| ICD-10-CM | C4080 | Malignant neoplasm of overlapping sites of bone and articular cartilage of unspecified limb |
| ICD-10-CM | C4081 | Malignant neoplasm of overlapping sites of bone and articular cartilage of right limb |
| ICD-10-CM | C4082 | Malignant neoplasm of overlapping sites of bone and articular cartilage of left limb |
| ICD-10-CM | C4090 | Malignant neoplasm of unspecified bones and articular cartilage of unspecified limb |
| ICD-10-CM | C4091 | Malignant neoplasm of unspecified bones and articular cartilage of right limb |
| ICD-10-CM | C4092 | Malignant neoplasm of unspecified bones and articular cartilage of left limb |
| ICD-10-CM | C410 | Malignant neoplasm of bones of skull and face |
| ICD-10-CM | C411 | Malignant neoplasm of mandible |
| ICD-10-CM | C412 | Malignant neoplasm of vertebral column |
| ICD-10-CM | C413 | Malignant neoplasm of ribs, sternum and clavicle |
| ICD-10-CM | C414 | Malignant neoplasm of pelvic bones, sacrum and coccyx |
| ICD-10-CM | C419 | Malignant neoplasm of bone and articular cartilage, unspecified |
| ICD-10-CM | C430 | Malignant melanoma of lip |
| ICD-10-CM | C4310 | Malignant melanoma of unspecified eyelid, including canthus |
| ICD-10-CM | C4311 | Malignant melanoma of right eyelid, including canthus |
| ICD-10-CM | C43111 | Malignant melanoma of right upper eyelid, including canthus |
| ICD-10-CM | C43112 | Malignant melanoma of right lower eyelid, including canthus |
| ICD-10-CM | C4312 | Malignant melanoma of left eyelid, including canthus |
| ICD-10-CM | C43121 | Malignant melanoma of left upper eyelid, including canthus |
| ICD-10-CM | C43122 | Malignant melanoma of left lower eyelid, including canthus |
| ICD-10-CM | C4320 | Malignant melanoma of unspecified ear and external auricular canal |
| ICD-10-CM | C4321 | Malignant melanoma of right ear and external auricular canal |
| ICD-10-CM | C4322 | Malignant melanoma of left ear and external auricular canal |
| ICD-10-CM | C4330 | Malignant melanoma of unspecified part of face |
| ICD-10-CM | C4331 | Malignant melanoma of nose |
| ICD-10-CM | C4339 | Malignant melanoma of other parts of face |
| ICD-10-CM | C434 | Malignant melanoma of scalp and neck |
| ICD-10-CM | C4351 | Malignant melanoma of anal skin |
| ICD-10-CM | C4352 | Malignant melanoma of skin of breast |
| ICD-10-CM | C4359 | Malignant melanoma of other part of trunk |
| ICD-10-CM | C4360 | Malignant melanoma of unspecified upper limb, including shoulder |
| ICD-10-CM | C4361 | Malignant melanoma of right upper limb, including shoulder |
| ICD-10-CM | C4362 | Malignant melanoma of left upper limb, including shoulder |
| ICD-10-CM | C4370 | Malignant melanoma of unspecified lower limb, including hip |
| ICD-10-CM | C4371 | Malignant melanoma of right lower limb, including hip |
| ICD-10-CM | C4372 | Malignant melanoma of left lower limb, including hip |
| ICD-10-CM | C438 | Malignant melanoma of overlapping sites of skin |
| ICD-10-CM | C439 | Malignant melanoma of skin, unspecified |
| ICD-10-CM | C450 | Mesothelioma of pleura |
| ICD-10-CM | C451 | Mesothelioma of peritoneum |
| ICD-10-CM | C452 | Mesothelioma of pericardium |
| ICD-10-CM | C457 | Mesothelioma of other sites |
| ICD-10-CM | C459 | Mesothelioma, unspecified |
| ICD-10-CM | C460 | Kaposi's sarcoma of skin |
| ICD-10-CM | C461 | Kaposi's sarcoma of soft tissue |
| ICD-10-CM | C462 | Kaposi's sarcoma of palate |
| ICD-10-CM | C463 | Kaposi's sarcoma of lymph nodes |
| ICD-10-CM | C464 | Kaposi's sarcoma of gastrointestinal sites |
| ICD-10-CM | C4650 | Kaposi's sarcoma of unspecified lung |
| ICD-10-CM | C4651 | Kaposi's sarcoma of right lung |
| ICD-10-CM | C4652 | Kaposi's sarcoma of left lung |
| ICD-10-CM | C467 | Kaposi's sarcoma of other sites |
| ICD-10-CM | C469 | Kaposi's sarcoma, unspecified |
| ICD-10-CM | C470 | Malignant neoplasm of peripheral nerves of head, face and neck |
| ICD-10-CM | C4710 | Malignant neoplasm of peripheral nerves of unspecified upper limb, including shoulder |
| ICD-10-CM | C4711 | Malignant neoplasm of peripheral nerves of right upper limb, including shoulder |
| ICD-10-CM | C4712 | Malignant neoplasm of peripheral nerves of left upper limb, including shoulder |
| ICD-10-CM | C4720 | Malignant neoplasm of peripheral nerves of unspecified lower limb, including hip |
| ICD-10-CM | C4721 | Malignant neoplasm of peripheral nerves of right lower limb, including hip |
| ICD-10-CM | C4722 | Malignant neoplasm of peripheral nerves of left lower limb, including hip |
| ICD-10-CM | C473 | Malignant neoplasm of peripheral nerves of thorax |
| ICD-10-CM | C474 | Malignant neoplasm of peripheral nerves of abdomen |
| ICD-10-CM | C475 | Malignant neoplasm of peripheral nerves of pelvis |
| ICD-10-CM | C476 | Malignant neoplasm of peripheral nerves of trunk, unspecified |
| ICD-10-CM | C478 | Malignant neoplasm of overlapping sites of peripheral nerves and autonomic nervous system |
| ICD-10-CM | C479 | Malignant neoplasm of peripheral nerves and autonomic nervous system, unspecified |
| ICD-10-CM | C480 | Malignant neoplasm of retroperitoneum |
| ICD-10-CM | C481 | Malignant neoplasm of specified parts of peritoneum |
| ICD-10-CM | C482 | Malignant neoplasm of peritoneum, unspecified |
| ICD-10-CM | C488 | Malignant neoplasm of overlapping sites of retroperitoneum and peritoneum |
| ICD-10-CM | C490 | Malignant neoplasm of connective and soft tissue of head, face and neck |
| ICD-10-CM | C4910 | Malignant neoplasm of connective and soft tissue of unspecified upper limb, including shoulder |
| ICD-10-CM | C4911 | Malignant neoplasm of connective and soft tissue of right upper limb, including shoulder |
| ICD-10-CM | C4912 | Malignant neoplasm of connective and soft tissue of left upper limb, including shoulder |
| ICD-10-CM | C4920 | Malignant neoplasm of connective and soft tissue of unspecified lower limb, including hip |
| ICD-10-CM | C4921 | Malignant neoplasm of connective and soft tissue of right lower limb, including hip |
| ICD-10-CM | C4922 | Malignant neoplasm of connective and soft tissue of left lower limb, including hip |
| ICD-10-CM | C493 | Malignant neoplasm of connective and soft tissue of thorax |
| ICD-10-CM | C494 | Malignant neoplasm of connective and soft tissue of abdomen |
| ICD-10-CM | C495 | Malignant neoplasm of connective and soft tissue of pelvis |
| ICD-10-CM | C496 | Malignant neoplasm of connective and soft tissue of trunk, unspecified |
| ICD-10-CM | C498 | Malignant neoplasm of overlapping sites of connective and soft tissue |
| ICD-10-CM | C499 | Malignant neoplasm of connective and soft tissue, unspecified |
| ICD-10-CM | C49A0 | Gastrointestinal stromal tumor, unspecified site |
| ICD-10-CM | C49A1 | Gastrointestinal stromal tumor of esophagus |
| ICD-10-CM | C49A2 | Gastrointestinal stromal tumor of stomach |
| ICD-10-CM | C49A3 | Gastrointestinal stromal tumor of small intestine |
| ICD-10-CM | C49A4 | Gastrointestinal stromal tumor of large intestine |
| ICD-10-CM | C49A5 | Gastrointestinal stromal tumor of rectum |
| ICD-10-CM | C49A9 | Gastrointestinal stromal tumor of other sites |
| ICD-10-CM | C4A0 | Merkel cell carcinoma of lip |
| ICD-10-CM | C4A10 | Merkel cell carcinoma of unspecified eyelid, including canthus |
| ICD-10-CM | C4A11 | Merkel cell carcinoma of right eyelid, including canthus |
| ICD-10-CM | C4A111 | Merkel cell carcinoma of right upper eyelid, including canthus |
| ICD-10-CM | C4A112 | Merkel cell carcinoma of right lower eyelid, including canthus |
| ICD-10-CM | C4A12 | Merkel cell carcinoma of left eyelid, including canthus |
| ICD-10-CM | C4A121 | Merkel cell carcinoma of left upper eyelid, including canthus |
| ICD-10-CM | C4A122 | Merkel cell carcinoma of left lower eyelid, including canthus |
| ICD-10-CM | C4A20 | Merkel cell carcinoma of unspecified ear and external auricular canal |
| ICD-10-CM | C4A21 | Merkel cell carcinoma of right ear and external auricular canal |
| ICD-10-CM | C4A22 | Merkel cell carcinoma of left ear and external auricular canal |
| ICD-10-CM | C4A30 | Merkel cell carcinoma of unspecified part of face |
| ICD-10-CM | C4A31 | Merkel cell carcinoma of nose |
| ICD-10-CM | C4A39 | Merkel cell carcinoma of other parts of face |
| ICD-10-CM | C4A4 | Merkel cell carcinoma of scalp and neck |
| ICD-10-CM | C4A51 | Merkel cell carcinoma of anal skin |
| ICD-10-CM | C4A52 | Merkel cell carcinoma of skin of breast |
| ICD-10-CM | C4A59 | Merkel cell carcinoma of other part of trunk |
| ICD-10-CM | C4A60 | Merkel cell carcinoma of unspecified upper limb, including shoulder |
| ICD-10-CM | C4A61 | Merkel cell carcinoma of right upper limb, including shoulder |
| ICD-10-CM | C4A62 | Merkel cell carcinoma of left upper limb, including shoulder |
| ICD-10-CM | C4A70 | Merkel cell carcinoma of unspecified lower limb, including hip |
| ICD-10-CM | C4A71 | Merkel cell carcinoma of right lower limb, including hip |
| ICD-10-CM | C4A72 | Merkel cell carcinoma of left lower limb, including hip |
| ICD-10-CM | C4A8 | Merkel cell carcinoma of overlapping sites |
| ICD-10-CM | C4A9 | Merkel cell carcinoma, unspecified |
| ICD-10-CM | C50011 | Malignant neoplasm of nipple and areola, right female breast |
| ICD-10-CM | C50012 | Malignant neoplasm of nipple and areola, left female breast |
| ICD-10-CM | C50019 | Malignant neoplasm of nipple and areola, unspecified female breast |
| ICD-10-CM | C50021 | Malignant neoplasm of nipple and areola, right male breast |
| ICD-10-CM | C50022 | Malignant neoplasm of nipple and areola, left male breast |
| ICD-10-CM | C50029 | Malignant neoplasm of nipple and areola, unspecified male breast |
| ICD-10-CM | C50111 | Malignant neoplasm of central portion of right female breast |
| ICD-10-CM | C50112 | Malignant neoplasm of central portion of left female breast |
| ICD-10-CM | C50119 | Malignant neoplasm of central portion of unspecified female breast |
| ICD-10-CM | C50121 | Malignant neoplasm of central portion of right male breast |
| ICD-10-CM | C50122 | Malignant neoplasm of central portion of left male breast |
| ICD-10-CM | C50129 | Malignant neoplasm of central portion of unspecified male breast |
| ICD-10-CM | C50211 | Malignant neoplasm of upper-inner quadrant of right female breast |
| ICD-10-CM | C50212 | Malignant neoplasm of upper-inner quadrant of left female breast |
| ICD-10-CM | C50219 | Malignant neoplasm of upper-inner quadrant of unspecified female breast |
| ICD-10-CM | C50221 | Malignant neoplasm of upper-inner quadrant of right male breast |
| ICD-10-CM | C50222 | Malignant neoplasm of upper-inner quadrant of left male breast |
| ICD-10-CM | C50229 | Malignant neoplasm of upper-inner quadrant of unspecified male breast |
| ICD-10-CM | C50311 | Malignant neoplasm of lower-inner quadrant of right female breast |
| ICD-10-CM | C50312 | Malignant neoplasm of lower-inner quadrant of left female breast |
| ICD-10-CM | C50319 | Malignant neoplasm of lower-inner quadrant of unspecified female breast |
| ICD-10-CM | C50321 | Malignant neoplasm of lower-inner quadrant of right male breast |
| ICD-10-CM | C50322 | Malignant neoplasm of lower-inner quadrant of left male breast |
| ICD-10-CM | C50329 | Malignant neoplasm of lower-inner quadrant of unspecified male breast |
| ICD-10-CM | C50411 | Malignant neoplasm of upper-outer quadrant of right female breast |
| ICD-10-CM | C50412 | Malignant neoplasm of upper-outer quadrant of left female breast |
| ICD-10-CM | C50419 | Malignant neoplasm of upper-outer quadrant of unspecified female breast |
| ICD-10-CM | C50421 | Malignant neoplasm of upper-outer quadrant of right male breast |
| ICD-10-CM | C50422 | Malignant neoplasm of upper-outer quadrant of left male breast |
| ICD-10-CM | C50429 | Malignant neoplasm of upper-outer quadrant of unspecified male breast |
| ICD-10-CM | C50511 | Malignant neoplasm of lower-outer quadrant of right female breast |
| ICD-10-CM | C50512 | Malignant neoplasm of lower-outer quadrant of left female breast |
| ICD-10-CM | C50519 | Malignant neoplasm of lower-outer quadrant of unspecified female breast |
| ICD-10-CM | C50521 | Malignant neoplasm of lower-outer quadrant of right male breast |
| ICD-10-CM | C50522 | Malignant neoplasm of lower-outer quadrant of left male breast |
| ICD-10-CM | C50529 | Malignant neoplasm of lower-outer quadrant of unspecified male breast |
| ICD-10-CM | C50611 | Malignant neoplasm of axillary tail of right female breast |
| ICD-10-CM | C50612 | Malignant neoplasm of axillary tail of left female breast |
| ICD-10-CM | C50619 | Malignant neoplasm of axillary tail of unspecified female breast |
| ICD-10-CM | C50621 | Malignant neoplasm of axillary tail of right male breast |
| ICD-10-CM | C50622 | Malignant neoplasm of axillary tail of left male breast |
| ICD-10-CM | C50629 | Malignant neoplasm of axillary tail of unspecified male breast |
| ICD-10-CM | C50811 | Malignant neoplasm of overlapping sites of right female breast |
| ICD-10-CM | C50812 | Malignant neoplasm of overlapping sites of left female breast |
| ICD-10-CM | C50819 | Malignant neoplasm of overlapping sites of unspecified female breast |
| ICD-10-CM | C50821 | Malignant neoplasm of overlapping sites of right male breast |
| ICD-10-CM | C50822 | Malignant neoplasm of overlapping sites of left male breast |
| ICD-10-CM | C50829 | Malignant neoplasm of overlapping sites of unspecified male breast |
| ICD-10-CM | C50911 | Malignant neoplasm of unspecified site of right female breast |
| ICD-10-CM | C50912 | Malignant neoplasm of unspecified site of left female breast |
| ICD-10-CM | C50919 | Malignant neoplasm of unspecified site of unspecified female breast |
| ICD-10-CM | C50921 | Malignant neoplasm of unspecified site of right male breast |
| ICD-10-CM | C50922 | Malignant neoplasm of unspecified site of left male breast |
| ICD-10-CM | C50929 | Malignant neoplasm of unspecified site of unspecified male breast |
| ICD-10-CM | C510 | Malignant neoplasm of labium majus |
| ICD-10-CM | C511 | Malignant neoplasm of labium minus |
| ICD-10-CM | C512 | Malignant neoplasm of clitoris |
| ICD-10-CM | C518 | Malignant neoplasm of overlapping sites of vulva |
| ICD-10-CM | C519 | Malignant neoplasm of vulva, unspecified |
| ICD-10-CM | C52 | Malignant neoplasm of vagina |
| ICD-10-CM | C530 | Malignant neoplasm of endocervix |
| ICD-10-CM | C531 | Malignant neoplasm of exocervix |
| ICD-10-CM | C538 | Malignant neoplasm of overlapping sites of cervix uteri |
| ICD-10-CM | C539 | Malignant neoplasm of cervix uteri, unspecified |
| ICD-10-CM | C540 | Malignant neoplasm of isthmus uteri |
| ICD-10-CM | C541 | Malignant neoplasm of endometrium |
| ICD-10-CM | C542 | Malignant neoplasm of myometrium |
| ICD-10-CM | C543 | Malignant neoplasm of fundus uteri |
| ICD-10-CM | C548 | Malignant neoplasm of overlapping sites of corpus uteri |
| ICD-10-CM | C549 | Malignant neoplasm of corpus uteri, unspecified |
| ICD-10-CM | C55 | Malignant neoplasm of uterus, part unspecified |
| ICD-10-CM | C561 | Malignant neoplasm of right ovary |
| ICD-10-CM | C562 | Malignant neoplasm of left ovary |
| ICD-10-CM | C569 | Malignant neoplasm of unspecified ovary |
| ICD-10-CM | C5700 | Malignant neoplasm of unspecified fallopian tube |
| ICD-10-CM | C5701 | Malignant neoplasm of right fallopian tube |
| ICD-10-CM | C5702 | Malignant neoplasm of left fallopian tube |
| ICD-10-CM | C5710 | Malignant neoplasm of unspecified broad ligament |
| ICD-10-CM | C5711 | Malignant neoplasm of right broad ligament |
| ICD-10-CM | C5712 | Malignant neoplasm of left broad ligament |
| ICD-10-CM | C5720 | Malignant neoplasm of unspecified round ligament |
| ICD-10-CM | C5721 | Malignant neoplasm of right round ligament |
| ICD-10-CM | C5722 | Malignant neoplasm of left round ligament |
| ICD-10-CM | C573 | Malignant neoplasm of parametrium |
| ICD-10-CM | C574 | Malignant neoplasm of uterine adnexa, unspecified |
| ICD-10-CM | C577 | Malignant neoplasm of other specified female genital organs |
| ICD-10-CM | C578 | Malignant neoplasm of overlapping sites of female genital organs |
| ICD-10-CM | C579 | Malignant neoplasm of female genital organ, unspecified |
| ICD-10-CM | C58 | Malignant neoplasm of placenta |
| ICD-10-CM | C601 | Malignant neoplasm of glans penis |
| ICD-10-CM | C602 | Malignant neoplasm of body of penis |
| ICD-10-CM | C608 | Malignant neoplasm of overlapping sites of penis |
| ICD-10-CM | C609 | Malignant neoplasm of penis, unspecified |
| ICD-10-CM | C61 | Malignant neoplasm of prostate |
| ICD-10-CM | C6200 | Malignant neoplasm of unspecified undescended testis |
| ICD-10-CM | C6201 | Malignant neoplasm of undescended right testis |
| ICD-10-CM | C6202 | Malignant neoplasm of undescended left testis |
| ICD-10-CM | C6210 | Malignant neoplasm of unspecified descended testis |
| ICD-10-CM | C6211 | Malignant neoplasm of descended right testis |
| ICD-10-CM | C6212 | Malignant neoplasm of descended left testis |
| ICD-10-CM | C6290 | Malignant neoplasm of unspecified testis, unspecified whether descended or undescended |
| ICD-10-CM | C6291 | Malignant neoplasm of right testis, unspecified whether descended or undescended |
| ICD-10-CM | C6292 | Malignant neoplasm of left testis, unspecified whether descended or undescended |
| ICD-10-CM | C6300 | Malignant neoplasm of unspecified epididymis |
| ICD-10-CM | C6301 | Malignant neoplasm of right epididymis |
| ICD-10-CM | C6302 | Malignant neoplasm of left epididymis |
| ICD-10-CM | C6310 | Malignant neoplasm of unspecified spermatic cord |
| ICD-10-CM | C6311 | Malignant neoplasm of right spermatic cord |
| ICD-10-CM | C6312 | Malignant neoplasm of left spermatic cord |
| ICD-10-CM | C637 | Malignant neoplasm of other specified male genital organs |
| ICD-10-CM | C638 | Malignant neoplasm of overlapping sites of male genital organs |
| ICD-10-CM | C639 | Malignant neoplasm of male genital organ, unspecified |
| ICD-10-CM | C641 | Malignant neoplasm of right kidney, except renal pelvis |
| ICD-10-CM | C642 | Malignant neoplasm of left kidney, except renal pelvis |
| ICD-10-CM | C649 | Malignant neoplasm of unspecified kidney, except renal pelvis |
| ICD-10-CM | C651 | Malignant neoplasm of right renal pelvis |
| ICD-10-CM | C652 | Malignant neoplasm of left renal pelvis |
| ICD-10-CM | C659 | Malignant neoplasm of unspecified renal pelvis |
| ICD-10-CM | C661 | Malignant neoplasm of right ureter |
| ICD-10-CM | C662 | Malignant neoplasm of left ureter |
| ICD-10-CM | C669 | Malignant neoplasm of unspecified ureter |
| ICD-10-CM | C670 | Malignant neoplasm of trigone of bladder |
| ICD-10-CM | C671 | Malignant neoplasm of dome of bladder |
| ICD-10-CM | C672 | Malignant neoplasm of lateral wall of bladder |
| ICD-10-CM | C673 | Malignant neoplasm of anterior wall of bladder |
| ICD-10-CM | C674 | Malignant neoplasm of posterior wall of bladder |
| ICD-10-CM | C675 | Malignant neoplasm of bladder neck |
| ICD-10-CM | C676 | Malignant neoplasm of ureteric orifice |
| ICD-10-CM | C677 | Malignant neoplasm of urachus |
| ICD-10-CM | C678 | Malignant neoplasm of overlapping sites of bladder |
| ICD-10-CM | C679 | Malignant neoplasm of bladder, unspecified |
| ICD-10-CM | C680 | Malignant neoplasm of urethra |
| ICD-10-CM | C681 | Malignant neoplasm of paraurethral glands |
| ICD-10-CM | C688 | Malignant neoplasm of overlapping sites of urinary organs |
| ICD-10-CM | C689 | Malignant neoplasm of urinary organ, unspecified |
| ICD-10-CM | C6900 | Malignant neoplasm of unspecified conjunctiva |
| ICD-10-CM | C6901 | Malignant neoplasm of right conjunctiva |
| ICD-10-CM | C6902 | Malignant neoplasm of left conjunctiva |
| ICD-10-CM | C6910 | Malignant neoplasm of unspecified cornea |
| ICD-10-CM | C6911 | Malignant neoplasm of right cornea |
| ICD-10-CM | C6912 | Malignant neoplasm of left cornea |
| ICD-10-CM | C6920 | Malignant neoplasm of unspecified retina |
| ICD-10-CM | C6921 | Malignant neoplasm of right retina |
| ICD-10-CM | C6922 | Malignant neoplasm of left retina |
| ICD-10-CM | C6930 | Malignant neoplasm of unspecified choroid |
| ICD-10-CM | C6931 | Malignant neoplasm of right choroid |
| ICD-10-CM | C6932 | Malignant neoplasm of left choroid |
| ICD-10-CM | C6940 | Malignant neoplasm of unspecified ciliary body |
| ICD-10-CM | C6941 | Malignant neoplasm of right ciliary body |
| ICD-10-CM | C6942 | Malignant neoplasm of left ciliary body |
| ICD-10-CM | C6950 | Malignant neoplasm of unspecified lacrimal gland and duct |
| ICD-10-CM | C6951 | Malignant neoplasm of right lacrimal gland and duct |
| ICD-10-CM | C6952 | Malignant neoplasm of left lacrimal gland and duct |
| ICD-10-CM | C6960 | Malignant neoplasm of unspecified orbit |
| ICD-10-CM | C6961 | Malignant neoplasm of right orbit |
| ICD-10-CM | C6962 | Malignant neoplasm of left orbit |
| ICD-10-CM | C6980 | Malignant neoplasm of overlapping sites of unspecified eye and adnexa |
| ICD-10-CM | C6981 | Malignant neoplasm of overlapping sites of right eye and adnexa |
| ICD-10-CM | C6982 | Malignant neoplasm of overlapping sites of left eye and adnexa |
| ICD-10-CM | C6990 | Malignant neoplasm of unspecified site of unspecified eye |
| ICD-10-CM | C6991 | Malignant neoplasm of unspecified site of right eye |
| ICD-10-CM | C6992 | Malignant neoplasm of unspecified site of left eye |
| ICD-10-CM | C700 | Malignant neoplasm of cerebral meninges |
| ICD-10-CM | C701 | Malignant neoplasm of spinal meninges |
| ICD-10-CM | C709 | Malignant neoplasm of meninges, unspecified |
| ICD-10-CM | C710 | Malignant neoplasm of cerebrum, except lobes and ventricles |
| ICD-10-CM | C711 | Malignant neoplasm of frontal lobe |
| ICD-10-CM | C712 | Malignant neoplasm of temporal lobe |
| ICD-10-CM | C713 | Malignant neoplasm of parietal lobe |
| ICD-10-CM | C714 | Malignant neoplasm of occipital lobe |
| ICD-10-CM | C715 | Malignant neoplasm of cerebral ventricle |
| ICD-10-CM | C716 | Malignant neoplasm of cerebellum |
| ICD-10-CM | C717 | Malignant neoplasm of brain stem |
| ICD-10-CM | C718 | Malignant neoplasm of overlapping sites of brain |
| ICD-10-CM | C719 | Malignant neoplasm of brain, unspecified |
| ICD-10-CM | C720 | Malignant neoplasm of spinal cord |
| ICD-10-CM | C721 | Malignant neoplasm of cauda equina |
| ICD-10-CM | C7220 | Malignant neoplasm of unspecified olfactory nerve |
| ICD-10-CM | C7221 | Malignant neoplasm of right olfactory nerve |
| ICD-10-CM | C7222 | Malignant neoplasm of left olfactory nerve |
| ICD-10-CM | C7230 | Malignant neoplasm of unspecified optic nerve |
| ICD-10-CM | C7231 | Malignant neoplasm of right optic nerve |
| ICD-10-CM | C7232 | Malignant neoplasm of left optic nerve |
| ICD-10-CM | C7240 | Malignant neoplasm of unspecified acoustic nerve |
| ICD-10-CM | C7241 | Malignant neoplasm of right acoustic nerve |
| ICD-10-CM | C7242 | Malignant neoplasm of left acoustic nerve |
| ICD-10-CM | C7250 | Malignant neoplasm of unspecified cranial nerve |
| ICD-10-CM | C7259 | Malignant neoplasm of other cranial nerves |
| ICD-10-CM | C729 | Malignant neoplasm of central nervous system, unspecified |
| ICD-10-CM | C73 | Malignant neoplasm of thyroid gland |
| ICD-10-CM | C7400 | Malignant neoplasm of cortex of unspecified adrenal gland |
| ICD-10-CM | C7401 | Malignant neoplasm of cortex of right adrenal gland |
| ICD-10-CM | C7402 | Malignant neoplasm of cortex of left adrenal gland |
| ICD-10-CM | C7410 | Malignant neoplasm of medulla of unspecified adrenal gland |
| ICD-10-CM | C7411 | Malignant neoplasm of medulla of right adrenal gland |
| ICD-10-CM | C7412 | Malignant neoplasm of medulla of left adrenal gland |
| ICD-10-CM | C7490 | Malignant neoplasm of unspecified part of unspecified adrenal gland |
| ICD-10-CM | C7491 | Malignant neoplasm of unspecified part of right adrenal gland |
| ICD-10-CM | C7492 | Malignant neoplasm of unspecified part of left adrenal gland |
| ICD-10-CM | C750 | Malignant neoplasm of parathyroid gland |
| ICD-10-CM | C751 | Malignant neoplasm of pituitary gland |
| ICD-10-CM | C752 | Malignant neoplasm of craniopharyngeal duct |
| ICD-10-CM | C753 | Malignant neoplasm of pineal gland |
| ICD-10-CM | C754 | Malignant neoplasm of carotid body |
| ICD-10-CM | C755 | Malignant neoplasm of aortic body and other paraganglia |
| ICD-10-CM | C758 | Malignant neoplasm with pluriglandular involvement, unspecified |
| ICD-10-CM | C759 | Malignant neoplasm of endocrine gland, unspecified |
| ICD-10-CM | C760 | Malignant neoplasm of head, face and neck |
| ICD-10-CM | C761 | Malignant neoplasm of thorax |
| ICD-10-CM | C762 | Malignant neoplasm of abdomen |
| ICD-10-CM | C763 | Malignant neoplasm of pelvis |
| ICD-10-CM | C7640 | Malignant neoplasm of unspecified upper limb |
| ICD-10-CM | C7641 | Malignant neoplasm of right upper limb |
| ICD-10-CM | C7642 | Malignant neoplasm of left upper limb |
| ICD-10-CM | C7650 | Malignant neoplasm of unspecified lower limb |
| ICD-10-CM | C7651 | Malignant neoplasm of right lower limb |
| ICD-10-CM | C7652 | Malignant neoplasm of left lower limb |
| ICD-10-CM | C768 | Malignant neoplasm of other specified ill-defined sites |
| ICD-10-CM | C770 | Secondary and unspecified malignant neoplasm of lymph nodes of head, face and neck |
| ICD-10-CM | C771 | Secondary and unspecified malignant neoplasm of intrathoracic lymph nodes |
| ICD-10-CM | C772 | Secondary and unspecified malignant neoplasm of intra-abdominal lymph nodes |
| ICD-10-CM | C773 | Secondary and unspecified malignant neoplasm of axilla and upper limb lymph nodes |
| ICD-10-CM | C774 | Secondary and unspecified malignant neoplasm of inguinal and lower limb lymph nodes |
| ICD-10-CM | C775 | Secondary and unspecified malignant neoplasm of intrapelvic lymph nodes |
| ICD-10-CM | C778 | Secondary and unspecified malignant neoplasm of lymph nodes of multiple regions |
| ICD-10-CM | C779 | Secondary and unspecified malignant neoplasm of lymph node, unspecified |
| ICD-10-CM | C7800 | Secondary malignant neoplasm of unspecified lung |
| ICD-10-CM | C7801 | Secondary malignant neoplasm of right lung |
| ICD-10-CM | C7802 | Secondary malignant neoplasm of left lung |
| ICD-10-CM | C781 | Secondary malignant neoplasm of mediastinum |
| ICD-10-CM | C782 | Secondary malignant neoplasm of pleura |
| ICD-10-CM | C7830 | Secondary malignant neoplasm of unspecified respiratory organ |
| ICD-10-CM | C7839 | Secondary malignant neoplasm of other respiratory organs |
| ICD-10-CM | C784 | Secondary malignant neoplasm of small intestine |
| ICD-10-CM | C785 | Secondary malignant neoplasm of large intestine and rectum |
| ICD-10-CM | C786 | Secondary malignant neoplasm of retroperitoneum and peritoneum |
| ICD-10-CM | C787 | Secondary malignant neoplasm of liver and intrahepatic bile duct |
| ICD-10-CM | C7880 | Secondary malignant neoplasm of unspecified digestive organ |
| ICD-10-CM | C7889 | Secondary malignant neoplasm of other digestive organs |
| ICD-10-CM | C7900 | Secondary malignant neoplasm of unspecified kidney and renal pelvis |
| ICD-10-CM | C7901 | Secondary malignant neoplasm of right kidney and renal pelvis |
| ICD-10-CM | C7902 | Secondary malignant neoplasm of left kidney and renal pelvis |
| ICD-10-CM | C7910 | Secondary malignant neoplasm of unspecified urinary organs |
| ICD-10-CM | C7911 | Secondary malignant neoplasm of bladder |
| ICD-10-CM | C7919 | Secondary malignant neoplasm of other urinary organs |
| ICD-10-CM | C792 | Secondary malignant neoplasm of skin |
| ICD-10-CM | C7931 | Secondary malignant neoplasm of brain |
| ICD-10-CM | C7932 | Secondary malignant neoplasm of cerebral meninges |
| ICD-10-CM | C7940 | Secondary malignant neoplasm of unspecified part of nervous system |
| ICD-10-CM | C7949 | Secondary malignant neoplasm of other parts of nervous system |
| ICD-10-CM | C7951 | Secondary malignant neoplasm of bone |
| ICD-10-CM | C7952 | Secondary malignant neoplasm of bone marrow |
| ICD-10-CM | C7960 | Secondary malignant neoplasm of unspecified ovary |
| ICD-10-CM | C7961 | Secondary malignant neoplasm of right ovary |
| ICD-10-CM | C7962 | Secondary malignant neoplasm of left ovary |
| ICD-10-CM | C7970 | Secondary malignant neoplasm of unspecified adrenal gland |
| ICD-10-CM | C7971 | Secondary malignant neoplasm of right adrenal gland |
| ICD-10-CM | C7972 | Secondary malignant neoplasm of left adrenal gland |
| ICD-10-CM | C7981 | Secondary malignant neoplasm of breast |
| ICD-10-CM | C7982 | Secondary malignant neoplasm of genital organs |
| ICD-10-CM | C7989 | Secondary malignant neoplasm of other specified sites |
| ICD-10-CM | C799 | Secondary malignant neoplasm of unspecified site |
| ICD-10-CM | C7A00 | Malignant carcinoid tumor of unspecified site |
| ICD-10-CM | C7A010 | Malignant carcinoid tumor of the duodenum |
| ICD-10-CM | C7A011 | Malignant carcinoid tumor of the jejunum |
| ICD-10-CM | C7A012 | Malignant carcinoid tumor of the ileum |
| ICD-10-CM | C7A019 | Malignant carcinoid tumor of the small intestine, unspecified portion |
| ICD-10-CM | C7A020 | Malignant carcinoid tumor of the appendix |
| ICD-10-CM | C7A021 | Malignant carcinoid tumor of the cecum |
| ICD-10-CM | C7A022 | Malignant carcinoid tumor of the ascending colon |
| ICD-10-CM | C7A023 | Malignant carcinoid tumor of the transverse colon |
| ICD-10-CM | C7A024 | Malignant carcinoid tumor of the descending colon |
| ICD-10-CM | C7A025 | Malignant carcinoid tumor of the sigmoid colon |
| ICD-10-CM | C7A026 | Malignant carcinoid tumor of the rectum |
| ICD-10-CM | C7A029 | Malignant carcinoid tumor of the large intestine, unspecified portion |
| ICD-10-CM | C7A090 | Malignant carcinoid tumor of the bronchus and lung |
| ICD-10-CM | C7A091 | Malignant carcinoid tumor of the thymus |
| ICD-10-CM | C7A092 | Malignant carcinoid tumor of the stomach |
| ICD-10-CM | C7A093 | Malignant carcinoid tumor of the kidney |
| ICD-10-CM | C7A094 | Malignant carcinoid tumor of the foregut NOS |
| ICD-10-CM | C7A095 | Malignant carcinoid tumor of the midgut NOS |
| ICD-10-CM | C7A096 | Malignant carcinoid tumor of the hindgut NOS |
| ICD-10-CM | C7A098 | Malignant carcinoid tumors of other sites |
| ICD-10-CM | C7A1 | Malignant poorly differentiated neuroendocrine tumors |
| ICD-10-CM | C7A8 | Other malignant neuroendocrine tumors |
| ICD-10-CM | C7B00 | Secondary carcinoid tumors, unspecified site |
| ICD-10-CM | C7B01 | Secondary carcinoid tumors of distant lymph nodes |
| ICD-10-CM | C7B02 | Secondary carcinoid tumors of liver |
| ICD-10-CM | C7B03 | Secondary carcinoid tumors of bone |
| ICD-10-CM | C7B04 | Secondary carcinoid tumors of peritoneum |
| ICD-10-CM | C7B09 | Secondary carcinoid tumors of other sites |
| ICD-10-CM | C7B1 | Secondary Merkel cell carcinoma |
| ICD-10-CM | C7B8 | Other secondary neuroendocrine tumors |
| ICD-10-CM | C800 | Disseminated malignant neoplasm, unspecified |
| ICD-10-CM | C801 | Malignant (primary) neoplasm, unspecified |
| ICD-10-CM | C802 | Malignant neoplasm associated with transplanted organ |
| ICD-10-CM | C8100 | Nodular lymphocyte predominant Hodgkin lymphoma, unspecified site |
| ICD-10-CM | C8101 | Nodular lymphocyte predominant Hodgkin lymphoma, lymph nodes of head, face, and neck |
| ICD-10-CM | C8102 | Nodular lymphocyte predominant Hodgkin lymphoma, intrathoracic lymph nodes |
| ICD-10-CM | C8103 | Nodular lymphocyte predominant Hodgkin lymphoma, intra-abdominal lymph nodes |
| ICD-10-CM | C8104 | Nodular lymphocyte predominant Hodgkin lymphoma, lymph nodes of axilla and upper limb |
| ICD-10-CM | C8105 | Nodular lymphocyte predominant Hodgkin lymphoma, lymph nodes of inguinal region and lower limb |
| ICD-10-CM | C8106 | Nodular lymphocyte predominant Hodgkin lymphoma, intrapelvic lymph nodes |
| ICD-10-CM | C8107 | Nodular lymphocyte predominant Hodgkin lymphoma, spleen |
| ICD-10-CM | C8108 | Nodular lymphocyte predominant Hodgkin lymphoma, lymph nodes of multiple sites |
| ICD-10-CM | C8109 | Nodular lymphocyte predominant Hodgkin lymphoma, extranodal and solid organ sites |
| ICD-10-CM | C8110 | Nodular sclerosis classical Hodgkin lymphoma, unspecified site |
| ICD-10-CM | C8111 | Nodular sclerosis classical Hodgkin lymphoma, lymph nodes of head, face, and neck |
| ICD-10-CM | C8112 | Nodular sclerosis classical Hodgkin lymphoma, intrathoracic lymph nodes |
| ICD-10-CM | C8113 | Nodular sclerosis classical Hodgkin lymphoma, intra-abdominal lymph nodes |
| ICD-10-CM | C8114 | Nodular sclerosis classical Hodgkin lymphoma, lymph nodes of axilla and upper limb |
| ICD-10-CM | C8115 | Nodular sclerosis classical Hodgkin lymphoma, lymph nodes of inguinal region and lower limb |
| ICD-10-CM | C8116 | Nodular sclerosis classical Hodgkin lymphoma, intrapelvic lymph nodes |
| ICD-10-CM | C8117 | Nodular sclerosis classical Hodgkin lymphoma, spleen |
| ICD-10-CM | C8118 | Nodular sclerosis classical Hodgkin lymphoma, lymph nodes of multiple sites |
| ICD-10-CM | C8119 | Nodular sclerosis classical Hodgkin lymphoma, extranodal and solid organ sites |
| ICD-10-CM | C8120 | Mixed cellularity classical Hodgkin lymphoma, unspecified site |
| ICD-10-CM | C8121 | Mixed cellularity classical Hodgkin lymphoma, lymph nodes of head, face, and neck |
| ICD-10-CM | C8122 | Mixed cellularity classical Hodgkin lymphoma, intrathoracic lymph nodes |
| ICD-10-CM | C8123 | Mixed cellularity classical Hodgkin lymphoma, intra-abdominal lymph nodes |
| ICD-10-CM | C8124 | Mixed cellularity classical Hodgkin lymphoma, lymph nodes of axilla and upper limb |
| ICD-10-CM | C8125 | Mixed cellularity classical Hodgkin lymphoma, lymph nodes of inguinal region and lower limb |
| ICD-10-CM | C8126 | Mixed cellularity classical Hodgkin lymphoma, intrapelvic lymph nodes |
| ICD-10-CM | C8127 | Mixed cellularity classical Hodgkin lymphoma, spleen |
| ICD-10-CM | C8128 | Mixed cellularity classical Hodgkin lymphoma, lymph nodes of multiple sites |
| ICD-10-CM | C8129 | Mixed cellularity classical Hodgkin lymphoma, extranodal and solid organ sites |
| ICD-10-CM | C8130 | Lymphocyte depleted classical Hodgkin lymphoma, unspecified site |
| ICD-10-CM | C8131 | Lymphocyte depleted classical Hodgkin lymphoma, lymph nodes of head, face, and neck |
| ICD-10-CM | C8132 | Lymphocyte depleted classical Hodgkin lymphoma, intrathoracic lymph nodes |
| ICD-10-CM | C8133 | Lymphocyte depleted classical Hodgkin lymphoma, intra-abdominal lymph nodes |
| ICD-10-CM | C8134 | Lymphocyte depleted classical Hodgkin lymphoma, lymph nodes of axilla and upper limb |
| ICD-10-CM | C8135 | Lymphocyte depleted classical Hodgkin lymphoma, lymph nodes of inguinal region and lower limb |
| ICD-10-CM | C8136 | Lymphocyte depleted classical Hodgkin lymphoma, intrapelvic lymph nodes |
| ICD-10-CM | C8137 | Lymphocyte depleted classical Hodgkin lymphoma, spleen |
| ICD-10-CM | C8138 | Lymphocyte depleted classical Hodgkin lymphoma, lymph nodes of multiple sites |
| ICD-10-CM | C8139 | Lymphocyte depleted classical Hodgkin lymphoma, extranodal and solid organ sites |
| ICD-10-CM | C8140 | Lymphocyte-rich classical Hodgkin lymphoma, unspecified site |
| ICD-10-CM | C8141 | Lymphocyte-rich classical Hodgkin lymphoma, lymph nodes of head, face, and neck |
| ICD-10-CM | C8142 | Lymphocyte-rich classical Hodgkin lymphoma, intrathoracic lymph nodes |
| ICD-10-CM | C8143 | Lymphocyte-rich classical Hodgkin lymphoma, intra-abdominal lymph nodes |
| ICD-10-CM | C8144 | Lymphocyte-rich classical Hodgkin lymphoma, lymph nodes of axilla and upper limb |
| ICD-10-CM | C8145 | Lymphocyte-rich classical Hodgkin lymphoma, lymph nodes of inguinal region and lower limb |
| ICD-10-CM | C8146 | Lymphocyte-rich classical Hodgkin lymphoma, intrapelvic lymph nodes |
| ICD-10-CM | C8147 | Lymphocyte-rich classical Hodgkin lymphoma, spleen |
| ICD-10-CM | C8148 | Lymphocyte-rich classical Hodgkin lymphoma, lymph nodes of multiple sites |
| ICD-10-CM | C8149 | Lymphocyte-rich classical Hodgkin lymphoma, extranodal and solid organ sites |
| ICD-10-CM | C8170 | Other classical Hodgkin lymphoma, unspecified site |
| ICD-10-CM | C8171 | Other classical Hodgkin lymphoma, lymph nodes of head, face, and neck |
| ICD-10-CM | C8172 | Other classical Hodgkin lymphoma, intrathoracic lymph nodes |
| ICD-10-CM | C8173 | Other classical Hodgkin lymphoma, intra-abdominal lymph nodes |
| ICD-10-CM | C8174 | Other classical Hodgkin lymphoma, lymph nodes of axilla and upper limb |
| ICD-10-CM | C8175 | Other classical Hodgkin lymphoma, lymph nodes of inguinal region and lower limb |
| ICD-10-CM | C8176 | Other classical Hodgkin lymphoma, intrapelvic lymph nodes |
| ICD-10-CM | C8177 | Other classical Hodgkin lymphoma, spleen |
| ICD-10-CM | C8178 | Other classical Hodgkin lymphoma, lymph nodes of multiple sites |
| ICD-10-CM | C8179 | Other classical Hodgkin lymphoma, extranodal and solid organ sites |
| ICD-10-CM | C8190 | Hodgkin lymphoma, unspecified, unspecified site |
| ICD-10-CM | C8191 | Hodgkin lymphoma, unspecified, lymph nodes of head, face, and neck |
| ICD-10-CM | C8192 | Hodgkin lymphoma, unspecified, intrathoracic lymph nodes |
| ICD-10-CM | C8193 | Hodgkin lymphoma, unspecified, intra-abdominal lymph nodes |
| ICD-10-CM | C8194 | Hodgkin lymphoma, unspecified, lymph nodes of axilla and upper limb |
| ICD-10-CM | C8195 | Hodgkin lymphoma, unspecified, lymph nodes of inguinal region and lower limb |
| ICD-10-CM | C8196 | Hodgkin lymphoma, unspecified, intrapelvic lymph nodes |
| ICD-10-CM | C8197 | Hodgkin lymphoma, unspecified, spleen |
| ICD-10-CM | C8198 | Hodgkin lymphoma, unspecified, lymph nodes of multiple sites |
| ICD-10-CM | C8199 | Hodgkin lymphoma, unspecified, extranodal and solid organ sites |
| ICD-10-CM | C8200 | Follicular lymphoma grade I, unspecified site |
| ICD-10-CM | C8201 | Follicular lymphoma grade I, lymph nodes of head, face, and neck |
| ICD-10-CM | C8202 | Follicular lymphoma grade I, intrathoracic lymph nodes |
| ICD-10-CM | C8203 | Follicular lymphoma grade I, intra-abdominal lymph nodes |
| ICD-10-CM | C8204 | Follicular lymphoma grade I, lymph nodes of axilla and upper limb |
| ICD-10-CM | C8205 | Follicular lymphoma grade I, lymph nodes of inguinal region and lower limb |
| ICD-10-CM | C8206 | Follicular lymphoma grade I, intrapelvic lymph nodes |
| ICD-10-CM | C8207 | Follicular lymphoma grade I, spleen |
| ICD-10-CM | C8208 | Follicular lymphoma grade I, lymph nodes of multiple sites |
| ICD-10-CM | C8209 | Follicular lymphoma grade I, extranodal and solid organ sites |
| ICD-10-CM | C8210 | Follicular lymphoma grade II, unspecified site |
| ICD-10-CM | C8211 | Follicular lymphoma grade II, lymph nodes of head, face, and neck |
| ICD-10-CM | C8212 | Follicular lymphoma grade II, intrathoracic lymph nodes |
| ICD-10-CM | C8213 | Follicular lymphoma grade II, intra-abdominal lymph nodes |
| ICD-10-CM | C8214 | Follicular lymphoma grade II, lymph nodes of axilla and upper limb |
| ICD-10-CM | C8215 | Follicular lymphoma grade II, lymph nodes of inguinal region and lower limb |
| ICD-10-CM | C8216 | Follicular lymphoma grade II, intrapelvic lymph nodes |
| ICD-10-CM | C8217 | Follicular lymphoma grade II, spleen |
| ICD-10-CM | C8218 | Follicular lymphoma grade II, lymph nodes of multiple sites |
| ICD-10-CM | C8219 | Follicular lymphoma grade II, extranodal and solid organ sites |
| ICD-10-CM | C8220 | Follicular lymphoma grade III, unspecified, unspecified site |
| ICD-10-CM | C8221 | Follicular lymphoma grade III, unspecified, lymph nodes of head, face, and neck |
| ICD-10-CM | C8222 | Follicular lymphoma grade III, unspecified, intrathoracic lymph nodes |
| ICD-10-CM | C8223 | Follicular lymphoma grade III, unspecified, intra-abdominal lymph nodes |
| ICD-10-CM | C8224 | Follicular lymphoma grade III, unspecified, lymph nodes of axilla and upper limb |
| ICD-10-CM | C8225 | Follicular lymphoma grade III, unspecified, lymph nodes of inguinal region and lower limb |
| ICD-10-CM | C8226 | Follicular lymphoma grade III, unspecified, intrapelvic lymph nodes |
| ICD-10-CM | C8227 | Follicular lymphoma grade III, unspecified, spleen |
| ICD-10-CM | C8228 | Follicular lymphoma grade III, unspecified, lymph nodes of multiple sites |
| ICD-10-CM | C8229 | Follicular lymphoma grade III, unspecified, extranodal and solid organ sites |
| ICD-10-CM | C8230 | Follicular lymphoma grade IIIa, unspecified site |
| ICD-10-CM | C8231 | Follicular lymphoma grade IIIa, lymph nodes of head, face, and neck |
| ICD-10-CM | C8232 | Follicular lymphoma grade IIIa, intrathoracic lymph nodes |
| ICD-10-CM | C8233 | Follicular lymphoma grade IIIa, intra-abdominal lymph nodes |
| ICD-10-CM | C8234 | Follicular lymphoma grade IIIa, lymph nodes of axilla and upper limb |
| ICD-10-CM | C8235 | Follicular lymphoma grade IIIa, lymph nodes of inguinal region and lower limb |
| ICD-10-CM | C8236 | Follicular lymphoma grade IIIa, intrapelvic lymph nodes |
| ICD-10-CM | C8237 | Follicular lymphoma grade IIIa, spleen |
| ICD-10-CM | C8238 | Follicular lymphoma grade IIIa, lymph nodes of multiple sites |
| ICD-10-CM | C8239 | Follicular lymphoma grade IIIa, extranodal and solid organ sites |
| ICD-10-CM | C8240 | Follicular lymphoma grade IIIb, unspecified site |
| ICD-10-CM | C8241 | Follicular lymphoma grade IIIb, lymph nodes of head, face, and neck |
| ICD-10-CM | C8242 | Follicular lymphoma grade IIIb, intrathoracic lymph nodes |
| ICD-10-CM | C8243 | Follicular lymphoma grade IIIb, intra-abdominal lymph nodes |
| ICD-10-CM | C8244 | Follicular lymphoma grade IIIb, lymph nodes of axilla and upper limb |
| ICD-10-CM | C8245 | Follicular lymphoma grade IIIb, lymph nodes of inguinal region and lower limb |
| ICD-10-CM | C8246 | Follicular lymphoma grade IIIb, intrapelvic lymph nodes |
| ICD-10-CM | C8247 | Follicular lymphoma grade IIIb, spleen |
| ICD-10-CM | C8248 | Follicular lymphoma grade IIIb, lymph nodes of multiple sites |
| ICD-10-CM | C8249 | Follicular lymphoma grade IIIb, extranodal and solid organ sites |
| ICD-10-CM | C8250 | Diffuse follicle center lymphoma, unspecified site |
| ICD-10-CM | C8251 | Diffuse follicle center lymphoma, lymph nodes of head, face, and neck |
| ICD-10-CM | C8252 | Diffuse follicle center lymphoma, intrathoracic lymph nodes |
| ICD-10-CM | C8253 | Diffuse follicle center lymphoma, intra-abdominal lymph nodes |
| ICD-10-CM | C8254 | Diffuse follicle center lymphoma, lymph nodes of axilla and upper limb |
| ICD-10-CM | C8255 | Diffuse follicle center lymphoma, lymph nodes of inguinal region and lower limb |
| ICD-10-CM | C8256 | Diffuse follicle center lymphoma, intrapelvic lymph nodes |
| ICD-10-CM | C8257 | Diffuse follicle center lymphoma, spleen |
| ICD-10-CM | C8258 | Diffuse follicle center lymphoma, lymph nodes of multiple sites |
| ICD-10-CM | C8259 | Diffuse follicle center lymphoma, extranodal and solid organ sites |
| ICD-10-CM | C8260 | Cutaneous follicle center lymphoma, unspecified site |
| ICD-10-CM | C8261 | Cutaneous follicle center lymphoma, lymph nodes of head, face, and neck |
| ICD-10-CM | C8262 | Cutaneous follicle center lymphoma, intrathoracic lymph nodes |
| ICD-10-CM | C8263 | Cutaneous follicle center lymphoma, intra-abdominal lymph nodes |
| ICD-10-CM | C8264 | Cutaneous follicle center lymphoma, lymph nodes of axilla and upper limb |
| ICD-10-CM | C8265 | Cutaneous follicle center lymphoma, lymph nodes of inguinal region and lower limb |
| ICD-10-CM | C8266 | Cutaneous follicle center lymphoma, intrapelvic lymph nodes |
| ICD-10-CM | C8267 | Cutaneous follicle center lymphoma, spleen |
| ICD-10-CM | C8268 | Cutaneous follicle center lymphoma, lymph nodes of multiple sites |
| ICD-10-CM | C8269 | Cutaneous follicle center lymphoma, extranodal and solid organ sites |
| ICD-10-CM | C8280 | Other types of follicular lymphoma, unspecified site |
| ICD-10-CM | C8281 | Other types of follicular lymphoma, lymph nodes of head, face, and neck |
| ICD-10-CM | C8282 | Other types of follicular lymphoma, intrathoracic lymph nodes |
| ICD-10-CM | C8283 | Other types of follicular lymphoma, intra-abdominal lymph nodes |
| ICD-10-CM | C8284 | Other types of follicular lymphoma, lymph nodes of axilla and upper limb |
| ICD-10-CM | C8285 | Other types of follicular lymphoma, lymph nodes of inguinal region and lower limb |
| ICD-10-CM | C8286 | Other types of follicular lymphoma, intrapelvic lymph nodes |
| ICD-10-CM | C8287 | Other types of follicular lymphoma, spleen |
| ICD-10-CM | C8288 | Other types of follicular lymphoma, lymph nodes of multiple sites |
| ICD-10-CM | C8289 | Other types of follicular lymphoma, extranodal and solid organ sites |
| ICD-10-CM | C8290 | Follicular lymphoma, unspecified, unspecified site |
| ICD-10-CM | C8291 | Follicular lymphoma, unspecified, lymph nodes of head, face, and neck |
| ICD-10-CM | C8292 | Follicular lymphoma, unspecified, intrathoracic lymph nodes |
| ICD-10-CM | C8293 | Follicular lymphoma, unspecified, intra-abdominal lymph nodes |
| ICD-10-CM | C8294 | Follicular lymphoma, unspecified, lymph nodes of axilla and upper limb |
| ICD-10-CM | C8295 | Follicular lymphoma, unspecified, lymph nodes of inguinal region and lower limb |
| ICD-10-CM | C8296 | Follicular lymphoma, unspecified, intrapelvic lymph nodes |
| ICD-10-CM | C8297 | Follicular lymphoma, unspecified, spleen |
| ICD-10-CM | C8298 | Follicular lymphoma, unspecified, lymph nodes of multiple sites |
| ICD-10-CM | C8299 | Follicular lymphoma, unspecified, extranodal and solid organ sites |
| ICD-10-CM | C8300 | Small cell B-cell lymphoma, unspecified site |
| ICD-10-CM | C8301 | Small cell B-cell lymphoma, lymph nodes of head, face, and neck |
| ICD-10-CM | C8302 | Small cell B-cell lymphoma, intrathoracic lymph nodes |
| ICD-10-CM | C8303 | Small cell B-cell lymphoma, intra-abdominal lymph nodes |
| ICD-10-CM | C8304 | Small cell B-cell lymphoma, lymph nodes of axilla and upper limb |
| ICD-10-CM | C8305 | Small cell B-cell lymphoma, lymph nodes of inguinal region and lower limb |
| ICD-10-CM | C8306 | Small cell B-cell lymphoma, intrapelvic lymph nodes |
| ICD-10-CM | C8307 | Small cell B-cell lymphoma, spleen |
| ICD-10-CM | C8308 | Small cell B-cell lymphoma, lymph nodes of multiple sites |
| ICD-10-CM | C8309 | Small cell B-cell lymphoma, extranodal and solid organ sites |
| ICD-10-CM | C8310 | Mantle cell lymphoma, unspecified site |
| ICD-10-CM | C8311 | Mantle cell lymphoma, lymph nodes of head, face, and neck |
| ICD-10-CM | C8312 | Mantle cell lymphoma, intrathoracic lymph nodes |
| ICD-10-CM | C8313 | Mantle cell lymphoma, intra-abdominal lymph nodes |
| ICD-10-CM | C8314 | Mantle cell lymphoma, lymph nodes of axilla and upper limb |
| ICD-10-CM | C8315 | Mantle cell lymphoma, lymph nodes of inguinal region and lower limb |
| ICD-10-CM | C8316 | Mantle cell lymphoma, intrapelvic lymph nodes |
| ICD-10-CM | C8317 | Mantle cell lymphoma, spleen |
| ICD-10-CM | C8318 | Mantle cell lymphoma, lymph nodes of multiple sites |
| ICD-10-CM | C8319 | Mantle cell lymphoma, extranodal and solid organ sites |
| ICD-10-CM | C8330 | Diffuse large B-cell lymphoma, unspecified site |
| ICD-10-CM | C8331 | Diffuse large B-cell lymphoma, lymph nodes of head, face, and neck |
| ICD-10-CM | C8332 | Diffuse large B-cell lymphoma, intrathoracic lymph nodes |
| ICD-10-CM | C8333 | Diffuse large B-cell lymphoma, intra-abdominal lymph nodes |
| ICD-10-CM | C8334 | Diffuse large B-cell lymphoma, lymph nodes of axilla and upper limb |
| ICD-10-CM | C8335 | Diffuse large B-cell lymphoma, lymph nodes of inguinal region and lower limb |
| ICD-10-CM | C8336 | Diffuse large B-cell lymphoma, intrapelvic lymph nodes |
| ICD-10-CM | C8337 | Diffuse large B-cell lymphoma, spleen |
| ICD-10-CM | C8338 | Diffuse large B-cell lymphoma, lymph nodes of multiple sites |
| ICD-10-CM | C8339 | Diffuse large B-cell lymphoma, extranodal and solid organ sites |
| ICD-10-CM | C8350 | Lymphoblastic (diffuse) lymphoma, unspecified site |
| ICD-10-CM | C8351 | Lymphoblastic (diffuse) lymphoma, lymph nodes of head, face, and neck |
| ICD-10-CM | C8352 | Lymphoblastic (diffuse) lymphoma, intrathoracic lymph nodes |
| ICD-10-CM | C8353 | Lymphoblastic (diffuse) lymphoma, intra-abdominal lymph nodes |
| ICD-10-CM | C8354 | Lymphoblastic (diffuse) lymphoma, lymph nodes of axilla and upper limb |
| ICD-10-CM | C8355 | Lymphoblastic (diffuse) lymphoma, lymph nodes of inguinal region and lower limb |
| ICD-10-CM | C8356 | Lymphoblastic (diffuse) lymphoma, intrapelvic lymph nodes |
| ICD-10-CM | C8357 | Lymphoblastic (diffuse) lymphoma, spleen |
| ICD-10-CM | C8358 | Lymphoblastic (diffuse) lymphoma, lymph nodes of multiple sites |
| ICD-10-CM | C8359 | Lymphoblastic (diffuse) lymphoma, extranodal and solid organ sites |
| ICD-10-CM | C8370 | Burkitt lymphoma, unspecified site |
| ICD-10-CM | C8371 | Burkitt lymphoma, lymph nodes of head, face, and neck |
| ICD-10-CM | C8372 | Burkitt lymphoma, intrathoracic lymph nodes |
| ICD-10-CM | C8373 | Burkitt lymphoma, intra-abdominal lymph nodes |
| ICD-10-CM | C8374 | Burkitt lymphoma, lymph nodes of axilla and upper limb |
| ICD-10-CM | C8375 | Burkitt lymphoma, lymph nodes of inguinal region and lower limb |
| ICD-10-CM | C8376 | Burkitt lymphoma, intrapelvic lymph nodes |
| ICD-10-CM | C8377 | Burkitt lymphoma, spleen |
| ICD-10-CM | C8378 | Burkitt lymphoma, lymph nodes of multiple sites |
| ICD-10-CM | C8379 | Burkitt lymphoma, extranodal and solid organ sites |
| ICD-10-CM | C8380 | Other non-follicular lymphoma, unspecified site |
| ICD-10-CM | C8381 | Other non-follicular lymphoma, lymph nodes of head, face, and neck |
| ICD-10-CM | C8382 | Other non-follicular lymphoma, intrathoracic lymph nodes |
| ICD-10-CM | C8383 | Other non-follicular lymphoma, intra-abdominal lymph nodes |
| ICD-10-CM | C8384 | Other non-follicular lymphoma, lymph nodes of axilla and upper limb |
| ICD-10-CM | C8385 | Other non-follicular lymphoma, lymph nodes of inguinal region and lower limb |
| ICD-10-CM | C8386 | Other non-follicular lymphoma, intrapelvic lymph nodes |
| ICD-10-CM | C8387 | Other non-follicular lymphoma, spleen |
| ICD-10-CM | C8388 | Other non-follicular lymphoma, lymph nodes of multiple sites |
| ICD-10-CM | C8389 | Other non-follicular lymphoma, extranodal and solid organ sites |
| ICD-10-CM | C8390 | Non-follicular (diffuse) lymphoma, unspecified, unspecified site |
| ICD-10-CM | C8391 | Non-follicular (diffuse) lymphoma, unspecified, lymph nodes of head, face, and neck |
| ICD-10-CM | C8392 | Non-follicular (diffuse) lymphoma, unspecified, intrathoracic lymph nodes |
| ICD-10-CM | C8393 | Non-follicular (diffuse) lymphoma, unspecified, intra-abdominal lymph nodes |
| ICD-10-CM | C8394 | Non-follicular (diffuse) lymphoma, unspecified, lymph nodes of axilla and upper limb |
| ICD-10-CM | C8395 | Non-follicular (diffuse) lymphoma, unspecified, lymph nodes of inguinal region and lower limb |
| ICD-10-CM | C8396 | Non-follicular (diffuse) lymphoma, unspecified, intrapelvic lymph nodes |
| ICD-10-CM | C8397 | Non-follicular (diffuse) lymphoma, unspecified, spleen |
| ICD-10-CM | C8398 | Non-follicular (diffuse) lymphoma, unspecified, lymph nodes of multiple sites |
| ICD-10-CM | C8399 | Non-follicular (diffuse) lymphoma, unspecified, extranodal and solid organ sites |
| ICD-10-CM | C8400 | Mycosis fungoides, unspecified site |
| ICD-10-CM | C8401 | Mycosis fungoides, lymph nodes of head, face, and neck |
| ICD-10-CM | C8402 | Mycosis fungoides, intrathoracic lymph nodes |
| ICD-10-CM | C8403 | Mycosis fungoides, intra-abdominal lymph nodes |
| ICD-10-CM | C8404 | Mycosis fungoides, lymph nodes of axilla and upper limb |
| ICD-10-CM | C8405 | Mycosis fungoides, lymph nodes of inguinal region and lower limb |
| ICD-10-CM | C8406 | Mycosis fungoides, intrapelvic lymph nodes |
| ICD-10-CM | C8407 | Mycosis fungoides, spleen |
| ICD-10-CM | C8408 | Mycosis fungoides, lymph nodes of multiple sites |
| ICD-10-CM | C8409 | Mycosis fungoides, extranodal and solid organ sites |
| ICD-10-CM | C8410 | Sezary disease, unspecified site |
| ICD-10-CM | C8411 | Sezary disease, lymph nodes of head, face, and neck |
| ICD-10-CM | C8412 | Sezary disease, intrathoracic lymph nodes |
| ICD-10-CM | C8413 | Sezary disease, intra-abdominal lymph nodes |
| ICD-10-CM | C8414 | Sezary disease, lymph nodes of axilla and upper limb |
| ICD-10-CM | C8415 | Sezary disease, lymph nodes of inguinal region and lower limb |
| ICD-10-CM | C8416 | Sezary disease, intrapelvic lymph nodes |
| ICD-10-CM | C8417 | Sezary disease, spleen |
| ICD-10-CM | C8418 | Sezary disease, lymph nodes of multiple sites |
| ICD-10-CM | C8419 | Sezary disease, extranodal and solid organ sites |
| ICD-10-CM | C8440 | Peripheral T-cell lymphoma, not classified, unspecified site |
| ICD-10-CM | C8441 | Peripheral T-cell lymphoma, not classified, lymph nodes of head, face, and neck |
| ICD-10-CM | C8442 | Peripheral T-cell lymphoma, not classified, intrathoracic lymph nodes |
| ICD-10-CM | C8443 | Peripheral T-cell lymphoma, not classified, intra-abdominal lymph nodes |
| ICD-10-CM | C8444 | Peripheral T-cell lymphoma, not classified, lymph nodes of axilla and upper limb |
| ICD-10-CM | C8445 | Peripheral T-cell lymphoma, not classified, lymph nodes of inguinal region and lower limb |
| ICD-10-CM | C8446 | Peripheral T-cell lymphoma, not classified, intrapelvic lymph nodes |
| ICD-10-CM | C8447 | Peripheral T-cell lymphoma, not classified, spleen |
| ICD-10-CM | C8448 | Peripheral T-cell lymphoma, not classified, lymph nodes of multiple sites |
| ICD-10-CM | C8449 | Peripheral T-cell lymphoma, not classified, extranodal and solid organ sites |
| ICD-10-CM | C8460 | Anaplastic large cell lymphoma, ALK-positive, unspecified site |
| ICD-10-CM | C8461 | Anaplastic large cell lymphoma, ALK-positive, lymph nodes of head, face, and neck |
| ICD-10-CM | C8462 | Anaplastic large cell lymphoma, ALK-positive, intrathoracic lymph nodes |
| ICD-10-CM | C8463 | Anaplastic large cell lymphoma, ALK-positive, intra-abdominal lymph nodes |
| ICD-10-CM | C8464 | Anaplastic large cell lymphoma, ALK-positive, lymph nodes of axilla and upper limb |
| ICD-10-CM | C8465 | Anaplastic large cell lymphoma, ALK-positive, lymph nodes of inguinal region and lower limb |
| ICD-10-CM | C8466 | Anaplastic large cell lymphoma, ALK-positive, intrapelvic lymph nodes |
| ICD-10-CM | C8467 | Anaplastic large cell lymphoma, ALK-positive, spleen |
| ICD-10-CM | C8468 | Anaplastic large cell lymphoma, ALK-positive, lymph nodes of multiple sites |
| ICD-10-CM | C8469 | Anaplastic large cell lymphoma, ALK-positive, extranodal and solid organ sites |
| ICD-10-CM | C8470 | Anaplastic large cell lymphoma, ALK-negative, unspecified site |
| ICD-10-CM | C8471 | Anaplastic large cell lymphoma, ALK-negative, lymph nodes of head, face, and neck |
| ICD-10-CM | C8472 | Anaplastic large cell lymphoma, ALK-negative, intrathoracic lymph nodes |
| ICD-10-CM | C8473 | Anaplastic large cell lymphoma, ALK-negative, intra-abdominal lymph nodes |
| ICD-10-CM | C8474 | Anaplastic large cell lymphoma, ALK-negative, lymph nodes of axilla and upper limb |
| ICD-10-CM | C8475 | Anaplastic large cell lymphoma, ALK-negative, lymph nodes of inguinal region and lower limb |
| ICD-10-CM | C8476 | Anaplastic large cell lymphoma, ALK-negative, intrapelvic lymph nodes |
| ICD-10-CM | C8477 | Anaplastic large cell lymphoma, ALK-negative, spleen |
| ICD-10-CM | C8478 | Anaplastic large cell lymphoma, ALK-negative, lymph nodes of multiple sites |
| ICD-10-CM | C8479 | Anaplastic large cell lymphoma, ALK-negative, extranodal and solid organ sites |
| ICD-10-CM | C8490 | Mature T/NK-cell lymphomas, unspecified, unspecified site |
| ICD-10-CM | C8491 | Mature T/NK-cell lymphomas, unspecified, lymph nodes of head, face, and neck |
| ICD-10-CM | C8492 | Mature T/NK-cell lymphomas, unspecified, intrathoracic lymph nodes |
| ICD-10-CM | C8493 | Mature T/NK-cell lymphomas, unspecified, intra-abdominal lymph nodes |
| ICD-10-CM | C8494 | Mature T/NK-cell lymphomas, unspecified, lymph nodes of axilla and upper limb |
| ICD-10-CM | C8495 | Mature T/NK-cell lymphomas, unspecified, lymph nodes of inguinal region and lower limb |
| ICD-10-CM | C8496 | Mature T/NK-cell lymphomas, unspecified, intrapelvic lymph nodes |
| ICD-10-CM | C8497 | Mature T/NK-cell lymphomas, unspecified, spleen |
| ICD-10-CM | C8498 | Mature T/NK-cell lymphomas, unspecified, lymph nodes of multiple sites |
| ICD-10-CM | C8499 | Mature T/NK-cell lymphomas, unspecified, extranodal and solid organ sites |
| ICD-10-CM | C84A0 | Cutaneous T-cell lymphoma, unspecified, unspecified site |
| ICD-10-CM | C84A1 | Cutaneous T-cell lymphoma, unspecified lymph nodes of head, face, and neck |
| ICD-10-CM | C84A2 | Cutaneous T-cell lymphoma, unspecified, intrathoracic lymph nodes |
| ICD-10-CM | C84A3 | Cutaneous T-cell lymphoma, unspecified, intra-abdominal lymph nodes |
| ICD-10-CM | C84A4 | Cutaneous T-cell lymphoma, unspecified, lymph nodes of axilla and upper limb |
| ICD-10-CM | C84A5 | Cutaneous T-cell lymphoma, unspecified, lymph nodes of inguinal region and lower limb |
| ICD-10-CM | C84A6 | Cutaneous T-cell lymphoma, unspecified, intrapelvic lymph nodes |
| ICD-10-CM | C84A7 | Cutaneous T-cell lymphoma, unspecified, spleen |
| ICD-10-CM | C84A8 | Cutaneous T-cell lymphoma, unspecified, lymph nodes of multiple sites |
| ICD-10-CM | C84A9 | Cutaneous T-cell lymphoma, unspecified, extranodal and solid organ sites |
| ICD-10-CM | C84Z0 | Other mature T/NK-cell lymphomas, unspecified site |
| ICD-10-CM | C84Z1 | Other mature T/NK-cell lymphomas, lymph nodes of head, face, and neck |
| ICD-10-CM | C84Z2 | Other mature T/NK-cell lymphomas, intrathoracic lymph nodes |
| ICD-10-CM | C84Z3 | Other mature T/NK-cell lymphomas, intra-abdominal lymph nodes |
| ICD-10-CM | C84Z4 | Other mature T/NK-cell lymphomas, lymph nodes of axilla and upper limb |
| ICD-10-CM | C84Z5 | Other mature T/NK-cell lymphomas, lymph nodes of inguinal region and lower limb |
| ICD-10-CM | C84Z6 | Other mature T/NK-cell lymphomas, intrapelvic lymph nodes |
| ICD-10-CM | C84Z7 | Other mature T/NK-cell lymphomas, spleen |
| ICD-10-CM | C84Z8 | Other mature T/NK-cell lymphomas, lymph nodes of multiple sites |
| ICD-10-CM | C84Z9 | Other mature T/NK-cell lymphomas, extranodal and solid organ sites |
| ICD-10-CM | C8510 | Unspecified B-cell lymphoma, unspecified site |
| ICD-10-CM | C8511 | Unspecified B-cell lymphoma, lymph nodes of head, face, and neck |
| ICD-10-CM | C8512 | Unspecified B-cell lymphoma, intrathoracic lymph nodes |
| ICD-10-CM | C8513 | Unspecified B-cell lymphoma, intra-abdominal lymph nodes |
| ICD-10-CM | C8514 | Unspecified B-cell lymphoma, lymph nodes of axilla and upper limb |
| ICD-10-CM | C8515 | Unspecified B-cell lymphoma, lymph nodes of inguinal region and lower limb |
| ICD-10-CM | C8516 | Unspecified B-cell lymphoma, intrapelvic lymph nodes |
| ICD-10-CM | C8517 | Unspecified B-cell lymphoma, spleen |
| ICD-10-CM | C8518 | Unspecified B-cell lymphoma, lymph nodes of multiple sites |
| ICD-10-CM | C8519 | Unspecified B-cell lymphoma, extranodal and solid organ sites |
| ICD-10-CM | C8520 | Mediastinal (thymic) large B-cell lymphoma, unspecified site |
| ICD-10-CM | C8521 | Mediastinal (thymic) large B-cell lymphoma, lymph nodes of head, face, and neck |
| ICD-10-CM | C8522 | Mediastinal (thymic) large B-cell lymphoma, intrathoracic lymph nodes |
| ICD-10-CM | C8523 | Mediastinal (thymic) large B-cell lymphoma, intra-abdominal lymph nodes |
| ICD-10-CM | C8524 | Mediastinal (thymic) large B-cell lymphoma, lymph nodes of axilla and upper limb |
| ICD-10-CM | C8525 | Mediastinal (thymic) large B-cell lymphoma, lymph nodes of inguinal region and lower limb |
| ICD-10-CM | C8526 | Mediastinal (thymic) large B-cell lymphoma, intrapelvic lymph nodes |
| ICD-10-CM | C8527 | Mediastinal (thymic) large B-cell lymphoma, spleen |
| ICD-10-CM | C8528 | Mediastinal (thymic) large B-cell lymphoma, lymph nodes of multiple sites |
| ICD-10-CM | C8529 | Mediastinal (thymic) large B-cell lymphoma, extranodal and solid organ sites |
| ICD-10-CM | C8580 | Other specified types of non-Hodgkin lymphoma, unspecified site |
| ICD-10-CM | C8581 | Other specified types of non-Hodgkin lymphoma, lymph nodes of head, face, and neck |
| ICD-10-CM | C8582 | Other specified types of non-Hodgkin lymphoma, intrathoracic lymph nodes |
| ICD-10-CM | C8583 | Other specified types of non-Hodgkin lymphoma, intra-abdominal lymph nodes |
| ICD-10-CM | C8584 | Other specified types of non-Hodgkin lymphoma, lymph nodes of axilla and upper limb |
| ICD-10-CM | C8585 | Other specified types of non-Hodgkin lymphoma, lymph nodes of inguinal region and lower limb |
| ICD-10-CM | C8586 | Other specified types of non-Hodgkin lymphoma, intrapelvic lymph nodes |
| ICD-10-CM | C8587 | Other specified types of non-Hodgkin lymphoma, spleen |
| ICD-10-CM | C8588 | Other specified types of non-Hodgkin lymphoma, lymph nodes of multiple sites |
| ICD-10-CM | C8589 | Other specified types of non-Hodgkin lymphoma, extranodal and solid organ sites |
| ICD-10-CM | C8590 | Non-Hodgkin lymphoma, unspecified, unspecified site |
| ICD-10-CM | C8591 | Non-Hodgkin lymphoma, unspecified, lymph nodes of head, face, and neck |
| ICD-10-CM | C8592 | Non-Hodgkin lymphoma, unspecified, intrathoracic lymph nodes |
| ICD-10-CM | C8593 | Non-Hodgkin lymphoma, unspecified, intra-abdominal lymph nodes |
| ICD-10-CM | C8594 | Non-Hodgkin lymphoma, unspecified, lymph nodes of axilla and upper limb |
| ICD-10-CM | C8595 | Non-Hodgkin lymphoma, unspecified, lymph nodes of inguinal region and lower limb |
| ICD-10-CM | C8596 | Non-Hodgkin lymphoma, unspecified, intrapelvic lymph nodes |
| ICD-10-CM | C8597 | Non-Hodgkin lymphoma, unspecified, spleen |
| ICD-10-CM | C8598 | Non-Hodgkin lymphoma, unspecified, lymph nodes of multiple sites |
| ICD-10-CM | C8599 | Non-Hodgkin lymphoma, unspecified, extranodal and solid organ sites |
| ICD-10-CM | C860 | Extranodal NK/T-cell lymphoma, nasal type |
| ICD-10-CM | C861 | Hepatosplenic T-cell lymphoma |
| ICD-10-CM | C862 | Enteropathy-type (intestinal) T-cell lymphoma |
| ICD-10-CM | C863 | Subcutaneous panniculitis-like T-cell lymphoma |
| ICD-10-CM | C864 | Blastic NK-cell lymphoma |
| ICD-10-CM | C865 | Angioimmunoblastic T-cell lymphoma |
| ICD-10-CM | C866 | Primary cutaneous CD30-positive T-cell proliferations |
| ICD-10-CM | C880 | Waldenstrom macroglobulinemia |
| ICD-10-CM | C882 | Heavy chain disease |
| ICD-10-CM | C883 | Immunoproliferative small intestinal disease |
| ICD-10-CM | C884 | Extranodal marginal zone B-cell lymphoma of mucosa-associated lymphoid tissue [MALT-lymphoma] |
| ICD-10-CM | C888 | Other malignant immunoproliferative diseases |
| ICD-10-CM | C889 | Malignant immunoproliferative disease, unspecified |
| ICD-10-CM | C9000 | Multiple myeloma not having achieved remission |
| ICD-10-CM | C9001 | Multiple myeloma in remission |
| ICD-10-CM | C9002 | Multiple myeloma in relapse |
| ICD-10-CM | C9010 | Plasma cell leukemia not having achieved remission |
| ICD-10-CM | C9011 | Plasma cell leukemia in remission |
| ICD-10-CM | C9012 | Plasma cell leukemia in relapse |
| ICD-10-CM | C9020 | Extramedullary plasmacytoma not having achieved remission |
| ICD-10-CM | C9021 | Extramedullary plasmacytoma in remission |
| ICD-10-CM | C9022 | Extramedullary plasmacytoma in relapse |
| ICD-10-CM | C9030 | Solitary plasmacytoma not having achieved remission |
| ICD-10-CM | C9031 | Solitary plasmacytoma in remission |
| ICD-10-CM | C9032 | Solitary plasmacytoma in relapse |
| ICD-10-CM | C9100 | Acute lymphoblastic leukemia not having achieved remission |
| ICD-10-CM | C9101 | Acute lymphoblastic leukemia, in remission |
| ICD-10-CM | C9102 | Acute lymphoblastic leukemia, in relapse |
| ICD-10-CM | C9110 | Chronic lymphocytic leukemia of B-cell type not having achieved remission |
| ICD-10-CM | C9111 | Chronic lymphocytic leukemia of B-cell type in remission |
| ICD-10-CM | C9112 | Chronic lymphocytic leukemia of B-cell type in relapse |
| ICD-10-CM | C9130 | Prolymphocytic leukemia of B-cell type not having achieved remission |
| ICD-10-CM | C9131 | Prolymphocytic leukemia of B-cell type, in remission |
| ICD-10-CM | C9132 | Prolymphocytic leukemia of B-cell type, in relapse |
| ICD-10-CM | C9140 | Hairy cell leukemia not having achieved remission |
| ICD-10-CM | C9141 | Hairy cell leukemia, in remission |
| ICD-10-CM | C9142 | Hairy cell leukemia, in relapse |
| ICD-10-CM | C9150 | Adult T-cell lymphoma/leukemia (HTLV-1-associated) not having achieved remission |
| ICD-10-CM | C9151 | Adult T-cell lymphoma/leukemia (HTLV-1-associated), in remission |
| ICD-10-CM | C9152 | Adult T-cell lymphoma/leukemia (HTLV-1-associated), in relapse |
| ICD-10-CM | C9160 | Prolymphocytic leukemia of T-cell type not having achieved remission |
| ICD-10-CM | C9161 | Prolymphocytic leukemia of T-cell type, in remission |
| ICD-10-CM | C9162 | Prolymphocytic leukemia of T-cell type, in relapse |
| ICD-10-CM | C9190 | Lymphoid leukemia, unspecified not having achieved remission |
| ICD-10-CM | C9191 | Lymphoid leukemia, unspecified, in remission |
| ICD-10-CM | C9192 | Lymphoid leukemia, unspecified, in relapse |
| ICD-10-CM | C91A0 | Mature B-cell leukemia Burkitt-type not having achieved remission |
| ICD-10-CM | C91A1 | Mature B-cell leukemia Burkitt-type, in remission |
| ICD-10-CM | C91A2 | Mature B-cell leukemia Burkitt-type, in relapse |
| ICD-10-CM | C91Z0 | Other lymphoid leukemia not having achieved remission |
| ICD-10-CM | C91Z1 | Other lymphoid leukemia, in remission |
| ICD-10-CM | C91Z2 | Other lymphoid leukemia, in relapse |
| ICD-10-CM | C9200 | Acute myeloblastic leukemia, not having achieved remission |
| ICD-10-CM | C9201 | Acute myeloblastic leukemia, in remission |
| ICD-10-CM | C9202 | Acute myeloblastic leukemia, in relapse |
| ICD-10-CM | C9210 | Chronic myeloid leukemia, BCR/ABL-positive, not having achieved remission |
| ICD-10-CM | C9211 | Chronic myeloid leukemia, BCR/ABL-positive, in remission |
| ICD-10-CM | C9212 | Chronic myeloid leukemia, BCR/ABL-positive, in relapse |
| ICD-10-CM | C9220 | Atypical chronic myeloid leukemia, BCR/ABL-negative, not having achieved remission |
| ICD-10-CM | C9221 | Atypical chronic myeloid leukemia, BCR/ABL-negative, in remission |
| ICD-10-CM | C9222 | Atypical chronic myeloid leukemia, BCR/ABL-negative, in relapse |
| ICD-10-CM | C9230 | Myeloid sarcoma, not having achieved remission |
| ICD-10-CM | C9231 | Myeloid sarcoma, in remission |
| ICD-10-CM | C9232 | Myeloid sarcoma, in relapse |
| ICD-10-CM | C9240 | Acute promyelocytic leukemia, not having achieved remission |
| ICD-10-CM | C9241 | Acute promyelocytic leukemia, in remission |
| ICD-10-CM | C9242 | Acute promyelocytic leukemia, in relapse |
| ICD-10-CM | C9250 | Acute myelomonocytic leukemia, not having achieved remission |
| ICD-10-CM | C9251 | Acute myelomonocytic leukemia, in remission |
| ICD-10-CM | C9252 | Acute myelomonocytic leukemia, in relapse |
| ICD-10-CM | C9260 | Acute myeloid leukemia with 11q23-abnormality not having achieved remission |
| ICD-10-CM | C9261 | Acute myeloid leukemia with 11q23-abnormality in remission |
| ICD-10-CM | C9262 | Acute myeloid leukemia with 11q23-abnormality in relapse |
| ICD-10-CM | C9290 | Myeloid leukemia, unspecified, not having achieved remission |
| ICD-10-CM | C9291 | Myeloid leukemia, unspecified in remission |
| ICD-10-CM | C9292 | Myeloid leukemia, unspecified in relapse |
| ICD-10-CM | C92A0 | Acute myeloid leukemia with multilineage dysplasia, not having achieved remission |
| ICD-10-CM | C92A1 | Acute myeloid leukemia with multilineage dysplasia, in remission |
| ICD-10-CM | C92A2 | Acute myeloid leukemia with multilineage dysplasia, in relapse |
| ICD-10-CM | C92Z0 | Other myeloid leukemia not having achieved remission |
| ICD-10-CM | C92Z1 | Other myeloid leukemia, in remission |
| ICD-10-CM | C92Z2 | Other myeloid leukemia, in relapse |
| ICD-10-CM | C9300 | Acute monoblastic/monocytic leukemia, not having achieved remission |
| ICD-10-CM | C9301 | Acute monoblastic/monocytic leukemia, in remission |
| ICD-10-CM | C9302 | Acute monoblastic/monocytic leukemia, in relapse |
| ICD-10-CM | C9310 | Chronic myelomonocytic leukemia not having achieved remission |
| ICD-10-CM | C9311 | Chronic myelomonocytic leukemia, in remission |
| ICD-10-CM | C9312 | Chronic myelomonocytic leukemia, in relapse |
| ICD-10-CM | C9330 | Juvenile myelomonocytic leukemia, not having achieved remission |
| ICD-10-CM | C9331 | Juvenile myelomonocytic leukemia, in remission |
| ICD-10-CM | C9332 | Juvenile myelomonocytic leukemia, in relapse |
| ICD-10-CM | C9390 | Monocytic leukemia, unspecified, not having achieved remission |
| ICD-10-CM | C9391 | Monocytic leukemia, unspecified in remission |
| ICD-10-CM | C9392 | Monocytic leukemia, unspecified in relapse |
| ICD-10-CM | C93Z0 | Other monocytic leukemia, not having achieved remission |
| ICD-10-CM | C93Z1 | Other monocytic leukemia, in remission |
| ICD-10-CM | C93Z2 | Other monocytic leukemia, in relapse |
| ICD-10-CM | C9400 | Acute erythroid leukemia, not having achieved remission |
| ICD-10-CM | C9401 | Acute erythroid leukemia, in remission |
| ICD-10-CM | C9402 | Acute erythroid leukemia, in relapse |
| ICD-10-CM | C9420 | Acute megakaryoblastic leukemia not having achieved remission |
| ICD-10-CM | C9421 | Acute megakaryoblastic leukemia, in remission |
| ICD-10-CM | C9422 | Acute megakaryoblastic leukemia, in relapse |
| ICD-10-CM | C9430 | Mast cell leukemia not having achieved remission |
| ICD-10-CM | C9431 | Mast cell leukemia, in remission |
| ICD-10-CM | C9432 | Mast cell leukemia, in relapse |
| ICD-10-CM | C9440 | Acute panmyelosis with myelofibrosis not having achieved remission |
| ICD-10-CM | C9441 | Acute panmyelosis with myelofibrosis, in remission |
| ICD-10-CM | C9442 | Acute panmyelosis with myelofibrosis, in relapse |
| ICD-10-CM | C946 | Myelodysplastic disease, not classified |
| ICD-10-CM | C9480 | Other specified leukemias not having achieved remission |
| ICD-10-CM | C9481 | Other specified leukemias, in remission |
| ICD-10-CM | C9482 | Other specified leukemias, in relapse |
| ICD-10-CM | C9500 | Acute leukemia of unspecified cell type not having achieved remission |
| ICD-10-CM | C9501 | Acute leukemia of unspecified cell type, in remission |
| ICD-10-CM | C9502 | Acute leukemia of unspecified cell type, in relapse |
| ICD-10-CM | C9510 | Chronic leukemia of unspecified cell type not having achieved remission |
| ICD-10-CM | C9511 | Chronic leukemia of unspecified cell type, in remission |
| ICD-10-CM | C9512 | Chronic leukemia of unspecified cell type, in relapse |
| ICD-10-CM | C9590 | Leukemia, unspecified not having achieved remission |
| ICD-10-CM | C9591 | Leukemia, unspecified, in remission |
| ICD-10-CM | C9592 | Leukemia, unspecified, in relapse |
| ICD-10-CM | C960 | Multifocal and multisystemic (disseminated) Langerhans-cell histiocytosis |
| ICD-10-CM | C962 | Malignant mast cell tumor |
| ICD-10-CM | C9620 | Malignant mast cell neoplasm, unspecified |
| ICD-10-CM | C9621 | Aggressive systemic mastocytosis |
| ICD-10-CM | C9622 | Mast cell sarcoma |
| ICD-10-CM | C9629 | Other malignant mast cell neoplasm |
| ICD-10-CM | C964 | Sarcoma of dendritic cells (accessory cells) |
| ICD-10-CM | C965 | Multifocal and unisystemic Langerhans-cell histiocytosis |
| ICD-10-CM | C966 | Unifocal Langerhans-cell histiocytosis |
| ICD-10-CM | C969 | Malignant neoplasm of lymphoid, hematopoietic and related tissue, unspecified |
| ICD-10-CM | C96A | Histiocytic sarcoma |
| ICD-10-CM | C96Z | Other specified malignant neoplasms of lymphoid, hematopoietic and related tissue |
| ICD-10-CM | D45 | Polycythemia vera |
| ICD-10-CM | D460 | Refractory anemia without ring sideroblasts, so stated |
| ICD-10-CM | D461 | Refractory anemia with ring sideroblasts |
| ICD-10-CM | D4620 | Refractory anemia with excess of blasts, unspecified |
| ICD-10-CM | D4621 | Refractory anemia with excess of blasts 1 |
| ICD-10-CM | D4622 | Refractory anemia with excess of blasts 2 |
| ICD-10-CM | D464 | Refractory anemia, unspecified |
| ICD-10-CM | D469 | Myelodysplastic syndrome, unspecified |
| ICD-10-CM | D46A | Refractory cytopenia with multilineage dysplasia |
| ICD-10-CM | D46B | Refractory cytopenia with multilineage dysplasia and ring sideroblasts |
| ICD-10-CM | D46C | Myelodysplastic syndrome with isolated del(5q) chromosomal abnormality |
| ICD-10-CM | D46Z | Other myelodysplastic syndromes |
| ICD-10-CM | D470 | Histiocytic and mast cell tumors of uncertain behavior |
| ICD-10-CM | D4702 | Systemic mastocytosis |
| ICD-10-CM | D471 | Chronic myeloproliferative disease |
| ICD-10-CM | D472 | Monoclonal gammopathy |
| ICD-10-CM | D473 | Essential (hemorrhagic) thrombocythemia |
| ICD-10-CM | D474 | Osteomyelofibrosis |
| ICD-10-CM | D47Z1 | Post-transplant lymphoproliferative disorder (PTLD) |
| ICD-10-CM | D630 | Anemia in neoplastic disease |
| ICD-10-CM | D6481 | Anemia due to antineoplastic chemotherapy |
| ICD-10-CM | D7581 | Myelofibrosis |
|  |  |  |
| *Non-vitamin-related nutritional deficiencies* |  |  |
| ICD-10-CM | E58 | Dietary calcium deficiency |
| ICD-10-CM | E59 | Dietary selenium deficiency |
| ICD-10-CM | E60 | Dietary zinc deficiency |
| ICD-10-CM | E610 | Copper deficiency |
| ICD-10-CM | E611 | Iron deficiency |
| ICD-10-CM | E612 | Magnesium deficiency |
| ICD-10-CM | E613 | Manganese deficiency |
| ICD-10-CM | E614 | Chromium deficiency |
| ICD-10-CM | E615 | Molybdenum deficiency |
| ICD-10-CM | E616 | Vanadium deficiency |
| ICD-10-CM | E617 | Deficiency of multiple nutrient elements |
| ICD-10-CM | E618 | Deficiency of other specified nutrient elements |
| ICD-10-CM | E619 | Deficiency of nutrient element, unspecified |
| ICD-10-CM | E630 | Essential fatty acid [EFA] deficiency |
| ICD-10-CM | E631 | Imbalance of constituents of food intake |
| ICD-10-CM | E638 | Other specified nutritional deficiencies |
| ICD-10-CM | E639 | Nutritional deficiency, unspecified |
| ICD-10-CM | E40 | Kwashiorkor |
| ICD-10-CM | E41 | Nutritional marasmus |
| ICD-10-CM | E43 | Unspecified severe protien-calore malnutrition |
| ICD-10-CM | E44x | Protein- Calorie malnutrition of moderate and mild degree |
| ICD-10-CM | E45 | Retarded developmen following protien calore malnutrition |
| ICD-10-CM | E46 | Unspecified severe protien-calore malnutrition |
| *Pregnancy* |  |  |
| CPT | 57022 | Incision and drainage of vaginal hematoma; obstetrical/postpartum |
| CPT | 58974 | Embryo transfer, intrauterine |
| CPT | 58976 | Gamete, zygote, or embryo intrafallopian transfer, any method |
| CPT | 59000 | Amniocentesis; diagnostic |
| CPT | 59001 | Amniocentesis; therapeutic amniotic fluid reduction (includes ultrasound guidance) |
| CPT | 59012 | Cordocentesis (intrauterine), any method |
| CPT | 59015 | Chorionic villus sampling, any method |
| CPT | 59020 | Fetal contraction stress test |
| CPT | 59025 | Fetal non-stress test |
| CPT | 59030 | Fetal scalp blood sampling |
| CPT | 59050 | Fetal monitoring during labor by consulting physician (ie, non-attending physician) with written report; supervision and interpretation |
| CPT | 59051 | Fetal monitoring during labor by consulting physician (ie, non-attending physician) with written report; interpretation only |
| CPT | 59070 | Transabdominal amnioinfusion, including ultrasound guidance |
| CPT | 59072 | Fetal umbilical cord occlusion, including ultrasound guidance |
| CPT | 59074 | Fetal fluid drainage (eg, vesicocentesis, thoracocentesis, paracentesis), including ultrasound guidance |
| CPT | 59076 | Fetal shunt placement, including ultrasound guidance |
| CPT | 59100 | Hysterotomy, abdominal (eg, for hydatidiform mole, abortion) |
| CPT | 59120 | Surgical treatment of ectopic pregnancy; tubal or ovarian, requiring salpingectomy and/or oophorectomy, abdominal or vaginal approach |
| CPT | 59121 | Surgical treatment of ectopic pregnancy; tubal or ovarian, without salpingectomy and/or oophorectomy |
| CPT | 59130 | Surgical treatment of ectopic pregnancy; abdominal pregnancy |
| CPT | 59135 | Surgical treatment of ectopic pregnancy; interstitial, uterine pregnancy requiring total hysterectomy |
| CPT | 59136 | Surgical treatment of ectopic pregnancy; interstitial, uterine pregnancy with partial resection of uterus |
| CPT | 59140 | Surgical treatment of ectopic pregnancy; cervical, with evacuation |
| CPT | 59150 | Laparoscopic treatment of ectopic pregnancy; without salpingectomy and/or oophorectomy |
| CPT | 59151 | Laparoscopic treatment of ectopic pregnancy; with salpingectomy and/or oophorectomy |
| CPT | 59160 | Curettage, postpartum |
| CPT | 59200 | Insertion of cervical dilator (eg, laminaria, prostaglandin) (separate procedure) |
| CPT | 59300 | Episiotomy or vaginal repair, by other than attending physician |
| CPT | 59320 | Cerclage of cervix, during pregnancy; vaginal |
| CPT | 59325 | Cerclage of cervix, during pregnancy; abdominal |
| CPT | 59350 | Hysterorrhaphy of ruptured uterus |
| CPT | 59400 | Routine obstetric care including antepartum care, vaginal delivery (with or without episiotomy, and/or forceps) and postpartum care |
| CPT | 59409 | Vaginal delivery only (with or without episiotomy and/or forceps); |
| CPT | 59410 | Vaginal delivery only (with or without episiotomy and/or forceps); including postpartum care |
| CPT | 59412 | External cephalic version, with or without tocolysis |
| CPT | 59414 | Delivery of placenta (separate procedure) |
| CPT | 59425 | Antepartum care only; 4-6 visits |
| CPT | 59426 | Antepartum care only; 7 or more visits |
| CPT | 59430 | Postpartum care only (separate procedure) |
| CPT | 59510 | Routine obstetric care including antepartum care, cesarean delivery, and postpartum care |
| CPT | 59514 | Cesarean delivery only; |
| CPT | 59515 | Cesarean delivery only; including postpartum care |
| CPT | 59525 | Subtotal or total hysterectomy after cesarean delivery (List separately in addition to code for primary procedure) |
| CPT | 59610 | Routine obstetric care including antepartum care, vaginal delivery (with or without episiotomy, and/or forceps) and postpartum care, after previous cesarean delivery |
| CPT | 59612 | Vaginal delivery only, after previous cesarean delivery (with or without episiotomy and/or forceps); |
| CPT | 59614 | Vaginal delivery only, after previous cesarean delivery (with or without episiotomy and/or forceps); including postpartum care |
| CPT | 59618 | Routine obstetric care including antepartum care, cesarean delivery, and postpartum care, following attempted vaginal delivery after previous cesarean delivery |
| CPT | 59620 | Cesarean delivery only, following attempted vaginal delivery after previous cesarean delivery; |
| CPT | 59622 | Cesarean delivery only, following attempted vaginal delivery after previous cesarean delivery; including postpartum care |
| CPT | 59812 | Treatment of incomplete abortion, any trimester, completed surgically |
| CPT | 59820 | Treatment of missed abortion, completed surgically; first trimester |
| CPT | 59821 | Treatment of missed abortion, completed surgically; second trimester |
| CPT | 59830 | Treatment of septic abortion, completed surgically |
| CPT | 59840 | Induced abortion, by dilation and curettage |
| CPT | 59841 | Induced abortion, by dilation and evacuation |
| CPT | 59850 | Induced abortion, by 1 or more intra-amniotic injections (amniocentesis injections), including hospital admission and visits, delivery of fetus and secundines; |
| CPT | 59851 | Induced abortion, by 1 or more intra-amniotic injections (amniocentesis injections), including hospital admission and visits, delivery of fetus and secundines; with dilation and curettage and/or evacuation |
| CPT | 59852 | Induced abortion, by 1 or more intra-amniotic injections (amniocentesis injections), including hospital admission and visits, delivery of fetus and secundines; with hysterotomy (failed intra-amniotic injection) |
| CPT | 59855 | Induced abortion, by 1 or more vaginal suppositories (eg, prostaglandin) with or without cervical dilation (eg, laminaria), including hospital admission and visits, delivery of fetus and secundines; |
| CPT | 59856 | Induced abortion, by 1 or more vaginal suppositories (eg, prostaglandin) with or without cervical dilation (eg, laminaria), including hospital admission and visits, delivery of fetus and secundines; with dilation and curettage and/or evacuation |
| CPT | 59857 | Induced abortion, by 1 or more vaginal suppositories (eg, prostaglandin) with or without cervical dilation (eg, laminaria), including hospital admission and visits, delivery of fetus and secundines; with hysterotomy (failed medical evacuation) |
| CPT | 59866 | Multifetal pregnancy reduction(s) (MPR) |
| CPT | 59870 | Uterine evacuation and curettage for hydatidiform mole |
| CPT | 59871 | Removal of cerclage suture under anesthesia (other than local) |
| CPT | 59897 | Unlisted fetal invasive procedure, including ultrasound guidance, when performed |
| CPT | 59898 | Unlisted laparoscopy procedure, maternity care and delivery |
| CPT | 59899 | Unlisted procedure, maternity care and delivery |
| CPT | 76801 | Ultrasound, pregnant uterus, real time with image documentation, fetal and maternal evaluation, first trimester (< 14 weeks 0 days), transabdominal approach; single or first gestation |
| CPT | 76802 | Ultrasound, pregnant uterus, real time with image documentation, fetal and maternal evaluation, first trimester (< 14 weeks 0 days), transabdominal approach; each additional gestation (List separately in addition to code for primary procedure) |
| CPT | 76805 | Ultrasound, pregnant uterus, real time with image documentation, fetal and maternal evaluation, after first trimester (> or = 14 weeks 0 days), transabdominal approach; single or first gestation |
| CPT | 76810 | Ultrasound, pregnant uterus, real time with image documentation, fetal and maternal evaluation, after first trimester (> or = 14 weeks 0 days), transabdominal approach; each additional gestation (List separately in addition to code for primary procedure) |
| CPT | 76811 | Ultrasound, pregnant uterus, real time with image documentation, fetal and maternal evaluation plus detailed fetal anatomic examination, transabdominal approach; single or first gestation |
| CPT | 76812 | Ultrasound, pregnant uterus, real time with image documentation, fetal and maternal evaluation plus detailed fetal anatomic examination, transabdominal approach; each additional gestation (List separately in addition to code for primary procedure) |
| CPT | 76813 | Ultrasound, pregnant uterus, real time with image documentation, first trimester fetal nuchal translucency measurement, transabdominal or transvaginal approach; single or first gestation |
| CPT | 76814 | Ultrasound, pregnant uterus, real time with image documentation, first trimester fetal nuchal translucency measurement, transabdominal or transvaginal approach; each additional gestation (List separately in addition to code for primary procedure) |
| CPT | 76815 | Ultrasound, pregnant uterus, real time with image documentation, limited (eg, fetal heart beat, placental location, fetal position and/or qualitative amniotic fluid volume), 1 or more fetuses |
| CPT | 76816 | Ultrasound, pregnant uterus, real time with image documentation, follow-up (eg, re-evaluation of fetal size by measuring standard growth parameters and amniotic fluid volume, re-evaluation of organ system(s) suspected or confirmed to be abnormal on a previous scan), transabdominal approach, per fetus |
| CPT | 76817 | Ultrasound, pregnant uterus, real time with image documentation, transvaginal |
| CPT | 76818 | Fetal biophysical profile; with non-stress testing |
| CPT | 76819 | Fetal biophysical profile; without non-stress testing |
| CPT | 76820 | Doppler velocimetry, fetal; umbilical artery |
| CPT | 76821 | Doppler velocimetry, fetal; middle cerebral artery |
| CPT | 76825 | Echocardiography, fetal, cardiovascular system, real time with image documentation (2D), with or without M-mode recording; |
| CPT | 76826 | Echocardiography, fetal, cardiovascular system, real time with image documentation (2D), with or without M-mode recording; follow-up or repeat study |
| CPT | 76827 | Doppler echocardiography, fetal, pulsed wave and/or continuous wave with spectral display; complete |
| CPT | 76828 | Doppler echocardiography, fetal, pulsed wave and/or continuous wave with spectral display; follow-up or repeat study |
| CPT | 76941 | Ultrasonic guidance for intrauterine fetal transfusion or cordocentesis, imaging supervision and interpretation |
| CPT | 76945 | Ultrasonic guidance for chorionic villus sampling, imaging supervision and interpretation |
| CPT | 76946 | Ultrasonic guidance for amniocentesis, imaging supervision and interpretation |
| CPT | 82106 | Alpha-fetoprotein (AFP); amniotic fluid |
| CPT | 0124U | Fetal congenital abnormalities, biochemical assays of 3 analytes (free beta-hCG, PAPP-A, AFP), time-resolved fluorescence immunoassay, maternal dried-blood spot, algorithm reported as risk scores for fetal trisomies 13/18 and 21 |
| CPT | 0125U | Fetal congenital abnormalities and perinatal complications, biochemical assays of 5 analytes (free beta-hCG, PAPP-A, AFP, placental growth factor, and inhibin-A), time-resolved fluorescence immunoassay, maternal serum, algorithm reported as risk scores for fetal trisomies 13/18, 21, and preeclampsia |
| CPT | 0126U | Fetal congenital abnormalities and perinatal complications, biochemical assays of 5 analytes (free beta-hCG, PAPP-A, AFP, placental growth factor, and inhibin-A), time-resolved fluorescence immunoassay, includes qualitative assessment of Y chromosome in cell-free fetal DNA, maternal serum and plasma, predictive algorithm reported as a risk scores for fetal trisomies 13/18, 21, and preeclampsia |
| CPT | 0127U | Obstetrics (preeclampsia), biochemical assays of 3 analytes (PAPP-A, AFP, and placental growth factor), time-resolved fluorescence immunoassay, maternal serum, predictive algorithm reported as a risk score for preeclampsia |
| CPT | 0128U | Obstetrics (preeclampsia), biochemical assays of 3 analytes (PAPP-A, AFP, and placental growth factor), time-resolved fluorescence immunoassay, includes qualitative assessment of Y chromosome in cell-free fetal DNA, maternal serum and plasma, predictive algorithm reported as a risk score for preeclampsia |
| CPT | 01960 | Anesthesia for vaginal delivery only |
| CPT | 01961 | Anesthesia for cesarean delivery only |
| CPT | 01962 | Anesthesia for urgent hysterectomy following delivery |
| CPT | 01963 | Anesthesia for cesarean hysterectomy without any labor analgesia/anesthesia care |
| CPT | 01965 | Anesthesia for incomplete or missed abortion procedures |
| CPT | 01966 | Anesthesia for induced abortion procedures |
| CPT | 01967 | Neuraxial labor analgesia/anesthesia for planned vaginal delivery (this includes any repeat subarachnoid needle placement and drug injection and/or any necessary replacement of an epidural catheter during labor) |
| CPT | 01968 | Anesthesia for cesarean delivery following neuraxial labor analgesia/anesthesia (List separately in addition to code for primary procedure performed) |
| CPT | 01969 | Anesthesia for cesarean hysterectomy following neuraxial labor analgesia/anesthesia (List separately in addition to code for primary procedure performed) |
| CPT | 58605 | Ligation or transection of fallopian tube(s), abdominal or vaginal approach, postpartum, unilateral or bilateral, during same hospitalization (separate procedure) |
| CPT | 58611 | Ligation or transection of fallopian tube(s) when done at the time of cesarean delivery or intra-abdominal surgery (not a separate procedure) (List separately in addition to code for primary procedure) |
| CPT | 81420 | Fetal chromosomal aneuploidy (eg, trisomy 21, monosomy X) genomic sequence analysis panel, circulating cell-free fetal DNA in maternal blood, must include analysis of chromosomes 13, 18, and 21 |
| HCPCS | H1000 | Prenatal care, at-risk assessment |
| HCPCS | H1001 | Prenatal care, at-risk enhanced service; antepartum management |
| HCPCS | H1002 | Prenatal care, at risk enhanced service; care coordination |
| HCPCS | H1003 | Prenatal care, at-risk enhanced service; education |
| HCPCS | H1004 | Prenatal care, at-risk enhanced service; follow-up home visit |
| HCPCS | H1005 | Prenatal care, at-risk enhanced service package (includes H1001-H1004) |
| HCPCS | S0197 | Prenatal vitamins, 30-day supply |
| HCPCS | S0199 | Medically induced abortion by oral ingestion of medication including all associated services and supplies (e.g., patient counseling, office visits, confirmation of pregnancy by HCG, ultrasound to confirm duration of pregnancy, ultrasound to confirm completion of abortion) except drugs |
| HCPCS | S2260 | Induced abortion, 17 to 24 weeks |
| HCPCS | S2265 | Induced abortion, 25 to 28 weeks |
| HCPCS | S2266 | Induced abortion, 29 to 31 weeks |
| HCPCS | S2267 | Induced abortion, 32 weeks or greater |
| HCPCS | S2400 | Repair, congenital diaphragmatic hernia in the fetus using temporary tracheal occlusion, procedure performed in utero |
| HCPCS | S2401 | Repair, urinary tract obstruction in the fetus, procedure performed in utero |
| HCPCS | S2402 | Repair, congenital cystic adenomatoid malformation in the fetus, procedure performed in utero |
| HCPCS | S2403 | Repair, extralobar pulmonary sequestration in the fetus, procedure performed in utero |
| HCPCS | S2404 | Repair, myelomeningocele in the fetus, procedure performed in utero |
| HCPCS | S2405 | Repair of sacrococcygeal teratoma in the fetus, procedure performed in utero |
| HCPCS | S2409 | Repair, congenital malformation of fetus, procedure performed in utero, not otherwise classified |
| HCPCS | S2411 | Fetoscopic laser therapy for treatment of twin-to-twin transfusion syndrome |
| HCPCS | S3625 | Maternal serum triple marker screen including alpha-fetoprotein (AFP), estriol, and human chorionic gonadotropin (HCG) |
| HCPCS | S3626 | Maternal serum quadruple marker screen including alpha-fetoprotein (AFP), estriol, human chorionic gonadotropin hCG) and inhibin A |
| HCPCS | S8055 | Ultrasound guidance for multifetal pregnancy reduction(s), technical component (only to be used when the physician doing the reduction procedure does not perform the ultrasound, guidance is included in the CPT code for multifetal pregnancy reduction (59866) |
| HCPCS | S9212 | Home management of postpartum hypertension, includes administrative services, professional pharmacy services, care coordination, and all necessary supplies and equipment (drugs and nursing visits coded separately), per diem (do not use this code with any home infusion per diem code) |
| HCPCS | S9217 | Nursing services and all necessary equipment and supplies for postpartum hypertension program (includes maternal assessment as needed, telephonic collection of blood pressure, urine protein, weight, compliance management support, patient status reports, 24 hour/7 day a week nursing support, and all education to the patient and caregiver); per diem |
| HCPCS | S9436 | Childbirth preparation/Lamaze classes, nonphysician provider, per session |
| HCPCS | S9437 | Childbirth refresher classes, nonphysician provider, per session |
| HCPCS | S9438 | Cesarean birth classes, nonphysician provider, per session |
| HCPCS | S9439 | VBAC (vaginal birth after cesarean) classes, nonphysician provider, per session |
| HCPCS | S9442 | Birthing classes, nonphysician provider, per session |
| HCPCS | S9443 | Lactation classes, nonphysician provider, per session |
| ICD-10-CM | O000 | Abdominal pregnancy |
| ICD-10-CM | O0000 | Abdominal pregnancy without intrauterine pregnancy |
| ICD-10-CM | O0001 | Abdominal pregnancy with intrauterine pregnancy |
| ICD-10-CM | O001 | Tubal pregnancy |
| ICD-10-CM | O0010 | Tubal pregnancy without intrauterine pregnancy |
| ICD-10-CM | O00101 | Right tubal pregnancy without intrauterine pregnancy |
| ICD-10-CM | O00102 | Left tubal pregnancy without intrauterine pregnancy |
| ICD-10-CM | O00109 | Unspecified tubal pregnancy without intrauterine pregnancy |
| ICD-10-CM | O0011 | Tubal pregnancy with intrauterine pregnancy |
| ICD-10-CM | O00111 | Right tubal pregnancy with intrauterine pregnancy |
| ICD-10-CM | O00112 | Left tubal pregnancy with intrauterine pregnancy |
| ICD-10-CM | O00119 | Unspecified tubal pregnancy with intrauterine pregnancy |
| ICD-10-CM | O002 | Ovarian pregnancy |
| ICD-10-CM | O0020 | Ovarian pregnancy without intrauterine pregnancy |
| ICD-10-CM | O00201 | Right ovarian pregnancy without intrauterine pregnancy |
| ICD-10-CM | O00202 | Left ovarian pregnancy without intrauterine pregnancy |
| ICD-10-CM | O00209 | Unspecified ovarian pregnancy without intrauterine pregnancy |
| ICD-10-CM | O0021 | Ovarian pregnancy with intrauterine pregnancy |
| ICD-10-CM | O00211 | Right ovarian pregnancy with intrauterine pregnancy |
| ICD-10-CM | O00212 | Left ovarian pregnancy with intrauterine pregnancy |
| ICD-10-CM | O00219 | Unspecified ovarian pregnancy with intrauterine pregnancy |
| ICD-10-CM | O008 | Other ectopic pregnancy |
| ICD-10-CM | O0080 | Other ectopic pregnancy without intrauterine pregnancy |
| ICD-10-CM | O0081 | Other ectopic pregnancy with intrauterine pregnancy |
| ICD-10-CM | O009 | Ectopic pregnancy, unspecified |
| ICD-10-CM | O0090 | Unspecified ectopic pregnancy without intrauterine pregnancy |
| ICD-10-CM | O0091 | Unspecified ectopic pregnancy with intrauterine pregnancy |
| ICD-10-CM | O010 | Classical hydatidiform mole |
| ICD-10-CM | O011 | Incomplete and partial hydatidiform mole |
| ICD-10-CM | O019 | Hydatidiform mole, unspecified |
| ICD-10-CM | O020 | Blighted ovum and nonhydatidiform mole |
| ICD-10-CM | O021 | Missed abortion |
| ICD-10-CM | O0281 | Inappropriate change in quantitative human chorionic gonadotropin (hCG) in early pregnancy |
| ICD-10-CM | O0289 | Other abnormal products of conception |
| ICD-10-CM | O029 | Abnormal product of conception, unspecified |
| ICD-10-CM | O030 | Genital tract and pelvic infection following incomplete spontaneous abortion |
| ICD-10-CM | O031 | Delayed or excessive hemorrhage following incomplete spontaneous abortion |
| ICD-10-CM | O032 | Embolism following incomplete spontaneous abortion |
| ICD-10-CM | O0330 | Unspecified complication following incomplete spontaneous abortion |
| ICD-10-CM | O0331 | Shock following incomplete spontaneous abortion |
| ICD-10-CM | O0332 | Renal failure following incomplete spontaneous abortion |
| ICD-10-CM | O0333 | Metabolic disorder following incomplete spontaneous abortion |
| ICD-10-CM | O0334 | Damage to pelvic organs following incomplete spontaneous abortion |
| ICD-10-CM | O0335 | Other venous complications following incomplete spontaneous abortion |
| ICD-10-CM | O0336 | Cardiac arrest following incomplete spontaneous abortion |
| ICD-10-CM | O0337 | Sepsis following incomplete spontaneous abortion |
| ICD-10-CM | O0338 | Urinary tract infection following incomplete spontaneous abortion |
| ICD-10-CM | O0339 | Incomplete spontaneous abortion with other complications |
| ICD-10-CM | O034 | Incomplete spontaneous abortion without complication |
| ICD-10-CM | O035 | Genital tract and pelvic infection following complete or unspecified spontaneous abortion |
| ICD-10-CM | O036 | Delayed or excessive hemorrhage following complete or unspecified spontaneous abortion |
| ICD-10-CM | O037 | Embolism following complete or unspecified spontaneous abortion |
| ICD-10-CM | O0380 | Unspecified complication following complete or unspecified spontaneous abortion |
| ICD-10-CM | O0381 | Shock following complete or unspecified spontaneous abortion |
| ICD-10-CM | O0382 | Renal failure following complete or unspecified spontaneous abortion |
| ICD-10-CM | O0383 | Metabolic disorder following complete or unspecified spontaneous abortion |
| ICD-10-CM | O0384 | Damage to pelvic organs following complete or unspecified spontaneous abortion |
| ICD-10-CM | O0385 | Other venous complications following complete or unspecified spontaneous abortion |
| ICD-10-CM | O0386 | Cardiac arrest following complete or unspecified spontaneous abortion |
| ICD-10-CM | O0387 | Sepsis following complete or unspecified spontaneous abortion |
| ICD-10-CM | O0388 | Urinary tract infection following complete or unspecified spontaneous abortion |
| ICD-10-CM | O0389 | Complete or unspecified spontaneous abortion with other complications |
| ICD-10-CM | O039 | Complete or unspecified spontaneous abortion without complication |
| ICD-10-CM | O045 | Genital tract and pelvic infection following (induced) termination of pregnancy |
| ICD-10-CM | O046 | Delayed or excessive hemorrhage following (induced) termination of pregnancy |
| ICD-10-CM | O047 | Embolism following (induced) termination of pregnancy |
| ICD-10-CM | O0480 | (Induced) termination of pregnancy with unspecified complications |
| ICD-10-CM | O0481 | Shock following (induced) termination of pregnancy |
| ICD-10-CM | O0482 | Renal failure following (induced) termination of pregnancy |
| ICD-10-CM | O0483 | Metabolic disorder following (induced) termination of pregnancy |
| ICD-10-CM | O0484 | Damage to pelvic organs following (induced) termination of pregnancy |
| ICD-10-CM | O0485 | Other venous complications following (induced) termination of pregnancy |
| ICD-10-CM | O0486 | Cardiac arrest following (induced) termination of pregnancy |
| ICD-10-CM | O0487 | Sepsis following (induced) termination of pregnancy |
| ICD-10-CM | O0488 | Urinary tract infection following (induced) termination of pregnancy |
| ICD-10-CM | O0489 | (Induced) termination of pregnancy with other complications |
| ICD-10-CM | O070 | Genital tract and pelvic infection following failed attempted termination of pregnancy |
| ICD-10-CM | O071 | Delayed or excessive hemorrhage following failed attempted termination of pregnancy |
| ICD-10-CM | O072 | Embolism following failed attempted termination of pregnancy |
| ICD-10-CM | O0730 | Failed attempted termination of pregnancy with unspecified complications |
| ICD-10-CM | O0731 | Shock following failed attempted termination of pregnancy |
| ICD-10-CM | O0732 | Renal failure following failed attempted termination of pregnancy |
| ICD-10-CM | O0733 | Metabolic disorder following failed attempted termination of pregnancy |
| ICD-10-CM | O0734 | Damage to pelvic organs following failed attempted termination of pregnancy |
| ICD-10-CM | O0735 | Other venous complications following failed attempted termination of pregnancy |
| ICD-10-CM | O0736 | Cardiac arrest following failed attempted termination of pregnancy |
| ICD-10-CM | O0737 | Sepsis following failed attempted termination of pregnancy |
| ICD-10-CM | O0738 | Urinary tract infection following failed attempted termination of pregnancy |
| ICD-10-CM | O0739 | Failed attempted termination of pregnancy with other complications |
| ICD-10-CM | O074 | Failed attempted termination of pregnancy without complication |
| ICD-10-CM | O080 | Genital tract and pelvic infection following ectopic and molar pregnancy |
| ICD-10-CM | O081 | Delayed or excessive hemorrhage following ectopic and molar pregnancy |
| ICD-10-CM | O082 | Embolism following ectopic and molar pregnancy |
| ICD-10-CM | O083 | Shock following ectopic and molar pregnancy |
| ICD-10-CM | O084 | Renal failure following ectopic and molar pregnancy |
| ICD-10-CM | O085 | Metabolic disorders following an ectopic and molar pregnancy |
| ICD-10-CM | O086 | Damage to pelvic organs and tissues following an ectopic and molar pregnancy |
| ICD-10-CM | O087 | Other venous complications following an ectopic and molar pregnancy |
| ICD-10-CM | O0881 | Cardiac arrest following an ectopic and molar pregnancy |
| ICD-10-CM | O0882 | Sepsis following ectopic and molar pregnancy |
| ICD-10-CM | O0883 | Urinary tract infection following an ectopic and molar pregnancy |
| ICD-10-CM | O0889 | Other complications following an ectopic and molar pregnancy |
| ICD-10-CM | O089 | Unspecified complication following an ectopic and molar pregnancy |
| ICD-10-CM | O0900 | Supervision of pregnancy with history of infertility, unspecified trimester |
| ICD-10-CM | O0901 | Supervision of pregnancy with history of infertility, first trimester |
| ICD-10-CM | O0902 | Supervision of pregnancy with history of infertility, second trimester |
| ICD-10-CM | O0903 | Supervision of pregnancy with history of infertility, third trimester |
| ICD-10-CM | O0910 | Supervision of pregnancy with history of ectopic or molar pregnancy, unspecified trimester |
| ICD-10-CM | O0911 | Supervision of pregnancy with history of ectopic or molar pregnancy, first trimester |
| ICD-10-CM | O0912 | Supervision of pregnancy with history of ectopic or molar pregnancy, second trimester |
| ICD-10-CM | O0913 | Supervision of pregnancy with history of ectopic or molar pregnancy, third trimester |
| ICD-10-CM | O09211 | Supervision of pregnancy with history of pre-term labor, first trimester |
| ICD-10-CM | O09212 | Supervision of pregnancy with history of pre-term labor, second trimester |
| ICD-10-CM | O09213 | Supervision of pregnancy with history of pre-term labor, third trimester |
| ICD-10-CM | O09219 | Supervision of pregnancy with history of pre-term labor, unspecified trimester |
| ICD-10-CM | O09291 | Supervision of pregnancy with other poor reproductive or obstetric history, first trimester |
| ICD-10-CM | O09292 | Supervision of pregnancy with other poor reproductive or obstetric history, second trimester |
| ICD-10-CM | O09293 | Supervision of pregnancy with other poor reproductive or obstetric history, third trimester |
| ICD-10-CM | O09299 | Supervision of pregnancy with other poor reproductive or obstetric history, unspecified trimester |
| ICD-10-CM | O0930 | Supervision of pregnancy with insufficient antenatal care, unspecified trimester |
| ICD-10-CM | O0931 | Supervision of pregnancy with insufficient antenatal care, first trimester |
| ICD-10-CM | O0932 | Supervision of pregnancy with insufficient antenatal care, second trimester |
| ICD-10-CM | O0933 | Supervision of pregnancy with insufficient antenatal care, third trimester |
| ICD-10-CM | O0940 | Supervision of pregnancy with grand multiparity, unspecified trimester |
| ICD-10-CM | O0941 | Supervision of pregnancy with grand multiparity, first trimester |
| ICD-10-CM | O0942 | Supervision of pregnancy with grand multiparity, second trimester |
| ICD-10-CM | O0943 | Supervision of pregnancy with grand multiparity, third trimester |
| ICD-10-CM | O09511 | Supervision of elderly primigravida, first trimester |
| ICD-10-CM | O09512 | Supervision of elderly primigravida, second trimester |
| ICD-10-CM | O09513 | Supervision of elderly primigravida, third trimester |
| ICD-10-CM | O09519 | Supervision of elderly primigravida, unspecified trimester |
| ICD-10-CM | O09521 | Supervision of elderly multigravida, first trimester |
| ICD-10-CM | O09522 | Supervision of elderly multigravida, second trimester |
| ICD-10-CM | O09523 | Supervision of elderly multigravida, third trimester |
| ICD-10-CM | O09529 | Supervision of elderly multigravida, unspecified trimester |
| ICD-10-CM | O09611 | Supervision of young primigravida, first trimester |
| ICD-10-CM | O09612 | Supervision of young primigravida, second trimester |
| ICD-10-CM | O09613 | Supervision of young primigravida, third trimester |
| ICD-10-CM | O09619 | Supervision of young primigravida, unspecified trimester |
| ICD-10-CM | O09621 | Supervision of young multigravida, first trimester |
| ICD-10-CM | O09622 | Supervision of young multigravida, second trimester |
| ICD-10-CM | O09623 | Supervision of young multigravida, third trimester |
| ICD-10-CM | O09629 | Supervision of young multigravida, unspecified trimester |
| ICD-10-CM | O0970 | Supervision of high risk pregnancy due to social problems, unspecified trimester |
| ICD-10-CM | O0971 | Supervision of high risk pregnancy due to social problems, first trimester |
| ICD-10-CM | O0972 | Supervision of high risk pregnancy due to social problems, second trimester |
| ICD-10-CM | O0973 | Supervision of high risk pregnancy due to social problems, third trimester |
| ICD-10-CM | O09811 | Supervision of pregnancy resulting from assisted reproductive technology, first trimester |
| ICD-10-CM | O09812 | Supervision of pregnancy resulting from assisted reproductive technology, second trimester |
| ICD-10-CM | O09813 | Supervision of pregnancy resulting from assisted reproductive technology, third trimester |
| ICD-10-CM | O09819 | Supervision of pregnancy resulting from assisted reproductive technology, unspecified trimester |
| ICD-10-CM | O09821 | Supervision of pregnancy with history of in utero procedure during previous pregnancy, first trimester |
| ICD-10-CM | O09822 | Supervision of pregnancy with history of in utero procedure during previous pregnancy, second trimester |
| ICD-10-CM | O09823 | Supervision of pregnancy with history of in utero procedure during previous pregnancy, third trimester |
| ICD-10-CM | O09829 | Supervision of pregnancy with history of in utero procedure during previous pregnancy, unspecified trimester |
| ICD-10-CM | O09891 | Supervision of other high risk pregnancies, first trimester |
| ICD-10-CM | O09892 | Supervision of other high risk pregnancies, second trimester |
| ICD-10-CM | O09893 | Supervision of other high risk pregnancies, third trimester |
| ICD-10-CM | O09899 | Supervision of other high risk pregnancies, unspecified trimester |
| ICD-10-CM | O0990 | Supervision of high risk pregnancy, unspecified, unspecified trimester |
| ICD-10-CM | O0991 | Supervision of high risk pregnancy, unspecified, first trimester |
| ICD-10-CM | O0992 | Supervision of high risk pregnancy, unspecified, second trimester |
| ICD-10-CM | O0993 | Supervision of high risk pregnancy, unspecified, third trimester |
| ICD-10-CM | O09A0 | Supervision of pregnancy with history of molar pregnancy, unspecified trimester |
| ICD-10-CM | O09A1 | Supervision of pregnancy with history of molar pregnancy, first trimester |
| ICD-10-CM | O09A2 | Supervision of pregnancy with history of molar pregnancy, second trimester |
| ICD-10-CM | O09A3 | Supervision of pregnancy with history of molar pregnancy, third trimester |
| ICD-10-CM | O10011 | Pre-existing essential hypertension complicating pregnancy, first trimester |
| ICD-10-CM | O10012 | Pre-existing essential hypertension complicating pregnancy, second trimester |
| ICD-10-CM | O10013 | Pre-existing essential hypertension complicating pregnancy, third trimester |
| ICD-10-CM | O10019 | Pre-existing essential hypertension complicating pregnancy, unspecified trimester |
| ICD-10-CM | O1002 | Pre-existing essential hypertension complicating childbirth |
| ICD-10-CM | O1003 | Pre-existing essential hypertension complicating the puerperium |
| ICD-10-CM | O10111 | Pre-existing hypertensive heart disease complicating pregnancy, first trimester |
| ICD-10-CM | O10112 | Pre-existing hypertensive heart disease complicating pregnancy, second trimester |
| ICD-10-CM | O10113 | Pre-existing hypertensive heart disease complicating pregnancy, third trimester |
| ICD-10-CM | O10119 | Pre-existing hypertensive heart disease complicating pregnancy, unspecified trimester |
| ICD-10-CM | O1012 | Pre-existing hypertensive heart disease complicating childbirth |
| ICD-10-CM | O1013 | Pre-existing hypertensive heart disease complicating the puerperium |
| ICD-10-CM | O10211 | Pre-existing hypertensive chronic kidney disease complicating pregnancy, first trimester |
| ICD-10-CM | O10212 | Pre-existing hypertensive chronic kidney disease complicating pregnancy, second trimester |
| ICD-10-CM | O10213 | Pre-existing hypertensive chronic kidney disease complicating pregnancy, third trimester |
| ICD-10-CM | O10219 | Pre-existing hypertensive chronic kidney disease complicating pregnancy, unspecified trimester |
| ICD-10-CM | O1022 | Pre-existing hypertensive chronic kidney disease complicating childbirth |
| ICD-10-CM | O1023 | Pre-existing hypertensive chronic kidney disease complicating the puerperium |
| ICD-10-CM | O10311 | Pre-existing hypertensive heart and chronic kidney disease complicating pregnancy, first trimester |
| ICD-10-CM | O10312 | Pre-existing hypertensive heart and chronic kidney disease complicating pregnancy, second trimester |
| ICD-10-CM | O10313 | Pre-existing hypertensive heart and chronic kidney disease complicating pregnancy, third trimester |
| ICD-10-CM | O10319 | Pre-existing hypertensive heart and chronic kidney disease complicating pregnancy, unspecified trimester |
| ICD-10-CM | O1032 | Pre-existing hypertensive heart and chronic kidney disease complicating childbirth |
| ICD-10-CM | O1033 | Pre-existing hypertensive heart and chronic kidney disease complicating the puerperium |
| ICD-10-CM | O10411 | Pre-existing secondary hypertension complicating pregnancy, first trimester |
| ICD-10-CM | O10412 | Pre-existing secondary hypertension complicating pregnancy, second trimester |
| ICD-10-CM | O10413 | Pre-existing secondary hypertension complicating pregnancy, third trimester |
| ICD-10-CM | O10419 | Pre-existing secondary hypertension complicating pregnancy, unspecified trimester |
| ICD-10-CM | O1042 | Pre-existing secondary hypertension complicating childbirth |
| ICD-10-CM | O1043 | Pre-existing secondary hypertension complicating the puerperium |
| ICD-10-CM | O10911 | Unspecified pre-existing hypertension complicating pregnancy, first trimester |
| ICD-10-CM | O10912 | Unspecified pre-existing hypertension complicating pregnancy, second trimester |
| ICD-10-CM | O10913 | Unspecified pre-existing hypertension complicating pregnancy, third trimester |
| ICD-10-CM | O10919 | Unspecified pre-existing hypertension complicating pregnancy, unspecified trimester |
| ICD-10-CM | O1092 | Unspecified pre-existing hypertension complicating childbirth |
| ICD-10-CM | O1093 | Unspecified pre-existing hypertension complicating the puerperium |
| ICD-10-CM | O111 | Pre-existing hypertension with pre-eclampsia, first trimester |
| ICD-10-CM | O112 | Pre-existing hypertension with pre-eclampsia, second trimester |
| ICD-10-CM | O113 | Pre-existing hypertension with pre-eclampsia, third trimester |
| ICD-10-CM | O114 | Pre-existing hypertension with pre-eclampsia, complicating childbirth |
| ICD-10-CM | O115 | Pre-existing hypertension with pre-eclampsia, complicating the puerperium |
| ICD-10-CM | O119 | Pre-existing hypertension with pre-eclampsia, unspecified trimester |
| ICD-10-CM | O1200 | Gestational edema, unspecified trimester |
| ICD-10-CM | O1201 | Gestational edema, first trimester |
| ICD-10-CM | O1202 | Gestational edema, second trimester |
| ICD-10-CM | O1203 | Gestational edema, third trimester |
| ICD-10-CM | O1204 | Gestational edema, complicating childbirth |
| ICD-10-CM | O1205 | Gestational edema, complicating the puerperium |
| ICD-10-CM | O1210 | Gestational proteinuria, unspecified trimester |
| ICD-10-CM | O1211 | Gestational proteinuria, first trimester |
| ICD-10-CM | O1212 | Gestational proteinuria, second trimester |
| ICD-10-CM | O1213 | Gestational proteinuria, third trimester |
| ICD-10-CM | O1214 | Gestational proteinuria, complicating childbirth |
| ICD-10-CM | O1215 | Gestational proteinuria, complicating the puerperium |
| ICD-10-CM | O1220 | Gestational edema with proteinuria, unspecified trimester |
| ICD-10-CM | O1221 | Gestational edema with proteinuria, first trimester |
| ICD-10-CM | O1222 | Gestational edema with proteinuria, second trimester |
| ICD-10-CM | O1223 | Gestational edema with proteinuria, third trimester |
| ICD-10-CM | O1224 | Gestational edema with proteinuria, complicating childbirth |
| ICD-10-CM | O1225 | Gestational edema with proteinuria, complicating the puerperium |
| ICD-10-CM | O131 | Gestational [pregnancy-induced] hypertension without significant proteinuria, first trimester |
| ICD-10-CM | O132 | Gestational [pregnancy-induced] hypertension without significant proteinuria, second trimester |
| ICD-10-CM | O133 | Gestational [pregnancy-induced] hypertension without significant proteinuria, third trimester |
| ICD-10-CM | O134 | Gestational [pregnancy-induced] hypertension without significant proteinuria, complicating childbirth |
| ICD-10-CM | O135 | Gestational [pregnancy-induced] hypertension without significant proteinuria, complicating the puerperium |
| ICD-10-CM | O139 | Gestational [pregnancy-induced] hypertension without significant proteinuria, unspecified trimester |
| ICD-10-CM | O1400 | Mild to moderate pre-eclampsia, unspecified trimester |
| ICD-10-CM | O1402 | Mild to moderate pre-eclampsia, second trimester |
| ICD-10-CM | O1403 | Mild to moderate pre-eclampsia, third trimester |
| ICD-10-CM | O1404 | Mild to moderate pre-eclampsia, complicating childbirth |
| ICD-10-CM | O1405 | Mild to moderate pre-eclampsia, complicating the puerperium |
| ICD-10-CM | O1410 | Severe pre-eclampsia, unspecified trimester |
| ICD-10-CM | O1412 | Severe pre-eclampsia, second trimester |
| ICD-10-CM | O1413 | Severe pre-eclampsia, third trimester |
| ICD-10-CM | O1414 | Severe pre-eclampsia complicating childbirth |
| ICD-10-CM | O1415 | Severe pre-eclampsia, complicating the puerperium |
| ICD-10-CM | O1420 | HELLP syndrome (HELLP), unspecified trimester |
| ICD-10-CM | O1422 | HELLP syndrome (HELLP), second trimester |
| ICD-10-CM | O1423 | HELLP syndrome (HELLP), third trimester |
| ICD-10-CM | O1424 | HELLP syndrome, complicating childbirth |
| ICD-10-CM | O1425 | HELLP syndrome, complicating the puerperium |
| ICD-10-CM | O1490 | Unspecified pre-eclampsia, unspecified trimester |
| ICD-10-CM | O1492 | Unspecified pre-eclampsia, second trimester |
| ICD-10-CM | O1493 | Unspecified pre-eclampsia, third trimester |
| ICD-10-CM | O1494 | Unspecified pre-eclampsia, complicating childbirth |
| ICD-10-CM | O1495 | Unspecified pre-eclampsia, complicating the puerperium |
| ICD-10-CM | O1500 | Eclampsia in pregnancy, unspecified trimester |
| ICD-10-CM | O1502 | Eclampsia in pregnancy, second trimester |
| ICD-10-CM | O1503 | Eclampsia in pregnancy, third trimester |
| ICD-10-CM | O151 | Eclampsia in labor |
| ICD-10-CM | O152 | Eclampsia in the puerperium |
| ICD-10-CM | O159 | Eclampsia, unspecified as to time period |
| ICD-10-CM | O161 | Unspecified maternal hypertension, first trimester |
| ICD-10-CM | O162 | Unspecified maternal hypertension, second trimester |
| ICD-10-CM | O163 | Unspecified maternal hypertension, third trimester |
| ICD-10-CM | O164 | Unspecified maternal hypertension, complicating childbirth |
| ICD-10-CM | O165 | Unspecified maternal hypertension, complicating the puerperium |
| ICD-10-CM | O169 | Unspecified maternal hypertension, unspecified trimester |
| ICD-10-CM | O200 | Threatened abortion |
| ICD-10-CM | O208 | Other hemorrhage in early pregnancy |
| ICD-10-CM | O209 | Hemorrhage in early pregnancy, unspecified |
| ICD-10-CM | O210 | Mild hyperemesis gravidarum |
| ICD-10-CM | O211 | Hyperemesis gravidarum with metabolic disturbance |
| ICD-10-CM | O212 | Late vomiting of pregnancy |
| ICD-10-CM | O218 | Other vomiting complicating pregnancy |
| ICD-10-CM | O219 | Vomiting of pregnancy, unspecified |
| ICD-10-CM | O2200 | Varicose veins of lower extremity in pregnancy, unspecified trimester |
| ICD-10-CM | O2201 | Varicose veins of lower extremity in pregnancy, first trimester |
| ICD-10-CM | O2202 | Varicose veins of lower extremity in pregnancy, second trimester |
| ICD-10-CM | O2203 | Varicose veins of lower extremity in pregnancy, third trimester |
| ICD-10-CM | O2210 | Genital varices in pregnancy, unspecified trimester |
| ICD-10-CM | O2211 | Genital varices in pregnancy, first trimester |
| ICD-10-CM | O2212 | Genital varices in pregnancy, second trimester |
| ICD-10-CM | O2213 | Genital varices in pregnancy, third trimester |
| ICD-10-CM | O2220 | Superficial thrombophlebitis in pregnancy, unspecified trimester |
| ICD-10-CM | O2221 | Superficial thrombophlebitis in pregnancy, first trimester |
| ICD-10-CM | O2222 | Superficial thrombophlebitis in pregnancy, second trimester |
| ICD-10-CM | O2223 | Superficial thrombophlebitis in pregnancy, third trimester |
| ICD-10-CM | O2230 | Deep phlebothrombosis in pregnancy, unspecified trimester |
| ICD-10-CM | O2231 | Deep phlebothrombosis in pregnancy, first trimester |
| ICD-10-CM | O2232 | Deep phlebothrombosis in pregnancy, second trimester |
| ICD-10-CM | O2233 | Deep phlebothrombosis in pregnancy, third trimester |
| ICD-10-CM | O2240 | Hemorrhoids in pregnancy, unspecified trimester |
| ICD-10-CM | O2241 | Hemorrhoids in pregnancy, first trimester |
| ICD-10-CM | O2242 | Hemorrhoids in pregnancy, second trimester |
| ICD-10-CM | O2243 | Hemorrhoids in pregnancy, third trimester |
| ICD-10-CM | O2250 | Cerebral venous thrombosis in pregnancy, unspecified trimester |
| ICD-10-CM | O2251 | Cerebral venous thrombosis in pregnancy, first trimester |
| ICD-10-CM | O2252 | Cerebral venous thrombosis in pregnancy, second trimester |
| ICD-10-CM | O2253 | Cerebral venous thrombosis in pregnancy, third trimester |
| ICD-10-CM | O228X1 | Other venous complications in pregnancy, first trimester |
| ICD-10-CM | O228X2 | Other venous complications in pregnancy, second trimester |
| ICD-10-CM | O228X3 | Other venous complications in pregnancy, third trimester |
| ICD-10-CM | O228X9 | Other venous complications in pregnancy, unspecified trimester |
| ICD-10-CM | O2290 | Venous complication in pregnancy, unspecified, unspecified trimester |
| ICD-10-CM | O2291 | Venous complication in pregnancy, unspecified, first trimester |
| ICD-10-CM | O2292 | Venous complication in pregnancy, unspecified, second trimester |
| ICD-10-CM | O2293 | Venous complication in pregnancy, unspecified, third trimester |
| ICD-10-CM | O2300 | Infections of kidney in pregnancy, unspecified trimester |
| ICD-10-CM | O2301 | Infections of kidney in pregnancy, first trimester |
| ICD-10-CM | O2302 | Infections of kidney in pregnancy, second trimester |
| ICD-10-CM | O2303 | Infections of kidney in pregnancy, third trimester |
| ICD-10-CM | O2310 | Infections of bladder in pregnancy, unspecified trimester |
| ICD-10-CM | O2311 | Infections of bladder in pregnancy, first trimester |
| ICD-10-CM | O2312 | Infections of bladder in pregnancy, second trimester |
| ICD-10-CM | O2313 | Infections of bladder in pregnancy, third trimester |
| ICD-10-CM | O2320 | Infections of urethra in pregnancy, unspecified trimester |
| ICD-10-CM | O2321 | Infections of urethra in pregnancy, first trimester |
| ICD-10-CM | O2322 | Infections of urethra in pregnancy, second trimester |
| ICD-10-CM | O2323 | Infections of urethra in pregnancy, third trimester |
| ICD-10-CM | O2330 | Infections of other parts of urinary tract in pregnancy, unspecified trimester |
| ICD-10-CM | O2331 | Infections of other parts of urinary tract in pregnancy, first trimester |
| ICD-10-CM | O2332 | Infections of other parts of urinary tract in pregnancy, second trimester |
| ICD-10-CM | O2333 | Infections of other parts of urinary tract in pregnancy, third trimester |
| ICD-10-CM | O2340 | Unspecified infection of urinary tract in pregnancy, unspecified trimester |
| ICD-10-CM | O2341 | Unspecified infection of urinary tract in pregnancy, first trimester |
| ICD-10-CM | O2342 | Unspecified infection of urinary tract in pregnancy, second trimester |
| ICD-10-CM | O2343 | Unspecified infection of urinary tract in pregnancy, third trimester |
| ICD-10-CM | O23511 | Infections of cervix in pregnancy, first trimester |
| ICD-10-CM | O23512 | Infections of cervix in pregnancy, second trimester |
| ICD-10-CM | O23513 | Infections of cervix in pregnancy, third trimester |
| ICD-10-CM | O23519 | Infections of cervix in pregnancy, unspecified trimester |
| ICD-10-CM | O23521 | Salpingo-oophoritis in pregnancy, first trimester |
| ICD-10-CM | O23522 | Salpingo-oophoritis in pregnancy, second trimester |
| ICD-10-CM | O23523 | Salpingo-oophoritis in pregnancy, third trimester |
| ICD-10-CM | O23529 | Salpingo-oophoritis in pregnancy, unspecified trimester |
| ICD-10-CM | O23591 | Infection of other part of genital tract in pregnancy, first trimester |
| ICD-10-CM | O23592 | Infection of other part of genital tract in pregnancy, second trimester |
| ICD-10-CM | O23593 | Infection of other part of genital tract in pregnancy, third trimester |
| ICD-10-CM | O23599 | Infection of other part of genital tract in pregnancy, unspecified trimester |
| ICD-10-CM | O2390 | Unspecified genitourinary tract infection in pregnancy, unspecified trimester |
| ICD-10-CM | O2391 | Unspecified genitourinary tract infection in pregnancy, first trimester |
| ICD-10-CM | O2392 | Unspecified genitourinary tract infection in pregnancy, second trimester |
| ICD-10-CM | O2393 | Unspecified genitourinary tract infection in pregnancy, third trimester |
| ICD-10-CM | O24011 | Pre-existing diabetes mellitus, type 1, in pregnancy, first trimester |
| ICD-10-CM | O24012 | Pre-existing diabetes mellitus, type 1, in pregnancy, second trimester |
| ICD-10-CM | O24013 | Pre-existing diabetes mellitus, type 1, in pregnancy, third trimester |
| ICD-10-CM | O24019 | Pre-existing diabetes mellitus, type 1, in pregnancy, unspecified trimester |
| ICD-10-CM | O2402 | Pre-existing diabetes mellitus, type 1, in childbirth |
| ICD-10-CM | O2403 | Pre-existing diabetes mellitus, type 1, in the puerperium |
| ICD-10-CM | O24111 | Pre-existing diabetes mellitus, type 2, in pregnancy, first trimester |
| ICD-10-CM | O24112 | Pre-existing diabetes mellitus, type 2, in pregnancy, second trimester |
| ICD-10-CM | O24113 | Pre-existing diabetes mellitus, type 2, in pregnancy, third trimester |
| ICD-10-CM | O24119 | Pre-existing diabetes mellitus, type 2, in pregnancy, unspecified trimester |
| ICD-10-CM | O2412 | Pre-existing diabetes mellitus, type 2, in childbirth |
| ICD-10-CM | O2413 | Pre-existing diabetes mellitus, type 2, in the puerperium |
| ICD-10-CM | O24311 | Unspecified pre-existing diabetes mellitus in pregnancy, first trimester |
| ICD-10-CM | O24312 | Unspecified pre-existing diabetes mellitus in pregnancy, second trimester |
| ICD-10-CM | O24313 | Unspecified pre-existing diabetes mellitus in pregnancy, third trimester |
| ICD-10-CM | O24319 | Unspecified pre-existing diabetes mellitus in pregnancy, unspecified trimester |
| ICD-10-CM | O2432 | Unspecified pre-existing diabetes mellitus in childbirth |
| ICD-10-CM | O2433 | Unspecified pre-existing diabetes mellitus in the puerperium |
| ICD-10-CM | O24410 | Gestational diabetes mellitus in pregnancy, diet controlled |
| ICD-10-CM | O24414 | Gestational diabetes mellitus in pregnancy, insulin controlled |
| ICD-10-CM | O24415 | Gestational diabetes mellitus in pregnancy, controlled by oral hypoglycemic drugs |
| ICD-10-CM | O24419 | Gestational diabetes mellitus in pregnancy, unspecified control |
| ICD-10-CM | O24420 | Gestational diabetes mellitus in childbirth, diet controlled |
| ICD-10-CM | O24424 | Gestational diabetes mellitus in childbirth, insulin controlled |
| ICD-10-CM | O24425 | Gestational diabetes mellitus in childbirth, controlled by oral hypoglycemic drugs |
| ICD-10-CM | O24429 | Gestational diabetes mellitus in childbirth, unspecified control |
| ICD-10-CM | O24430 | Gestational diabetes mellitus in the puerperium, diet controlled |
| ICD-10-CM | O24434 | Gestational diabetes mellitus in the puerperium, insulin controlled |
| ICD-10-CM | O24435 | Gestational diabetes mellitus in puerperium, controlled by oral hypoglycemic drugs |
| ICD-10-CM | O24439 | Gestational diabetes mellitus in the puerperium, unspecified control |
| ICD-10-CM | O24811 | Other pre-existing diabetes mellitus in pregnancy, first trimester |
| ICD-10-CM | O24812 | Other pre-existing diabetes mellitus in pregnancy, second trimester |
| ICD-10-CM | O24813 | Other pre-existing diabetes mellitus in pregnancy, third trimester |
| ICD-10-CM | O24819 | Other pre-existing diabetes mellitus in pregnancy, unspecified trimester |
| ICD-10-CM | O2482 | Other pre-existing diabetes mellitus in childbirth |
| ICD-10-CM | O2483 | Other pre-existing diabetes mellitus in the puerperium |
| ICD-10-CM | O24911 | Unspecified diabetes mellitus in pregnancy, first trimester |
| ICD-10-CM | O24912 | Unspecified diabetes mellitus in pregnancy, second trimester |
| ICD-10-CM | O24913 | Unspecified diabetes mellitus in pregnancy, third trimester |
| ICD-10-CM | O24919 | Unspecified diabetes mellitus in pregnancy, unspecified trimester |
| ICD-10-CM | O2492 | Unspecified diabetes mellitus in childbirth |
| ICD-10-CM | O2493 | Unspecified diabetes mellitus in the puerperium |
| ICD-10-CM | O2510 | Malnutrition in pregnancy, unspecified trimester |
| ICD-10-CM | O2511 | Malnutrition in pregnancy, first trimester |
| ICD-10-CM | O2512 | Malnutrition in pregnancy, second trimester |
| ICD-10-CM | O2513 | Malnutrition in pregnancy, third trimester |
| ICD-10-CM | O252 | Malnutrition in childbirth |
| ICD-10-CM | O253 | Malnutrition in the puerperium |
| ICD-10-CM | O2600 | Excessive weight gain in pregnancy, unspecified trimester |
| ICD-10-CM | O2601 | Excessive weight gain in pregnancy, first trimester |
| ICD-10-CM | O2602 | Excessive weight gain in pregnancy, second trimester |
| ICD-10-CM | O2603 | Excessive weight gain in pregnancy, third trimester |
| ICD-10-CM | O2610 | Low weight gain in pregnancy, unspecified trimester |
| ICD-10-CM | O2611 | Low weight gain in pregnancy, first trimester |
| ICD-10-CM | O2612 | Low weight gain in pregnancy, second trimester |
| ICD-10-CM | O2613 | Low weight gain in pregnancy, third trimester |
| ICD-10-CM | O2620 | Pregnancy care for patient with recurrent pregnancy loss, unspecified trimester |
| ICD-10-CM | O2621 | Pregnancy care for patient with recurrent pregnancy loss, first trimester |
| ICD-10-CM | O2622 | Pregnancy care for patient with recurrent pregnancy loss, second trimester |
| ICD-10-CM | O2623 | Pregnancy care for patient with recurrent pregnancy loss, third trimester |
| ICD-10-CM | O2630 | Retained intrauterine contraceptive device in pregnancy, unspecified trimester |
| ICD-10-CM | O2631 | Retained intrauterine contraceptive device in pregnancy, first trimester |
| ICD-10-CM | O2632 | Retained intrauterine contraceptive device in pregnancy, second trimester |
| ICD-10-CM | O2633 | Retained intrauterine contraceptive device in pregnancy, third trimester |
| ICD-10-CM | O2640 | Herpes gestationis, unspecified trimester |
| ICD-10-CM | O2641 | Herpes gestationis, first trimester |
| ICD-10-CM | O2642 | Herpes gestationis, second trimester |
| ICD-10-CM | O2643 | Herpes gestationis, third trimester |
| ICD-10-CM | O2650 | Maternal hypotension syndrome, unspecified trimester |
| ICD-10-CM | O2651 | Maternal hypotension syndrome, first trimester |
| ICD-10-CM | O2652 | Maternal hypotension syndrome, second trimester |
| ICD-10-CM | O2653 | Maternal hypotension syndrome, third trimester |
| ICD-10-CM | O26611 | Liver and biliary tract disorders in pregnancy, first trimester |
| ICD-10-CM | O26612 | Liver and biliary tract disorders in pregnancy, second trimester |
| ICD-10-CM | O26613 | Liver and biliary tract disorders in pregnancy, third trimester |
| ICD-10-CM | O26619 | Liver and biliary tract disorders in pregnancy, unspecified trimester |
| ICD-10-CM | O2662 | Liver and biliary tract disorders in childbirth |
| ICD-10-CM | O2663 | Liver and biliary tract disorders in the puerperium |
| ICD-10-CM | O26711 | Subluxation of symphysis (pubis) in pregnancy, first trimester |
| ICD-10-CM | O26712 | Subluxation of symphysis (pubis) in pregnancy, second trimester |
| ICD-10-CM | O26713 | Subluxation of symphysis (pubis) in pregnancy, third trimester |
| ICD-10-CM | O26719 | Subluxation of symphysis (pubis) in pregnancy, unspecified trimester |
| ICD-10-CM | O2672 | Subluxation of symphysis (pubis) in childbirth |
| ICD-10-CM | O2673 | Subluxation of symphysis (pubis) in the puerperium |
| ICD-10-CM | O26811 | Pregnancy related exhaustion and fatigue, first trimester |
| ICD-10-CM | O26812 | Pregnancy related exhaustion and fatigue, second trimester |
| ICD-10-CM | O26813 | Pregnancy related exhaustion and fatigue, third trimester |
| ICD-10-CM | O26819 | Pregnancy related exhaustion and fatigue, unspecified trimester |
| ICD-10-CM | O26821 | Pregnancy related peripheral neuritis, first trimester |
| ICD-10-CM | O26822 | Pregnancy related peripheral neuritis, second trimester |
| ICD-10-CM | O26823 | Pregnancy related peripheral neuritis, third trimester |
| ICD-10-CM | O26829 | Pregnancy related peripheral neuritis, unspecified trimester |
| ICD-10-CM | O26831 | Pregnancy related renal disease, first trimester |
| ICD-10-CM | O26832 | Pregnancy related renal disease, second trimester |
| ICD-10-CM | O26833 | Pregnancy related renal disease, third trimester |
| ICD-10-CM | O26839 | Pregnancy related renal disease, unspecified trimester |
| ICD-10-CM | O26841 | Uterine size-date discrepancy, first trimester |
| ICD-10-CM | O26842 | Uterine size-date discrepancy, second trimester |
| ICD-10-CM | O26843 | Uterine size-date discrepancy, third trimester |
| ICD-10-CM | O26849 | Uterine size-date discrepancy, unspecified trimester |
| ICD-10-CM | O26851 | Spotting complicating pregnancy, first trimester |
| ICD-10-CM | O26852 | Spotting complicating pregnancy, second trimester |
| ICD-10-CM | O26853 | Spotting complicating pregnancy, third trimester |
| ICD-10-CM | O26859 | Spotting complicating pregnancy, unspecified trimester |
| ICD-10-CM | O2686 | Pruritic urticarial papules and plaques of pregnancy (PUPPP) |
| ICD-10-CM | O26872 | Cervical shortening, second trimester |
| ICD-10-CM | O26873 | Cervical shortening, third trimester |
| ICD-10-CM | O26879 | Cervical shortening, unspecified trimester |
| ICD-10-CM | O26891 | Other specified pregnancy related conditions, first trimester |
| ICD-10-CM | O26892 | Other specified pregnancy related conditions, second trimester |
| ICD-10-CM | O26893 | Other specified pregnancy related conditions, third trimester |
| ICD-10-CM | O26899 | Other specified pregnancy related conditions, unspecified trimester |
| ICD-10-CM | O2690 | Pregnancy related conditions, unspecified, unspecified trimester |
| ICD-10-CM | O2691 | Pregnancy related conditions, unspecified, first trimester |
| ICD-10-CM | O2692 | Pregnancy related conditions, unspecified, second trimester |
| ICD-10-CM | O2693 | Pregnancy related conditions, unspecified, third trimester |
| ICD-10-CM | O280 | Abnormal hematological finding on antenatal screening of mother |
| ICD-10-CM | O281 | Abnormal biochemical finding on antenatal screening of mother |
| ICD-10-CM | O282 | Abnormal cytological finding on antenatal screening of mother |
| ICD-10-CM | O283 | Abnormal ultrasonic finding on antenatal screening of mother |
| ICD-10-CM | O284 | Abnormal radiological finding on antenatal screening of mother |
| ICD-10-CM | O285 | Abnormal chromosomal and genetic finding on antenatal screening of mother |
| ICD-10-CM | O288 | Other abnormal findings on antenatal screening of mother |
| ICD-10-CM | O289 | Unspecified abnormal findings on antenatal screening of mother |
| ICD-10-CM | O29011 | Aspiration pneumonitis due to anesthesia during pregnancy, first trimester |
| ICD-10-CM | O29012 | Aspiration pneumonitis due to anesthesia during pregnancy, second trimester |
| ICD-10-CM | O29013 | Aspiration pneumonitis due to anesthesia during pregnancy, third trimester |
| ICD-10-CM | O29019 | Aspiration pneumonitis due to anesthesia during pregnancy, unspecified trimester |
| ICD-10-CM | O29021 | Pressure collapse of lung due to anesthesia during pregnancy, first trimester |
| ICD-10-CM | O29022 | Pressure collapse of lung due to anesthesia during pregnancy, second trimester |
| ICD-10-CM | O29023 | Pressure collapse of lung due to anesthesia during pregnancy, third trimester |
| ICD-10-CM | O29029 | Pressure collapse of lung due to anesthesia during pregnancy, unspecified trimester |
| ICD-10-CM | O29091 | Other pulmonary complications of anesthesia during pregnancy, first trimester |
| ICD-10-CM | O29092 | Other pulmonary complications of anesthesia during pregnancy, second trimester |
| ICD-10-CM | O29093 | Other pulmonary complications of anesthesia during pregnancy, third trimester |
| ICD-10-CM | O29099 | Other pulmonary complications of anesthesia during pregnancy, unspecified trimester |
| ICD-10-CM | O29111 | Cardiac arrest due to anesthesia during pregnancy, first trimester |
| ICD-10-CM | O29112 | Cardiac arrest due to anesthesia during pregnancy, second trimester |
| ICD-10-CM | O29113 | Cardiac arrest due to anesthesia during pregnancy, third trimester |
| ICD-10-CM | O29119 | Cardiac arrest due to anesthesia during pregnancy, unspecified trimester |
| ICD-10-CM | O29121 | Cardiac failure due to anesthesia during pregnancy, first trimester |
| ICD-10-CM | O29122 | Cardiac failure due to anesthesia during pregnancy, second trimester |
| ICD-10-CM | O29123 | Cardiac failure due to anesthesia during pregnancy, third trimester |
| ICD-10-CM | O29129 | Cardiac failure due to anesthesia during pregnancy, unspecified trimester |
| ICD-10-CM | O29191 | Other cardiac complications of anesthesia during pregnancy, first trimester |
| ICD-10-CM | O29192 | Other cardiac complications of anesthesia during pregnancy, second trimester |
| ICD-10-CM | O29193 | Other cardiac complications of anesthesia during pregnancy, third trimester |
| ICD-10-CM | O29199 | Other cardiac complications of anesthesia during pregnancy, unspecified trimester |
| ICD-10-CM | O29211 | Cerebral anoxia due to anesthesia during pregnancy, first trimester |
| ICD-10-CM | O29212 | Cerebral anoxia due to anesthesia during pregnancy, second trimester |
| ICD-10-CM | O29213 | Cerebral anoxia due to anesthesia during pregnancy, third trimester |
| ICD-10-CM | O29219 | Cerebral anoxia due to anesthesia during pregnancy, unspecified trimester |
| ICD-10-CM | O29291 | Other central nervous system complications of anesthesia during pregnancy, first trimester |
| ICD-10-CM | O29292 | Other central nervous system complications of anesthesia during pregnancy, second trimester |
| ICD-10-CM | O29293 | Other central nervous system complications of anesthesia during pregnancy, third trimester |
| ICD-10-CM | O29299 | Other central nervous system complications of anesthesia during pregnancy, unspecified trimester |
| ICD-10-CM | O293X1 | Toxic reaction to local anesthesia during pregnancy, first trimester |
| ICD-10-CM | O293X2 | Toxic reaction to local anesthesia during pregnancy, second trimester |
| ICD-10-CM | O293X3 | Toxic reaction to local anesthesia during pregnancy, third trimester |
| ICD-10-CM | O293X9 | Toxic reaction to local anesthesia during pregnancy, unspecified trimester |
| ICD-10-CM | O2940 | Spinal and epidural anesthesia induced headache during pregnancy, unspecified trimester |
| ICD-10-CM | O2941 | Spinal and epidural anesthesia induced headache during pregnancy, first trimester |
| ICD-10-CM | O2942 | Spinal and epidural anesthesia induced headache during pregnancy, second trimester |
| ICD-10-CM | O2943 | Spinal and epidural anesthesia induced headache during pregnancy, third trimester |
| ICD-10-CM | O295X1 | Other complications of spinal and epidural anesthesia during pregnancy, first trimester |
| ICD-10-CM | O295X2 | Other complications of spinal and epidural anesthesia during pregnancy, second trimester |
| ICD-10-CM | O295X3 | Other complications of spinal and epidural anesthesia during pregnancy, third trimester |
| ICD-10-CM | O295X9 | Other complications of spinal and epidural anesthesia during pregnancy, unspecified trimester |
| ICD-10-CM | O2960 | Failed or difficult intubation for anesthesia during pregnancy, unspecified trimester |
| ICD-10-CM | O2961 | Failed or difficult intubation for anesthesia during pregnancy, first trimester |
| ICD-10-CM | O2962 | Failed or difficult intubation for anesthesia during pregnancy, second trimester |
| ICD-10-CM | O2963 | Failed or difficult intubation for anesthesia during pregnancy, third trimester |
| ICD-10-CM | O298X1 | Other complications of anesthesia during pregnancy, first trimester |
| ICD-10-CM | O298X2 | Other complications of anesthesia during pregnancy, second trimester |
| ICD-10-CM | O298X3 | Other complications of anesthesia during pregnancy, third trimester |
| ICD-10-CM | O298X9 | Other complications of anesthesia during pregnancy, unspecified trimester |
| ICD-10-CM | O2990 | Unspecified complication of anesthesia during pregnancy, unspecified trimester |
| ICD-10-CM | O2991 | Unspecified complication of anesthesia during pregnancy, first trimester |
| ICD-10-CM | O2992 | Unspecified complication of anesthesia during pregnancy, second trimester |
| ICD-10-CM | O2993 | Unspecified complication of anesthesia during pregnancy, third trimester |
| ICD-10-CM | O30001 | Twin pregnancy, unspecified number of placenta and unspecified number of amniotic sacs, first trimester |
| ICD-10-CM | O30002 | Twin pregnancy, unspecified number of placenta and unspecified number of amniotic sacs, second trimester |
| ICD-10-CM | O30003 | Twin pregnancy, unspecified number of placenta and unspecified number of amniotic sacs, third trimester |
| ICD-10-CM | O30009 | Twin pregnancy, unspecified number of placenta and unspecified number of amniotic sacs, unspecified trimester |
| ICD-10-CM | O30011 | Twin pregnancy, monochorionic/monoamniotic, first trimester |
| ICD-10-CM | O30012 | Twin pregnancy, monochorionic/monoamniotic, second trimester |
| ICD-10-CM | O30013 | Twin pregnancy, monochorionic/monoamniotic, third trimester |
| ICD-10-CM | O30019 | Twin pregnancy, monochorionic/monoamniotic, unspecified trimester |
| ICD-10-CM | O30021 | Conjoined twin pregnancy, first trimester |
| ICD-10-CM | O30022 | Conjoined twin pregnancy, second trimester |
| ICD-10-CM | O30023 | Conjoined twin pregnancy, third trimester |
| ICD-10-CM | O30029 | Conjoined twin pregnancy, unspecified trimester |
| ICD-10-CM | O30031 | Twin pregnancy, monochorionic/diamniotic, first trimester |
| ICD-10-CM | O30032 | Twin pregnancy, monochorionic/diamniotic, second trimester |
| ICD-10-CM | O30033 | Twin pregnancy, monochorionic/diamniotic, third trimester |
| ICD-10-CM | O30039 | Twin pregnancy, monochorionic/diamniotic, unspecified trimester |
| ICD-10-CM | O30041 | Twin pregnancy, dichorionic/diamniotic, first trimester |
| ICD-10-CM | O30042 | Twin pregnancy, dichorionic/diamniotic, second trimester |
| ICD-10-CM | O30043 | Twin pregnancy, dichorionic/diamniotic, third trimester |
| ICD-10-CM | O30049 | Twin pregnancy, dichorionic/diamniotic, unspecified trimester |
| ICD-10-CM | O30091 | Twin pregnancy, unable to determine number of placenta and number of amniotic sacs, first trimester |
| ICD-10-CM | O30092 | Twin pregnancy, unable to determine number of placenta and number of amniotic sacs, second trimester |
| ICD-10-CM | O30093 | Twin pregnancy, unable to determine number of placenta and number of amniotic sacs, third trimester |
| ICD-10-CM | O30099 | Twin pregnancy, unable to determine number of placenta and number of amniotic sacs, unspecified trimester |
| ICD-10-CM | O30101 | Triplet pregnancy, unspecified number of placenta and unspecified number of amniotic sacs, first trimester |
| ICD-10-CM | O30102 | Triplet pregnancy, unspecified number of placenta and unspecified number of amniotic sacs, second trimester |
| ICD-10-CM | O30103 | Triplet pregnancy, unspecified number of placenta and unspecified number of amniotic sacs, third trimester |
| ICD-10-CM | O30109 | Triplet pregnancy, unspecified number of placenta and unspecified number of amniotic sacs, unspecified trimester |
| ICD-10-CM | O30111 | Triplet pregnancy with two or more monochorionic fetuses, first trimester |
| ICD-10-CM | O30112 | Triplet pregnancy with two or more monochorionic fetuses, second trimester |
| ICD-10-CM | O30113 | Triplet pregnancy with two or more monochorionic fetuses, third trimester |
| ICD-10-CM | O30119 | Triplet pregnancy with two or more monochorionic fetuses, unspecified trimester |
| ICD-10-CM | O30121 | Triplet pregnancy with two or more monoamniotic fetuses, first trimester |
| ICD-10-CM | O30122 | Triplet pregnancy with two or more monoamniotic fetuses, second trimester |
| ICD-10-CM | O30123 | Triplet pregnancy with two or more monoamniotic fetuses, third trimester |
| ICD-10-CM | O30129 | Triplet pregnancy with two or more monoamniotic fetuses, unspecified trimester |
| ICD-10-CM | O30131 | Triplet pregnancy, trichorionic/triamniotic, first trimester |
| ICD-10-CM | O30132 | Triplet pregnancy, trichorionic/triamniotic, second trimester |
| ICD-10-CM | O30133 | Triplet pregnancy, trichorionic/triamniotic, third trimester |
| ICD-10-CM | O30139 | Triplet pregnancy, trichorionic/triamniotic, unspecified trimester |
| ICD-10-CM | O30191 | Triplet pregnancy, unable to determine number of placenta and number of amniotic sacs, first trimester |
| ICD-10-CM | O30192 | Triplet pregnancy, unable to determine number of placenta and number of amniotic sacs, second trimester |
| ICD-10-CM | O30193 | Triplet pregnancy, unable to determine number of placenta and number of amniotic sacs, third trimester |
| ICD-10-CM | O30199 | Triplet pregnancy, unable to determine number of placenta and number of amniotic sacs, unspecified trimester |
| ICD-10-CM | O30201 | Quadruplet pregnancy, unspecified number of placenta and unspecified number of amniotic sacs, first trimester |
| ICD-10-CM | O30202 | Quadruplet pregnancy, unspecified number of placenta and unspecified number of amniotic sacs, second trimester |
| ICD-10-CM | O30203 | Quadruplet pregnancy, unspecified number of placenta and unspecified number of amniotic sacs, third trimester |
| ICD-10-CM | O30209 | Quadruplet pregnancy, unspecified number of placenta and unspecified number of amniotic sacs, unspecified trimester |
| ICD-10-CM | O30211 | Quadruplet pregnancy with two or more monochorionic fetuses, first trimester |
| ICD-10-CM | O30212 | Quadruplet pregnancy with two or more monochorionic fetuses, second trimester |
| ICD-10-CM | O30213 | Quadruplet pregnancy with two or more monochorionic fetuses, third trimester |
| ICD-10-CM | O30219 | Quadruplet pregnancy with two or more monochorionic fetuses, unspecified trimester |
| ICD-10-CM | O30221 | Quadruplet pregnancy with two or more monoamniotic fetuses, first trimester |
| ICD-10-CM | O30222 | Quadruplet pregnancy with two or more monoamniotic fetuses, second trimester |
| ICD-10-CM | O30223 | Quadruplet pregnancy with two or more monoamniotic fetuses, third trimester |
| ICD-10-CM | O30229 | Quadruplet pregnancy with two or more monoamniotic fetuses, unspecified trimester |
| ICD-10-CM | O30231 | Quadruplet pregnancy, quadrachorionic/quadra-amniotic, first trimester |
| ICD-10-CM | O30232 | Quadruplet pregnancy, quadrachorionic/quadra-amniotic, second trimester |
| ICD-10-CM | O30233 | Quadruplet pregnancy, quadrachorionic/quadra-amniotic, third trimester |
| ICD-10-CM | O30239 | Quadruplet pregnancy, quadrachorionic/quadra-amniotic, unspecified trimester |
| ICD-10-CM | O30291 | Quadruplet pregnancy, unable to determine number of placenta and number of amniotic sacs, first trimester |
| ICD-10-CM | O30292 | Quadruplet pregnancy, unable to determine number of placenta and number of amniotic sacs, second trimester |
| ICD-10-CM | O30293 | Quadruplet pregnancy, unable to determine number of placenta and number of amniotic sacs, third trimester |
| ICD-10-CM | O30299 | Quadruplet pregnancy, unable to determine number of placenta and number of amniotic sacs, unspecified trimester |
| ICD-10-CM | O30801 | Other specified multiple gestation, unspecified number of placenta and unspecified number of amniotic sacs, first trimester |
| ICD-10-CM | O30802 | Other specified multiple gestation, unspecified number of placenta and unspecified number of amniotic sacs, second trimester |
| ICD-10-CM | O30803 | Other specified multiple gestation, unspecified number of placenta and unspecified number of amniotic sacs, third trimester |
| ICD-10-CM | O30809 | Other specified multiple gestation, unspecified number of placenta and unspecified number of amniotic sacs, unspecified trimester |
| ICD-10-CM | O30811 | Other specified multiple gestation with two or more monochorionic fetuses, first trimester |
| ICD-10-CM | O30812 | Other specified multiple gestation with two or more monochorionic fetuses, second trimester |
| ICD-10-CM | O30813 | Other specified multiple gestation with two or more monochorionic fetuses, third trimester |
| ICD-10-CM | O30819 | Other specified multiple gestation with two or more monochorionic fetuses, unspecified trimester |
| ICD-10-CM | O30821 | Other specified multiple gestation with two or more monoamniotic fetuses, first trimester |
| ICD-10-CM | O30822 | Other specified multiple gestation with two or more monoamniotic fetuses, second trimester |
| ICD-10-CM | O30823 | Other specified multiple gestation with two or more monoamniotic fetuses, third trimester |
| ICD-10-CM | O30829 | Other specified multiple gestation with two or more monoamniotic fetuses, unspecified trimester |
| ICD-10-CM | O30831 | Other specified multiple gestation, number of chorions and amnions are both equal to the number of fetuses, first trimester |
| ICD-10-CM | O30832 | Other specified multiple gestation, number of chorions and amnions are both equal to the number of fetuses, second trimester |
| ICD-10-CM | O30833 | Other specified multiple gestation, number of chorions and amnions are both equal to the number of fetuses, third trimester |
| ICD-10-CM | O30839 | Other specified multiple gestation, number of chorions and amnions are both equal to the number of fetuses, unspecified trimester |
| ICD-10-CM | O30891 | Other specified multiple gestation, unable to determine number of placenta and number of amniotic sacs, first trimester |
| ICD-10-CM | O30892 | Other specified multiple gestation, unable to determine number of placenta and number of amniotic sacs, second trimester |
| ICD-10-CM | O30893 | Other specified multiple gestation, unable to determine number of placenta and number of amniotic sacs, third trimester |
| ICD-10-CM | O30899 | Other specified multiple gestation, unable to determine number of placenta and number of amniotic sacs, unspecified trimester |
| ICD-10-CM | O3090 | Multiple gestation, unspecified, unspecified trimester |
| ICD-10-CM | O3091 | Multiple gestation, unspecified, first trimester |
| ICD-10-CM | O3092 | Multiple gestation, unspecified, second trimester |
| ICD-10-CM | O3093 | Multiple gestation, unspecified, third trimester |
| ICD-10-CM | O3100X0 | Papyraceous fetus, unspecified trimester, not applicable or unspecified |
| ICD-10-CM | O3100X1 | Papyraceous fetus, unspecified trimester, fetus 1 |
| ICD-10-CM | O3100X2 | Papyraceous fetus, unspecified trimester, fetus 2 |
| ICD-10-CM | O3100X3 | Papyraceous fetus, unspecified trimester, fetus 3 |
| ICD-10-CM | O3100X4 | Papyraceous fetus, unspecified trimester, fetus 4 |
| ICD-10-CM | O3100X5 | Papyraceous fetus, unspecified trimester, fetus 5 |
| ICD-10-CM | O3100X9 | Papyraceous fetus, unspecified trimester, other fetus |
| ICD-10-CM | O3101X0 | Papyraceous fetus, first trimester, not applicable or unspecified |
| ICD-10-CM | O3101X1 | Papyraceous fetus, first trimester, fetus 1 |
| ICD-10-CM | O3101X2 | Papyraceous fetus, first trimester, fetus 2 |
| ICD-10-CM | O3101X3 | Papyraceous fetus, first trimester, fetus 3 |
| ICD-10-CM | O3101X4 | Papyraceous fetus, first trimester, fetus 4 |
| ICD-10-CM | O3101X5 | Papyraceous fetus, first trimester, fetus 5 |
| ICD-10-CM | O3101X9 | Papyraceous fetus, first trimester, other fetus |
| ICD-10-CM | O3102X0 | Papyraceous fetus, second trimester, not applicable or unspecified |
| ICD-10-CM | O3102X1 | Papyraceous fetus, second trimester, fetus 1 |
| ICD-10-CM | O3102X2 | Papyraceous fetus, second trimester, fetus 2 |
| ICD-10-CM | O3102X3 | Papyraceous fetus, second trimester, fetus 3 |
| ICD-10-CM | O3102X4 | Papyraceous fetus, second trimester, fetus 4 |
| ICD-10-CM | O3102X5 | Papyraceous fetus, second trimester, fetus 5 |
| ICD-10-CM | O3102X9 | Papyraceous fetus, second trimester, other fetus |
| ICD-10-CM | O3103X0 | Papyraceous fetus, third trimester, not applicable or unspecified |
| ICD-10-CM | O3103X1 | Papyraceous fetus, third trimester, fetus 1 |
| ICD-10-CM | O3103X2 | Papyraceous fetus, third trimester, fetus 2 |
| ICD-10-CM | O3103X3 | Papyraceous fetus, third trimester, fetus 3 |
| ICD-10-CM | O3103X4 | Papyraceous fetus, third trimester, fetus 4 |
| ICD-10-CM | O3103X5 | Papyraceous fetus, third trimester, fetus 5 |
| ICD-10-CM | O3103X9 | Papyraceous fetus, third trimester, other fetus |
| ICD-10-CM | O3110X0 | Continuing pregnancy after spontaneous abortion of one fetus or more, unspecified trimester, not applicable or unspecified |
| ICD-10-CM | O3110X1 | Continuing pregnancy after spontaneous abortion of one fetus or more, unspecified trimester, fetus 1 |
| ICD-10-CM | O3110X2 | Continuing pregnancy after spontaneous abortion of one fetus or more, unspecified trimester, fetus 2 |
| ICD-10-CM | O3110X3 | Continuing pregnancy after spontaneous abortion of one fetus or more, unspecified trimester, fetus 3 |
| ICD-10-CM | O3110X4 | Continuing pregnancy after spontaneous abortion of one fetus or more, unspecified trimester, fetus 4 |
| ICD-10-CM | O3110X5 | Continuing pregnancy after spontaneous abortion of one fetus or more, unspecified trimester, fetus 5 |
| ICD-10-CM | O3110X9 | Continuing pregnancy after spontaneous abortion of one fetus or more, unspecified trimester, other fetus |
| ICD-10-CM | O3111X0 | Continuing pregnancy after spontaneous abortion of one fetus or more, first trimester, not applicable or unspecified |
| ICD-10-CM | O3111X1 | Continuing pregnancy after spontaneous abortion of one fetus or more, first trimester, fetus 1 |
| ICD-10-CM | O3111X2 | Continuing pregnancy after spontaneous abortion of one fetus or more, first trimester, fetus 2 |
| ICD-10-CM | O3111X3 | Continuing pregnancy after spontaneous abortion of one fetus or more, first trimester, fetus 3 |
| ICD-10-CM | O3111X4 | Continuing pregnancy after spontaneous abortion of one fetus or more, first trimester, fetus 4 |
| ICD-10-CM | O3111X5 | Continuing pregnancy after spontaneous abortion of one fetus or more, first trimester, fetus 5 |
| ICD-10-CM | O3111X9 | Continuing pregnancy after spontaneous abortion of one fetus or more, first trimester, other fetus |
| ICD-10-CM | O3112X0 | Continuing pregnancy after spontaneous abortion of one fetus or more, second trimester, not applicable or unspecified |
| ICD-10-CM | O3112X1 | Continuing pregnancy after spontaneous abortion of one fetus or more, second trimester, fetus 1 |
| ICD-10-CM | O3112X2 | Continuing pregnancy after spontaneous abortion of one fetus or more, second trimester, fetus 2 |
| ICD-10-CM | O3112X3 | Continuing pregnancy after spontaneous abortion of one fetus or more, second trimester, fetus 3 |
| ICD-10-CM | O3112X4 | Continuing pregnancy after spontaneous abortion of one fetus or more, second trimester, fetus 4 |
| ICD-10-CM | O3112X5 | Continuing pregnancy after spontaneous abortion of one fetus or more, second trimester, fetus 5 |
| ICD-10-CM | O3112X9 | Continuing pregnancy after spontaneous abortion of one fetus or more, second trimester, other fetus |
| ICD-10-CM | O3113X0 | Continuing pregnancy after spontaneous abortion of one fetus or more, third trimester, not applicable or unspecified |
| ICD-10-CM | O3113X1 | Continuing pregnancy after spontaneous abortion of one fetus or more, third trimester, fetus 1 |
| ICD-10-CM | O3113X2 | Continuing pregnancy after spontaneous abortion of one fetus or more, third trimester, fetus 2 |
| ICD-10-CM | O3113X3 | Continuing pregnancy after spontaneous abortion of one fetus or more, third trimester, fetus 3 |
| ICD-10-CM | O3113X4 | Continuing pregnancy after spontaneous abortion of one fetus or more, third trimester, fetus 4 |
| ICD-10-CM | O3113X5 | Continuing pregnancy after spontaneous abortion of one fetus or more, third trimester, fetus 5 |
| ICD-10-CM | O3113X9 | Continuing pregnancy after spontaneous abortion of one fetus or more, third trimester, other fetus |
| ICD-10-CM | O3120X0 | Continuing pregnancy after intrauterine death of one fetus or more, unspecified trimester, not applicable or unspecified |
| ICD-10-CM | O3120X1 | Continuing pregnancy after intrauterine death of one fetus or more, unspecified trimester, fetus 1 |
| ICD-10-CM | O3120X2 | Continuing pregnancy after intrauterine death of one fetus or more, unspecified trimester, fetus 2 |
| ICD-10-CM | O3120X3 | Continuing pregnancy after intrauterine death of one fetus or more, unspecified trimester, fetus 3 |
| ICD-10-CM | O3120X4 | Continuing pregnancy after intrauterine death of one fetus or more, unspecified trimester, fetus 4 |
| ICD-10-CM | O3120X5 | Continuing pregnancy after intrauterine death of one fetus or more, unspecified trimester, fetus 5 |
| ICD-10-CM | O3120X9 | Continuing pregnancy after intrauterine death of one fetus or more, unspecified trimester, other fetus |
| ICD-10-CM | O3121X0 | Continuing pregnancy after intrauterine death of one fetus or more, first trimester, not applicable or unspecified |
| ICD-10-CM | O3121X1 | Continuing pregnancy after intrauterine death of one fetus or more, first trimester, fetus 1 |
| ICD-10-CM | O3121X2 | Continuing pregnancy after intrauterine death of one fetus or more, first trimester, fetus 2 |
| ICD-10-CM | O3121X3 | Continuing pregnancy after intrauterine death of one fetus or more, first trimester, fetus 3 |
| ICD-10-CM | O3121X4 | Continuing pregnancy after intrauterine death of one fetus or more, first trimester, fetus 4 |
| ICD-10-CM | O3121X5 | Continuing pregnancy after intrauterine death of one fetus or more, first trimester, fetus 5 |
| ICD-10-CM | O3121X9 | Continuing pregnancy after intrauterine death of one fetus or more, first trimester, other fetus |
| ICD-10-CM | O3122X0 | Continuing pregnancy after intrauterine death of one fetus or more, second trimester, not applicable or unspecified |
| ICD-10-CM | O3122X1 | Continuing pregnancy after intrauterine death of one fetus or more, second trimester, fetus 1 |
| ICD-10-CM | O3122X2 | Continuing pregnancy after intrauterine death of one fetus or more, second trimester, fetus 2 |
| ICD-10-CM | O3122X3 | Continuing pregnancy after intrauterine death of one fetus or more, second trimester, fetus 3 |
| ICD-10-CM | O3122X4 | Continuing pregnancy after intrauterine death of one fetus or more, second trimester, fetus 4 |
| ICD-10-CM | O3122X5 | Continuing pregnancy after intrauterine death of one fetus or more, second trimester, fetus 5 |
| ICD-10-CM | O3122X9 | Continuing pregnancy after intrauterine death of one fetus or more, second trimester, other fetus |
| ICD-10-CM | O3123X0 | Continuing pregnancy after intrauterine death of one fetus or more, third trimester, not applicable or unspecified |
| ICD-10-CM | O3123X1 | Continuing pregnancy after intrauterine death of one fetus or more, third trimester, fetus 1 |
| ICD-10-CM | O3123X2 | Continuing pregnancy after intrauterine death of one fetus or more, third trimester, fetus 2 |
| ICD-10-CM | O3123X3 | Continuing pregnancy after intrauterine death of one fetus or more, third trimester, fetus 3 |
| ICD-10-CM | O3123X4 | Continuing pregnancy after intrauterine death of one fetus or more, third trimester, fetus 4 |
| ICD-10-CM | O3123X5 | Continuing pregnancy after intrauterine death of one fetus or more, third trimester, fetus 5 |
| ICD-10-CM | O3123X9 | Continuing pregnancy after intrauterine death of one fetus or more, third trimester, other fetus |
| ICD-10-CM | O3130X0 | Continuing pregnancy after elective fetal reduction of one fetus or more, unspecified trimester, not applicable or unspecified |
| ICD-10-CM | O3130X1 | Continuing pregnancy after elective fetal reduction of one fetus or more, unspecified trimester, fetus 1 |
| ICD-10-CM | O3130X2 | Continuing pregnancy after elective fetal reduction of one fetus or more, unspecified trimester, fetus 2 |
| ICD-10-CM | O3130X3 | Continuing pregnancy after elective fetal reduction of one fetus or more, unspecified trimester, fetus 3 |
| ICD-10-CM | O3130X4 | Continuing pregnancy after elective fetal reduction of one fetus or more, unspecified trimester, fetus 4 |
| ICD-10-CM | O3130X5 | Continuing pregnancy after elective fetal reduction of one fetus or more, unspecified trimester, fetus 5 |
| ICD-10-CM | O3130X9 | Continuing pregnancy after elective fetal reduction of one fetus or more, unspecified trimester, other fetus |
| ICD-10-CM | O3131X0 | Continuing pregnancy after elective fetal reduction of one fetus or more, first trimester, not applicable or unspecified |
| ICD-10-CM | O3131X1 | Continuing pregnancy after elective fetal reduction of one fetus or more, first trimester, fetus 1 |
| ICD-10-CM | O3131X2 | Continuing pregnancy after elective fetal reduction of one fetus or more, first trimester, fetus 2 |
| ICD-10-CM | O3131X3 | Continuing pregnancy after elective fetal reduction of one fetus or more, first trimester, fetus 3 |
| ICD-10-CM | O3131X4 | Continuing pregnancy after elective fetal reduction of one fetus or more, first trimester, fetus 4 |
| ICD-10-CM | O3131X5 | Continuing pregnancy after elective fetal reduction of one fetus or more, first trimester, fetus 5 |
| ICD-10-CM | O3131X9 | Continuing pregnancy after elective fetal reduction of one fetus or more, first trimester, other fetus |
| ICD-10-CM | O3132X0 | Continuing pregnancy after elective fetal reduction of one fetus or more, second trimester, not applicable or unspecified |
| ICD-10-CM | O3132X1 | Continuing pregnancy after elective fetal reduction of one fetus or more, second trimester, fetus 1 |
| ICD-10-CM | O3132X2 | Continuing pregnancy after elective fetal reduction of one fetus or more, second trimester, fetus 2 |
| ICD-10-CM | O3132X3 | Continuing pregnancy after elective fetal reduction of one fetus or more, second trimester, fetus 3 |
| ICD-10-CM | O3132X4 | Continuing pregnancy after elective fetal reduction of one fetus or more, second trimester, fetus 4 |
| ICD-10-CM | O3132X5 | Continuing pregnancy after elective fetal reduction of one fetus or more, second trimester, fetus 5 |
| ICD-10-CM | O3132X9 | Continuing pregnancy after elective fetal reduction of one fetus or more, second trimester, other fetus |
| ICD-10-CM | O3133X0 | Continuing pregnancy after elective fetal reduction of one fetus or more, third trimester, not applicable or unspecified |
| ICD-10-CM | O3133X1 | Continuing pregnancy after elective fetal reduction of one fetus or more, third trimester, fetus 1 |
| ICD-10-CM | O3133X2 | Continuing pregnancy after elective fetal reduction of one fetus or more, third trimester, fetus 2 |
| ICD-10-CM | O3133X3 | Continuing pregnancy after elective fetal reduction of one fetus or more, third trimester, fetus 3 |
| ICD-10-CM | O3133X4 | Continuing pregnancy after elective fetal reduction of one fetus or more, third trimester, fetus 4 |
| ICD-10-CM | O3133X5 | Continuing pregnancy after elective fetal reduction of one fetus or more, third trimester, fetus 5 |
| ICD-10-CM | O3133X9 | Continuing pregnancy after elective fetal reduction of one fetus or more, third trimester, other fetus |
| ICD-10-CM | O318X10 | Other complications specific to multiple gestation, first trimester, not applicable or unspecified |
| ICD-10-CM | O318X11 | Other complications specific to multiple gestation, first trimester, fetus 1 |
| ICD-10-CM | O318X12 | Other complications specific to multiple gestation, first trimester, fetus 2 |
| ICD-10-CM | O318X13 | Other complications specific to multiple gestation, first trimester, fetus 3 |
| ICD-10-CM | O318X14 | Other complications specific to multiple gestation, first trimester, fetus 4 |
| ICD-10-CM | O318X15 | Other complications specific to multiple gestation, first trimester, fetus 5 |
| ICD-10-CM | O318X19 | Other complications specific to multiple gestation, first trimester, other fetus |
| ICD-10-CM | O318X20 | Other complications specific to multiple gestation, second trimester, not applicable or unspecified |
| ICD-10-CM | O318X21 | Other complications specific to multiple gestation, second trimester, fetus 1 |
| ICD-10-CM | O318X22 | Other complications specific to multiple gestation, second trimester, fetus 2 |
| ICD-10-CM | O318X23 | Other complications specific to multiple gestation, second trimester, fetus 3 |
| ICD-10-CM | O318X24 | Other complications specific to multiple gestation, second trimester, fetus 4 |
| ICD-10-CM | O318X25 | Other complications specific to multiple gestation, second trimester, fetus 5 |
| ICD-10-CM | O318X29 | Other complications specific to multiple gestation, second trimester, other fetus |
| ICD-10-CM | O318X30 | Other complications specific to multiple gestation, third trimester, not applicable or unspecified |
| ICD-10-CM | O318X31 | Other complications specific to multiple gestation, third trimester, fetus 1 |
| ICD-10-CM | O318X32 | Other complications specific to multiple gestation, third trimester, fetus 2 |
| ICD-10-CM | O318X33 | Other complications specific to multiple gestation, third trimester, fetus 3 |
| ICD-10-CM | O318X34 | Other complications specific to multiple gestation, third trimester, fetus 4 |
| ICD-10-CM | O318X35 | Other complications specific to multiple gestation, third trimester, fetus 5 |
| ICD-10-CM | O318X39 | Other complications specific to multiple gestation, third trimester, other fetus |
| ICD-10-CM | O318X90 | Other complications specific to multiple gestation, unspecified trimester, not applicable or unspecified |
| ICD-10-CM | O318X91 | Other complications specific to multiple gestation, unspecified trimester, fetus 1 |
| ICD-10-CM | O318X92 | Other complications specific to multiple gestation, unspecified trimester, fetus 2 |
| ICD-10-CM | O318X93 | Other complications specific to multiple gestation, unspecified trimester, fetus 3 |
| ICD-10-CM | O318X94 | Other complications specific to multiple gestation, unspecified trimester, fetus 4 |
| ICD-10-CM | O318X95 | Other complications specific to multiple gestation, unspecified trimester, fetus 5 |
| ICD-10-CM | O318X99 | Other complications specific to multiple gestation, unspecified trimester, other fetus |
| ICD-10-CM | O320XX0 | Maternal care for unstable lie, not applicable or unspecified |
| ICD-10-CM | O320XX1 | Maternal care for unstable lie, fetus 1 |
| ICD-10-CM | O320XX2 | Maternal care for unstable lie, fetus 2 |
| ICD-10-CM | O320XX3 | Maternal care for unstable lie, fetus 3 |
| ICD-10-CM | O320XX4 | Maternal care for unstable lie, fetus 4 |
| ICD-10-CM | O320XX5 | Maternal care for unstable lie, fetus 5 |
| ICD-10-CM | O320XX9 | Maternal care for unstable lie, other fetus |
| ICD-10-CM | O321XX0 | Maternal care for breech presentation, not applicable or unspecified |
| ICD-10-CM | O321XX1 | Maternal care for breech presentation, fetus 1 |
| ICD-10-CM | O321XX2 | Maternal care for breech presentation, fetus 2 |
| ICD-10-CM | O321XX3 | Maternal care for breech presentation, fetus 3 |
| ICD-10-CM | O321XX4 | Maternal care for breech presentation, fetus 4 |
| ICD-10-CM | O321XX5 | Maternal care for breech presentation, fetus 5 |
| ICD-10-CM | O321XX9 | Maternal care for breech presentation, other fetus |
| ICD-10-CM | O322XX0 | Maternal care for transverse and oblique lie, not applicable or unspecified |
| ICD-10-CM | O322XX1 | Maternal care for transverse and oblique lie, fetus 1 |
| ICD-10-CM | O322XX2 | Maternal care for transverse and oblique lie, fetus 2 |
| ICD-10-CM | O322XX3 | Maternal care for transverse and oblique lie, fetus 3 |
| ICD-10-CM | O322XX4 | Maternal care for transverse and oblique lie, fetus 4 |
| ICD-10-CM | O322XX5 | Maternal care for transverse and oblique lie, fetus 5 |
| ICD-10-CM | O322XX9 | Maternal care for transverse and oblique lie, other fetus |
| ICD-10-CM | O323XX0 | Maternal care for face, brow and chin presentation, not applicable or unspecified |
| ICD-10-CM | O323XX1 | Maternal care for face, brow and chin presentation, fetus 1 |
| ICD-10-CM | O323XX2 | Maternal care for face, brow and chin presentation, fetus 2 |
| ICD-10-CM | O323XX3 | Maternal care for face, brow and chin presentation, fetus 3 |
| ICD-10-CM | O323XX4 | Maternal care for face, brow and chin presentation, fetus 4 |
| ICD-10-CM | O323XX5 | Maternal care for face, brow and chin presentation, fetus 5 |
| ICD-10-CM | O323XX9 | Maternal care for face, brow and chin presentation, other fetus |
| ICD-10-CM | O324XX0 | Maternal care for high head at term, not applicable or unspecified |
| ICD-10-CM | O324XX1 | Maternal care for high head at term, fetus 1 |
| ICD-10-CM | O324XX2 | Maternal care for high head at term, fetus 2 |
| ICD-10-CM | O324XX3 | Maternal care for high head at term, fetus 3 |
| ICD-10-CM | O324XX4 | Maternal care for high head at term, fetus 4 |
| ICD-10-CM | O324XX5 | Maternal care for high head at term, fetus 5 |
| ICD-10-CM | O324XX9 | Maternal care for high head at term, other fetus |
| ICD-10-CM | O326XX0 | Maternal care for compound presentation, not applicable or unspecified |
| ICD-10-CM | O326XX1 | Maternal care for compound presentation, fetus 1 |
| ICD-10-CM | O326XX2 | Maternal care for compound presentation, fetus 2 |
| ICD-10-CM | O326XX3 | Maternal care for compound presentation, fetus 3 |
| ICD-10-CM | O326XX4 | Maternal care for compound presentation, fetus 4 |
| ICD-10-CM | O326XX5 | Maternal care for compound presentation, fetus 5 |
| ICD-10-CM | O326XX9 | Maternal care for compound presentation, other fetus |
| ICD-10-CM | O328XX0 | Maternal care for other malpresentation of fetus, not applicable or unspecified |
| ICD-10-CM | O328XX1 | Maternal care for other malpresentation of fetus, fetus 1 |
| ICD-10-CM | O328XX2 | Maternal care for other malpresentation of fetus, fetus 2 |
| ICD-10-CM | O328XX3 | Maternal care for other malpresentation of fetus, fetus 3 |
| ICD-10-CM | O328XX4 | Maternal care for other malpresentation of fetus, fetus 4 |
| ICD-10-CM | O328XX5 | Maternal care for other malpresentation of fetus, fetus 5 |
| ICD-10-CM | O328XX9 | Maternal care for other malpresentation of fetus, other fetus |
| ICD-10-CM | O329XX0 | Maternal care for malpresentation of fetus, unspecified, not applicable or unspecified |
| ICD-10-CM | O329XX1 | Maternal care for malpresentation of fetus, unspecified, fetus 1 |
| ICD-10-CM | O329XX2 | Maternal care for malpresentation of fetus, unspecified, fetus 2 |
| ICD-10-CM | O329XX3 | Maternal care for malpresentation of fetus, unspecified, fetus 3 |
| ICD-10-CM | O329XX4 | Maternal care for malpresentation of fetus, unspecified, fetus 4 |
| ICD-10-CM | O329XX5 | Maternal care for malpresentation of fetus, unspecified, fetus 5 |
| ICD-10-CM | O329XX9 | Maternal care for malpresentation of fetus, unspecified, other fetus |
| ICD-10-CM | O330 | Maternal care for disproportion due to deformity of maternal pelvic bones |
| ICD-10-CM | O331 | Maternal care for disproportion due to generally contracted pelvis |
| ICD-10-CM | O332 | Maternal care for disproportion due to inlet contraction of pelvis |
| ICD-10-CM | O333XX0 | Maternal care for disproportion due to outlet contraction of pelvis, not applicable or unspecified |
| ICD-10-CM | O333XX1 | Maternal care for disproportion due to outlet contraction of pelvis, fetus 1 |
| ICD-10-CM | O333XX2 | Maternal care for disproportion due to outlet contraction of pelvis, fetus 2 |
| ICD-10-CM | O333XX3 | Maternal care for disproportion due to outlet contraction of pelvis, fetus 3 |
| ICD-10-CM | O333XX4 | Maternal care for disproportion due to outlet contraction of pelvis, fetus 4 |
| ICD-10-CM | O333XX5 | Maternal care for disproportion due to outlet contraction of pelvis, fetus 5 |
| ICD-10-CM | O333XX9 | Maternal care for disproportion due to outlet contraction of pelvis, other fetus |
| ICD-10-CM | O334XX0 | Maternal care for disproportion of mixed maternal and fetal origin, not applicable or unspecified |
| ICD-10-CM | O334XX1 | Maternal care for disproportion of mixed maternal and fetal origin, fetus 1 |
| ICD-10-CM | O334XX2 | Maternal care for disproportion of mixed maternal and fetal origin, fetus 2 |
| ICD-10-CM | O334XX3 | Maternal care for disproportion of mixed maternal and fetal origin, fetus 3 |
| ICD-10-CM | O334XX4 | Maternal care for disproportion of mixed maternal and fetal origin, fetus 4 |
| ICD-10-CM | O334XX5 | Maternal care for disproportion of mixed maternal and fetal origin, fetus 5 |
| ICD-10-CM | O334XX9 | Maternal care for disproportion of mixed maternal and fetal origin, other fetus |
| ICD-10-CM | O335XX0 | Maternal care for disproportion due to unusually large fetus, not applicable or unspecified |
| ICD-10-CM | O335XX1 | Maternal care for disproportion due to unusually large fetus, fetus 1 |
| ICD-10-CM | O335XX2 | Maternal care for disproportion due to unusually large fetus, fetus 2 |
| ICD-10-CM | O335XX3 | Maternal care for disproportion due to unusually large fetus, fetus 3 |
| ICD-10-CM | O335XX4 | Maternal care for disproportion due to unusually large fetus, fetus 4 |
| ICD-10-CM | O335XX5 | Maternal care for disproportion due to unusually large fetus, fetus 5 |
| ICD-10-CM | O335XX9 | Maternal care for disproportion due to unusually large fetus, other fetus |
| ICD-10-CM | O336XX0 | Maternal care for disproportion due to hydrocephalic fetus, not applicable or unspecified |
| ICD-10-CM | O336XX1 | Maternal care for disproportion due to hydrocephalic fetus, fetus 1 |
| ICD-10-CM | O336XX2 | Maternal care for disproportion due to hydrocephalic fetus, fetus 2 |
| ICD-10-CM | O336XX3 | Maternal care for disproportion due to hydrocephalic fetus, fetus 3 |
| ICD-10-CM | O336XX4 | Maternal care for disproportion due to hydrocephalic fetus, fetus 4 |
| ICD-10-CM | O336XX5 | Maternal care for disproportion due to hydrocephalic fetus, fetus 5 |
| ICD-10-CM | O336XX9 | Maternal care for disproportion due to hydrocephalic fetus, other fetus |
| ICD-10-CM | O337 | Maternal care for disproportion due to other fetal deformities |
| ICD-10-CM | O337XX0 | Maternal care for disproportion due to other fetal deformities, not applicable or unspecified |
| ICD-10-CM | O337XX1 | Maternal care for disproportion due to other fetal deformities, fetus 1 |
| ICD-10-CM | O337XX2 | Maternal care for disproportion due to other fetal deformities, fetus 2 |
| ICD-10-CM | O337XX3 | Maternal care for disproportion due to other fetal deformities, fetus 3 |
| ICD-10-CM | O337XX4 | Maternal care for disproportion due to other fetal deformities, fetus 4 |
| ICD-10-CM | O337XX5 | Maternal care for disproportion due to other fetal deformities, fetus 5 |
| ICD-10-CM | O337XX9 | Maternal care for disproportion due to other fetal deformities, other fetus |
| ICD-10-CM | O338 | Maternal care for disproportion of other origin |
| ICD-10-CM | O339 | Maternal care for disproportion, unspecified |
| ICD-10-CM | O3400 | Maternal care for unspecified congenital malformation of uterus, unspecified trimester |
| ICD-10-CM | O3401 | Maternal care for unspecified congenital malformation of uterus, first trimester |
| ICD-10-CM | O3402 | Maternal care for unspecified congenital malformation of uterus, second trimester |
| ICD-10-CM | O3403 | Maternal care for unspecified congenital malformation of uterus, third trimester |
| ICD-10-CM | O3410 | Maternal care for benign tumor of corpus uteri, unspecified trimester |
| ICD-10-CM | O3411 | Maternal care for benign tumor of corpus uteri, first trimester |
| ICD-10-CM | O3412 | Maternal care for benign tumor of corpus uteri, second trimester |
| ICD-10-CM | O3413 | Maternal care for benign tumor of corpus uteri, third trimester |
| ICD-10-CM | O3421 | Maternal care for scar from previous cesarean delivery |
| ICD-10-CM | O34211 | Maternal care for low transverse scar from previous cesarean delivery |
| ICD-10-CM | O34212 | Maternal care for vertical scar from previous cesarean delivery |
| ICD-10-CM | O34218 | Maternal care for other type scar from previous cesarean delivery |
| ICD-10-CM | O34219 | Maternal care for unspecified type scar from previous cesarean delivery |
| ICD-10-CM | O3422 | Maternal care for cesarean scar defect (isthmocele) |
| ICD-10-CM | O3429 | Maternal care due to uterine scar from other previous surgery |
| ICD-10-CM | O3430 | Maternal care for cervical incompetence, unspecified trimester |
| ICD-10-CM | O3431 | Maternal care for cervical incompetence, first trimester |
| ICD-10-CM | O3432 | Maternal care for cervical incompetence, second trimester |
| ICD-10-CM | O3433 | Maternal care for cervical incompetence, third trimester |
| ICD-10-CM | O3440 | Maternal care for other abnormalities of cervix, unspecified trimester |
| ICD-10-CM | O3441 | Maternal care for other abnormalities of cervix, first trimester |
| ICD-10-CM | O3442 | Maternal care for other abnormalities of cervix, second trimester |
| ICD-10-CM | O3443 | Maternal care for other abnormalities of cervix, third trimester |
| ICD-10-CM | O34511 | Maternal care for incarceration of gravid uterus, first trimester |
| ICD-10-CM | O34512 | Maternal care for incarceration of gravid uterus, second trimester |
| ICD-10-CM | O34513 | Maternal care for incarceration of gravid uterus, third trimester |
| ICD-10-CM | O34519 | Maternal care for incarceration of gravid uterus, unspecified trimester |
| ICD-10-CM | O34521 | Maternal care for prolapse of gravid uterus, first trimester |
| ICD-10-CM | O34522 | Maternal care for prolapse of gravid uterus, second trimester |
| ICD-10-CM | O34523 | Maternal care for prolapse of gravid uterus, third trimester |
| ICD-10-CM | O34529 | Maternal care for prolapse of gravid uterus, unspecified trimester |
| ICD-10-CM | O34531 | Maternal care for retroversion of gravid uterus, first trimester |
| ICD-10-CM | O34532 | Maternal care for retroversion of gravid uterus, second trimester |
| ICD-10-CM | O34533 | Maternal care for retroversion of gravid uterus, third trimester |
| ICD-10-CM | O34539 | Maternal care for retroversion of gravid uterus, unspecified trimester |
| ICD-10-CM | O34591 | Maternal care for other abnormalities of gravid uterus, first trimester |
| ICD-10-CM | O34592 | Maternal care for other abnormalities of gravid uterus, second trimester |
| ICD-10-CM | O34593 | Maternal care for other abnormalities of gravid uterus, third trimester |
| ICD-10-CM | O34599 | Maternal care for other abnormalities of gravid uterus, unspecified trimester |
| ICD-10-CM | O3460 | Maternal care for abnormality of vagina, unspecified trimester |
| ICD-10-CM | O3461 | Maternal care for abnormality of vagina, first trimester |
| ICD-10-CM | O3462 | Maternal care for abnormality of vagina, second trimester |
| ICD-10-CM | O3463 | Maternal care for abnormality of vagina, third trimester |
| ICD-10-CM | O3470 | Maternal care for abnormality of vulva and perineum, unspecified trimester |
| ICD-10-CM | O3471 | Maternal care for abnormality of vulva and perineum, first trimester |
| ICD-10-CM | O3472 | Maternal care for abnormality of vulva and perineum, second trimester |
| ICD-10-CM | O3473 | Maternal care for abnormality of vulva and perineum, third trimester |
| ICD-10-CM | O3480 | Maternal care for other abnormalities of pelvic organs, unspecified trimester |
| ICD-10-CM | O3481 | Maternal care for other abnormalities of pelvic organs, first trimester |
| ICD-10-CM | O3482 | Maternal care for other abnormalities of pelvic organs, second trimester |
| ICD-10-CM | O3483 | Maternal care for other abnormalities of pelvic organs, third trimester |
| ICD-10-CM | O3490 | Maternal care for abnormality of pelvic organ, unspecified, unspecified trimester |
| ICD-10-CM | O3491 | Maternal care for abnormality of pelvic organ, unspecified, first trimester |
| ICD-10-CM | O3492 | Maternal care for abnormality of pelvic organ, unspecified, second trimester |
| ICD-10-CM | O3493 | Maternal care for abnormality of pelvic organ, unspecified, third trimester |
| ICD-10-CM | O350XX0 | Maternal care for (suspected) central nervous system malformation in fetus, not applicable or unspecified |
| ICD-10-CM | O350XX1 | Maternal care for (suspected) central nervous system malformation in fetus, fetus 1 |
| ICD-10-CM | O350XX2 | Maternal care for (suspected) central nervous system malformation in fetus, fetus 2 |
| ICD-10-CM | O350XX3 | Maternal care for (suspected) central nervous system malformation in fetus, fetus 3 |
| ICD-10-CM | O350XX4 | Maternal care for (suspected) central nervous system malformation in fetus, fetus 4 |
| ICD-10-CM | O350XX5 | Maternal care for (suspected) central nervous system malformation in fetus, fetus 5 |
| ICD-10-CM | O350XX9 | Maternal care for (suspected) central nervous system malformation in fetus, other fetus |
| ICD-10-CM | O351XX0 | Maternal care for (suspected) chromosomal abnormality in fetus, not applicable or unspecified |
| ICD-10-CM | O351XX1 | Maternal care for (suspected) chromosomal abnormality in fetus, fetus 1 |
| ICD-10-CM | O351XX2 | Maternal care for (suspected) chromosomal abnormality in fetus, fetus 2 |
| ICD-10-CM | O351XX3 | Maternal care for (suspected) chromosomal abnormality in fetus, fetus 3 |
| ICD-10-CM | O351XX4 | Maternal care for (suspected) chromosomal abnormality in fetus, fetus 4 |
| ICD-10-CM | O351XX5 | Maternal care for (suspected) chromosomal abnormality in fetus, fetus 5 |
| ICD-10-CM | O351XX9 | Maternal care for (suspected) chromosomal abnormality in fetus, other fetus |
| ICD-10-CM | O352XX0 | Maternal care for (suspected) hereditary disease in fetus, not applicable or unspecified |
| ICD-10-CM | O352XX1 | Maternal care for (suspected) hereditary disease in fetus, fetus 1 |
| ICD-10-CM | O352XX2 | Maternal care for (suspected) hereditary disease in fetus, fetus 2 |
| ICD-10-CM | O352XX3 | Maternal care for (suspected) hereditary disease in fetus, fetus 3 |
| ICD-10-CM | O352XX4 | Maternal care for (suspected) hereditary disease in fetus, fetus 4 |
| ICD-10-CM | O352XX5 | Maternal care for (suspected) hereditary disease in fetus, fetus 5 |
| ICD-10-CM | O352XX9 | Maternal care for (suspected) hereditary disease in fetus, other fetus |
| ICD-10-CM | O353XX0 | Maternal care for (suspected) damage to fetus from viral disease in mother, not applicable or unspecified |
| ICD-10-CM | O353XX1 | Maternal care for (suspected) damage to fetus from viral disease in mother, fetus 1 |
| ICD-10-CM | O353XX2 | Maternal care for (suspected) damage to fetus from viral disease in mother, fetus 2 |
| ICD-10-CM | O353XX3 | Maternal care for (suspected) damage to fetus from viral disease in mother, fetus 3 |
| ICD-10-CM | O353XX4 | Maternal care for (suspected) damage to fetus from viral disease in mother, fetus 4 |
| ICD-10-CM | O353XX5 | Maternal care for (suspected) damage to fetus from viral disease in mother, fetus 5 |
| ICD-10-CM | O353XX9 | Maternal care for (suspected) damage to fetus from viral disease in mother, other fetus |
| ICD-10-CM | O354XX0 | Maternal care for (suspected) damage to fetus from alcohol, not applicable or unspecified |
| ICD-10-CM | O354XX1 | Maternal care for (suspected) damage to fetus from alcohol, fetus 1 |
| ICD-10-CM | O354XX2 | Maternal care for (suspected) damage to fetus from alcohol, fetus 2 |
| ICD-10-CM | O354XX3 | Maternal care for (suspected) damage to fetus from alcohol, fetus 3 |
| ICD-10-CM | O354XX4 | Maternal care for (suspected) damage to fetus from alcohol, fetus 4 |
| ICD-10-CM | O354XX5 | Maternal care for (suspected) damage to fetus from alcohol, fetus 5 |
| ICD-10-CM | O354XX9 | Maternal care for (suspected) damage to fetus from alcohol, other fetus |
| ICD-10-CM | O355XX0 | Maternal care for (suspected) damage to fetus by drugs, not applicable or unspecified |
| ICD-10-CM | O355XX1 | Maternal care for (suspected) damage to fetus by drugs, fetus 1 |
| ICD-10-CM | O355XX2 | Maternal care for (suspected) damage to fetus by drugs, fetus 2 |
| ICD-10-CM | O355XX3 | Maternal care for (suspected) damage to fetus by drugs, fetus 3 |
| ICD-10-CM | O355XX4 | Maternal care for (suspected) damage to fetus by drugs, fetus 4 |
| ICD-10-CM | O355XX5 | Maternal care for (suspected) damage to fetus by drugs, fetus 5 |
| ICD-10-CM | O355XX9 | Maternal care for (suspected) damage to fetus by drugs, other fetus |
| ICD-10-CM | O356XX0 | Maternal care for (suspected) damage to fetus by radiation, not applicable or unspecified |
| ICD-10-CM | O356XX1 | Maternal care for (suspected) damage to fetus by radiation, fetus 1 |
| ICD-10-CM | O356XX2 | Maternal care for (suspected) damage to fetus by radiation, fetus 2 |
| ICD-10-CM | O356XX3 | Maternal care for (suspected) damage to fetus by radiation, fetus 3 |
| ICD-10-CM | O356XX4 | Maternal care for (suspected) damage to fetus by radiation, fetus 4 |
| ICD-10-CM | O356XX5 | Maternal care for (suspected) damage to fetus by radiation, fetus 5 |
| ICD-10-CM | O356XX9 | Maternal care for (suspected) damage to fetus by radiation, other fetus |
| ICD-10-CM | O357XX0 | Maternal care for (suspected) damage to fetus by other medical procedures, not applicable or unspecified |
| ICD-10-CM | O357XX1 | Maternal care for (suspected) damage to fetus by other medical procedures, fetus 1 |
| ICD-10-CM | O357XX2 | Maternal care for (suspected) damage to fetus by other medical procedures, fetus 2 |
| ICD-10-CM | O357XX3 | Maternal care for (suspected) damage to fetus by other medical procedures, fetus 3 |
| ICD-10-CM | O357XX4 | Maternal care for (suspected) damage to fetus by other medical procedures, fetus 4 |
| ICD-10-CM | O357XX5 | Maternal care for (suspected) damage to fetus by other medical procedures, fetus 5 |
| ICD-10-CM | O357XX9 | Maternal care for (suspected) damage to fetus by other medical procedures, other fetus |
| ICD-10-CM | O358XX0 | Maternal care for other (suspected) fetal abnormality and damage, not applicable or unspecified |
| ICD-10-CM | O358XX1 | Maternal care for other (suspected) fetal abnormality and damage, fetus 1 |
| ICD-10-CM | O358XX2 | Maternal care for other (suspected) fetal abnormality and damage, fetus 2 |
| ICD-10-CM | O358XX3 | Maternal care for other (suspected) fetal abnormality and damage, fetus 3 |
| ICD-10-CM | O358XX4 | Maternal care for other (suspected) fetal abnormality and damage, fetus 4 |
| ICD-10-CM | O358XX5 | Maternal care for other (suspected) fetal abnormality and damage, fetus 5 |
| ICD-10-CM | O358XX9 | Maternal care for other (suspected) fetal abnormality and damage, other fetus |
| ICD-10-CM | O359XX0 | Maternal care for (suspected) fetal abnormality and damage, unspecified, not applicable or unspecified |
| ICD-10-CM | O359XX1 | Maternal care for (suspected) fetal abnormality and damage, unspecified, fetus 1 |
| ICD-10-CM | O359XX2 | Maternal care for (suspected) fetal abnormality and damage, unspecified, fetus 2 |
| ICD-10-CM | O359XX3 | Maternal care for (suspected) fetal abnormality and damage, unspecified, fetus 3 |
| ICD-10-CM | O359XX4 | Maternal care for (suspected) fetal abnormality and damage, unspecified, fetus 4 |
| ICD-10-CM | O359XX5 | Maternal care for (suspected) fetal abnormality and damage, unspecified, fetus 5 |
| ICD-10-CM | O359XX9 | Maternal care for (suspected) fetal abnormality and damage, unspecified, other fetus |
| ICD-10-CM | O360110 | Maternal care for anti-D [Rh] antibodies, first trimester, not applicable or unspecified |
| ICD-10-CM | O360111 | Maternal care for anti-D [Rh] antibodies, first trimester, fetus 1 |
| ICD-10-CM | O360112 | Maternal care for anti-D [Rh] antibodies, first trimester, fetus 2 |
| ICD-10-CM | O360113 | Maternal care for anti-D [Rh] antibodies, first trimester, fetus 3 |
| ICD-10-CM | O360114 | Maternal care for anti-D [Rh] antibodies, first trimester, fetus 4 |
| ICD-10-CM | O360115 | Maternal care for anti-D [Rh] antibodies, first trimester, fetus 5 |
| ICD-10-CM | O360119 | Maternal care for anti-D [Rh] antibodies, first trimester, other fetus |
| ICD-10-CM | O360120 | Maternal care for anti-D [Rh] antibodies, second trimester, not applicable or unspecified |
| ICD-10-CM | O360121 | Maternal care for anti-D [Rh] antibodies, second trimester, fetus 1 |
| ICD-10-CM | O360122 | Maternal care for anti-D [Rh] antibodies, second trimester, fetus 2 |
| ICD-10-CM | O360123 | Maternal care for anti-D [Rh] antibodies, second trimester, fetus 3 |
| ICD-10-CM | O360124 | Maternal care for anti-D [Rh] antibodies, second trimester, fetus 4 |
| ICD-10-CM | O360125 | Maternal care for anti-D [Rh] antibodies, second trimester, fetus 5 |
| ICD-10-CM | O360129 | Maternal care for anti-D [Rh] antibodies, second trimester, other fetus |
| ICD-10-CM | O360130 | Maternal care for anti-D [Rh] antibodies, third trimester, not applicable or unspecified |
| ICD-10-CM | O360131 | Maternal care for anti-D [Rh] antibodies, third trimester, fetus 1 |
| ICD-10-CM | O360132 | Maternal care for anti-D [Rh] antibodies, third trimester, fetus 2 |
| ICD-10-CM | O360133 | Maternal care for anti-D [Rh] antibodies, third trimester, fetus 3 |
| ICD-10-CM | O360134 | Maternal care for anti-D [Rh] antibodies, third trimester, fetus 4 |
| ICD-10-CM | O360135 | Maternal care for anti-D [Rh] antibodies, third trimester, fetus 5 |
| ICD-10-CM | O360139 | Maternal care for anti-D [Rh] antibodies, third trimester, other fetus |
| ICD-10-CM | O360190 | Maternal care for anti-D [Rh] antibodies, unspecified trimester, not applicable or unspecified |
| ICD-10-CM | O360191 | Maternal care for anti-D [Rh] antibodies, unspecified trimester, fetus 1 |
| ICD-10-CM | O360192 | Maternal care for anti-D [Rh] antibodies, unspecified trimester, fetus 2 |
| ICD-10-CM | O360193 | Maternal care for anti-D [Rh] antibodies, unspecified trimester, fetus 3 |
| ICD-10-CM | O360194 | Maternal care for anti-D [Rh] antibodies, unspecified trimester, fetus 4 |
| ICD-10-CM | O360195 | Maternal care for anti-D [Rh] antibodies, unspecified trimester, fetus 5 |
| ICD-10-CM | O360199 | Maternal care for anti-D [Rh] antibodies, unspecified trimester, other fetus |
| ICD-10-CM | O360910 | Maternal care for other rhesus isoimmunization, first trimester, not applicable or unspecified |
| ICD-10-CM | O360911 | Maternal care for other rhesus isoimmunization, first trimester, fetus 1 |
| ICD-10-CM | O360912 | Maternal care for other rhesus isoimmunization, first trimester, fetus 2 |
| ICD-10-CM | O360913 | Maternal care for other rhesus isoimmunization, first trimester, fetus 3 |
| ICD-10-CM | O360914 | Maternal care for other rhesus isoimmunization, first trimester, fetus 4 |
| ICD-10-CM | O360915 | Maternal care for other rhesus isoimmunization, first trimester, fetus 5 |
| ICD-10-CM | O360919 | Maternal care for other rhesus isoimmunization, first trimester, other fetus |
| ICD-10-CM | O360920 | Maternal care for other rhesus isoimmunization, second trimester, not applicable or unspecified |
| ICD-10-CM | O360921 | Maternal care for other rhesus isoimmunization, second trimester, fetus 1 |
| ICD-10-CM | O360922 | Maternal care for other rhesus isoimmunization, second trimester, fetus 2 |
| ICD-10-CM | O360923 | Maternal care for other rhesus isoimmunization, second trimester, fetus 3 |
| ICD-10-CM | O360924 | Maternal care for other rhesus isoimmunization, second trimester, fetus 4 |
| ICD-10-CM | O360925 | Maternal care for other rhesus isoimmunization, second trimester, fetus 5 |
| ICD-10-CM | O360929 | Maternal care for other rhesus isoimmunization, second trimester, other fetus |
| ICD-10-CM | O360930 | Maternal care for other rhesus isoimmunization, third trimester, not applicable or unspecified |
| ICD-10-CM | O360931 | Maternal care for other rhesus isoimmunization, third trimester, fetus 1 |
| ICD-10-CM | O360932 | Maternal care for other rhesus isoimmunization, third trimester, fetus 2 |
| ICD-10-CM | O360933 | Maternal care for other rhesus isoimmunization, third trimester, fetus 3 |
| ICD-10-CM | O360934 | Maternal care for other rhesus isoimmunization, third trimester, fetus 4 |
| ICD-10-CM | O360935 | Maternal care for other rhesus isoimmunization, third trimester, fetus 5 |
| ICD-10-CM | O360939 | Maternal care for other rhesus isoimmunization, third trimester, other fetus |
| ICD-10-CM | O360990 | Maternal care for other rhesus isoimmunization, unspecified trimester, not applicable or unspecified |
| ICD-10-CM | O360991 | Maternal care for other rhesus isoimmunization, unspecified trimester, fetus 1 |
| ICD-10-CM | O360992 | Maternal care for other rhesus isoimmunization, unspecified trimester, fetus 2 |
| ICD-10-CM | O360993 | Maternal care for other rhesus isoimmunization, unspecified trimester, fetus 3 |
| ICD-10-CM | O360994 | Maternal care for other rhesus isoimmunization, unspecified trimester, fetus 4 |
| ICD-10-CM | O360995 | Maternal care for other rhesus isoimmunization, unspecified trimester, fetus 5 |
| ICD-10-CM | O360999 | Maternal care for other rhesus isoimmunization, unspecified trimester, other fetus |
| ICD-10-CM | O361110 | Maternal care for Anti-A sensitization, first trimester, not applicable or unspecified |
| ICD-10-CM | O361111 | Maternal care for Anti-A sensitization, first trimester, fetus 1 |
| ICD-10-CM | O361112 | Maternal care for Anti-A sensitization, first trimester, fetus 2 |
| ICD-10-CM | O361113 | Maternal care for Anti-A sensitization, first trimester, fetus 3 |
| ICD-10-CM | O361114 | Maternal care for Anti-A sensitization, first trimester, fetus 4 |
| ICD-10-CM | O361115 | Maternal care for Anti-A sensitization, first trimester, fetus 5 |
| ICD-10-CM | O361119 | Maternal care for Anti-A sensitization, first trimester, other fetus |
| ICD-10-CM | O361120 | Maternal care for Anti-A sensitization, second trimester, not applicable or unspecified |
| ICD-10-CM | O361121 | Maternal care for Anti-A sensitization, second trimester, fetus 1 |
| ICD-10-CM | O361122 | Maternal care for Anti-A sensitization, second trimester, fetus 2 |
| ICD-10-CM | O361123 | Maternal care for Anti-A sensitization, second trimester, fetus 3 |
| ICD-10-CM | O361124 | Maternal care for Anti-A sensitization, second trimester, fetus 4 |
| ICD-10-CM | O361125 | Maternal care for Anti-A sensitization, second trimester, fetus 5 |
| ICD-10-CM | O361129 | Maternal care for Anti-A sensitization, second trimester, other fetus |
| ICD-10-CM | O361130 | Maternal care for Anti-A sensitization, third trimester, not applicable or unspecified |
| ICD-10-CM | O361131 | Maternal care for Anti-A sensitization, third trimester, fetus 1 |
| ICD-10-CM | O361132 | Maternal care for Anti-A sensitization, third trimester, fetus 2 |
| ICD-10-CM | O361133 | Maternal care for Anti-A sensitization, third trimester, fetus 3 |
| ICD-10-CM | O361134 | Maternal care for Anti-A sensitization, third trimester, fetus 4 |
| ICD-10-CM | O361135 | Maternal care for Anti-A sensitization, third trimester, fetus 5 |
| ICD-10-CM | O361139 | Maternal care for Anti-A sensitization, third trimester, other fetus |
| ICD-10-CM | O361190 | Maternal care for Anti-A sensitization, unspecified trimester, not applicable or unspecified |
| ICD-10-CM | O361191 | Maternal care for Anti-A sensitization, unspecified trimester, fetus 1 |
| ICD-10-CM | O361192 | Maternal care for Anti-A sensitization, unspecified trimester, fetus 2 |
| ICD-10-CM | O361193 | Maternal care for Anti-A sensitization, unspecified trimester, fetus 3 |
| ICD-10-CM | O361194 | Maternal care for Anti-A sensitization, unspecified trimester, fetus 4 |
| ICD-10-CM | O361195 | Maternal care for Anti-A sensitization, unspecified trimester, fetus 5 |
| ICD-10-CM | O361199 | Maternal care for Anti-A sensitization, unspecified trimester, other fetus |
| ICD-10-CM | O361910 | Maternal care for other isoimmunization, first trimester, not applicable or unspecified |
| ICD-10-CM | O361911 | Maternal care for other isoimmunization, first trimester, fetus 1 |
| ICD-10-CM | O361912 | Maternal care for other isoimmunization, first trimester, fetus 2 |
| ICD-10-CM | O361913 | Maternal care for other isoimmunization, first trimester, fetus 3 |
| ICD-10-CM | O361914 | Maternal care for other isoimmunization, first trimester, fetus 4 |
| ICD-10-CM | O361915 | Maternal care for other isoimmunization, first trimester, fetus 5 |
| ICD-10-CM | O361919 | Maternal care for other isoimmunization, first trimester, other fetus |
| ICD-10-CM | O361920 | Maternal care for other isoimmunization, second trimester, not applicable or unspecified |
| ICD-10-CM | O361921 | Maternal care for other isoimmunization, second trimester, fetus 1 |
| ICD-10-CM | O361922 | Maternal care for other isoimmunization, second trimester, fetus 2 |
| ICD-10-CM | O361923 | Maternal care for other isoimmunization, second trimester, fetus 3 |
| ICD-10-CM | O361924 | Maternal care for other isoimmunization, second trimester, fetus 4 |
| ICD-10-CM | O361925 | Maternal care for other isoimmunization, second trimester, fetus 5 |
| ICD-10-CM | O361929 | Maternal care for other isoimmunization, second trimester, other fetus |
| ICD-10-CM | O361930 | Maternal care for other isoimmunization, third trimester, not applicable or unspecified |
| ICD-10-CM | O361931 | Maternal care for other isoimmunization, third trimester, fetus 1 |
| ICD-10-CM | O361932 | Maternal care for other isoimmunization, third trimester, fetus 2 |
| ICD-10-CM | O361933 | Maternal care for other isoimmunization, third trimester, fetus 3 |
| ICD-10-CM | O361934 | Maternal care for other isoimmunization, third trimester, fetus 4 |
| ICD-10-CM | O361935 | Maternal care for other isoimmunization, third trimester, fetus 5 |
| ICD-10-CM | O361939 | Maternal care for other isoimmunization, third trimester, other fetus |
| ICD-10-CM | O361990 | Maternal care for other isoimmunization, unspecified trimester, not applicable or unspecified |
| ICD-10-CM | O361991 | Maternal care for other isoimmunization, unspecified trimester, fetus 1 |
| ICD-10-CM | O361992 | Maternal care for other isoimmunization, unspecified trimester, fetus 2 |
| ICD-10-CM | O361993 | Maternal care for other isoimmunization, unspecified trimester, fetus 3 |
| ICD-10-CM | O361994 | Maternal care for other isoimmunization, unspecified trimester, fetus 4 |
| ICD-10-CM | O361995 | Maternal care for other isoimmunization, unspecified trimester, fetus 5 |
| ICD-10-CM | O361999 | Maternal care for other isoimmunization, unspecified trimester, other fetus |
| ICD-10-CM | O3620X0 | Maternal care for hydrops fetalis, unspecified trimester, not applicable or unspecified |
| ICD-10-CM | O3620X1 | Maternal care for hydrops fetalis, unspecified trimester, fetus 1 |
| ICD-10-CM | O3620X2 | Maternal care for hydrops fetalis, unspecified trimester, fetus 2 |
| ICD-10-CM | O3620X3 | Maternal care for hydrops fetalis, unspecified trimester, fetus 3 |
| ICD-10-CM | O3620X4 | Maternal care for hydrops fetalis, unspecified trimester, fetus 4 |
| ICD-10-CM | O3620X5 | Maternal care for hydrops fetalis, unspecified trimester, fetus 5 |
| ICD-10-CM | O3620X9 | Maternal care for hydrops fetalis, unspecified trimester, other fetus |
| ICD-10-CM | O3621X0 | Maternal care for hydrops fetalis, first trimester, not applicable or unspecified |
| ICD-10-CM | O3621X1 | Maternal care for hydrops fetalis, first trimester, fetus 1 |
| ICD-10-CM | O3621X2 | Maternal care for hydrops fetalis, first trimester, fetus 2 |
| ICD-10-CM | O3621X3 | Maternal care for hydrops fetalis, first trimester, fetus 3 |
| ICD-10-CM | O3621X4 | Maternal care for hydrops fetalis, first trimester, fetus 4 |
| ICD-10-CM | O3621X5 | Maternal care for hydrops fetalis, first trimester, fetus 5 |
| ICD-10-CM | O3621X9 | Maternal care for hydrops fetalis, first trimester, other fetus |
| ICD-10-CM | O3622X0 | Maternal care for hydrops fetalis, second trimester, not applicable or unspecified |
| ICD-10-CM | O3622X1 | Maternal care for hydrops fetalis, second trimester, fetus 1 |
| ICD-10-CM | O3622X2 | Maternal care for hydrops fetalis, second trimester, fetus 2 |
| ICD-10-CM | O3622X3 | Maternal care for hydrops fetalis, second trimester, fetus 3 |
| ICD-10-CM | O3622X4 | Maternal care for hydrops fetalis, second trimester, fetus 4 |
| ICD-10-CM | O3622X5 | Maternal care for hydrops fetalis, second trimester, fetus 5 |
| ICD-10-CM | O3622X9 | Maternal care for hydrops fetalis, second trimester, other fetus |
| ICD-10-CM | O3623X0 | Maternal care for hydrops fetalis, third trimester, not applicable or unspecified |
| ICD-10-CM | O3623X1 | Maternal care for hydrops fetalis, third trimester, fetus 1 |
| ICD-10-CM | O3623X2 | Maternal care for hydrops fetalis, third trimester, fetus 2 |
| ICD-10-CM | O3623X3 | Maternal care for hydrops fetalis, third trimester, fetus 3 |
| ICD-10-CM | O3623X4 | Maternal care for hydrops fetalis, third trimester, fetus 4 |
| ICD-10-CM | O3623X5 | Maternal care for hydrops fetalis, third trimester, fetus 5 |
| ICD-10-CM | O3623X9 | Maternal care for hydrops fetalis, third trimester, other fetus |
| ICD-10-CM | O364XX0 | Maternal care for intrauterine death, not applicable or unspecified |
| ICD-10-CM | O364XX1 | Maternal care for intrauterine death, fetus 1 |
| ICD-10-CM | O364XX2 | Maternal care for intrauterine death, fetus 2 |
| ICD-10-CM | O364XX3 | Maternal care for intrauterine death, fetus 3 |
| ICD-10-CM | O364XX4 | Maternal care for intrauterine death, fetus 4 |
| ICD-10-CM | O364XX5 | Maternal care for intrauterine death, fetus 5 |
| ICD-10-CM | O364XX9 | Maternal care for intrauterine death, other fetus |
| ICD-10-CM | O365110 | Maternal care for known or suspected placental insufficiency, first trimester, not applicable or unspecified |
| ICD-10-CM | O365111 | Maternal care for known or suspected placental insufficiency, first trimester, fetus 1 |
| ICD-10-CM | O365112 | Maternal care for known or suspected placental insufficiency, first trimester, fetus 2 |
| ICD-10-CM | O365113 | Maternal care for known or suspected placental insufficiency, first trimester, fetus 3 |
| ICD-10-CM | O365114 | Maternal care for known or suspected placental insufficiency, first trimester, fetus 4 |
| ICD-10-CM | O365115 | Maternal care for known or suspected placental insufficiency, first trimester, fetus 5 |
| ICD-10-CM | O365119 | Maternal care for known or suspected placental insufficiency, first trimester, other fetus |
| ICD-10-CM | O365120 | Maternal care for known or suspected placental insufficiency, second trimester, not applicable or unspecified |
| ICD-10-CM | O365121 | Maternal care for known or suspected placental insufficiency, second trimester, fetus 1 |
| ICD-10-CM | O365122 | Maternal care for known or suspected placental insufficiency, second trimester, fetus 2 |
| ICD-10-CM | O365123 | Maternal care for known or suspected placental insufficiency, second trimester, fetus 3 |
| ICD-10-CM | O365124 | Maternal care for known or suspected placental insufficiency, second trimester, fetus 4 |
| ICD-10-CM | O365125 | Maternal care for known or suspected placental insufficiency, second trimester, fetus 5 |
| ICD-10-CM | O365129 | Maternal care for known or suspected placental insufficiency, second trimester, other fetus |
| ICD-10-CM | O365130 | Maternal care for known or suspected placental insufficiency, third trimester, not applicable or unspecified |
| ICD-10-CM | O365131 | Maternal care for known or suspected placental insufficiency, third trimester, fetus 1 |
| ICD-10-CM | O365132 | Maternal care for known or suspected placental insufficiency, third trimester, fetus 2 |
| ICD-10-CM | O365133 | Maternal care for known or suspected placental insufficiency, third trimester, fetus 3 |
| ICD-10-CM | O365134 | Maternal care for known or suspected placental insufficiency, third trimester, fetus 4 |
| ICD-10-CM | O365135 | Maternal care for known or suspected placental insufficiency, third trimester, fetus 5 |
| ICD-10-CM | O365139 | Maternal care for known or suspected placental insufficiency, third trimester, other fetus |
| ICD-10-CM | O365190 | Maternal care for known or suspected placental insufficiency, unspecified trimester, not applicable or unspecified |
| ICD-10-CM | O365191 | Maternal care for known or suspected placental insufficiency, unspecified trimester, fetus 1 |
| ICD-10-CM | O365192 | Maternal care for known or suspected placental insufficiency, unspecified trimester, fetus 2 |
| ICD-10-CM | O365193 | Maternal care for known or suspected placental insufficiency, unspecified trimester, fetus 3 |
| ICD-10-CM | O365194 | Maternal care for known or suspected placental insufficiency, unspecified trimester, fetus 4 |
| ICD-10-CM | O365195 | Maternal care for known or suspected placental insufficiency, unspecified trimester, fetus 5 |
| ICD-10-CM | O365199 | Maternal care for known or suspected placental insufficiency, unspecified trimester, other fetus |
| ICD-10-CM | O365910 | Maternal care for other known or suspected poor fetal growth, first trimester, not applicable or unspecified |
| ICD-10-CM | O365911 | Maternal care for other known or suspected poor fetal growth, first trimester, fetus 1 |
| ICD-10-CM | O365912 | Maternal care for other known or suspected poor fetal growth, first trimester, fetus 2 |
| ICD-10-CM | O365913 | Maternal care for other known or suspected poor fetal growth, first trimester, fetus 3 |
| ICD-10-CM | O365914 | Maternal care for other known or suspected poor fetal growth, first trimester, fetus 4 |
| ICD-10-CM | O365915 | Maternal care for other known or suspected poor fetal growth, first trimester, fetus 5 |
| ICD-10-CM | O365919 | Maternal care for other known or suspected poor fetal growth, first trimester, other fetus |
| ICD-10-CM | O365920 | Maternal care for other known or suspected poor fetal growth, second trimester, not applicable or unspecified |
| ICD-10-CM | O365921 | Maternal care for other known or suspected poor fetal growth, second trimester, fetus 1 |
| ICD-10-CM | O365922 | Maternal care for other known or suspected poor fetal growth, second trimester, fetus 2 |
| ICD-10-CM | O365923 | Maternal care for other known or suspected poor fetal growth, second trimester, fetus 3 |
| ICD-10-CM | O365924 | Maternal care for other known or suspected poor fetal growth, second trimester, fetus 4 |
| ICD-10-CM | O365925 | Maternal care for other known or suspected poor fetal growth, second trimester, fetus 5 |
| ICD-10-CM | O365929 | Maternal care for other known or suspected poor fetal growth, second trimester, other fetus |
| ICD-10-CM | O365930 | Maternal care for other known or suspected poor fetal growth, third trimester, not applicable or unspecified |
| ICD-10-CM | O365931 | Maternal care for other known or suspected poor fetal growth, third trimester, fetus 1 |
| ICD-10-CM | O365932 | Maternal care for other known or suspected poor fetal growth, third trimester, fetus 2 |
| ICD-10-CM | O365933 | Maternal care for other known or suspected poor fetal growth, third trimester, fetus 3 |
| ICD-10-CM | O365934 | Maternal care for other known or suspected poor fetal growth, third trimester, fetus 4 |
| ICD-10-CM | O365935 | Maternal care for other known or suspected poor fetal growth, third trimester, fetus 5 |
| ICD-10-CM | O365939 | Maternal care for other known or suspected poor fetal growth, third trimester, other fetus |
| ICD-10-CM | O365990 | Maternal care for other known or suspected poor fetal growth, unspecified trimester, not applicable or unspecified |
| ICD-10-CM | O365991 | Maternal care for other known or suspected poor fetal growth, unspecified trimester, fetus 1 |
| ICD-10-CM | O365992 | Maternal care for other known or suspected poor fetal growth, unspecified trimester, fetus 2 |
| ICD-10-CM | O365993 | Maternal care for other known or suspected poor fetal growth, unspecified trimester, fetus 3 |
| ICD-10-CM | O365994 | Maternal care for other known or suspected poor fetal growth, unspecified trimester, fetus 4 |
| ICD-10-CM | O365995 | Maternal care for other known or suspected poor fetal growth, unspecified trimester, fetus 5 |
| ICD-10-CM | O365999 | Maternal care for other known or suspected poor fetal growth, unspecified trimester, other fetus |
| ICD-10-CM | O3660X0 | Maternal care for excessive fetal growth, unspecified trimester, not applicable or unspecified |
| ICD-10-CM | O3660X1 | Maternal care for excessive fetal growth, unspecified trimester, fetus 1 |
| ICD-10-CM | O3660X2 | Maternal care for excessive fetal growth, unspecified trimester, fetus 2 |
| ICD-10-CM | O3660X3 | Maternal care for excessive fetal growth, unspecified trimester, fetus 3 |
| ICD-10-CM | O3660X4 | Maternal care for excessive fetal growth, unspecified trimester, fetus 4 |
| ICD-10-CM | O3660X5 | Maternal care for excessive fetal growth, unspecified trimester, fetus 5 |
| ICD-10-CM | O3660X9 | Maternal care for excessive fetal growth, unspecified trimester, other fetus |
| ICD-10-CM | O3661X0 | Maternal care for excessive fetal growth, first trimester, not applicable or unspecified |
| ICD-10-CM | O3661X1 | Maternal care for excessive fetal growth, first trimester, fetus 1 |
| ICD-10-CM | O3661X2 | Maternal care for excessive fetal growth, first trimester, fetus 2 |
| ICD-10-CM | O3661X3 | Maternal care for excessive fetal growth, first trimester, fetus 3 |
| ICD-10-CM | O3661X4 | Maternal care for excessive fetal growth, first trimester, fetus 4 |
| ICD-10-CM | O3661X5 | Maternal care for excessive fetal growth, first trimester, fetus 5 |
| ICD-10-CM | O3661X9 | Maternal care for excessive fetal growth, first trimester, other fetus |
| ICD-10-CM | O3662X0 | Maternal care for excessive fetal growth, second trimester, not applicable or unspecified |
| ICD-10-CM | O3662X1 | Maternal care for excessive fetal growth, second trimester, fetus 1 |
| ICD-10-CM | O3662X2 | Maternal care for excessive fetal growth, second trimester, fetus 2 |
| ICD-10-CM | O3662X3 | Maternal care for excessive fetal growth, second trimester, fetus 3 |
| ICD-10-CM | O3662X4 | Maternal care for excessive fetal growth, second trimester, fetus 4 |
| ICD-10-CM | O3662X5 | Maternal care for excessive fetal growth, second trimester, fetus 5 |
| ICD-10-CM | O3662X9 | Maternal care for excessive fetal growth, second trimester, other fetus |
| ICD-10-CM | O3663X0 | Maternal care for excessive fetal growth, third trimester, not applicable or unspecified |
| ICD-10-CM | O3663X1 | Maternal care for excessive fetal growth, third trimester, fetus 1 |
| ICD-10-CM | O3663X2 | Maternal care for excessive fetal growth, third trimester, fetus 2 |
| ICD-10-CM | O3663X3 | Maternal care for excessive fetal growth, third trimester, fetus 3 |
| ICD-10-CM | O3663X4 | Maternal care for excessive fetal growth, third trimester, fetus 4 |
| ICD-10-CM | O3663X5 | Maternal care for excessive fetal growth, third trimester, fetus 5 |
| ICD-10-CM | O3663X9 | Maternal care for excessive fetal growth, third trimester, other fetus |
| ICD-10-CM | O3670X0 | Maternal care for viable fetus in abdominal pregnancy, unspecified trimester, not applicable or unspecified |
| ICD-10-CM | O3670X1 | Maternal care for viable fetus in abdominal pregnancy, unspecified trimester, fetus 1 |
| ICD-10-CM | O3670X2 | Maternal care for viable fetus in abdominal pregnancy, unspecified trimester, fetus 2 |
| ICD-10-CM | O3670X3 | Maternal care for viable fetus in abdominal pregnancy, unspecified trimester, fetus 3 |
| ICD-10-CM | O3670X4 | Maternal care for viable fetus in abdominal pregnancy, unspecified trimester, fetus 4 |
| ICD-10-CM | O3670X5 | Maternal care for viable fetus in abdominal pregnancy, unspecified trimester, fetus 5 |
| ICD-10-CM | O3670X9 | Maternal care for viable fetus in abdominal pregnancy, unspecified trimester, other fetus |
| ICD-10-CM | O3671X0 | Maternal care for viable fetus in abdominal pregnancy, first trimester, not applicable or unspecified |
| ICD-10-CM | O3671X1 | Maternal care for viable fetus in abdominal pregnancy, first trimester, fetus 1 |
| ICD-10-CM | O3671X2 | Maternal care for viable fetus in abdominal pregnancy, first trimester, fetus 2 |
| ICD-10-CM | O3671X3 | Maternal care for viable fetus in abdominal pregnancy, first trimester, fetus 3 |
| ICD-10-CM | O3671X4 | Maternal care for viable fetus in abdominal pregnancy, first trimester, fetus 4 |
| ICD-10-CM | O3671X5 | Maternal care for viable fetus in abdominal pregnancy, first trimester, fetus 5 |
| ICD-10-CM | O3671X9 | Maternal care for viable fetus in abdominal pregnancy, first trimester, other fetus |
| ICD-10-CM | O3672X0 | Maternal care for viable fetus in abdominal pregnancy, second trimester, not applicable or unspecified |
| ICD-10-CM | O3672X1 | Maternal care for viable fetus in abdominal pregnancy, second trimester, fetus 1 |
| ICD-10-CM | O3672X2 | Maternal care for viable fetus in abdominal pregnancy, second trimester, fetus 2 |
| ICD-10-CM | O3672X3 | Maternal care for viable fetus in abdominal pregnancy, second trimester, fetus 3 |
| ICD-10-CM | O3672X4 | Maternal care for viable fetus in abdominal pregnancy, second trimester, fetus 4 |
| ICD-10-CM | O3672X5 | Maternal care for viable fetus in abdominal pregnancy, second trimester, fetus 5 |
| ICD-10-CM | O3672X9 | Maternal care for viable fetus in abdominal pregnancy, second trimester, other fetus |
| ICD-10-CM | O3673X0 | Maternal care for viable fetus in abdominal pregnancy, third trimester, not applicable or unspecified |
| ICD-10-CM | O3673X1 | Maternal care for viable fetus in abdominal pregnancy, third trimester, fetus 1 |
| ICD-10-CM | O3673X2 | Maternal care for viable fetus in abdominal pregnancy, third trimester, fetus 2 |
| ICD-10-CM | O3673X3 | Maternal care for viable fetus in abdominal pregnancy, third trimester, fetus 3 |
| ICD-10-CM | O3673X4 | Maternal care for viable fetus in abdominal pregnancy, third trimester, fetus 4 |
| ICD-10-CM | O3673X5 | Maternal care for viable fetus in abdominal pregnancy, third trimester, fetus 5 |
| ICD-10-CM | O3673X9 | Maternal care for viable fetus in abdominal pregnancy, third trimester, other fetus |
| ICD-10-CM | O3680X0 | Pregnancy with inconclusive fetal viability, not applicable or unspecified |
| ICD-10-CM | O3680X1 | Pregnancy with inconclusive fetal viability, fetus 1 |
| ICD-10-CM | O3680X2 | Pregnancy with inconclusive fetal viability, fetus 2 |
| ICD-10-CM | O3680X3 | Pregnancy with inconclusive fetal viability, fetus 3 |
| ICD-10-CM | O3680X4 | Pregnancy with inconclusive fetal viability, fetus 4 |
| ICD-10-CM | O3680X5 | Pregnancy with inconclusive fetal viability, fetus 5 |
| ICD-10-CM | O3680X9 | Pregnancy with inconclusive fetal viability, other fetus |
| ICD-10-CM | O368120 | Decreased fetal movements, second trimester, not applicable or unspecified |
| ICD-10-CM | O368121 | Decreased fetal movements, second trimester, fetus 1 |
| ICD-10-CM | O368122 | Decreased fetal movements, second trimester, fetus 2 |
| ICD-10-CM | O368123 | Decreased fetal movements, second trimester, fetus 3 |
| ICD-10-CM | O368124 | Decreased fetal movements, second trimester, fetus 4 |
| ICD-10-CM | O368125 | Decreased fetal movements, second trimester, fetus 5 |
| ICD-10-CM | O368129 | Decreased fetal movements, second trimester, other fetus |
| ICD-10-CM | O368130 | Decreased fetal movements, third trimester, not applicable or unspecified |
| ICD-10-CM | O368131 | Decreased fetal movements, third trimester, fetus 1 |
| ICD-10-CM | O368132 | Decreased fetal movements, third trimester, fetus 2 |
| ICD-10-CM | O368133 | Decreased fetal movements, third trimester, fetus 3 |
| ICD-10-CM | O368134 | Decreased fetal movements, third trimester, fetus 4 |
| ICD-10-CM | O368135 | Decreased fetal movements, third trimester, fetus 5 |
| ICD-10-CM | O368139 | Decreased fetal movements, third trimester, other fetus |
| ICD-10-CM | O368190 | Decreased fetal movements, unspecified trimester, not applicable or unspecified |
| ICD-10-CM | O368191 | Decreased fetal movements, unspecified trimester, fetus 1 |
| ICD-10-CM | O368192 | Decreased fetal movements, unspecified trimester, fetus 2 |
| ICD-10-CM | O368193 | Decreased fetal movements, unspecified trimester, fetus 3 |
| ICD-10-CM | O368194 | Decreased fetal movements, unspecified trimester, fetus 4 |
| ICD-10-CM | O368195 | Decreased fetal movements, unspecified trimester, fetus 5 |
| ICD-10-CM | O368199 | Decreased fetal movements, unspecified trimester, other fetus |
| ICD-10-CM | O368210 | Fetal anemia and thrombocytopenia, first trimester, not applicable or unspecified |
| ICD-10-CM | O368211 | Fetal anemia and thrombocytopenia, first trimester, fetus 1 |
| ICD-10-CM | O368212 | Fetal anemia and thrombocytopenia, first trimester, fetus 2 |
| ICD-10-CM | O368213 | Fetal anemia and thrombocytopenia, first trimester, fetus 3 |
| ICD-10-CM | O368214 | Fetal anemia and thrombocytopenia, first trimester, fetus 4 |
| ICD-10-CM | O368215 | Fetal anemia and thrombocytopenia, first trimester, fetus 5 |
| ICD-10-CM | O368219 | Fetal anemia and thrombocytopenia, first trimester, other fetus |
| ICD-10-CM | O368220 | Fetal anemia and thrombocytopenia, second trimester, not applicable or unspecified |
| ICD-10-CM | O368221 | Fetal anemia and thrombocytopenia, second trimester, fetus 1 |
| ICD-10-CM | O368222 | Fetal anemia and thrombocytopenia, second trimester, fetus 2 |
| ICD-10-CM | O368223 | Fetal anemia and thrombocytopenia, second trimester, fetus 3 |
| ICD-10-CM | O368224 | Fetal anemia and thrombocytopenia, second trimester, fetus 4 |
| ICD-10-CM | O368225 | Fetal anemia and thrombocytopenia, second trimester, fetus 5 |
| ICD-10-CM | O368229 | Fetal anemia and thrombocytopenia, second trimester, other fetus |
| ICD-10-CM | O368230 | Fetal anemia and thrombocytopenia, third trimester, not applicable or unspecified |
| ICD-10-CM | O368231 | Fetal anemia and thrombocytopenia, third trimester, fetus 1 |
| ICD-10-CM | O368232 | Fetal anemia and thrombocytopenia, third trimester, fetus 2 |
| ICD-10-CM | O368233 | Fetal anemia and thrombocytopenia, third trimester, fetus 3 |
| ICD-10-CM | O368234 | Fetal anemia and thrombocytopenia, third trimester, fetus 4 |
| ICD-10-CM | O368235 | Fetal anemia and thrombocytopenia, third trimester, fetus 5 |
| ICD-10-CM | O368239 | Fetal anemia and thrombocytopenia, third trimester, other fetus |
| ICD-10-CM | O368290 | Fetal anemia and thrombocytopenia, unspecified trimester, not applicable or unspecified |
| ICD-10-CM | O368291 | Fetal anemia and thrombocytopenia, unspecified trimester, fetus 1 |
| ICD-10-CM | O368292 | Fetal anemia and thrombocytopenia, unspecified trimester, fetus 2 |
| ICD-10-CM | O368293 | Fetal anemia and thrombocytopenia, unspecified trimester, fetus 3 |
| ICD-10-CM | O368294 | Fetal anemia and thrombocytopenia, unspecified trimester, fetus 4 |
| ICD-10-CM | O368295 | Fetal anemia and thrombocytopenia, unspecified trimester, fetus 5 |
| ICD-10-CM | O368299 | Fetal anemia and thrombocytopenia, unspecified trimester, other fetus |
| ICD-10-CM | O368310 | Maternal care for abnormalities of the fetal heart rate or rhythm, first trimester, not applicable or unspecified |
| ICD-10-CM | O368311 | Maternal care for abnormalities of the fetal heart rate or rhythm, first trimester, fetus 1 |
| ICD-10-CM | O368312 | Maternal care for abnormalities of the fetal heart rate or rhythm, first trimester, fetus 2 |
| ICD-10-CM | O368313 | Maternal care for abnormalities of the fetal heart rate or rhythm, first trimester, fetus 3 |
| ICD-10-CM | O368314 | Maternal care for abnormalities of the fetal heart rate or rhythm, first trimester, fetus 4 |
| ICD-10-CM | O368315 | Maternal care for abnormalities of the fetal heart rate or rhythm, first trimester, fetus 5 |
| ICD-10-CM | O368319 | Maternal care for abnormalities of the fetal heart rate or rhythm, first trimester, other fetus |
| ICD-10-CM | O368320 | Maternal care for abnormalities of the fetal heart rate or rhythm, second trimester, not applicable or unspecified |
| ICD-10-CM | O368321 | Maternal care for abnormalities of the fetal heart rate or rhythm, second trimester, fetus 1 |
| ICD-10-CM | O368322 | Maternal care for abnormalities of the fetal heart rate or rhythm, second trimester, fetus 2 |
| ICD-10-CM | O368323 | Maternal care for abnormalities of the fetal heart rate or rhythm, second trimester, fetus 3 |
| ICD-10-CM | O368324 | Maternal care for abnormalities of the fetal heart rate or rhythm, second trimester, fetus 4 |
| ICD-10-CM | O368325 | Maternal care for abnormalities of the fetal heart rate or rhythm, second trimester, fetus 5 |
| ICD-10-CM | O368329 | Maternal care for abnormalities of the fetal heart rate or rhythm, second trimester, other fetus |
| ICD-10-CM | O368330 | Maternal care for abnormalities of the fetal heart rate or rhythm, third trimester, not applicable or unspecified |
| ICD-10-CM | O368331 | Maternal care for abnormalities of the fetal heart rate or rhythm, third trimester, fetus 1 |
| ICD-10-CM | O368332 | Maternal care for abnormalities of the fetal heart rate or rhythm, third trimester, fetus 2 |
| ICD-10-CM | O368333 | Maternal care for abnormalities of the fetal heart rate or rhythm, third trimester, fetus 3 |
| ICD-10-CM | O368334 | Maternal care for abnormalities of the fetal heart rate or rhythm, third trimester, fetus 4 |
| ICD-10-CM | O368335 | Maternal care for abnormalities of the fetal heart rate or rhythm, third trimester, fetus 5 |
| ICD-10-CM | O368339 | Maternal care for abnormalities of the fetal heart rate or rhythm, third trimester, other fetus |
| ICD-10-CM | O368390 | Maternal care for abnormalities of the fetal heart rate or rhythm, unspecified trimester, not applicable or unspecified |
| ICD-10-CM | O368391 | Maternal care for abnormalities of the fetal heart rate or rhythm, unspecified trimester, fetus 1 |
| ICD-10-CM | O368392 | Maternal care for abnormalities of the fetal heart rate or rhythm, unspecified trimester, fetus 2 |
| ICD-10-CM | O368393 | Maternal care for abnormalities of the fetal heart rate or rhythm, unspecified trimester, fetus 3 |
| ICD-10-CM | O368394 | Maternal care for abnormalities of the fetal heart rate or rhythm, unspecified trimester, fetus 4 |
| ICD-10-CM | O368395 | Maternal care for abnormalities of the fetal heart rate or rhythm, unspecified trimester, fetus 5 |
| ICD-10-CM | O368399 | Maternal care for abnormalities of the fetal heart rate or rhythm, unspecified trimester, other fetus |
| ICD-10-CM | O368910 | Maternal care for other specified fetal problems, first trimester, not applicable or unspecified |
| ICD-10-CM | O368911 | Maternal care for other specified fetal problems, first trimester, fetus 1 |
| ICD-10-CM | O368912 | Maternal care for other specified fetal problems, first trimester, fetus 2 |
| ICD-10-CM | O368913 | Maternal care for other specified fetal problems, first trimester, fetus 3 |
| ICD-10-CM | O368914 | Maternal care for other specified fetal problems, first trimester, fetus 4 |
| ICD-10-CM | O368915 | Maternal care for other specified fetal problems, first trimester, fetus 5 |
| ICD-10-CM | O368919 | Maternal care for other specified fetal problems, first trimester, other fetus |
| ICD-10-CM | O368920 | Maternal care for other specified fetal problems, second trimester, not applicable or unspecified |
| ICD-10-CM | O368921 | Maternal care for other specified fetal problems, second trimester, fetus 1 |
| ICD-10-CM | O368922 | Maternal care for other specified fetal problems, second trimester, fetus 2 |
| ICD-10-CM | O368923 | Maternal care for other specified fetal problems, second trimester, fetus 3 |
| ICD-10-CM | O368924 | Maternal care for other specified fetal problems, second trimester, fetus 4 |
| ICD-10-CM | O368925 | Maternal care for other specified fetal problems, second trimester, fetus 5 |
| ICD-10-CM | O368929 | Maternal care for other specified fetal problems, second trimester, other fetus |
| ICD-10-CM | O368930 | Maternal care for other specified fetal problems, third trimester, not applicable or unspecified |
| ICD-10-CM | O368931 | Maternal care for other specified fetal problems, third trimester, fetus 1 |
| ICD-10-CM | O368932 | Maternal care for other specified fetal problems, third trimester, fetus 2 |
| ICD-10-CM | O368933 | Maternal care for other specified fetal problems, third trimester, fetus 3 |
| ICD-10-CM | O368934 | Maternal care for other specified fetal problems, third trimester, fetus 4 |
| ICD-10-CM | O368935 | Maternal care for other specified fetal problems, third trimester, fetus 5 |
| ICD-10-CM | O368939 | Maternal care for other specified fetal problems, third trimester, other fetus |
| ICD-10-CM | O368990 | Maternal care for other specified fetal problems, unspecified trimester, not applicable or unspecified |
| ICD-10-CM | O368991 | Maternal care for other specified fetal problems, unspecified trimester, fetus 1 |
| ICD-10-CM | O368992 | Maternal care for other specified fetal problems, unspecified trimester, fetus 2 |
| ICD-10-CM | O368993 | Maternal care for other specified fetal problems, unspecified trimester, fetus 3 |
| ICD-10-CM | O368994 | Maternal care for other specified fetal problems, unspecified trimester, fetus 4 |
| ICD-10-CM | O368995 | Maternal care for other specified fetal problems, unspecified trimester, fetus 5 |
| ICD-10-CM | O368999 | Maternal care for other specified fetal problems, unspecified trimester, other fetus |
| ICD-10-CM | O3690X0 | Maternal care for fetal problem, unspecified, unspecified trimester, not applicable or unspecified |
| ICD-10-CM | O3690X1 | Maternal care for fetal problem, unspecified, unspecified trimester, fetus 1 |
| ICD-10-CM | O3690X2 | Maternal care for fetal problem, unspecified, unspecified trimester, fetus 2 |
| ICD-10-CM | O3690X3 | Maternal care for fetal problem, unspecified, unspecified trimester, fetus 3 |
| ICD-10-CM | O3690X4 | Maternal care for fetal problem, unspecified, unspecified trimester, fetus 4 |
| ICD-10-CM | O3690X5 | Maternal care for fetal problem, unspecified, unspecified trimester, fetus 5 |
| ICD-10-CM | O3690X9 | Maternal care for fetal problem, unspecified, unspecified trimester, other fetus |
| ICD-10-CM | O3691X0 | Maternal care for fetal problem, unspecified, first trimester, not applicable or unspecified |
| ICD-10-CM | O3691X1 | Maternal care for fetal problem, unspecified, first trimester, fetus 1 |
| ICD-10-CM | O3691X2 | Maternal care for fetal problem, unspecified, first trimester, fetus 2 |
| ICD-10-CM | O3691X3 | Maternal care for fetal problem, unspecified, first trimester, fetus 3 |
| ICD-10-CM | O3691X4 | Maternal care for fetal problem, unspecified, first trimester, fetus 4 |
| ICD-10-CM | O3691X5 | Maternal care for fetal problem, unspecified, first trimester, fetus 5 |
| ICD-10-CM | O3691X9 | Maternal care for fetal problem, unspecified, first trimester, other fetus |
| ICD-10-CM | O3692X0 | Maternal care for fetal problem, unspecified, second trimester, not applicable or unspecified |
| ICD-10-CM | O3692X1 | Maternal care for fetal problem, unspecified, second trimester, fetus 1 |
| ICD-10-CM | O3692X2 | Maternal care for fetal problem, unspecified, second trimester, fetus 2 |
| ICD-10-CM | O3692X3 | Maternal care for fetal problem, unspecified, second trimester, fetus 3 |
| ICD-10-CM | O3692X4 | Maternal care for fetal problem, unspecified, second trimester, fetus 4 |
| ICD-10-CM | O3692X5 | Maternal care for fetal problem, unspecified, second trimester, fetus 5 |
| ICD-10-CM | O3692X9 | Maternal care for fetal problem, unspecified, second trimester, other fetus |
| ICD-10-CM | O3693X0 | Maternal care for fetal problem, unspecified, third trimester, not applicable or unspecified |
| ICD-10-CM | O3693X1 | Maternal care for fetal problem, unspecified, third trimester, fetus 1 |
| ICD-10-CM | O3693X2 | Maternal care for fetal problem, unspecified, third trimester, fetus 2 |
| ICD-10-CM | O3693X3 | Maternal care for fetal problem, unspecified, third trimester, fetus 3 |
| ICD-10-CM | O3693X4 | Maternal care for fetal problem, unspecified, third trimester, fetus 4 |
| ICD-10-CM | O3693X5 | Maternal care for fetal problem, unspecified, third trimester, fetus 5 |
| ICD-10-CM | O3693X9 | Maternal care for fetal problem, unspecified, third trimester, other fetus |
| ICD-10-CM | O401XX0 | Polyhydramnios, first trimester, not applicable or unspecified |
| ICD-10-CM | O401XX1 | Polyhydramnios, first trimester, fetus 1 |
| ICD-10-CM | O401XX2 | Polyhydramnios, first trimester, fetus 2 |
| ICD-10-CM | O401XX3 | Polyhydramnios, first trimester, fetus 3 |
| ICD-10-CM | O401XX4 | Polyhydramnios, first trimester, fetus 4 |
| ICD-10-CM | O401XX5 | Polyhydramnios, first trimester, fetus 5 |
| ICD-10-CM | O401XX9 | Polyhydramnios, first trimester, other fetus |
| ICD-10-CM | O402XX0 | Polyhydramnios, second trimester, not applicable or unspecified |
| ICD-10-CM | O402XX1 | Polyhydramnios, second trimester, fetus 1 |
| ICD-10-CM | O402XX2 | Polyhydramnios, second trimester, fetus 2 |
| ICD-10-CM | O402XX3 | Polyhydramnios, second trimester, fetus 3 |
| ICD-10-CM | O402XX4 | Polyhydramnios, second trimester, fetus 4 |
| ICD-10-CM | O402XX5 | Polyhydramnios, second trimester, fetus 5 |
| ICD-10-CM | O402XX9 | Polyhydramnios, second trimester, other fetus |
| ICD-10-CM | O403XX0 | Polyhydramnios, third trimester, not applicable or unspecified |
| ICD-10-CM | O403XX1 | Polyhydramnios, third trimester, fetus 1 |
| ICD-10-CM | O403XX2 | Polyhydramnios, third trimester, fetus 2 |
| ICD-10-CM | O403XX3 | Polyhydramnios, third trimester, fetus 3 |
| ICD-10-CM | O403XX4 | Polyhydramnios, third trimester, fetus 4 |
| ICD-10-CM | O403XX5 | Polyhydramnios, third trimester, fetus 5 |
| ICD-10-CM | O403XX9 | Polyhydramnios, third trimester, other fetus |
| ICD-10-CM | O409XX0 | Polyhydramnios, unspecified trimester, not applicable or unspecified |
| ICD-10-CM | O409XX1 | Polyhydramnios, unspecified trimester, fetus 1 |
| ICD-10-CM | O409XX2 | Polyhydramnios, unspecified trimester, fetus 2 |
| ICD-10-CM | O409XX3 | Polyhydramnios, unspecified trimester, fetus 3 |
| ICD-10-CM | O409XX4 | Polyhydramnios, unspecified trimester, fetus 4 |
| ICD-10-CM | O409XX5 | Polyhydramnios, unspecified trimester, fetus 5 |
| ICD-10-CM | O409XX9 | Polyhydramnios, unspecified trimester, other fetus |
| ICD-10-CM | O4100X0 | Oligohydramnios, unspecified trimester, not applicable or unspecified |
| ICD-10-CM | O4100X1 | Oligohydramnios, unspecified trimester, fetus 1 |
| ICD-10-CM | O4100X2 | Oligohydramnios, unspecified trimester, fetus 2 |
| ICD-10-CM | O4100X3 | Oligohydramnios, unspecified trimester, fetus 3 |
| ICD-10-CM | O4100X4 | Oligohydramnios, unspecified trimester, fetus 4 |
| ICD-10-CM | O4100X5 | Oligohydramnios, unspecified trimester, fetus 5 |
| ICD-10-CM | O4100X9 | Oligohydramnios, unspecified trimester, other fetus |
| ICD-10-CM | O4101X0 | Oligohydramnios, first trimester, not applicable or unspecified |
| ICD-10-CM | O4101X1 | Oligohydramnios, first trimester, fetus 1 |
| ICD-10-CM | O4101X2 | Oligohydramnios, first trimester, fetus 2 |
| ICD-10-CM | O4101X3 | Oligohydramnios, first trimester, fetus 3 |
| ICD-10-CM | O4101X4 | Oligohydramnios, first trimester, fetus 4 |
| ICD-10-CM | O4101X5 | Oligohydramnios, first trimester, fetus 5 |
| ICD-10-CM | O4101X9 | Oligohydramnios, first trimester, other fetus |
| ICD-10-CM | O4102X0 | Oligohydramnios, second trimester, not applicable or unspecified |
| ICD-10-CM | O4102X1 | Oligohydramnios, second trimester, fetus 1 |
| ICD-10-CM | O4102X2 | Oligohydramnios, second trimester, fetus 2 |
| ICD-10-CM | O4102X3 | Oligohydramnios, second trimester, fetus 3 |
| ICD-10-CM | O4102X4 | Oligohydramnios, second trimester, fetus 4 |
| ICD-10-CM | O4102X5 | Oligohydramnios, second trimester, fetus 5 |
| ICD-10-CM | O4102X9 | Oligohydramnios, second trimester, other fetus |
| ICD-10-CM | O4103X0 | Oligohydramnios, third trimester, not applicable or unspecified |
| ICD-10-CM | O4103X1 | Oligohydramnios, third trimester, fetus 1 |
| ICD-10-CM | O4103X2 | Oligohydramnios, third trimester, fetus 2 |
| ICD-10-CM | O4103X3 | Oligohydramnios, third trimester, fetus 3 |
| ICD-10-CM | O4103X4 | Oligohydramnios, third trimester, fetus 4 |
| ICD-10-CM | O4103X5 | Oligohydramnios, third trimester, fetus 5 |
| ICD-10-CM | O4103X9 | Oligohydramnios, third trimester, other fetus |
| ICD-10-CM | O411010 | Infection of amniotic sac and membranes, unspecified, first trimester, not applicable or unspecified |
| ICD-10-CM | O411011 | Infection of amniotic sac and membranes, unspecified, first trimester, fetus 1 |
| ICD-10-CM | O411012 | Infection of amniotic sac and membranes, unspecified, first trimester, fetus 2 |
| ICD-10-CM | O411013 | Infection of amniotic sac and membranes, unspecified, first trimester, fetus 3 |
| ICD-10-CM | O411014 | Infection of amniotic sac and membranes, unspecified, first trimester, fetus 4 |
| ICD-10-CM | O411015 | Infection of amniotic sac and membranes, unspecified, first trimester, fetus 5 |
| ICD-10-CM | O411019 | Infection of amniotic sac and membranes, unspecified, first trimester, other fetus |
| ICD-10-CM | O411020 | Infection of amniotic sac and membranes, unspecified, second trimester, not applicable or unspecified |
| ICD-10-CM | O411021 | Infection of amniotic sac and membranes, unspecified, second trimester, fetus 1 |
| ICD-10-CM | O411022 | Infection of amniotic sac and membranes, unspecified, second trimester, fetus 2 |
| ICD-10-CM | O411023 | Infection of amniotic sac and membranes, unspecified, second trimester, fetus 3 |
| ICD-10-CM | O411024 | Infection of amniotic sac and membranes, unspecified, second trimester, fetus 4 |
| ICD-10-CM | O411025 | Infection of amniotic sac and membranes, unspecified, second trimester, fetus 5 |
| ICD-10-CM | O411029 | Infection of amniotic sac and membranes, unspecified, second trimester, other fetus |
| ICD-10-CM | O411030 | Infection of amniotic sac and membranes, unspecified, third trimester, not applicable or unspecified |
| ICD-10-CM | O411031 | Infection of amniotic sac and membranes, unspecified, third trimester, fetus 1 |
| ICD-10-CM | O411032 | Infection of amniotic sac and membranes, unspecified, third trimester, fetus 2 |
| ICD-10-CM | O411033 | Infection of amniotic sac and membranes, unspecified, third trimester, fetus 3 |
| ICD-10-CM | O411034 | Infection of amniotic sac and membranes, unspecified, third trimester, fetus 4 |
| ICD-10-CM | O411035 | Infection of amniotic sac and membranes, unspecified, third trimester, fetus 5 |
| ICD-10-CM | O411039 | Infection of amniotic sac and membranes, unspecified, third trimester, other fetus |
| ICD-10-CM | O411090 | Infection of amniotic sac and membranes, unspecified, unspecified trimester, not applicable or unspecified |
| ICD-10-CM | O411091 | Infection of amniotic sac and membranes, unspecified, unspecified trimester, fetus 1 |
| ICD-10-CM | O411092 | Infection of amniotic sac and membranes, unspecified, unspecified trimester, fetus 2 |
| ICD-10-CM | O411093 | Infection of amniotic sac and membranes, unspecified, unspecified trimester, fetus 3 |
| ICD-10-CM | O411094 | Infection of amniotic sac and membranes, unspecified, unspecified trimester, fetus 4 |
| ICD-10-CM | O411095 | Infection of amniotic sac and membranes, unspecified, unspecified trimester, fetus 5 |
| ICD-10-CM | O411099 | Infection of amniotic sac and membranes, unspecified, unspecified trimester, other fetus |
| ICD-10-CM | O411210 | Chorioamnionitis, first trimester, not applicable or unspecified |
| ICD-10-CM | O411211 | Chorioamnionitis, first trimester, fetus 1 |
| ICD-10-CM | O411212 | Chorioamnionitis, first trimester, fetus 2 |
| ICD-10-CM | O411213 | Chorioamnionitis, first trimester, fetus 3 |
| ICD-10-CM | O411214 | Chorioamnionitis, first trimester, fetus 4 |
| ICD-10-CM | O411215 | Chorioamnionitis, first trimester, fetus 5 |
| ICD-10-CM | O411219 | Chorioamnionitis, first trimester, other fetus |
| ICD-10-CM | O411220 | Chorioamnionitis, second trimester, not applicable or unspecified |
| ICD-10-CM | O411221 | Chorioamnionitis, second trimester, fetus 1 |
| ICD-10-CM | O411222 | Chorioamnionitis, second trimester, fetus 2 |
| ICD-10-CM | O411223 | Chorioamnionitis, second trimester, fetus 3 |
| ICD-10-CM | O411224 | Chorioamnionitis, second trimester, fetus 4 |
| ICD-10-CM | O411225 | Chorioamnionitis, second trimester, fetus 5 |
| ICD-10-CM | O411229 | Chorioamnionitis, second trimester, other fetus |
| ICD-10-CM | O411230 | Chorioamnionitis, third trimester, not applicable or unspecified |
| ICD-10-CM | O411231 | Chorioamnionitis, third trimester, fetus 1 |
| ICD-10-CM | O411232 | Chorioamnionitis, third trimester, fetus 2 |
| ICD-10-CM | O411233 | Chorioamnionitis, third trimester, fetus 3 |
| ICD-10-CM | O411234 | Chorioamnionitis, third trimester, fetus 4 |
| ICD-10-CM | O411235 | Chorioamnionitis, third trimester, fetus 5 |
| ICD-10-CM | O411239 | Chorioamnionitis, third trimester, other fetus |
| ICD-10-CM | O411290 | Chorioamnionitis, unspecified trimester, not applicable or unspecified |
| ICD-10-CM | O411291 | Chorioamnionitis, unspecified trimester, fetus 1 |
| ICD-10-CM | O411292 | Chorioamnionitis, unspecified trimester, fetus 2 |
| ICD-10-CM | O411293 | Chorioamnionitis, unspecified trimester, fetus 3 |
| ICD-10-CM | O411294 | Chorioamnionitis, unspecified trimester, fetus 4 |
| ICD-10-CM | O411295 | Chorioamnionitis, unspecified trimester, fetus 5 |
| ICD-10-CM | O411299 | Chorioamnionitis, unspecified trimester, other fetus |
| ICD-10-CM | O411410 | Placentitis, first trimester, not applicable or unspecified |
| ICD-10-CM | O411411 | Placentitis, first trimester, fetus 1 |
| ICD-10-CM | O411412 | Placentitis, first trimester, fetus 2 |
| ICD-10-CM | O411413 | Placentitis, first trimester, fetus 3 |
| ICD-10-CM | O411414 | Placentitis, first trimester, fetus 4 |
| ICD-10-CM | O411415 | Placentitis, first trimester, fetus 5 |
| ICD-10-CM | O411419 | Placentitis, first trimester, other fetus |
| ICD-10-CM | O411420 | Placentitis, second trimester, not applicable or unspecified |
| ICD-10-CM | O411421 | Placentitis, second trimester, fetus 1 |
| ICD-10-CM | O411422 | Placentitis, second trimester, fetus 2 |
| ICD-10-CM | O411423 | Placentitis, second trimester, fetus 3 |
| ICD-10-CM | O411424 | Placentitis, second trimester, fetus 4 |
| ICD-10-CM | O411425 | Placentitis, second trimester, fetus 5 |
| ICD-10-CM | O411429 | Placentitis, second trimester, other fetus |
| ICD-10-CM | O411430 | Placentitis, third trimester, not applicable or unspecified |
| ICD-10-CM | O411431 | Placentitis, third trimester, fetus 1 |
| ICD-10-CM | O411432 | Placentitis, third trimester, fetus 2 |
| ICD-10-CM | O411433 | Placentitis, third trimester, fetus 3 |
| ICD-10-CM | O411434 | Placentitis, third trimester, fetus 4 |
| ICD-10-CM | O411435 | Placentitis, third trimester, fetus 5 |
| ICD-10-CM | O411439 | Placentitis, third trimester, other fetus |
| ICD-10-CM | O411490 | Placentitis, unspecified trimester, not applicable or unspecified |
| ICD-10-CM | O411491 | Placentitis, unspecified trimester, fetus 1 |
| ICD-10-CM | O411492 | Placentitis, unspecified trimester, fetus 2 |
| ICD-10-CM | O411493 | Placentitis, unspecified trimester, fetus 3 |
| ICD-10-CM | O411494 | Placentitis, unspecified trimester, fetus 4 |
| ICD-10-CM | O411495 | Placentitis, unspecified trimester, fetus 5 |
| ICD-10-CM | O411499 | Placentitis, unspecified trimester, other fetus |
| ICD-10-CM | O418X10 | Other specified disorders of amniotic fluid and membranes, first trimester, not applicable or unspecified |
| ICD-10-CM | O418X11 | Other specified disorders of amniotic fluid and membranes, first trimester, fetus 1 |
| ICD-10-CM | O418X12 | Other specified disorders of amniotic fluid and membranes, first trimester, fetus 2 |
| ICD-10-CM | O418X13 | Other specified disorders of amniotic fluid and membranes, first trimester, fetus 3 |
| ICD-10-CM | O418X14 | Other specified disorders of amniotic fluid and membranes, first trimester, fetus 4 |
| ICD-10-CM | O418X15 | Other specified disorders of amniotic fluid and membranes, first trimester, fetus 5 |
| ICD-10-CM | O418X19 | Other specified disorders of amniotic fluid and membranes, first trimester, other fetus |
| ICD-10-CM | O418X20 | Other specified disorders of amniotic fluid and membranes, second trimester, not applicable or unspecified |
| ICD-10-CM | O418X21 | Other specified disorders of amniotic fluid and membranes, second trimester, fetus 1 |
| ICD-10-CM | O418X22 | Other specified disorders of amniotic fluid and membranes, second trimester, fetus 2 |
| ICD-10-CM | O418X23 | Other specified disorders of amniotic fluid and membranes, second trimester, fetus 3 |
| ICD-10-CM | O418X24 | Other specified disorders of amniotic fluid and membranes, second trimester, fetus 4 |
| ICD-10-CM | O418X25 | Other specified disorders of amniotic fluid and membranes, second trimester, fetus 5 |
| ICD-10-CM | O418X29 | Other specified disorders of amniotic fluid and membranes, second trimester, other fetus |
| ICD-10-CM | O418X30 | Other specified disorders of amniotic fluid and membranes, third trimester, not applicable or unspecified |
| ICD-10-CM | O418X31 | Other specified disorders of amniotic fluid and membranes, third trimester, fetus 1 |
| ICD-10-CM | O418X32 | Other specified disorders of amniotic fluid and membranes, third trimester, fetus 2 |
| ICD-10-CM | O418X33 | Other specified disorders of amniotic fluid and membranes, third trimester, fetus 3 |
| ICD-10-CM | O418X34 | Other specified disorders of amniotic fluid and membranes, third trimester, fetus 4 |
| ICD-10-CM | O418X35 | Other specified disorders of amniotic fluid and membranes, third trimester, fetus 5 |
| ICD-10-CM | O418X39 | Other specified disorders of amniotic fluid and membranes, third trimester, other fetus |
| ICD-10-CM | O418X90 | Other specified disorders of amniotic fluid and membranes, unspecified trimester, not applicable or unspecified |
| ICD-10-CM | O418X91 | Other specified disorders of amniotic fluid and membranes, unspecified trimester, fetus 1 |
| ICD-10-CM | O418X92 | Other specified disorders of amniotic fluid and membranes, unspecified trimester, fetus 2 |
| ICD-10-CM | O418X93 | Other specified disorders of amniotic fluid and membranes, unspecified trimester, fetus 3 |
| ICD-10-CM | O418X94 | Other specified disorders of amniotic fluid and membranes, unspecified trimester, fetus 4 |
| ICD-10-CM | O418X95 | Other specified disorders of amniotic fluid and membranes, unspecified trimester, fetus 5 |
| ICD-10-CM | O418X99 | Other specified disorders of amniotic fluid and membranes, unspecified trimester, other fetus |
| ICD-10-CM | O4190X0 | Disorder of amniotic fluid and membranes, unspecified, unspecified trimester, not applicable or unspecified |
| ICD-10-CM | O4190X1 | Disorder of amniotic fluid and membranes, unspecified, unspecified trimester, fetus 1 |
| ICD-10-CM | O4190X2 | Disorder of amniotic fluid and membranes, unspecified, unspecified trimester, fetus 2 |
| ICD-10-CM | O4190X3 | Disorder of amniotic fluid and membranes, unspecified, unspecified trimester, fetus 3 |
| ICD-10-CM | O4190X4 | Disorder of amniotic fluid and membranes, unspecified, unspecified trimester, fetus 4 |
| ICD-10-CM | O4190X5 | Disorder of amniotic fluid and membranes, unspecified, unspecified trimester, fetus 5 |
| ICD-10-CM | O4190X9 | Disorder of amniotic fluid and membranes, unspecified, unspecified trimester, other fetus |
| ICD-10-CM | O4191X0 | Disorder of amniotic fluid and membranes, unspecified, first trimester, not applicable or unspecified |
| ICD-10-CM | O4191X1 | Disorder of amniotic fluid and membranes, unspecified, first trimester, fetus 1 |
| ICD-10-CM | O4191X2 | Disorder of amniotic fluid and membranes, unspecified, first trimester, fetus 2 |
| ICD-10-CM | O4191X3 | Disorder of amniotic fluid and membranes, unspecified, first trimester, fetus 3 |
| ICD-10-CM | O4191X4 | Disorder of amniotic fluid and membranes, unspecified, first trimester, fetus 4 |
| ICD-10-CM | O4191X5 | Disorder of amniotic fluid and membranes, unspecified, first trimester, fetus 5 |
| ICD-10-CM | O4191X9 | Disorder of amniotic fluid and membranes, unspecified, first trimester, other fetus |
| ICD-10-CM | O4192X0 | Disorder of amniotic fluid and membranes, unspecified, second trimester, not applicable or unspecified |
| ICD-10-CM | O4192X1 | Disorder of amniotic fluid and membranes, unspecified, second trimester, fetus 1 |
| ICD-10-CM | O4192X2 | Disorder of amniotic fluid and membranes, unspecified, second trimester, fetus 2 |
| ICD-10-CM | O4192X3 | Disorder of amniotic fluid and membranes, unspecified, second trimester, fetus 3 |
| ICD-10-CM | O4192X4 | Disorder of amniotic fluid and membranes, unspecified, second trimester, fetus 4 |
| ICD-10-CM | O4192X5 | Disorder of amniotic fluid and membranes, unspecified, second trimester, fetus 5 |
| ICD-10-CM | O4192X9 | Disorder of amniotic fluid and membranes, unspecified, second trimester, other fetus |
| ICD-10-CM | O4193X0 | Disorder of amniotic fluid and membranes, unspecified, third trimester, not applicable or unspecified |
| ICD-10-CM | O4193X1 | Disorder of amniotic fluid and membranes, unspecified, third trimester, fetus 1 |
| ICD-10-CM | O4193X2 | Disorder of amniotic fluid and membranes, unspecified, third trimester, fetus 2 |
| ICD-10-CM | O4193X3 | Disorder of amniotic fluid and membranes, unspecified, third trimester, fetus 3 |
| ICD-10-CM | O4193X4 | Disorder of amniotic fluid and membranes, unspecified, third trimester, fetus 4 |
| ICD-10-CM | O4193X5 | Disorder of amniotic fluid and membranes, unspecified, third trimester, fetus 5 |
| ICD-10-CM | O4193X9 | Disorder of amniotic fluid and membranes, unspecified, third trimester, other fetus |
| ICD-10-CM | O4200 | Premature rupture of membranes, onset of labor within 24 hours of rupture, unspecified weeks of gestation |
| ICD-10-CM | O42011 | Preterm premature rupture of membranes, onset of labor within 24 hours of rupture, first trimester |
| ICD-10-CM | O42012 | Preterm premature rupture of membranes, onset of labor within 24 hours of rupture, second trimester |
| ICD-10-CM | O42013 | Preterm premature rupture of membranes, onset of labor within 24 hours of rupture, third trimester |
| ICD-10-CM | O42019 | Preterm premature rupture of membranes, onset of labor within 24 hours of rupture, unspecified trimester |
| ICD-10-CM | O4202 | Full-term premature rupture of membranes, onset of labor within 24 hours of rupture |
| ICD-10-CM | O4210 | Premature rupture of membranes, onset of labor more than 24 hours following rupture, unspecified weeks of gestation |
| ICD-10-CM | O42111 | Preterm premature rupture of membranes, onset of labor more than 24 hours following rupture, first trimester |
| ICD-10-CM | O42112 | Preterm premature rupture of membranes, onset of labor more than 24 hours following rupture, second trimester |
| ICD-10-CM | O42113 | Preterm premature rupture of membranes, onset of labor more than 24 hours following rupture, third trimester |
| ICD-10-CM | O42119 | Preterm premature rupture of membranes, onset of labor more than 24 hours following rupture, unspecified trimester |
| ICD-10-CM | O4212 | Full-term premature rupture of membranes, onset of labor more than 24 hours following rupture |
| ICD-10-CM | O4290 | Premature rupture of membranes, unspecified as to length of time between rupture and onset of labor, unspecified weeks of gestation |
| ICD-10-CM | O42911 | Preterm premature rupture of membranes, unspecified as to length of time between rupture and onset of labor, first trimester |
| ICD-10-CM | O42912 | Preterm premature rupture of membranes, unspecified as to length of time between rupture and onset of labor, second trimester |
| ICD-10-CM | O42913 | Preterm premature rupture of membranes, unspecified as to length of time between rupture and onset of labor, third trimester |
| ICD-10-CM | O42919 | Preterm premature rupture of membranes, unspecified as to length of time between rupture and onset of labor, unspecified trimester |
| ICD-10-CM | O4292 | Full-term premature rupture of membranes, unspecified as to length of time between rupture and onset of labor |
| ICD-10-CM | O43011 | Fetomaternal placental transfusion syndrome, first trimester |
| ICD-10-CM | O43012 | Fetomaternal placental transfusion syndrome, second trimester |
| ICD-10-CM | O43013 | Fetomaternal placental transfusion syndrome, third trimester |
| ICD-10-CM | O43019 | Fetomaternal placental transfusion syndrome, unspecified trimester |
| ICD-10-CM | O43021 | Fetus-to-fetus placental transfusion syndrome, first trimester |
| ICD-10-CM | O43022 | Fetus-to-fetus placental transfusion syndrome, second trimester |
| ICD-10-CM | O43023 | Fetus-to-fetus placental transfusion syndrome, third trimester |
| ICD-10-CM | O43029 | Fetus-to-fetus placental transfusion syndrome, unspecified trimester |
| ICD-10-CM | O43101 | Malformation of placenta, unspecified, first trimester |
| ICD-10-CM | O43102 | Malformation of placenta, unspecified, second trimester |
| ICD-10-CM | O43103 | Malformation of placenta, unspecified, third trimester |
| ICD-10-CM | O43109 | Malformation of placenta, unspecified, unspecified trimester |
| ICD-10-CM | O43111 | Circumvallate placenta, first trimester |
| ICD-10-CM | O43112 | Circumvallate placenta, second trimester |
| ICD-10-CM | O43113 | Circumvallate placenta, third trimester |
| ICD-10-CM | O43119 | Circumvallate placenta, unspecified trimester |
[truncated: 322,772 more chars]
